# Synthesis of 1,4-Dialkoxynaphthalene-Based Imidazolium Salts and Their Cytotoxicity in Cancer Cell Lines

**DOI:** 10.3390/ijms24032713

**Published:** 2023-02-01

**Authors:** Haena Lee, Yejin Jeon, Hyejin Moon, Eunjoo H. Lee, Tae Hoon Lee, Hakwon Kim

**Affiliations:** 1 Department of Applied Chemistry, Global Center for Pharmaceutical Ingredient Materials, Kyung Hee University, Yongin 1732, Republic of Korea; 2 Department of Gerontology, Graduate School of East-West Medicinal Science, Kyung Hee University, Yongin 17104, Republic of Korea

**Keywords:** imidazole, 1,4-dialkoxynaphthalne, cancer, cytotoxicity, HepG2, HT-29, CCD-18Co

## Abstract

In this study, we designed and synthesized novel 1,4-dialkoxynaphthalene-2-alkyl imidazolium salt (IMS) derivatives containing both 1,4-dialkoxynaphthalene and imidazole, which are well known as pharmacophores. The cytotoxicities of these newly synthesized IMS derivatives were investigated in order to explore the possibility of using them to develop anticancer drugs. It was found that some of the new IMS derivatives showed good cytotoxic activities. In addition, an initial, qualitative structure–activity relationship is presented on the basis of observations of activity changes corresponding to structural changes.

## 1. Introduction

Cancer is one of the leading causes of death worldwide. In 2020, there were approximately 10 million cancer deaths and approximately 19.3 million new types of cancer reported [1]. Uncontrolled cell proliferation is the hallmark of cancer; tumor cells typically acquire damage to genes that directly regulate their cell cycles [2,3]. In addition, cancer is known to develop due to genetic and environmental factors [4]. Among the various known cancer cells, HepG2, a human liver carcinoma hepatocellular cell, has a mutation in the CTNNB1 gene [5], and HT-29, a human colon adenocarcinoma colorectal cell, has a mutation in the BRAF (V600E) gene [6]. The use of such established cell culture lines is known to be beneficial when conducting cytotoxicity evaluation studies [7]. In this respect, the HepG2, HT-29, and CCD-18Co cell lines used in this study are well-differentiated, transformed cell lines that meet the biochemical requirements. Since the HepG2 cell line is one of the experimental models similar to human hepatocytes, it is widely used in liver cancer research, e.g., for cytotoxicity evaluation [8]. In addition, the HT-29 and the CCD-18Co cell lines are widely used in colon cancer research as models for comparing the difference in activity between cancer cells and normal cells [9]. Various methods are used to treat these cancers. In the past, radiation therapy or chemotherapy was often combined with surgery. However, since radiation and chemotherapy affect both normal cells and cancer cells, they cause serious side-effects, such as vomiting and hair loss. Therefore, in recent years, the development of targeted cancer treatment that interferes with a specific protein involved in cancer cell growth has emerged [10,11].

Imidazole is a five-membered heterocyclic compound containing two nitrogen atoms and is known to have a number of advantageous physical and biological properties. Imidazole exhibits various pharmacological activities, such as anticancer, antifungal, anticoagulant, and antimalarial, and it has been developed as a chemotherapeutic agent in various fields as a function of these activities [12,13]. Its anticancer efficacy was proven with the development of dacarbazine, and several other imidazole-based anticancer drugs have since been synthesized [14]. Studies aim to develop anticancer drugs that can increase anticancer effects and reduce side-effects [15].

**Figure 1:**
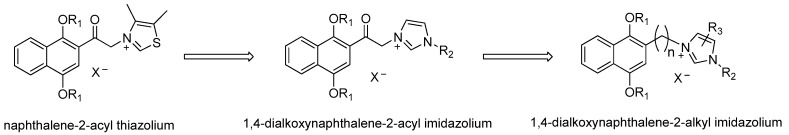
Design of new 1,4-dialkoxynaphthalene-2-alkyl imidazolium salt (IMS).

In our previous study [Figure 1], various naphthalene-2-acyl thiazolium salts combining the structures of 1,4-dialkoxynaphthalene and thiazole were synthesized, and their activities as AGE (advanced glycation end product) breakers were tested [16]. In this work, the 1,4-dialkoxynaphthalene moiety was found to be of pharmacological significance. Subsequently, an imidazole ring was introduced instead of thiazole to produce 1,4-dialkoxynaphthalene-2-acyl imidazolium salt derivatives, which were confirmed to exhibit antifungal activity [17]. Here, it was confirmed that the combination of the 1,4-dialkoxynaphthalene moiety and imidazolium moiety shows a good pharmacophore. However, there were several problems in the synthesis process of previous studies. The yield in this synthesis was highly dependent on the type of alkoxy group on the naphthalene ring and on the bromination step to produce monobromide, as well as dibromide and tribromide. Therefore, to find a compound that is easy to synthesize and can exhibit various activities, a new target compound was designed and synthesized in which the acyl moiety connecting the naphthalene ring and the imidazole ring was substituted with an alkyl moiety.

**Figure 2:**
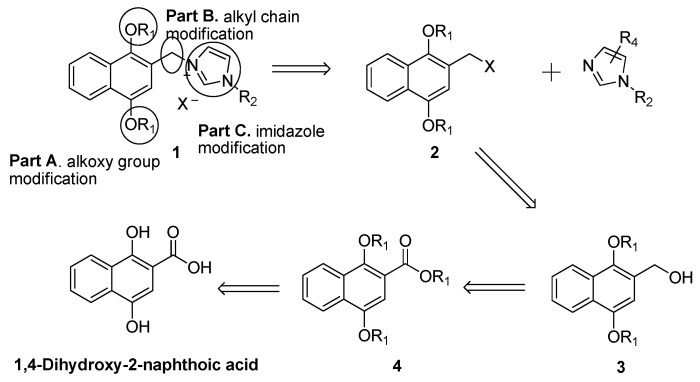
Retrosynthesis of 1,4-dialkoxylnaphthalene-2-alkyl imidazolium salt (IMS).

In this study, the synthesis of a novel 1,4-dialkoxynaphthalene-2-alkyl imidazolium salt (IMS) is described. The synthesis of the target compound IMS, 1, can be initiated from 1,4-dihydroxy-2-naphthoic acid via alkylation, reduction, bromination, and imidazole substitution [Figure 2]. The synthetic approach to new IMS derivatives is divided into three parts. In Part A, compound synthesis is carried out by changing the alkoxy group of the naphthalene ring. In Part B, derivatives are synthesized by modifying the alkyl moiety connecting the naphthalene ring and imidazole ring. Lastly, in Part C, the synthesis of various derivatives is attempted by changing the position and type of substituent attached to imidazole ring. To investigate the potential of theses derivatives as anticancer agents, their cytotoxicities against HepG2, HT-29, and CCD-18Co cells were evaluated. On the basis of the cytotoxicity results of the newly synthesized IMS compounds, we report the possibility of developing one such compound as a new anticancer agent.

## 2. Results and Discussion

### 2.1. Chemistry: Synthesis

#### 2.1.1. Part A: Modification of Alkoxy Groups in the Naphthalene Ring

The activity of acyl imidazolium salt is best when the isoamyloxy group is substituted [17]. To confirm that the same result can be obtained in an alkyl imidazolium salt, derivatives substituted with various alkoxy groups in the naphthalene ring were synthesized. After synthesizing the corresponding bromide intermediates in three steps from 1-hydroxy-2-naphthoic acid, methyl 4-hydroxy-2-naphthoate, or 1,4-dihydroxy-2-naphthoic acid (see Scheme S1, Figures S1–S24), **IMS-01** to **IMS-09** (see Figures S43–S51) were obtained through a subsequent substitution reaction with 1-benzylimidazole [Figure 3].

#### 2.1.2. Part B: Modification of Alkyl Chain between Naphthalene Ring and Imidazole Ring

**Scheme 1:**
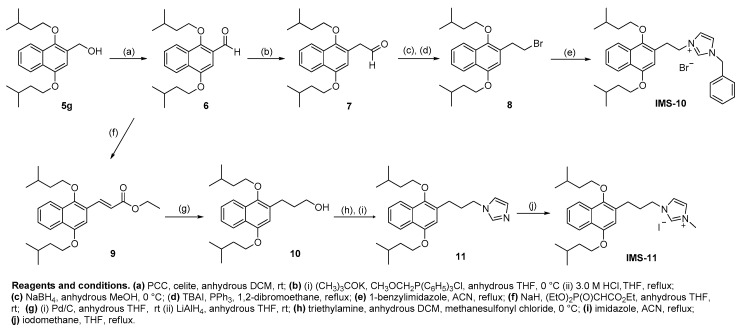
Synthesis of IMS-10 and IMS-11.

In **Part B**, the difference in activity as a function of the length of the carbon chain connecting the naphthalene ring and the imidazole ring was investigated. For comparison with **IMS-07**, **IMS-10** and **IMS-11**, each having an ethylene and propylene moiety between the naphthalene ring and the imidazole ring, were synthesized [Scheme 1]. First, to synthesize **IMS-10,** synthesis of key aldehyde intermediate **7**, in which the number of carbon atoms is increased by one, was attempted through a reduction reaction using DIBAL-H after synthesizing the corresponding cyanide intermediate from **12g** [18]. However, this approach did not proceed well. Therefore, we attempted to synthesize intermediate **7** via another method. Aldehyde intermediate **6** was synthesized through PCC oxidation of compound **5g**, and the subsequent Wittig reaction of **6** followed by acidic workup yielded key aldehyde intermediate **7** [19]. Intermediate **7** was reduced with NaBH_4_ and brominated with PBr_3_ to obtain bromide intermediate **8**. However, it was observed that this reaction is inefficient due to its long reaction time and low yield. To overcome this problem, a bromination reaction based on Xiao’s method using Ph_3_P, TBAI (tetrabutylammonium iodide), and 1,2-dibromoethane was adopted to obtain compound **8** in higher yield [20]. Even for bromide intermediates with low yields in **Part A**, the yield could be increased by using this method instead of PBr_3_. Finally, a substitution reaction between bromide **8** and 1-benzylimidazole gave the desired final product, **IMS-10** (see Figures S25–S27 and S52). Next, for the synthesis of **IMS-11**, ester intermediate **9**, which had its number of carbons increased by two compared to **IMS-07**, was prepared through an HWE (Horner–Wadsworth–Emmons) reaction of aldehyde **6 [21]**. Then, from intermediate **9**, alcohol intermediate **10** was obtained by Pd-catalyzed hydrogenation followed by LiAlH_4_ reduction. Halogenation of intermediate **10** provided the corresponding bromide in poor yield, which also did not react well with 1-benzylmidazole. Therefore, we made another attempt at synthesizing imidazolium salt. In order to change the hydroxy group to a better leaving group, mesylation was performed to obtain a mesylate [22]. Then, imidazole was attached to synthesize imidazole intermediate **11**. Intermediate **11** was then reacted with iodomethane to give the *N*-methylimidazolium salt, **IMS-11** (see Figures S28–S30 and S53) [23].

#### 2.1.3. Part C: Modification of Imidazole Ring

**Scheme 2:**
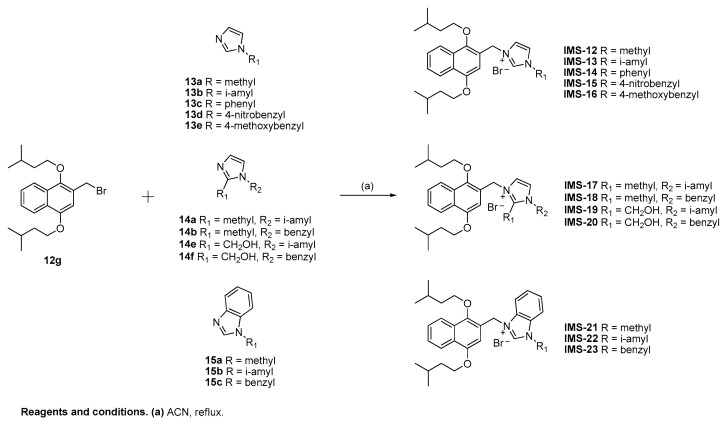
Synthesis of IMS-12 to IMS-23.

We synthesized IMS derivatives in which various substituents were substituted on the imidazole ring [Scheme 2]. First, derivatives were synthesized with substituents other than a benzyl group attached at the nitrogen position. The substituted imidazoles used were commercially or readily available, such as 1-methyl-1*H*-imidazole (**13a**) and 1-phenyl-1*H*-imidazole (**13c**), or other synthesized *N*-substituted imidazoles (**13b**, **13d**, and **13e**) (see Scheme S2, Figures S31–S33). Subsequently, **IMS-12**, **IMS-13**, **IMS-14**, **IMS-15**, and **IMS-16** (see Figures S54–S58) were obtained through a substitution reaction with bromide intermediate **12g**. Second, we tried to synthesize derivatives with a substituent attached to the position 2 of imidazole. *N*-Alkylation was performed using 2-methylimidazole and 2-imidazolecarboxaldehyde (see Scheme S2, Figures S34–S39). However, the derivatives synthesized using 2-imidazolecarboxaldehyde (**14c** and **14d**) easily decomposed during the purification process. Therefore, a change was made to synthesize the salt derivatives using 2-imidazolomethanols, **14e** and **14f**, through a reduction reaction (see Figures S59–S62). Lastly, we tried to introduce benzimidazole to synthesize derivatives substituted at both positions 4 and 5 on the imidazole ring. After *N*-alkylation (see Scheme S2, Figures S40–S42), the synthesis of the derivatives (**IMS-21**—**IMS-23**) was completed through a subsequent substitution reaction with the bromide intermediate **12g** (see Figures S63–S65).

### 2.2. Cytotoxicity of HepG2, HT-29, and CCD-18Co Cells

As shown in the cytotoxicity results (Table 1), **IMS-01 to IMS-06, IMS-08**, and **IMS-09** were not cytotoxic to HepG2, HT-29, and CCD-18Co cells when the maximum concentration of the compound was 10 μM. Therefore, compounds without an alkoxy group on the naphthalene ring (**IMS-09**) or compounds with a short or overly long alkoxy group (**IMS-01**, **IMS-03**, **IMS-05**, **IMS-06**, and **IMS-08**) did not show cytotoxicity. Even compounds with only one isoamyloxy group, which are considered optimal alkyl groups at positions 1 or 4 of the naphthalene ring, such as **IMS-02** and **IMS-04**, showed no activity. As a first conclusion, it was found that the isoamyloxy group is most suitable as an alkyl group substituted on the naphthalene ring, but it must be present at both positions 1 and 4 of the naphthalene ring.

In the remaining compounds, LD_50_ values were generally higher than 5 μM in HepG2 and HT-29 cells, and especially in CCD-18Co cells. All compounds, except for **IMS-11**, **IMS-13**, **IMS-15**, and **IMS-16,** exhibited LD_50_ values at low concentrations of 5 μM or less. Looking at the cytotoxicity results for HepG2, HT-29, and CCD-18Co of three compounds (**IMS-07**, **IMS-10**, and **IMS-11**) with different numbers of bridged carbon atoms between the naphthalene ring and the imidazole ring, it can be seen that there was no significant dependence on the length of alkyl chain. However, considering the ease of synthesis, the methylene-containing backbone in **IMS-07** was selected as the basic structure. When comparing the activities of **IMS-12**, **IMS-13**, **IMS-14**, **IMS-15**, and **IMS-16** with those of **IMS-07**, it was confirmed that all these compounds showed good activity. The results from **IMS-17, IMS-18, IMS-19**, and **IMS-20** demonstrate that derivatives with a substituent attached to position 2 of the imidazole ring also exhibited similar cytotoxicities to that of **IMS-07**. As shown in the results from **IMS-21**, **IMS-22**, and **IMS-23**, benzimidazolium salts substituted at positions 4 and 5 of the imidazole ring showed good activity without significant differences. It was confirmed that a similar activity was exhibited even when the type of alkyl group attached to the *N*-position was changed (see Figures S66–S68).

## 3. Materials and Methods

### 3.1. Chemicals and Instruments

All glassware was thoroughly dried in a convection oven. Reactions were monitored using thin-layer chromatography (TLC). Commercial TLC plates (silica gel 60 F254, Merck, Darmstadt, Germany) were developed, and the spots were visualized under UV light at 254 or 365 nm. Some products were purified by flash column chromatography using 230–400 mesh ASTM (Merck KGaA) silica gel or recrystallization using a combination of various solvents. Extra pure-grade solvents for column chromatography were purchased through Samchun Chemicals (Seoul, Korea) and Duksan Chemicals (Incheon, Korea). ^1^H- and ^13^C-NMR spectra were collected with a JEOL JNM-ECZ400S (at 400 MHz for ^1^H-NMR and 100 MHz for ^13^C-NMR). In ^1^H-NMR spectra, chemical shifts were expressed in parts per million (ppm) downfield from tetramethylsilane, and coupling constants were reported in hertz (Hz). Splitting patterns are indicated as follows: s, singlet; d, doublet; t, triplet; m, multiplet. ^13^C-NMR spectra were reported in ppm, referenced to chloroform-*d* and DMSO-*d_6_*. Melting points (m.p.) were determined on a Barnstead Electrothermal 9100 instrument and were uncorrected. High-resolution mass spectra were obtained with a JEOL JMS-700 mass spectrometer. All chemical reagents were acquired from Acros Organics (Brookline, MA, USA), Aldrich (St. Louis, MO, USA), or TCI (Tokyo, Japan) and were used as received. 1-Methylimidazole and 1-benzylimidazole from Sigma-Aldrich (St. Louis, MO, USA), and 1-phenylimidazole from TCI (Tokyo, Japan) are commercially available.

### 3.2. Intermediate Synthesis Process for the Synthesis of IMS-10 and IMS-11

**1,4-Bis(isoamyloxy)-2-naphthaldehyde (** **6** **)**

To **5g** (0.88 g, 2.68 mmol) in anhydrous dichloromethane (27 mL), celite (1.76 g) and PCC (0.87 g, 4.02 mmol) were added. It was stirred at room temperature until the starting material disappeared. When the completion of the reaction as monitored by TLC, the mixture was filtered through celite, and then washed with water, 1.0 M NaHCO_3_, and brine. It was dried over Na_2_SO_4_, filtered, and concentrated under reduced pressure. The concentrate was purified by column chromatography to give compound **6** as a yellow oil (85%). R_f_ = 0.71 (5% EtOAc/hexane); ^1^H-NMR (400 MHz, chloroform-*d*) δ (ppm): 1.01 (d, *J* = 6.8 Hz, 12H), 1.80–1.89 (m, 4H), 1.91–1.98 (m, 2H), 4.15 (t, *J* = 6.8 Hz, 2H), 4.19 (t, *J* = 6.6 Hz, 2H), 7.11 (s, 1H), 7.58–7.66 (m, 2H), 8.16–8.19 (m, 1H), 8.29–8.31 (m, 1H), 10.56 (s, 1H); ^13^C-NMR (100 MHz, chloroform-*d*) δ (ppm): 22.63, 22.69, 25.00, 25.28, 37.90, 39.00, 66.83, 77.66, 98.86, 122.96, 123.06, 124.91, 127.15, 128.74, 128.87, 130.48, 151.56, 156.14, 189.92.

**2-(1,4-Bis(isoamyloxy)naphthalen-2-yl)acetaldehyde (** **7** **)**

(Methoxymethyl)triphenyl-phosphonium chloride (1.54 g, 4.5 mmol) was dissolved in anhydrous THF (5 mL), and potassium *tert*-butoxide (1.0 M solution in THF, 6 mL, 6.0 mmol) was slowly dropwise at 0 °C. After 1 or 2 h, the solution of **6** (0.99 g, 3.0 mmol) in THF (5 mL) was added dropwise, followed by stirring at 0 °C until the starting material disappeared. The reaction was quenched with water and extracted with ethyl acetate three times. The combined organic layer was washed with brine, dried over Na_2_SO_4_, filtered, and then concentrated under reduced pressure. The concentrate was purified by column chromatography to give a yellow oily methoxyvinyl compound as an E/Z mixture (72%). Crude methoxyvinyl compound (0.73 g, 2.1 mmol) was dissolved in THF (14 mL), and then 3.0 M HCl (1.4 mL, 4.3 mmol) was slowly added dropwise. It was stirred at 60 °C until the starting material disappeared. The reaction was quenched with saturated aqueous solutions of NaHCO_3_ (10.5 mL). After evaporating THF solvent, water was added to the concentrate, followed by extraction with ethyl acetate three times. The combined organic layer was washed with brine, dried over Na_2_SO_4_, filtered, and then concentrated under reduced pressure. The concentrate was purified by column chromatography to give compound **7** as a brown oil (73%). R_f_ = 0.24 (5% EtOAc/hexane); ^1^H-NMR (400 MHz, chloroform-*d*) δ (ppm): 0.99–1.02 (m, 12H), 1.77–1.84 (m, 4H), 1.86–1.99 (m, 2H), 3.83 (d, *J* = 2.4 Hz, 2H), 3.91 (t, *J* = 6.8 Hz, 2H), 4.12 (t, *J* = 6.6 Hz, 2H), 6.53 (s, 1H), 7.46–7.56 (m, 2H), 8.00–8.03 (m, 1H), 8.25–8.27 (m, 1H), 9.79 (t, *J* = 2.4 Hz, 1H); ^13^C-NMR (100 MHz, chloroform-*d*) δ (ppm): 22.65, 22.72, 25.03, 25.25, 38.02, 39.08, 45.66, 66.71, 73.34, 106.12, 120.29, 121.76, 122.57, 125.41, 126.38, 126.74, 128.80, 147.04, 151.58, 199.85.

**2-(2-Bromoethyl)-1,4-bis(isoamyloxy)naphthalene (8)**

**Compound 7** (0.53 g, 1.55 mmol) was dissolved in anhydrous MeOH (15 mL) and cooled to 0 °C. After adding NaBH_4_ (88 mg, 2.32 mmol) slowly, it was stirred at 0 °C until the starting material disappeared. After the reaction was completed, the solution was concentrated under reduced pressure and was poured into water, followed by extraction with ethyl acetate three times. The combined organic layer was washed with brine, dried over Na_2_SO_4_, filtered, and then concentrated under reduced pressure. The concentrate was purified by column chromatography to give an ethanol intermediate. Ethanol intermediate (0.18 g, 0.51 mmol), PPh_3_ (0.16 g, 0.61 mmol), and TBAI (0.23 g, 0.61 mmol) in 1,2-dibromoethane (4.2 mL) were reacted as described in **method A** in the Supplementary Materials to give a bromo compound **8** as a yellow oil (56%). R_f_ = 0.64 (20% EtOAc/hexane); ^1^H-NMR (400 MHz, chloroform-*d*) δ (ppm): 1.01–1.03 (m, 12H), 1.79–1.86 (m, 4H), 1.90–2.00 (m, 2H), 3.32 (t, *J* = 8.0 Hz, 2H), 3.65 (t, *J* = 8.0 Hz, 2H), 3.96 (t, *J* = 6.8 Hz, 2H), 4.13 (t, *J* = 6.4 Hz, 2H), 6.60 (s, 1H), 7.43–7.47 (m, 1H), 7.50–7.54 (m, 1H), 7.98–8.00 (m, 1H), 8.22–8.24 (m, 1H); ^13^C-NMR (100 MHz, chloroform-*d*) δ (ppm): 22.67, 22.76, 25.08, 25.25, 31.93, 34.72, 38.07, 39.33, 66.68, 73.47, 105.92, 121.82, 122.46, 125.10, 126.06, 126.52, 126.73, 128.88, 146.45, 151.29.

**Ethyl 3-(1,4-bis(isoamyloxy)naphthalen-2-yl)acrylate (9)**

A suspension of NaH (60% dispersion in oil, 0.11 g, 4.4 mmol) in hexane was stirred for 10 min, and the solvent was syringed out. The oil-free NaH was then suspended in anhydrous THF (2 mL) and cooled in an ice bath. Triethyl phosphonoacetate (0.87 mL, 4.4 mmol) was added dropwise, and the reaction mixture was stirred for 30 min at room temperature. To the reaction mixture, a solution of **6** (0.72 g, 2.2 mmol) in anhydrous THF (3 mL) was added dropwise and it was stirred at room temperature until starting material disappeared. The reaction was then quenched by carefully adding saturated aqueous NH_4_Cl solution and extracted with ether three times. The combined organic layer was washed with brine, dried over Na_2_SO_4_, filtered, and then concentrated under reduced pressure. The concentrate was purified by column chromatography to give compound **9** as a yellow solid (85%). R_f_ = 0.50 (5% EtOAc/hexane); ^1^H-NMR (400 MHz, chloroform-*d*) δ (ppm): 1.02–1.04 (m, 12H), 1.37 (t, *J* = 7.2 Hz, 3H), 1.81–1.87 (m, 4H), 1.94–2.04 (m, 2H), 3.99 (t, *J* = 6.8 Hz, 2H), 4.16 (t, *J* = 6.4 Hz, 2H), 4.30 (q, *J* = 7.2 Hz, 2H), 6.47 (d, *J* = 16 Hz, 1H), 6.87 (s, 1H), 7.49–7.57 (m, 2H), 8.07–8.09 (m, 1H), 8.21–8.25 (m, 2H); ^13^C-NMR (100 MHz, chloroform-*d*) δ (ppm): 14.35, 22.66, 22.70, 24.95, 25.26, 38.00, 39.15, 60.43, 66.65, 75.16, 100.34, 118.06, 122.58, 122.80, 126.77, 126.98, 128.21, 129.00, 139.49, 149.58, 151.41, 167.19.

**3-(1,4-Bis(isoamyloxy)naphthalen-2-yl)propan-1-ol (10)**

To a solution of **9** (0.4 g, 1.0 mmol) in anhydrous THF (10 mL), Pd/C (10 wt.% in activated charcoal, 0.1 g, 0.1 mmol) was added. It was stirred at room temperature under atmospheric hydrogen gas until the starting material disappeared. After the reaction was completed, the solvent was evaporated after filtering through a celite. Water was added to the concentrate, followed by extraction with dichloromethane three times. The combined organic layer was washed with brine, dried over Na_2_SO_4_, filtered, and then concentrated under reduced pressure. Without further purification, crude compound (0.4 g, 1.0 mmol) in anhydrous THF (10 mL) was reacted with LiAlH_4_ (1.0 M in THF, 1.5 mL, 1.5 mmol) to give compound **10** as a light-yellow oil (98%). R_f_ = 0.38 (20% EtOAc/hexane); ^1^H-NMR (400 MHz, chloroform-*d*) δ (ppm): 1.00–1.03 (m, 12H), 1.79–2.00 (m, 8H), 2.90 (t, *J* = 7.0 Hz, 2H), 3.54 (t, *J* = 5.6 Hz, 2H), 3.97 (t, *J* = 6.8 Hz, 2H), 4.12 (t, *J* = 6.4 Hz, 2H), 6.59 (s, 1H), 7.41–7.45 (m, 1H), 7.49–7.53 (m, 1H), 7.97–7.99 (m, 1H), 8.22–8.24 (m, 1H); ^13^C-NMR (100 MHz, chloroform-*d*) δ (ppm): 22.68, 22.75, 25.11, 25.26, 25.91, 33.04, 38.10, 39.21, 60.90, 66.60, 73.65, 106.12, 121.63, 122.39, 124.69, 125.62, 126.46, 128.56, 128.95, 145.80, 151.56.

**1-(3-(1,4-Bis(isoamyloxy)naphthalen-2-yl)propyl)-1*H*-imidazole (11)**

To a solution of **10** (0.16 g, 0.45 mmol) in anhydrous dichloromethane (4 mL), triethylamine (0.19 mL, 1.34 mmol) was added. After cooling to 0 °C, methane sulfonyl chloride (0.07 mL, 0.9 mmol) was added dropwise, and the mixture was slowly warmed to room temperature and stirred until the starting material disappeared. The reaction was then quenched with saturated aqueous NaHCO_3_ solution and extracted with dichloromethane three times. The combined organic layer was washed with brine, dried over Na_2_SO_4_, filtered and then concentrated under reduced pressure. The concentrate was purified by column chromatography to give a mesylate intermediate. Mesylate (0.29 g, 0.66 mmol) was dissolved in acetonitrile (6 mL), followed by adding imidazole (0.18 g, 2.64 mmol). It was stirred under reflux until the starting material disappeared. After the reaction was completed, the solvent was evaporated, and the concentrate was purified by column chromatography to give compound **11** as a brown oil (63%). R_f_ = 0.45 (6% MeOH/DCM); ^1^H-NMR (400 MHz, chloroform-*d*) δ (ppm): 1.00–1.03 (m, 12H), 1.76–1.84 (m, 4H), 1.87–1.99 (m, 2H), 2.15–2.22 (m, 2H), 2.78 (t, *J* = 7.4 Hz, 2H), 3.89 (t, *J* = 7.0 Hz, 2H), 3.96 (t, *J* = 7.0 Hz, 2H), 4.11 (t, *J* = 6.4 Hz, 2H), 6.52 (s, 1H), 6.97 (s, 1H), 7.08 (s, 1H), 7.42–7.46 (m, 1H), 7.50–7.55 (m, 2H), 7.98–8.00 (m, 1H), 8.22–8.24 (m, 1H); ^13^C-NMR (100 MHz, chloroform-*d*) δ (ppm): 22.65, 22.74, 25.10, 25.23, 27.39, 31.90, 38.08, 39.23, 46.48, 66.66, 73.29, 105.78, 118.91, 121.69, 122.38, 124.88, 125.75, 126.52, 128.06, 128.87, 129.10, 137.10, 146.06, 151.39.

### 3.3. General Procedure for the Synthesis of IMS Derivatives

**Method A**: The bromide intermediate was dissolved in the solvent and reacted with various substituted imidazoles. It was refluxed until the starting material disappeared. Upon the completion of the reaction, it was concentrated, and the residue was purified by the recrystallization or column chromatography to give the corresponding IMS derivatives.

**Method B**: The imidazole-containing naphthalene intermediate was dissolved in the solvent and reacted with various alkyl halides. It was refluxed until the starting material disappeared. When the completion of the reaction, it was concentrated, and the residue was purified by the recrystallization or column chromatography to give the corresponding IMS derivatives.

**1-Benzyl-3-((1-methoxynaphthalen-2-yl)methyl)-1*H*-imidazol-3-ium bromide (IMS-01)**

**Compound 12a** (80 mg, 0.32 mmol) and 1-benzylimidazole (0.1 g, 0.64 mmol) in acetonitrile (3 mL) were reacted according to **method A,** and the crude was purified by column chromatography to give **IMS-01** as a yellow oil (34%). R_f_ = 0.15 (6% MeOH/DCM); ^1^H-NMR (400 MHz, DMSO-*d_6_*) δ (ppm): 3.92 (s, 3H), 5.42 (s, 2H), 5.60 (s, 2H), 7.37–7.42 (m, 5H), 7.47–7.50 (m, 1H), 7.57–7.64 (m, 2H), 7.78–7.83 (m, 3H), 7.97–7.99 (m, 1H), 8.07–8.09 (m, 1H), 9.41 (s, 1H); ^13^C-NMR (100 MHz, DMSO-*d_6_*) δ (ppm): 47.72, 51.97, 62.73, 122.10, 122.76, 122.80, 123.19, 124.77, 126.81, 126.91, 127.11, 127.16, 128.28, 128.76, 129.01, 134.91, 134.94, 136.47, 154.48; HRMS (FAB^+^) m/z calculated for C_22_H_21_N_2_O [M − Br]^+^ 329.1648, found 329.1646.

**1-Benzyl-3-((1-(isoamyloxy)naphthalen-2-yl)methyl)-1*H*-imidazol-3-ium bromide (IMS-02)**

**Compound 12b** (0.16 g, 0.52 mmol) and 1-benzylimidazole (0.1 g, 0.62 mmol) in acetonitrile (3 mL) were reacted according to **method A,** and the residue was recrystallized from ethyl acetate to give **IMS-02** as a white solid (94%). R_f_ = 0.26 (6% MeOH/DCM); m.p. 158.4–160.3 °C; ^1^H-NMR (400 MHz, DMSO-*d_6_*) δ (ppm): 0.95 (d, *J* = 6.4 Hz, 6H), 1.77–1.83 (m, 3H), 4.01 (t, *J* = 6.8 Hz, 2H), 5.43 (s, 2H), 5.61 (s, 2H), 7.37–7.44 (m, 6H), 7.59–7.63 (m, 2H), 7.77–7.79 (m, 2H), 7.84-7.85 (m, 1H), 7.97–8.00 (m, 1H), 8.05–8.07 (m, 1H), 9.34 (s, 1H); ^13^C-NMR (100 MHz, DMSO-*d_6_*) δ (ppm): 22.59, 24.59, 38.54, 47.85, 51.96, 73.95, 121.99, 122.69, 122.84, 123.19, 124.68, 126.73, 126.80, 127.13, 127.38, 128.24, 128.75, 128.99, 134.87, 136.35, 153.47; HRMS (FAB^+^) m/z calculated for C_26_H_29_N_2_O [M − Br]^+^ 385.2274, found 385.2275.

**1-Benzyl-3-((4-methoxynaphthalen-2-yl)methyl)-1*H*-imidazol-3-ium bromide (IMS-03)**

**Compound 12c** (0.1 g, 0.4 mmol) and 1-benzylimidazole (76 mg, 0.48 mmol) in acetonitrile (3 mL) were reacted according to **method A,** and the crude was purified by column chromatography to give **IMS-03** as a clear oil (87%). R_f_ = 0.17 (6% MeOH/DCM); ^1^H-NMR (400 MHz, DMSO-*d_6_*) δ (ppm): 3.96 (s, 3H), 5.47 (s, 2H), 5.58 (s, 2H), 7.08 (s, 1H), 7.37–7.46 (m, 5H), 7.52–7.57 (m, 3H), 7.85–7.88 (m, 2H), 7.94–7.95 (m, 1H), 8.11–8.13 (m, 1H), 9.60 (s, 1H); ^13^C-NMR (100 MHz, DMSO-*d_6_*) δ (ppm): 51.94, 52.53, 55.93, 104.43, 119.90, 121.46, 122.77, 123.00, 124.63, 126.15, 127.22, 127.72, 128.35, 128.73, 128.96, 132.67, 133.61, 134.95, 136.39, 155.41; HRMS (FAB^+^) m/z calculated for C_22_H_21_N_2_O [M − Br]^+^ 329.1648, found 329.1644.

**1-Benzyl-3-((4-(isoamyloxy)naphthalen-2-yl)methyl)-1*H*-imidazol-3-ium bromide (IMS-04)**

**Compound 12d** (0.1 g, 0.33 mmol) and 1-benzylimidazole (62 mg, 0.39 mmol) in acetonitrile (3 mL) were reacted according to **method A,** and the crude was purified by recrystallization from ethyl acetate to give **IMS-04** as a white solid (98%). R_f_ = 0.20 (6% MeOH/DCM); m.p. 174.7–175.8 °C; ^1^H-NMR (400 MHz, DMSO-*d_6_*) δ (ppm): 0.97 (d, *J* = 6.4 Hz, 6H), 1.73–1.78 (m, 2H), 1.86–1.92 (m, 1H), 4.15 (t, *J* = 6.4 Hz, 2H), 5.44 (s, 2H), 5.54 (s, 2H), 7.04 (s, 1H), 7.36–7.42 (m, 5H), 7.49 (s, 1H), 7.52–7.58 (m, 2H), 7.84–7.85 (m, 2H), 7.91 (s, 1H), 8.11–8.13 (m, 1H), 9.49 (s, 1H); ^13^C-NMR (100MHz, DMSO-*d_6_*) δ (ppm): 22.44, 24.82, 37.31, 52.02, 52.64, 66.43, 104.88, 119.65, 121.49, 122.81, 123.06, 124.79, 126.13, 127.21, 127.67, 128.23, 128.73, 128.96, 132.55, 133.62, 134.86, 136.35, 154.77; HRMS (FAB^+^) m/z calculated for C_26_H_29_N_2_O [M − Br]^+^ 385.2274, found 385.2275.

**1-Benzyl-3-((1,4-dimethoxynaphthalen-2-yl)methyl)-1*H*-imidazol-3-ium bromide (IMS-05)**

**Compound 12e** (0.1 g, 0.36 mmol) and 1-benzylimidazole (0.11 g, 0.72 mmol) in acetonitrile (3 mL) were reacted according to **method A,** and the crude was purified by column chromatography to give **IMS-05** as a brown oil (70%). R_f_ = 0.17 (6% MeOH/DCM); ^1^H-NMR (400 MHz, DMSO-*d_6_*) δ (ppm): 3.85 (s, 3H), 3.94 (s, 3H), 5.44 (s, 2H), 5.57 (s, 2H), 7.02 (s, 1H), 7.36–7.43 (m, 5H), 7.56–7.66 (m, 2H), 7.83–7.85 (m, 2H), 8.01–8.03 (m, 1H), 8.14–8.17 (m, 1H), 9.50 (s, 1H); ^13^C-NMR (100 MHz, DMSO-*d_6_*) δ (ppm): 48.01, 51.96, 55.97, 62.74, 105.13, 122.12, 122.16, 122.38, 122.71, 123.19, 126.22, 126.63, 127.35, 127.74, 128.27, 128.78, 129.02, 135.01, 136.40, 147.97, 151.62; HRMS (FAB^+^) m/z calculated for C_23_H_23_N_2_O_2_ [M − Br]^+^ 359.1754, found 359.1749.

**1-Benzyl-3-((1,4-diisopropoxynaphthalen-2-yl)methyl)-1*H*-imidazol-3-ium bromide (IMS-06)**

**Compound 12f** (0.14 g, 0.42 mmol) and 1-benzylimidazole (79 mg, 0.5 mmol) in acetonitrile (4 mL) were reacted according to **method A,** and the crude was purified by column chromatography to give **IMS-06** as a yellow oil (62%). R_f_ = 0.33 (6% MeOH/DCM); ^1^H-NMR (400 MHz, DMSO-*d_6_*) δ (ppm): 1.31–1.33 (m, 12H), 4.32–4.37 (m, 1H), 4.63–4.69 (m, 1H), 5.43 (s, 2H), 5.57 (s, 2H), 6.87 (s, 1H), 7.38–7.41 (m, 5H), 7.52–7.62 (m, 2H), 7.77 (t, *J* = 1.8 Hz, 1H), 7.82 (t, *J* = 1.8 Hz, 1H), 7.98–8.01 (m, 1H), 8.12–8.14 (m, 1H), 9.37 (s, 1H); ^13^C-NMR (100 MHz, DMSO-*d_6_*) δ (ppm): 21.78, 22.15, 48.40, 51.92, 70.18, 77.02, 106.69, 122.28, 122.75, 122.99, 123.16, 126.26, 126.89, 127.06, 128.00, 128.20, 128.75, 128.92, 128.98, 135.03, 136.33, 144.71, 149.38; HRMS (FAB^+^) m/z calculated for C_27_H_31_N_2_O_2_ [M − Br]^+^ 415.2380, found 415.2378.

**1-Benzyl-3-((1,4-bis(isoamyloxy)naphthalen-2-yl)methyl)-1*H*-imidazol-3-ium bromide (IMS-07)**

**Compound 12g** (0.12 g, 0.3 mmol) and 1-benzylimidazole (52 mg, 0.33 mmol) in acetonitrile (3 mL) were reacted according to **method A,** and the crude was recrystallized from ether to give **IMS-07** as a white solid (84%). R_f_ = 0.21 (6% MeOH/DCM); m.p. 124.7–126.1 °C; ^1^H-NMR (400 MHz, DMSO-*d_6_*) δ (ppm): 0.92–0.98 (m, 12H), 1.72–1.90 (m, 6H), 3.92 (t, *J* = 6.6 Hz, 2H), 4.10 (t, *J* = 6.4 Hz, 2H), 5.42 (s, 2H), 5.54 (s, 2H), 6.93 (s, 1H), 7.39 (s, 5H), 7.57–7.66 (m, 2H), 7.80 (s, 1H), 7.83 (s, 1H), 7.98–8.00 (m, 1H), 8.15–8.17 (m, 1H), 9.33 (s, 1H); ^13^C-NMR (100 MHz, DMSO-*d_6_*) δ (ppm): 22.44, 22.57, 24.58, 24.82, 37.34, 38.51, 48.12, 51.93, 66.50, 73.87, 105.55, 121.99, 122.09, 122.76, 123.16, 126.35, 126.53, 127.25, 128.01, 128.14, 128.72, 128.94, 134.93, 136.20, 146.80, 150.90; HRMS (FAB^+^) m/z calculated for C_31_H_39_N_2_O_2_ [M − Br]^+^ 471.3006, found 471.3007.

**1-Benzyl-3-((1,4-bis(octyloxy)naphthalen-2-yl)methyl)-1*H*-imidazol-3-ium bromide (IMS-08)**

**Compound 12h** (0.13 g, 0.27 mmol) and 1-benzylimidazole (85 mg, 0.54 mmol) in acetonitrile (3 mL) were reacted according to **method A,** and the crude was purified by column chromatography to give **IMS-08** as a brown oil (85%). R_f_ = 0.26 (6% MeOH/DCM); ^1^H-NMR (400 MHz, DMSO-*d_6_*) δ (ppm): 0.83–0.86 (m, 6H), 1.26–1.34 (m, 20H), 1.80–1.86 (m, 4H), 3.88 (t, *J* = 6.6 Hz, 2H), 4.06 (t, *J* = 6.2 Hz, 2H), 5.42 (s, 2H), 5.53 (s, 2H), 6.90 (s, 1H), 7.39 (s, 5H), 7.56–7.65 (m, 2H), 7.79 (t, *J* =1.6 Hz, 1H), 7.83 (t, *J* = 1.8 Hz, 1H), 7.98–8.00 (m, 1H), 8.15–8.17 (m, 1H), 9.33 (s, 1H); ^13^C-NMR (100 MHz, DMSO-*d_6_*) δ (ppm): 13.99, 22.11, 25.50, 25.76, 28.60, 28.70, 28.76, 28.94, 29.77, 31.28, 48.10, 51.94, 68.08, 75.30, 105.60, 122.07, 122.25, 122.77, 123.18, 126.36, 126.53, 127.25, 127.89, 128.03, 128.17, 128.73, 128.96, 134.99, 136.24, 146.71, 150.91; HRMS (FAB^+^) m/z calculated for C_37_H_51_N_2_O_2_ [M − Br]^+^ 555.3945, found 555.3947.

**1-Benzyl-3-(naphthalen-2-ylmethyl)-1*H*-imidazol-3-ium bromide (IMS-09)**

2-(Bromomethyl)naphthalene (0.22 g, 1.0 mmol) and 1-benzylimidazole (0.19 g, 1.2 mmol) in acetonitrile (3 mL) were reacted according to **method A,** and the residue was recrystallized from ethyl acetate to give **IMS-09** as a white solid (55%). R_f_ = 0.26 (6% MeOH/DCM); m.p. 169.5–171.3 °C; ^1^H-NMR (400 MHz, DMSO-*d_6_*) δ (ppm): 5.46 (s, 2H), 5.63 (s, 2H), 7.38–7.46 (m, 5H), 7.53–7.58 (m, 3H), 7.87–7.88 (m, 1H), 7.91–7.99 (m, 5H), 9.54 (s, 1H); ^13^C-NMR (100 MHz, DMSO-*d_6_*) δ (ppm): 52.01, 52.21, 122.87, 122.99, 125.74, 126.74, 126.77, 127.67, 127.87, 128.33, 128.74, 128.8, 128.98, 132.14, 132.68, 132.72, 134.79, 136.40; HRMS (FAB^+^) m/z calculated for C_21_H_19_N_2_ [M – Br]^+^ 299.1543, found 299.1547.

**1-Benzyl-3-(2-(1,4-bis(isoamyloxy)naphthalen-2-yl)ethyl)-1*H*-imidazol-3-ium bromide (IMS-10)**

**Compound 8** (0.12 g, 0.3 mmol) and 1-benzylimidazole (0.14 g, 0.9 mmol) in toluene (3 mL) were reacted according to **method A,** and the residue was recrystallized from ethyl acetate to give **IMS-10** as a white solid (75%). R_f_ = 0.27 (6% MeOH/DCM); m.p. 126.7–128.8 °C; ^1^H-NMR (400 MHz, DMSO-*d_6_*) δ (ppm): 0.92 (d, *J* = 6.4 Hz, 6H), 0.97 (d, *J* = 6.4 Hz, 6H), 1.64–1.90 (m, 6H), 3.25 (t, *J* = 6.4 Hz, 2H), 3.72 (t, *J* = 6.8 Hz, 2H), 4.07 (t, *J* = 6.4 Hz, 2H), 4.57 (t, *J* = 6.6 Hz, 2H), 5.32 (s, 2H), 6.82 (s, 1H), 7.13–7.15 (m, 2H), 7.25–7.33 (m, 3H), 7.49–7.52 (m, 1H), 7.55–7.58 (m, 1H), 7.73 (t, *J* = 1.6 Hz, 1H), 7.77 (t, *J* = 1.8 Hz, 1H), 7.85–7.87 (m, 1H), 8.10–8.12 (m, 1H), 9.08 (s, 1H); ^13^C-NMR (100 MHz, DMSO-*d_6_*) δ (ppm): 22.52, 22.60, 24.45, 24.87, 30.35, 37.51, 38.65, 49.43, 51.64, 66.34, 72.90, 106.22, 121.63, 121.98, 122.45, 123.15, 124.93, 125.27, 125.39, 126.72, 127.75, 128.07, 128.53, 128.82, 134.75, 136.07, 146.15, 150.60; HRMS (FAB^+^) m/z calculated for C_32_H_41_N_2_O_2_ [M − Br]^+^ 485.3162, found 485.3157.

**1-(3-(1,4-Bis(isoamyloxy)naphthalen-2-yl)propyl)-3-methyl-1*H*-imidazol-3-ium iodide (IMS-11)**

**Compound 11** (0.1 g, 0.24 mmol) and iodomethane (0.023 mL, 0.37 mmol) in tetrahydrofuran (2 mL) were reacted according to **method B**. The crude was purified by column chromatography to give **IMS-11** as a brown oil (52%). R_f_ = 0.27 (6% MeOH/DCM); ^1^H-NMR (400 MHz, DMSO-*d_6_*) δ (ppm): 0.95–0.99 (m, 12H), 1.66–1.77 (m, 4H), 1.80–1.94 (m, 2H), 2.16–2.23 (m, 2H), 2.73 (t, *J* = 7.8 Hz, 2H), 3.79–7.83 (m, 5H), 4.14 (t, *J* = 6.4 Hz, 2H), 4.20 (t, *J* = 7.2 Hz, 2H), 6.82 (s, 1H), 7.44–7.48 (m, 1H), 7.53–7.57 (m, 1H), 7.71 (t, *J* = 1.6 Hz, 1H), 7.81 (t, *J* = 1.6 Hz, 1H), 7.90–7.92 (m, 1H), 8.08–8.10 (m, 1H), 9.12 (s, 1H); ^13^C-NMR (100 MHz, DMSO-*d_6_*) δ (ppm): 22.52, 22.62, 24.57, 24.86, 26.10, 30.17, 35.74, 37.56, 38.72, 48.70, 66.31, 72.78, 106.45, 121.47, 121.86, 122.31, 123.55, 124.87, 125.00, 126.62, 128.21, 128.55, 136.70, 145.20, 150.48; HRMS (FAB^+^) m/z calculated for C_27_H_39_N_2_O_2_ [M – I]^+^ 423.3006, found 423.3004.

**3-((1,4-Bis(isoamyloxy)naphthalen-2-yl)methyl)-1-methyl-1*H*-imidazol-3-ium bromide (IMS-12)**

**Compound 12g** (0.1 g, 0.25 mmol) and 1-methylimidazole (30 mg, 0.37 mmol) in acetonitrile (3 mL) were reacted according to **method A,** and the residue was purified by column chromatography to give **IMS-12** as an opaque oil (61%). R_f_ = 0.15 (6% MeOH/DCM); ^1^H-NMR (400 MHz, DMSO-*d_6_*) δ (ppm): 0.95–0.98 (m, 12H), 1.72–1.91 (m, 6H), 3.84 (s, 3H), 3.94 (t, *J* = 6.6 Hz, 2H), 4.14 (t, *J* = 6.4 Hz, 2H), 5.51 (s, 2H), 7.00 (s, 1H), 7.56–7.65 (m, 2H), 7.72 (t, *J* = 1.6 Hz, 1H), 7.78 (t, *J* = 1.8 Hz, 1H), 7.98–8.00 (m, 1H), 8.14–8.17 (m, 1H), 9.13 (s, 1H); ^13^C-NMR (100 MHz, DMSO-*d_6_*) δ (ppm): 22.51, 22.63, 24.65, 24.86, 35.88, 37.41, 38.56, 47.84, 66.57, 74.02, 105.80, 122.04, 122.12, 122.39, 122.53, 123.86, 126.37, 126.54, 127.26, 128.01, 136.54, 146.78, 150.91; HRMS (FAB^+^) m/z calculated for C_25_H_35_N_2_O_2_ [M – Br]^+^ 395.2693, found 395.2693.

**3-((1,4-Bis(isoamyloxy)naphthalen-2-yl)methyl)-1-isoamyl-1*H*-imidazol-3-ium bromide (IMS-13)**

**Compound 12g** (0.2 g, 0.5 mmol) and **13b** (0.1 g, 0.75 mmol) in acetonitrile (5 mL) were reacted according to **method A,** and the crude was purified by column chromatography to give **IMS-13** as a brown oil (76%). R_f_ = 0.32 (6% MeOH/DCM); ^1^H-NMR (400 MHz, DMSO-*d_6_*) δ (ppm): 0.87 (d, *J* = 6.8 Hz, 6H), 0.94–0.97 (m, 12H), 1.44–1.48 (m, 1H), 1.64–1.90 (m, 8H), 3.93 (t, *J* = 6.6 Hz, 2H), 4.12 (t, *J* = 6.4 Hz, 2H), 4.19 (t, *J* = 7.6 Hz, 2H), 5.52 (s, 2H), 6.94 (s, 1H), 7.56–7.66 (m, 2H), 7.80 (t, *J* = 1.8 Hz, 1H), 7.84 (t, *J* = 1.8 Hz, 1H), 7.98–8.00 (m, 1H), 8.15–8.17 (m, 1H), 9.25 (s, 1H); ^13^C-NMR (100 MHz, DMSO-*d_6_*) δ (ppm): 22.02, 22.48, 22.63, 24.64, 24.85, 37.38, 38.16, 38.57, 47.34, 47.97, 66.52, 73.91, 105.61, 122.02, 122.12, 122.27, 122.66, 122.81, 126.35, 126.55, 127.28, 128.04, 136.08, 146.78, 150.91; HRMS (FAB^+^) m/z calculated for C_29_H_43_N_2_O_2_ [M – Br]^+^ 415.3319, found 415.3314.

**3-((1,4-Bis(isoamyloxy)naphthalen-2-yl)methyl)-1-phenyl-1*H*-imidazol-3-ium bromide (IMS-14)**

**Compound 12g** (0.15 g, 0.38 mmol) and 1-phenyimidazole (63 mg, 0.44 mmol) in acetonitrile (3.8 mL) were reacted according to **method A,** and the crude was recrystallized from ethyl acetate to give **IMS-14** as a white solid (72%). R_f_ = 0.18 (6% MeOH/DCM); m.p. 140.3–142.1 °C; ^1^H-NMR (400 MHz, DMSO-*d_6_*) δ (ppm): 0.94–0.97 (m, 12H), 1.71–1.76 (m, 2H), 1.78–1.92 (m, 4H), 3.98 (t, *J* = 6.6 Hz, 2H), 4.16 (t, *J* = 6.4 Hz, 2H), 5.62 (s, 2H), 7.03 (s, 1H), 7.56–7.67 (m, 5H), 7.78–7.81 (m, 2H), 7.97 (t, *J* = 1.8 Hz, 1H), 8.00–8.02 (m, 1H), 8.16–8.18 (m, 1H), 8.35 (t, *J* = 1.8 Hz, 1H), 9.96 (s, 1H); ^13^C-NMR (100 MHz, DMSO-*d_6_*) δ (ppm): 22.49, 22.63, 24.65, 24.84, 37.38, 38.56, 48.35, 66.55, 74.00, 105.74, 121.60, 121.96, 122.05, 122.14, 123.43, 126.43, 126.56, 127.26, 128.01, 129.85, 130.15, 134.71, 135.57, 146.87, 150.92; HRMS (FAB^+^) m/z calculated for C_30_H_37_N_2_O_2_ [M − Br]^+^ 457.2849, found 457.2849.

**3-((1,4-Bis(isoamyloxy)naphthalen-2-yl)methyl)-1-(4-nitrobenzyl)-1*H*-imidazol-3-ium bromide (IMS-15)**

**Compound 12g** (0.2 g, 0.51 mmol) and **13d** (0.12 g, 0.61 mmol) in acetonitrile (1.02 mL) were reacted according to **method A,** and the crude was purified by column chromatography to give **IMS-15** as a yellow oil (82%). R_f_ = 0.23 (6% MeOH/DCM); ^1^H-NMR (400 MHz, DMSO-*d_6_*) δ (ppm): 0.91–0.96 (m, 12H), 1.70–1.91 (m, 6H), 3.92 (t, *J* = 6.6 Hz, 2H), 4.11 (t, *J* = 6.4 Hz, 2H), 5.57 (s, 2H), 5.64 (s, 2H), 6.99 (s, 1H), 7.56–7.65 (m, 2H), 7.68–7.70 (m, 2H), 7.87–7.88 (m, 1H), 7.91–7.92 (m, 1H), 7.98–8.00 (m, 1H), 8.15–8.17 (m, 1H), 8.24–8.26 (m, 2H), 9.45 (s, 1H); ^13^C-NMR (100 MHz, DMSO-*d_6_*) δ (ppm): 22.47, 22.60, 24.62, 24.86, 37.39, 38.54, 48.17, 51.02, 66.57, 73.96, 105.67, 122.04, 122.14, 122.18, 122.94, 123.35, 123.98, 126.38, 126.57, 127.29, 128.03, 129.53, 136.71, 142.29, 146.83, 147.55, 150.94; HRMS (FAB^+^) m/z calculated for C_31_H_38_N_3_O_4_ [M − Br]^+^ 516.2857, found 516.2858.

**3-((1,4-Bis(isoamyloxy)naphthalen-2-yl)methyl)-1-(4-methoxybenzyl)-1*H*-imidazol-3-ium bromide (IMS-16)**

**Compound 12g** (0.2 g, 0.51 mmol) and **13e** (0.11 g, 0.61 mmol) in acetonitrile (2.04 mL) were reacted according to **method A,** and the crude was purified by column chromatography to give **IMS-16** as a brown solid (79%). R_f_ = 0.23 (6% MeOH/DCM); m.p. 105.4–106.8 °C; ^1^H-NMR (400 MHz, DMSO-*d_6_*) δ (ppm): 0.92–0.97 (m, 12H), 1.71–1.90 (m, 6H), 3.73 (s, 3H), 3.90 (t, *J* = 6.6 Hz, 2H), 4.09 (t, *J* = 6.4 Hz, 2H), 5.32 (s, 2H), 5.51 (s, 2H), 6.91 (s, 1H), 6.93–6.96 (m, 2H), 7.37–7.39 (m, 2H), 7.56–7.66 (m, 2H), 7.76–7.77 (m, 1H), 7.79–7.80 (m, 1H), 7.98–7.99 (m, 1H), 8.14–8.17 (m, 1H), 9.28 (s, 1H); ^13^C-NMR (100 MHz, DMSO-*d_6_*) δ (ppm): 22.51, 22.63, 24.65, 24.88, 37.40, 38.56, 48.11, 51.56, 55.24, 66.55, 73.93, 105.61, 114.34, 122.05, 122.16, 122.30, 122.59, 123.10, 126.38, 126.60, 126.83, 127.33, 128.05, 130.09, 135.94, 146.81, 150.95, 159.60; HRMS (FAB^+^) m/z calculated for C_32_H_41_N_2_O_3_ [M − Br]^+^ 501.3112, found 501.3112.

**3-((1,4-Bis(isoamyloxy)naphthalen-2-yl)methyl)-1-isoamyl-2-methyl-1*H*-imidazol-3-ium bromide (IMS-17)**

**Compound 12g** (0.1 g, 0.25 mmol) and **14a** (58 mg, 0.38 mmol) in acetonitrile (3 mL) were reacted according to **method A,** and the crude was recrystallized from ether and hexane to give **IMS-17** as a brown solid (86%). R_f_ = 0.12 (6% MeOH/DCM); m.p. 115.3–117.4 °C; ^1^H-NMR (400 MHz, DMSO-*d_6_*) δ (ppm): 0.90 (d, *J* = 6.4 Hz, 6H), 0.95–0.98 (m, 12H), 1.53–1.63 (m, 3H), 1.69–1.74 (m, 2H), 1.78–1.91 (m, 4H), 2.68 (s, 3H), 3.96 (t, *J* = 6.8 Hz, 2H), 4.08–4.14 (m, 4H), 5.49 (s, 2H), 6.79 (s, 1H), 7.55–7.66 (m, 2H), 7.73–7.74 (m, 2H), 7.98–8.00 (m, 1H), 8.13–8.15 (m, 1H); ^13^C-NMR (100 MHz, DMSO-*d_6_*) δ (ppm): 9.52, 22.10, 22.47, 22.63, 24.65, 24.81, 25.03, 37.32, 37.72, 38.57, 46.14, 46.52, 66.47, 74.01, 104.90, 121.39, 121.68, 121.96, 122.09, 122.20, 126.19, 126.49, 127.33, 127.98, 144.09, 146.29, 150.93; HRMS (FAB^+^) m/z calculated for C_30_H_45_N_2_O_2_ [M − Br]^+^ 465.3475, found 465.3472.

**1-Benzyl-3-((1,4-bis(isoamyloxy)naphthalen-2-yl)methyl)-2-methyl-1*H*-imidazol-3-ium bromide (IMS-18)**

**Compound 12g** (0.2 g, 0.5 mmol) and **14b** (0.18 g, 1.02 mmol) in acetonitrile (5 mL) were reacted according to **method A,** and the crude was recrystallized from ether to give **IMS-18** as a white solid (93%). R_f_ = 0.13 (6% MeOH/DCM); m.p. 218.1–219.6 °C; ^1^H-NMR (400 MHz, DMSO-*d_6_*) δ (ppm): 0.94–0.96 (m, 12H), 1.69–1.89 (m, 6H), 2.69 (s, 3H), 3.94 (t, *J* = 6.6 Hz, 2H), 4.07 (t, *J* = 6.4 Hz, 2H), 5.43 (s, 2H), 5.51 (s, 2H), 6.77 (s, 1H), 7.32–7.42 (m, 5H), 7.56–7.65 (m, 2H), 7.74 (d, *J* = 2.0 Hz, 1H), 7.79 (d, *J* = 2.0 Hz, 1H), 7.98–8.00 (m, 1H), 8.13–8.15 (m, 1H); ^13^C-NMR (100 MHz, DMSO-*d_6_*) δ (ppm): 9.88, 22.50, 22.65, 24.65, 24.83, 37.34, 38.57, 46.75, 50.73, 66.48, 74.02, 104.87, 121.89, 121.99, 122.11, 126.22, 126.55, 127.38, 127.81, 128.01, 128.58, 129.05, 134.53, 144.56, 146.37, 150.98; HRMS (FAB^+^) m/z calculated for C_32_H_41_N_2_O_2_ [M − Br]^+^ 485.3162, found 485.3163.

**3-((1,4-Bis(isoamyloxy)naphthalen-2-yl)methyl)-2-(hydroxymethyl)-1-isoamyl-1*H*-imidazol-3-ium bromide (IMS-19)**

**Compound 12g** (0.1 g, 0.25 mmol) and **14e** (64 mg, 0.38 mmol) in acetonitrile (3 mL) were reacted according to **method A** and the crude was recrystallized from ether and hexane to give **IMS-19** as a light brown solid (83%). R_f_ = 0.36 (6% MeOH/DCM); m.p. 127.6–129.2 °C; ^1^H-NMR (400 MHz, DMSO-*d_6_*) δ (ppm): 0.90–0.97 (m, 18H), 1.55–1.62 (m, 1H), 1.66–1.73 (m, 4H), 1.78–1.90 (m, 4H), 3.98 (t, *J* = 6.8 Hz, 2H), 4.08 (t, *J* = 6.4 Hz, 2H), 4.25 (t, *J* = 7.8 Hz, 2H), 4.93 (d, *J* = 3.69 Hz, 2H), 5.61 (s, 2H), 6.19 (s, 1H), 6.82 (s, 1H), 7.55–7.66 (m, 2H), 7.73 (d, *J* = 2.0 Hz, 1H), 7.84 (d, *J* = 2.0 Hz, 1H), 8.00–8.02 (m, 1H), 8.13–8.15 (m, 1H); ^13^C-NMR (100 MHz, DMSO-*d_6_*) δ (ppm): 22.13, 22.45, 22.63, 24.62, 24.81, 25.15, 37.29, 38.44, 38.57, 46.52, 50.46, 66.46, 74.04, 104.88, 121.99, 122.07, 122.14, 122.33, 126.19, 126.47, 127.28, 127.98, 144.92, 146.30, 150.88; HRMS (FAB^+^) m/z calculated for C_30_H_45_N_2_O_3_ [M − Br]^+^ 481.3425, found 481.3420.

**1-Benzyl-3-((1,4-bis(isoamyloxy)naphthalen-2-yl)methyl)-2-(hydroxymethyl)-1*H*-imidazol-3-ium bromide (IMS-20)**

**Compound 12g** (0.1 g, 0.25 mmol) and **14f** (72 mg, 0.38 mmol) in acetonitrile (2.5 mL) were reacted according to **method A,** and the crude was recrystallized from ethyl acetate to give **IMS-20** as a white solid (37%). R_f_ = 0.28 (6% MeOH/DCM); m.p. 128.4–130.3 °C; ^1^H-NMR (400 MHz, DMSO-*d_6_*) δ (ppm): 0.94–0.96 (m, 12H), 1.68–1.73 (m, 2H), 1.76–1.89 (m, 4H), 3.97 (t, *J* = 6.8 Hz, 2H), 4.05 (t, *J* = 6.2 Hz, 2H), 4.99 (d, *J* = 4.8 Hz, 2H), 5.53 (s, 2H), 5.63 (s, 2H), 6.28 (t, *J* = 5.2 Hz, 1H), 6.79 (s, 1H), 7.36–7.41 (m, 5H), 7.55–7.66 (m, 2H), 7.71 (d, *J* = 2.0 Hz, 1H), 7.77 (d, *J* = 2.0 Hz, 1H), 7.99–8.01 (m, 1H), 8.13–8.15 (m, 1H); ^13^C-NMR (100 MHz, DMSO-*d_6_*) δ (ppm): 22.46, 22.63, 24.61, 24.80, 37.29, 38.56, 46.71, 50.66, 50.88, 66.42, 74.02, 104.83, 122.00, 122.08, 122.20, 122.52, 126.21, 126.50, 127.31, 128.06, 128.60, 128.91, 134.66, 145.27, 146.36, 150.90; HRMS (FAB^+^) m/z calculated for C_32_H_41_N_2_O_3_ [M − Br]^+^ 501.3112, found 501.3110.

**3-((1,4-Bis(isoamyloxy)naphthalen-2-yl)methyl)-1-methyl-1*H*-benzo[*d*]imidazol-3-ium bromide (IMS-21)**

**Compound 12g** (0.1 g, 0.25 mmol) and **15a** (34 mg, 0.25 mmol) in acetonitrile (2.5 mL) were reacted according to **method A,** and the crude was recrystallized from ethyl acetate to give **IMS-21** as a white solid (82%). R_f_ = 0.48 (10% MeOH/DCM); m.p. 175.8–177.6 °C; ^1^H-NMR (400 MHz, DMSO-*d_6_*) δ (ppm): 0.91–0.95 (m, 12H), 1.68–1.73 (m, 2H), 1.76–1.88 (m, 4H), 4.00 (t, *J* = 6.6 Hz, 2H), 4.08 (s, 3H), 4.12 (t, *J* = 6.4 Hz, 2H), 5.82 (s, 2H), 7.05 (s, 1H), 7.56–7.70 (m, 4H), 8.02–8.06 (m, 3H), 8.14–8.16 (m, 1H), 9.73 (s, 1H); ^13^C-NMR (100 MHz, DMSO-*d_6_*) δ (ppm): 22.46, 22.58, 24.62, 24.80, 33.36, 37.33, 38.52, 45.67, 66.54, 74.26, 105.72, 113.45, 113.76, 121.40, 122.09, 126.44, 126.61, 126.67, 127.30, 128.02, 131.01, 131.93, 142.65, 146.82, 150.95; HRMS (FAB^+^) m/z calculated for C_29_H_37_N_2_O_2_ [M – Br]^+^ 445.2849, found 445.2844.

**3-((1,4-Bis(isoamyloxy)naphthalen-2-yl)methyl)-1-isoamyl-1*H*-benzo[*d*]14midazole-3-ium bromide (IMS-22)**

**Compound 12g** (0.1 g, 0.25 mmol) and **15b** (49 mg, 0.25 mmol) in acetonitrile (2.5 mL) were reacted according to **method A,** and the crude was recrystallized from ethyl acetate to give **IMS-22** as a white solid (65%). R_f_ = 0.24 (6% MeOH/DCM); m.p. 229.9–231.7 °C; ^1^H-NMR (400 MHz, DMSO-*d_6_*) δ (ppm): 0.90–0.94 (m, 18H), 1.56–1.60 (m, 1H), 1.68–0.87 (m, 8H), 4.00 (t, *J* = 6.6 Hz, 2H), 4.10 (t, *J* = 6.4 Hz, 2H), 4.52 (t, *J* = 7.6 Hz, 2H), 5.82 (s, 2H), 7.04 (s, 1H), 7.56–7.69 (m, 4H), 8.00–8.05 (m, 2H), 8.09–8.12 (m, 1H), 8.14–8.16 (m, 1H), 9.87 (s, 1H); ^13^C-NMR (100 MHz, DMSO-*d_6_*) δ (ppm): 22.09, 22.43, 22.56, 24.61, 24.80, 24.99, 37.22, 37.31, 38.54, 45.21, 45.85, 66.54, 74.19, 105.52, 113.66, 113.89, 121.36, 122.07, 122.12, 126.42, 126.62, 126.74, 127.34, 128.04, 131.10, 131.21, 142.12, 146.81, 150.97; HRMS (FAB^+^) m/z calculated for C_33_H_45_N_2_O_2_ [M − Br]^+^ 501.3475, found 501.3470.

**1-Benzyl-3-((1,4-bis(isoamyloxy)naphthalen-2-yl)methyl)-1*H*-benzo[*d*]imidazol-3-ium bromide (IMS-23)**

**Compound 12g** (0.1 g, 0.25 mmol) and **15c** (53 mg, 0.25 mmol) in acetonitrile (2.5 mL) were reacted according to **method A,** and the crude was recrystallized from hexane to give **IMS-23** as a white solid (73%). R_f_ = 0.31 (10% MeOH/DCM); m.p. 159.5–160.7 °C; ^1^H-NMR (400 MHz, DMSO-*d_6_*) δ (ppm): 0.88 (d, *J* = 6.0 Hz, 6H), 0.94 (d, *J* = 6.4 Hz, 6H), 1.69–1.89 (m, 6H), 4.00 (t, *J* = 6.6 Hz, 2H), 4.10 (t, *J* = 6.4 Hz, 2H), 5.78 (s, 2H), 5.85 (s, 2H), 7.05 (s, 1H), 7.35–7.41 (m, 3H), 7.47–7.49 (m, 2H), 7.57–7.68 (m, 4H), 7.96–7.98 (m, 1H), 8.01–8.06 (m, 2H), 8.15–8.17 (m, 1H), 9.95 (s, 1H); ^13^C-NMR (100 MHz, DMSO-*d_6_*) δ (ppm): 22.44, 22.54, 24.57, 24.81, 37.32, 38.50, 46.05, 49.84, 66.53, 74.19, 105.55, 113.81, 114.03, 121.30, 122.07, 122.13, 126.44, 126.65, 126.85, 127.35, 128.07, 128.71, 128.92, 130.91, 131.42, 134.12, 142.42, 146.90, 150.99; HRMS (FAB^+^) m/z calculated for C_35_H_41_N_2_O_2_ [M − Br]^+^ 521.3162, found 521.3160.

### 3.4. Cell Culture and Viability Assay

#### 3.4.1. Cell Culture

The human colon adenocarcinoma colorectal (HT-29) and human liver carcinoma hepatocellular (HepG2) [Korean cell line bank (KCLB), Korea] cell lines were cultured in Dulbecco’s modified Eagle’s medium (DMEM) containing 10% fetal bovine serum (FBS) and 1% penicillin/streptomycin. The CCD-18Co human colon normal cell line [Korean cell line bank (KCLB), Korea] was cultured in minimum essential medium (MEM) containing 10% fetal bovine serum (FBS) and 1% penicillin/streptomycin. The cells were incubated under a humidified atmosphere of 95% and 5% CO_2_ at 37 °C. The cells were serially sub-cultured three times per week.

#### 3.4.2. Cell Viability Assay

The HT-29 and HepG2 cells were seeded in 96-well plates at a density of 1.5 × 10^5^ cells/well, and CCD-18Co cells with a passage between 7 and 12 were seeded in 96-well plates at a density of 1 × 10^4^ cells/well, followed by incubation for 24 h. Five concentrations of **IMS-01** to **IMS-23** (0.5–10 μM) in serum-free medium were added, and the cells were further incubated for 24 h. After incubation, cells were treated with WST (water-soluble tetrazolium salt, Catalog no. EZ-3000, DoGenBio, Korea) in each well and incubated for 30 min at room temperature while avoiding light. The absorbance was determined by the optical density at 450 nm, measured using a LUX Multimode microplate reader (Thermo scientific, Waltham, MA, USA).

#### 3.4.3. Statistical Analysis

All experiments were performed at least three times, and each dataset is presented as the average of three replicates (mean ± standard deviation).

## 4. Conclusions

In this study, novel 1,4-dialkoxynaphthanelen-2-alkyl imidazolium salts (IMS) were synthesized in a manner that addressed the synthesis problems of previous studies. To evaluate the potential of the IMSs as anticancer agent, various derivatives were synthesized with modification of naphthalene rings with various alkoxy groups, the alkyl connector between the naphthalene and imidazole rings, and the imidazole ring with various substituents, and the toxicity of these compounds were evaluated using HepG2, HT-29, and CCD-18Co cells.

Cytotoxic activity was not observed when an alkoxy group was absent, short, or too long. In addition, cytotoxicity was only shown when the alkoxy group was an isoamyloxy group located at both positions 1 and 4 of the naphthalene ring. There was no significant difference in the activity of the compounds with the number of carbons in the alkyl connectors; however, considering its ease of synthesis, **IMS-07**, with a one-carbon alkyl group, was proposed to be the best. In the case of derivatives with substituents attached at various positions on the imidazole ring, all showed good activity without significant differences.

We expected differences in cytotoxic outcomes between cancer cells (HepG2 and HT-29) and normal cells (CCD-18Co). Unfortunately, as shown in our results, we could not identify a difference in the activity of IMS compounds according to cell characteristics. However, the difference in cytotoxicity as a function of the structure of the IMS compound was clearly confirmed. In a further study, we will investigate the exact mode of action (MOA) of how our compound is active in cancer cells and the differences in activity between each cell type. Furthermore, the synthesis of new IMS compounds showing better activity must be carried out on the basis of the identified MOA.

## Figures and Tables

**Figure 3:**
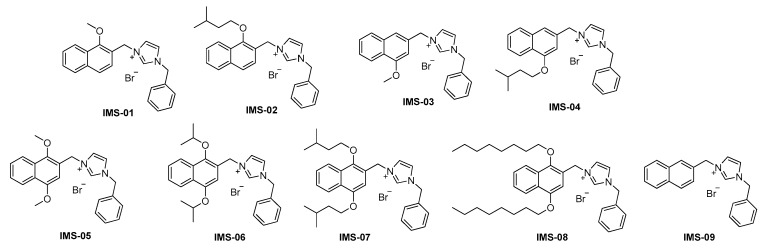
Structures of newly synthesized **IMS-01 to IMS-09**.

**Table 1:** Lethal dose 50 (LD_50_) values (μM) of **IMS-01** to **IMS-23** against HepG2, HT-29, and CCD-18Co cells.

	Compound	HepG2	HT-29	CCD-18Co
1	IMS-01	N.D.	N.D.	N.D.
2	IMS-02	N.D.	N.D.	N.D.
3	IMS-03	N.D.	N.D.	N.D.
4	IMS-04	N.D.	N.D.	N.D.
5	IMS-05	N.D.	N.D.	N.D.
6	IMS-06	N.D.	N.D.	N.D.
7	IMS-07	7.44 ± 0.95	9.64 ± 0.08	4.13 ± 0.05
8	IMS-08	N.D.	N.D.	N.D.
9	IMS-09	N.D.	N.D.	N.D.
10	IMS-10	6.20 ± 0.40	7.36 ± 0.03	3.58 ± 0.00
11	IMS-11	8.33 ± 0.07	8.20 ± 0.05	5.60 ± 0.11
12	IMS-12	9.38 ± 0.09	6.50 ± 0.12	4.39 ± 0.03
13	IMS-13	9.6 ± 0.16	8.33 ± 0.07	5.42 ± 0.12
14	IMS-14	8.06 ± 0.18	6.09 ± 0.69	3.56 ± 0.02
15	IMS-15	7.12 ± 0.07	9.75 ± 0.08	9.63 ± 0.09
16	IMS-16	7.95 ± 0.14	8.02 ± 0.42	6.69 ± 0.05
17	IMS-17	7.17 ± 0.16	6.64 ± 0.03	3.64 ± 0.05
18	IMS-18	7.64 ± 0.02	8.10 ± 0.09	3.00 ± 0.06
19	IMS-19	7.91 ± 0.14	7.81 ± 0.04	3.63 ± 0.03
20	IMS-20	7.56 ± 0.01	7.78 ± 0.01	3.49 ± 0.07
21	IMS-21	8.4 ± 0.11	8.75 ± 0.34	3.57 ± 0.08
22	IMS-22	8.11 ± 0.02	6.86 ± 0.05	3.45 ± 0.09
23	IMS-23	9.51 ± 0.42	7.41 ± 0.01	3.34 ± 0.10

N.D.: not determined.

## Data Availability

The data presented in this study are available on request from the corresponding author.

## Supplementary Materials

The supporting information can be downloaded at https://www.mdpi.com/article/10.3390/ijms24032713/s1. References [24,25,26,27] are cited in supplementary materials.
