# Supplementary material for: Synthesis of 1,4-Dialkoxynaphthalene-Based Imidazolium Salts and Their Cytotoxicity in Cancer Cell Lines
Source: Int J Mol Sci. 2023 Feb 1;24(3):2713. doi: 10.3390/ijms24032713 (PMC9917024; doi:10.3390/ijms24032713)
Supplement: Supplementary file 1 [file ijms-24-02713-s001.zip › ijms-2162474-supplementary.pdf]

Synthesis of 1,4-Dialkoxynaphthalene-Based Imidazolium Salts and Their Cytotoxicity in Cancer Cell Lines

Haena Lee [1,†], Yejin Jeon [2,†], Hyejin Moon [1], Eunjoo H. Lee [2],Tae Hoon Lee [1,*] and Hakwon Kim [1,*]

1 Department of Applied Chemistry, Global Center for Pharmaceutical Ingredient Materials, Kyung Hee University, Yongin 1732, Republic of Korea

2 Department of Gerontology, Graduate School of East-West Medicinal Science, Kyung Hee University, Yongin 17104, Republic of Korea

* Correspondence: thlee@khu.ac.kr (T.H.L.); hwkim@khu.ac.kr (H.K.); Tel.: +82-312012459 (H.K.)

† These authors contributed equally to this work.

Supporting information

Ⅰ. Synthetic procedures and characterization Data

1. Synthesis of bromide intermediate 12a-12h

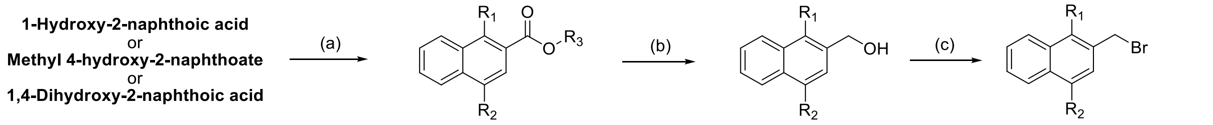

S1a R_1 = methoxy, R_2 = H, R_3 = methyl
S1b R_1 = i-amyloxy, R_2 = H, R_3 = i-amyl
S1c R_1 = H, R_2 = methoxy, R_3 = methyl
S1d R_1 = H, R_2 = i-amyloxy, R_3 = i-amyl
S1e R_1, R_2 = methoxy, R_3 = methyl
S1f R_1, R_2 = i-propyloxy, R_3 = i-Pr
S1g R_1, R_2 = i-amyloxy, R_3 = i-amyl
S1h R_1, R_2 = octyloxy, R_3 = octyl

5a R_1 = methoxy, R_2 = H
5b R_1 = i-amyloxy, R_2 = H
5c R_1 = H, R_2 = methoxy
5d R_1 = H, R_2 = i-amyloxy
5e R_1, R_2 = methoxy
5f R_1, R_2 = i-propyloxy
5g R_1, R_2 = i-amyloxy
5h R_1, R_2 = octyloxy

12a R_1 = methoxy, R_2 = H
12b R_1 = i-amyloxy, R_2 = H
12c R_1 = H, R_2 = methoxy
12d R_1 = H, R_2 = i-amyloxy
12e R_1, R_2 = methoxy
12f R_1, R_2 = i-propyloxy
12g R_1, R_2 = i-amyloxy
12h R_1, R_2 = octyloxy

Reagents and conditions. (a) R-X or $(MeO)_2SO_2$, K_2CO_3 or Cs_2CO_3, acetone or DMF, reflux; **(b)** $LiAlH_4$, anhydrous THF, rt; **(c)** PBr_3, anhydrous DCM, 0 °C.

Scheme S1. Synthesis of bromide intermediate **12a-12h**

1.1. General synthetic procedure for 1- or 4-alkoxy-2-naphthoate or 1,4-dialkoxy-2-naphthoate (**S1a-S1h**)

The starting material was dissolved in acetone or DMF and K_2CO_3 or Cs_2CO_3 was added in two portions. After 30 minutes, dimethyl sulfate or the corresponding alkyl halide was added dropwise and refluxed at 55 - 80 °C until the starting material disappeared. After the reaction was completed, the solution was filtered and concentrated in vacuo, and water was added, followed by extraction with ethyl acetate three times. The combined organic layer was washed with brine, dried over anhydrous Na_2SO_4, filtered, and then concentrated under reduced pressure. The crude was purified by column chromatography to give a naphthoate derivatives (**S1**) [24, 25].

Methyl 1-methoxy-2-naphthoate (S1a)

1-Hydroxy-2-naphthoic acid (1.0 g, 5.31 mmol) in acetone (15 ml) was reacted with dimethyl sulfate (1.5 ml, 15.94 mmol) in the presence of K_2CO_3 (4.4 g, 32.0 mmol) as described above to give compound **S1a** as a yellow oil (81%). R_f = 0.41 (20% EtOAc/hexane); ^1H NMR (400MHz, Chloroform-*d*) δ (ppm) : 3.99 (s, 3H), 4.07 (s, 3H), 7.56 - 7.63 (m, 3H), 7.84 -7.87 (m, 2H), 8.27 - 8.30 (m, 1H); ^{13}C NMR (100MHz, Chloroform-*d*) δ (ppm) : 52.26, 63.39, 119.14, 123.60, 126.50, 126.63, 127.85, 128.31, 128.52, 136.74, 158.26, 166.67.

Isoamyl 1-(isoamyloxy)-2-naphthoate (S1b)

1-Hydroxy-2-naphthoic acid (0.5 g, 2.66 mmol) in DMF (10 ml) was reacted with 1-bromo-3-methyl butane (1.0 ml, 8.0 mmol) in the presence of K_2CO_3 (2.2 g, 16.0 mmol) as described above to give compound **S1b** as a colorless oil (98%). R_f = 0.35 (20% EtOAc/hexane); ^{1}H NMR (400MHz, Chloroform-*d*) δ (ppm) : 0.98 - 1.01 (m, 12H), 1.68 – 1.73 (m, 2H), 1.77 - 1.96 (m, 4H), 4.14 (t, *J* = 6.8 Hz, 2H), 4.41 (t, *J* = 7.0 Hz, 2H), 7.52 - 7.60 (m, 3H), 7.82 - 7.85 (m, 2H), 8.26 - 8.28 (m, 1H); ^{13}C NMR (100MHz, Chloroform-*d*) δ (ppm) : 22.52, 22.75, 25.07, 25.14, 37.46, 39.13, 63.80, 75.00, 119.63, 123.26, 123.66, 126.33, 126.71, 127.77, 128.13, 128.84, 136.59, 157.27, 166.55.

Methyl 4-methoxy-2-naphthoate (S1c)

Methyl 4-hydroxy-2-naphthoate (0.8 g, 4.0 mmol) in acetone (11 ml) was reacted with dimethyl sulfate (1.14 ml, 12.0 mmol) in the presence of K_2CO_3 (1.64 g, 12.0 mmol) as described above to give compound **S1c** as a colorless oil (99%). R_f = 0.19 (5% EtOAc/hexane); ^{1}H NMR (400MHz, Chloroform-*d*) δ (ppm) : 3.98 (s, 3H), 4.07 (s, 3H), 7.39 (s, 1H), 7.53 - 7.61 (m, 2H), 7.89 - 7.92 (m, 1H), 8.22 (s, 1H), 8.26 - 8.29 (m, 1H); ^{13}C NMR (100MHz, Chloroform-*d*) δ (ppm) : 52.27, 55.74, 102.81, 122.09, 123.64, 127.12, 127.51, 127.59, 127.80, 128.99, 133.38, 155.51, 167.44.

Methyl 4-(isoamyloxy)-2-naphthoate (S1d)

Methyl 4-hydroxy-2-naphthoate (0.8 g, 4.0 mmol) in DMF (12 ml) was reacted with 1 -bromo-3-methyl butane (0.75 ml, 12.0 mmol) in the presence of K_2CO_3 (1.64 g, 12.0 mmol) as described above to give compound **S1d** as a yellow oil (96%). R_f = 0.38 (5% EtOAc/hexane); ^{1}H NMR (400MHz, Chloroform-*d*) δ (ppm) : 1.03 (d, *J* = 6.4 Hz, 6H), 1.83 - 1.88 (m, 2H), 1.95 - 2.01 (m, 1H), 3.98 (s, 3H), 4.24 (t, *J* = 6.4 Hz, 2H), 7.39 (s, 1H), 7.52 - 7.60 (m, 2H), 7.89 - 7.91 (m, 1H), 8.20 (s, 1H), 8.28 - 8.31 (m, 1H); ^{13}C NMR (100MHz, Chloroform-*d*) δ (ppm) : 22.65, 25.27, 37.94, 52.25, 66.76, 103.42, 122.22, 123.33, 127.04, 127.46, 127.54, 127.95, 128.97, 133.39, 154.98, 167.51.

Methyl 1,4-dimethoxy-2-naphthoate (S1e)

1,4-Dihydroxy-2-naphthoic acid (1.0 g, 4.9 mmol) in acetone (10 ml) was reacted with dimethyl sulfate (3.2 ml, 24.5 mmol) in the presence of K_2CO_3 (6.1 g, 44.1 mmol) as described above to give compound **S1e** as a pale red solid (90%). R_f = 0.59 (10% EtOAc/hexane); ^{1}H NMR (400MHz, Chloroform-*d*) δ (ppm) : 4.00 (s, 3H), 4.01 (s, 3H), 4.02 (s, 3H), 7.16 (s, 1H), 7.58 - 7.62 (m, 2H), 8.21 - 8.27 (m, 2H); ^{13}C NMR (100MHz, Chloroform-*d*) δ (ppm) : 52.33, 55.75, 63.35, 103.49, 118.63, 122.28, 123.48, 127.06, 127.76, 128.76, 129.17, 151.35, 152.03, 166.83.

Isopropyl 1,4-diisopropoxy-2-naphthoate (S1f)

1,4-Dihydroxy-2-naphthoic acid (0.8 g, 3.9 mmol) in DMF (12 ml) was reacted with 2-bromopropane (1.46 ml, 15.6 mmol) in the presence of Cs_2CO_3 (6.4 g, 19.6 mmol) as described above to give compound **S1f** as a dark yellow oil (85%). R_f = 0.59 (20% EtOAc/hexane); ^{1}H NMR (400MHz, Chloroform-*d*) δ (ppm) : 1.31 (d, *J* = 6.0 Hz, 6H), 1.42 (d, *J* = 6.0 Hz, 6H), 1.44 (d, *J* = 6.0 Hz, 6H), 4.37 - 4.43 (m, 1H), 4.72 - 4.78 (m, 1H), 5.26 - 5.36 (m, 1H), 7.14 (s, 1H), 7.48 - 7.55 (m, 2H), 8.20 - 8.25 (m, 2H); ^{13}C NMR (100MHz, Chloroform-*d*) δ (ppm) : 21.93, 22.11, 22.46, 68.61, 70.71, 78.03, 106.43, 120.32, 122.25, 124.22, 126.34, 127.18, 129.31, 130.76, 148.32, 149.12, 167.06.

Isoamyl 1,4-bis(isoamyloxy)-2-naphthoate (S1g)

1,4-Dihydroxy-2-naphthoic acid (2.04 g, 10.0 mmol) in DMF (30 ml) was reacted with 1-bromo-3-methyl butane (4.8 ml, 40.0 mmol) in the presence of K_2CO_3 (5.53 g, 40.0 mmol) as described above to give compound **S1g** as a yellow oil (81%). R_f = 0.37 (5% EtOAc/hexane); ^{1}H NMR (400MHz, Chloroform-*d*) δ (ppm) : 0.99 - 1.02 (m, 18H), 1.70 - 1.75 (m, 2H), 1.79 – 1.88 (m, 5H), 1.90 – 1.99 (m, 2H), 4.06 (t, *J* = 7.0 Hz, 2H), 4.18 (t, *J* = 6.4 Hz, 2H), 4.41 (t, *J* = 7.0 Hz, 2H), 7.14 (s, 1H), 7.56 - 7.58 (m, 2H), 8.18 – 8.21 (m, 1H), 8.25 – 8.27 (m, 1H); ^{13}C NMR (100MHz, Chloroform-*d*) δ (ppm) : 22.56, 22.65, 22.78, 25.10, 25.22, 25.27, 37.49, 37.96, 39.12, 63.93, 66.71, 74.88, 104.30, 119.25, 122.32, 123.45, 126.82, 127.46, 128.80, 129.48, 150.63, 150.66, 166.86.

Octyl 1,4-bis(octyloxy)-2-naphthoate (S1h)

1,4-Dihydroxy-2-naphthoic acid (0.5 g, 2.5 mmol) in DMF (25 ml) was reacted with 1-bromooactane (1.7 ml, 9.8 mmol) in the presence of Cs_2CO_3 (4.8 g, 14.7 mmol) as described above to give compound **S1h** as a yellow oil (95%). R_f = 0.83 (20% EtOAc/hexane); ^{1}H NMR (400MHz, Chloroform-*d*) δ (ppm) : 0.86 - 0.91 (m, 9H), 1.29 - 1.45 (m, 30H), 1.78 - 1.85 (m, 2H), 1.88 - 1.97 (m, 4H), 4.03 (t, *J* = 6.8 Hz, 2H), 4.14 (t, *J* = 6.4 Hz, 2H), 4.37 (t, *J* = 6.8 Hz, 2H), 7.13 (s, 1H), 7.54 - 7.59 (m, 2H), 8.18 - 8.22 (m, 1H), 8.25 - 8.29 (m, 1H); ^{13}C NMR (100MHz, Chloroform-*d*) δ (ppm) : 14.10, 22.67, 25.69, 26.11, 26.26, 28.67, 28.81, 29.14, 29.24, 29.32, 29.38, 29.59, 30.40, 31.75, 31.81, 31.83, 31.86, 65.41, 68.03, 68.38, 76.34, 104.40, 119.31, 122.30, 123.51, 126.78, 127.43, 128.81, 129.49, 150.60, 150.65, 166.89.

1.2. General synthetic procedure for 1- or 4-alkoxynaphthalene-2-methanol or 1,4-dialkoxynaphthalene-2-methanol (**5a-5h**)

To the corresponding starting naphthoate (**S1)** in anhydrous THF, $LiAlH_4$ (1.0 M in THF) was slowly added dropwise at 0 °C. At room temperature, the solution was stirred until the starting material disappeared. After the reaction was completed, water (1.5 eq) was slowly added at 0 °C. After 10 minutes, 10 wt% NaOH solution (3 eq) was slowly added dropwise, followed by adding water (1.5 eq) slowly dropwise additional 10 minutes later. The reaction mixture was dried over Na_2SO_4 and concentrated under reduced pressure. The residue was purified by column chromatography to give methanol derivatives (**5**).

(1-Methoxynaphthalen-2-yl)methanol (5a)

S1a (0.3 g, 1.39 mmol) in anhydrous THF (7 ml) was reacted with $LiAlH_4$ (1.0 M in THF, 2.1 ml, 2.1 mmol) as described above to give compound **5a** as a white solid (86%). R_f = 0.20 (20% EtOAc/hexane); ^{1}H NMR (400MHz, Chloroform-*d*) δ (ppm) : 4.00 (s, 3H), 4.92 (s, 2H), 7.48 - 7.55 (m, 3H), 7.64 - 7.66 (m, 1H), 7.84 - 7.86 (m, 1H), 8.10-8.13 (m, 1H); ^{13}C NMR (100MHz, Chloroform-*d*) δ (ppm) : 60.97, 62.65, 122.02, 124.43, 126.10, 126.18, 126.62, 127.79, 128.08, 128.94, 134.72, 153.78.

(1-(Isoamyloxy)naphthalen-2-yl)methanol (5b)

S1b (0.7 g, 1.22 mmol) in anhydrous THF (12.2 ml) was reacted with $LiAlH_4$ (1.0 M in THF, 3.05 ml, 3.05 mmol) as described above to give compound **5b** as a clear oil (83%). R_f = 0.34 (20% EtOAc/hexane); ^{1}H NMR (400MHz, Chloroform-*d*) δ (ppm) : 1.02 (d, *J* = 6.8 Hz, 6H), 1.83 - 1.88 (m, 2H), 1.90 - 1.98 (m, 1H), 2.18 (t, *J* = 5.6 Hz, 1H), 4.07 (t, *J* = 6.6 Hz, 2H), 4.90 (d, *J* = 5.2 Hz, 2H), 7.46 - 7.54 (m, 3H), 7.62 - 7.64 (m, 1H), 7.83 - 7.85 (m, 1H), 8.08 - 8.10 (m, 1H); ^{13}C NMR (100MHz, Chloroform-*d*) δ (ppm) : 22.72, 25.07, 39.30, 61.22, 74.07, 122.13, 124.22, 126.00, 126.12, 126.61, 128.06, 128.12, 129.07, 134.68, 153.02.

(4-Methoxynaphthalen-2-yl)methanol (5c)

S1c (0.3 g, 0.46 mmol) in anhydrous THF (2.3 ml) was reacted with $LiAlH_4$ (1.0 M in THF, 0.69 ml, 0.69 mmol) as described above to give compound **5c** as a yellow oil (97%). R_f = 0.28 (10% EtOAc/hexane); ^{1}H NMR (400MHz, Chloroform-*d*) δ (ppm) : 4.02 (s, 3H), 4.83 (s, 2H), 6.85 (s, 1H), 7.38 (s, 1H), 7.44 - 7.51 (m, 2H), 7.76 - 7.78 (m, 1H), 8.22 - 8.24 (m, 1H); ^{13}C NMR (100MHz, Chloroform-*d*) δ (ppm) : 55.54, 65.91, 102.91, 117.70, 121.94, 125.18, 125.26, 126.75, 127.44, 134.25, 138.59, 155.81.

(4-(Isoamyloxy)naphthalen-2-yl)methanol (5d)

S1d (0.2 g, 0.73 mmol) in anhydrous THF (3.65 ml) was reacted with $LiAlH_4$ (1.0 M in THF, 1.1 ml, 1.1 mmol) as described above to give compound **5d** as a yellow oil (98%). R_f = 0.39 (20 % EtOAc/hexane); ^{1}H NMR (400MHz, Chloroform-*d*) δ (ppm) : 1.03 (d, *J* = 6.8 Hz, 6H), 1.81 - 1.86 (m, 2H), 1.94 - 2.01 (m, 1H), 4.18 (t, *J* = 6.4 Hz, 2H), 4.81 (s, 2H), 6.84 (s, 1H), 7.36 (s, 1H), 7.43 - 7.51 (m, 2H), 7.75 - 7.78 (m, 1H), 8.24 - 8.26 (m, 1H); ^{13}C NMR (100MHz, Chloroform-*d*) δ (ppm) : 22.65, 25.26, 38.00, 65.90, 66.53, 103.55, 117.39, 122.07, 125.11, 125.32, 126.66, 127.41, 134.25, 138.64, 155.27.

(1,4-Dimethoxynaphthalen-2-yl)methanol (5e)

S1e (0.2 g, 0.81 mmol) in anhydrous THF (0.1 ml) was reacted with $LiAlH_4$ (1.0 M in THF, 0.97 ml, 0.97 mmol) as

described above to give compound **5e** as a white solid (94%). R_f = 0.22 (20% EtOAc/hexane); ^{1}H NMR (400MHz, Chloroform-*d*) δ (ppm) : 3.93 (s, 3H), 4.00 (s, 3H), 4.90 (s, 2H), 6.82 (s, 1H), 7.47 - 7.57 (m, 2H), 8.03 - 8.06 (m, 1H), 8.23 - 8.25 (m, 1H); ^{13}C NMR (100MHz, Chloroform-*d*) δ (ppm) : 55.67, 61.01, 62.63, 103.75, 121.74, 122.39, 125.51, 126.24, 126.70, 128.39, 128.46, 146.94, 152.14.

(1,4-Diisopropoxynaphthalen-2-yl)methanol (5f)

S1f (0.2 g, 0.61 mmol) in anhydrous THF (3.05 ml) was reacted with $LiAlH_4$ (1.0 M in THF, 0.91 ml, 0.91 mmol) as described above to give compound **5f** as a white solid (90%). R_f = 0.32 (20% EtOAc/hexane); ^{1}H NMR (400MHz, Chloroform-*d*) δ (ppm) : 1.37 (d, *J* = 6.0 Hz, 6H), 1.45 (d, *J* = 5.6 Hz, 6H), 4.34 - 4.40 (m, 1H), 4.70 - 4.76 (m, 1H), 4.88 (s, 2H), 6.86 (s, 1H), 7.42 - 7.51 (m, 2H), 8.00 - 8.02 (m, 1H), 8.23 - 8.25 (m, 1H); ^{13}C NMR (100MHz, Chloroform-*d*) δ (ppm) : 22.18, 22.57, 61.70, 70.58, 76.91, 106.41, 122.53, 122.60, 125.13, 126.12, 127.20, 129.28, 129.63, 144.13, 150.01.

(1,4-Bis(isoamyloxy)naphthalen-2-yl)methanol (5g)

S1g (0.7 g, 1.22 mmol) in anhydrous THF (12.2 ml) was reacted with $LiAlH_4$ (1.0 M in THF, 3.05 ml, 3.05 mmol) as described above to give compound **5g** as a white solid (83%). R_f = 0.27 (5% EtOAc/hexane); ^{1}H NMR (400MHz, Chloroform-*d*) δ (ppm) : 1.01 - 1.02 (m, 12H), 1.79 - 1.85 (m, 4H), 1.90 - 2.00 (m, 2H), 2.14 (t, *J* = 6.0 Hz, 1H), 3.99 (t, *J* = 6.8 Hz, 2H), 4.16 (t, *J* = 6.4 Hz, 2H), 4.88 (d, *J* = 6.0 Hz, 2H), 6.81 (s, 1H), 7.45 - 7.55 (m, 2H), 8.00 - 8.02 (m, 1H), 8.24 - 8.26 (m, 1H); ^{13}C NMR (100MHz, Chloroform-*d*) δ (ppm) : 22.66, 22.73, 25.07, 25.27, 38.07, 39.28, 61.35, 66.67, 73.97, 104.46, 121.80, 122.49, 125.34, 126.38, 126.52, 128.61, 128.74, 145.97, 151.53.

(1,4-Bis(octyloxy)naphthalen-2-yl)methanol (5h)

S1h (0.26 g, 0.48 mmol) in anhydrous THF (2.4ml) was reacted with $LiAlH_4$ (1.0 M in THF, 0.72 ml, 0.72 mmol) as described above to give compound **5h** as a yellow oil (91%). R_f = 0.50 (20% EtOAc/hexane); ^{1}H NMR (400MHz, Chloroform-*d*) δ (ppm) : 0.88 – 0.92 (m, 6H), 1.26 – 1.44 (m, 16H), 1.51 – 1.60 (m, 4H), 1.87 – 1.95 (m, 4H), 3.95 (t, *J* = 6.8 Hz, 2H), 4.11 (t, *J* = 6.6 Hz, 2H), 4.87 (s, 2H), 6.79 (s, 1H), 7.45 – 7.54 (m, 2H), 8.00 – 8.03 (m, 1H), 8.25 – 8.27 (m, 1H); ^{13}C NMR (100MHz, Chloroform-*d*) δ (ppm) : 14.10, 22.66, 26.16, 26.26, 29.27, 29.30, 29.37, 29.53, 30.48, 31.83, 61.29, 68.32, 75.52, 104.50, 121.82, 122.45, 125.30, 126.36, 126.48, 128.64, 128.72, 145.84, 151.51.

1.3. General synthetic procedure for 1- or 4-alkoxynaphthalene-2-methylbromide or 1,4-dialkoxynaphthalene-2-methylbromide (**12a-12h**)

Method A: Starting material (**5**), PPh_3, and TBAI (tetrabutyl ammonium iodide) were dissolved in 1,2-dibromoethane and refluxed until the starting material disappeared. After the reaction was completed, the reaction solution was concentrated under reduced pressure and purified by column chromatography to give the corresponding product (**12**).

Method B: To the starting material (**5**) in anhydrous dichloromethane, PBr_3 was slowly added dropwise at 0 °C. The reaction solution was warmed to room temperature and then stirred until the starting material disappeared. When the starting material disappeared as monitored by TLC, the reaction solution was diluted with dichloromethane, poured into water, and extracted with dichloromethane three times. The combined organic layer was washed with an aqueous saturated $NaHCO_3$ solution, dried over Na_2SO_4, filtered, and concentrated under reduced pressure. The residue was purified by column chromatography to give the corresponding product (**12)**.

2-(Bromomethyl)-1-methoxynaphthalene (12a)

5a (0.17 g, 0.92 mmol), PPh_3 (0.29 g, 1.1 mmol) and TBAI (0.41 g, 1.1 mmol) in 1,2-dibromoethane (9 ml) were reacted as **method A** to give compound **12a** as a white solid (74%). R_f = 0.68 (20% EtOAc/hexna); ^{1}H NMR (400MHz, Chloroform-*d*) δ (ppm) : 4.08 (s, 3H), 4.78 (s, 2H), 7.45 - 7.56 (m, 3H), 7.61 -7.64 (m, 1H), 7.82 - 7.84 (m, 1H), 8.10 - 8.13 (m, 1H); ^{13}C NMR (100MHz, Chloroform-*d*) δ (ppm) : 28.40, 62.47, 122.46, 124.76, 126.37, 126.80, 127.82, 127.88, 128.15, 135.08, 154.33.

2-(Bromomethyl)-1-(isoamyloxy)naphthalene (12b)

5b (0.25 g, 1.02 mmol), PPh_3 (0.32 g, 1.23 mmol) and TBAI (0.45 g, 1.23 mmol) in 1,2-dibromoethane (10 ml) were reacted as **method A** to give compound **12b** as a yellow oil (81%). R_f = 0.67 (10% EtOAc/hexane); ^{1}H NMR (400MHz, Chloroform-*d*) δ (ppm) : 1.04 – 1.06 (m, 6H), 1.88 - 2.00 (m, 3H). 4.17 (t, *J* = 6.8 Hz, 2H), 4.77 (s, 2H), 7.46 - 7.53 (m, 3H), 7.60 - 7.62 (m, 1H), 7.81 - 7.83 (m, 1H), 8.08 - 8.11 (m, 1H); ^{13}C NMR (100MHz, Chloroform-*d*) δ (ppm) : 22.77, 25.09, 28.67, 39.23, 73.80, 122.59, 124.54, 126.25, 126.34, 126.74, 127.96, 128.10, 128.19, 135.01, 153.63.

3-(Bromomethyl)-1-methoxynaphthalene (12c)

5c (0.36 g, 1.9 mmol), PPh_3 (0.6 g, 2.3 mmol) and TBAI (0.85 g, 2.3 mmol) in 1,2-dibromoethane (19 ml) were reacted as **method A** to give compound **12c** as a white solid (90%). R_f = 0.41 (5% EtOAc/hexane); ^{1}H NMR (400MHz, Chloroform-*d*) δ (ppm) : 4.03 (s, 3H), 4.65 (s, 2H), 6.82 (s, 1H), 7.43 (s, 1H), 7.45 - 7.52 (m, 2H), 7.75 - 7.77 (m, 1H), 8.21 - 8.23 (m, 1H); ^{13}C NMR (100MHz, Chloroform-*d*) δ (ppm) : 34.87, 55.59, 104.43, 120.25, 121.99, 125.93, 127.02, 127.58, 134.02, 135.20, 155.91.

3-(Bromomethyl)-1-(isoamyloxy)naphthalene (12d)

5d (0.48 g, 1.96 mmol), PPh_3 (0.62 g, 2.36 mmol) and TBAI (0.87 g, 2.36 mmol) in 1,2-dibromoethane (20 ml) were reacted as **method A** to give compound **12d** as a white solid (94%). R_f = 0.91 (10% EtOAc/hexane); ^{1}H NMR (400MHz, Chloroform-*d*) δ (ppm) : 1.03 (d, *J* = 6.4 Hz, 6H), 1.82 - 1.87 (m, 2H), 1.93 - 2.02 (m, 1H), 4.19 (t, *J* = 6.6 Hz, 2H), 4.65 (s, 2H), 6.82 (s, 1H), 7.41 (s, 1H), 7.45-7.52 (m, 2H), 7.74 - 7.76 (m, 1H), 8.23 - 8.25 (m, 1H); ^{13}C NMR (100MHz, Chloroform-*d*) δ (ppm) : 22.67, 25.28, 34.94, 37.97, 66.61, 105.08, 119.97, 122.14, 125.66, 125.81, 126.95, 127.55, 134.05, 135.24, 155.40.

2-(Bromomethyl)-1,4-dimethoxynaphthalene (12e)

5e (0.1 g, 0.46 mmol) and PBr_3 (0.05ml, 0.55 mmol) in anhydrous dichloromethane (4.6 ml) were reacted as **method B** to give compound **12e** as a light brown solid (76%). R_f = 0.67 (10% EtOAc/hexane); ^{1}H NMR (400MHz, Chloroform-*d*) δ (ppm) : 4.00 (s, 3H), 4.01 (s, 3H), 4.77 (s, 2H), 6.73 (s, 1H), 7.48 - 7.58 (m, 2H), 8.04 - 8.06 (m, 1H), 8.21 - 8.24 (m, 1H); ^{13}C NMR (100MHz, Chloroform-*d*) δ (ppm) : 29.05, 55.68, 62.39, 104.69, 122.23, 122.48, 125.68, 126.19, 126.96, 128.37, 147.82, 152.15.

2-(Bromomethyl)-1,4-diisopropoxynaphthalene (12f)

5f (0.17 g, 0.61 mmol) and PBr_3 (0.06ml, 0.68 mmol) in anhydrous dichloromethane (6 ml) were reacted as **method B** to give compound **12f** as a yellow solid (85%). R_f = 0.75 (20% EtOAc/hexane); ^{1}H NMR (400MHz, Chloroform-*d*) δ (ppm) : 1.40 (d, *J* = 6.0 Hz, 6H), 1.46 (d, *J* = 6.4 Hz, 6H), 4.44 - 4.51 (m, 1H), 4.70 - 4.77 (m, 3H), 6.80 (s, 1H), 7.44 - 7.52 (m, 2H), 8.02 - 8.04 (m, 1H), 8.21 - 8.23 (m, 1H); ^{13}C NMR (100MHz, Chloroform-*d*) δ (ppm) : 22.06, 22.74, 30.28, 70.49, 76.89, 107.55, 122.59, 123.11, 125.84, 125.98, 126.37, 127.77, 129.72, 144.93, 149.89.

2-(Bromomethyl)-1,4-bis(isoamyloxy)naphthalene (12g)

5g (1.65 g, 5.0 mmol) and PBr_3 (0.57 ml, 6.0 mmol) in anhydrous dichloromethane (50 ml) were reacted as **method B** to give compound **12g** as a yellow solid (90%). R_f = 0.68 (10% EtOAc/hexane); ^{1}H NMR (400MHz, Chloroform-*d*) δ (ppm) : 1.01 - 1.03 (m, 12H), 1.80 - 1.89 (m, 4H), 1.93 – 2.00 (m, 2H), 4.09 (t, *J* = 6.8 Hz, 2H), 4.15 (t, *J* = 6.4 Hz, 2H), 4.76 (s, 2H), 6.72 (s, 1H), 7.47 - 7.56 (m, 2H), 8.01 - 8.03 (m, 1H), 8.22 - 8.25 (m, 1H); ^{13}C NMR (100MHz, Chloroform-*d*) δ (ppm) : 22.68, 22.78, 25.10, 25.27, 29.40, 38.02, 39.24, 66.69, 73.65, 105.42, 122.32, 122.55, 125.69, 126.04, 126.77, 127.10, 128.75, 146.89, 151.50.

2-(Bromomethyl)-1,4-bis(octyloxy)naphthalene (12h)

5h (0.1 g, 0.24 mmol) and PBr_3 (0.03 ml, 0.29 mmol) in anhydrous dichloromethane (2.4ml) were reacted as **method B** to give compound **12h** as a brown solid (80%). R_f = 0.79 (20% EtOAc/hexane); ^{1}H NMR (400MHz, Chloroform-*d*) δ (ppm) : 0.85 – 0.92 (m, 6H), 1.29 – 1.43 (m, 16H), 1.52 – 1.63 (m, 4H), 1.88 – 1.99 (m, 4H), 4.06 (t, *J* = 6.8 Hz, 2H), 4.12 (t, *J* = 6.4 Hz, 2H), 4.75 (s, 2H), 6.71 (s, 1H), 7.47 – 7.55 (m, 2H), 8.01 – 8.04 (m, 1H), 8.23 – 8.25 (m, 1H); ^{13}C NMR (100MHz, Chloroform-*d*) δ (ppm) : 14.12, 22.68, 26.13, 26.27, 29.27, 29.30, 29.39, 29.42, 29.52, 30.45, 31.84, 68.35, 75.19,

105.49, 122.36, 122.53, 125.70, 126.02, 126.74, 127.09, 128.74, 146.80, 151.50.

2. Synthesis of *N*-substituted imidazoles

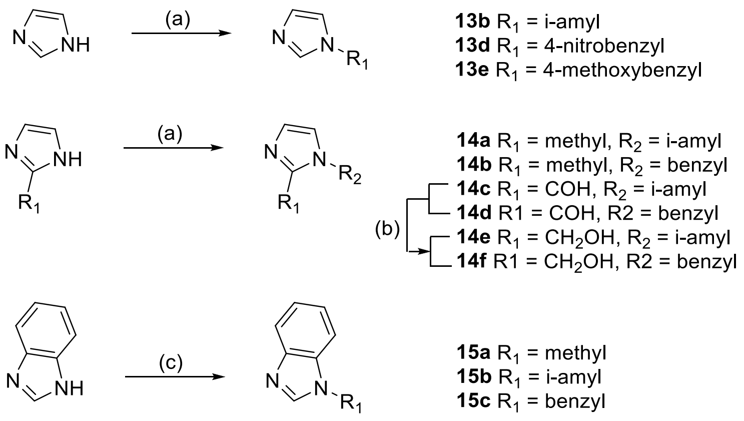

Reagents and conditions. (a) R-X, KOH, anhydrous DMSO, rt or R-X, K_2CO_3, anhydrous ACN, reflux; **(b)** $LiAlH_4$, anhydrous THF, rt; **(c)** TMEDA, dimehtyl carbonate, DMF, reflux or R-X, KOH, anhydrous DMSO, rt.

Scheme S2. Synthesis of *N*-substituted imidazoles

Method C : To an imidazole in anhydrous acetonitrile, K_2CO_3 was added in two portions. After 30 minutes, alkyl or benzylic halide was added dropwise and then the reaction was refluxed at 80 °C until the starting material disappeared. After the completion of the reaction, the mixture was filtered and poured into water, followed by extraction with ethyl acetate three times. The combined organic layer was washed with brine, dried over Na_2SO_4, filtered, and then concentrated under reduced pressure. The residue was purified by column chromatography to give the corresponding product.

Method D : Imidazole and KOH were dissolved in anhydrous DMSO and it was stirred at room temperature. After 2 hours, alkyl halide was added dropwise and the reaction mixrure was stirred at room temperature until the starting material disappeared. After the completion of the reaction, it was poured into water and extracted with dichloromethane three times. The combined organic layer was washed with brine, dried over Na_2SO_4, filtered and concentrated under reduced pressure. The residue was purified by column chromatography to give the corresponding product [26].

1-Isoamyl-1*H*-imidazole (13b)

Imidazole (1.0 g, 14.7 mmol) and 1-bromo-3-methyl butane (1.93 ml, 14.69 mmol) in DMSO (29.4 ml) were reacted in the presence of KOH (1.23 g, 22.03 mmol) as **method D** to give compound **13b** as a yellow oil (84%). R_f = 0.38 (10% MeOH/DCM); ^{1}H NMR (400MHz, Chloroform-*d*) δ (ppm) : 0.93 (d, *J* = 6.8 Hz, 6H), 1.52 - 1.59 (m, 1H), 1.63 - 1.69 (m, 2H), 3.93 (t, *J* = 7.4 Hz, 2H), 6.89 (s, 1H), 7.04 (s, 1H), 7.45 (s, 1H); ^{13}C NMR (100MHz, Chloroform-*d*) δ (ppm) : 22.20, 25.34, 39.86, 45.20, 118.69, 129.31, 136.95.

1-(4-Nitrobenzyl)-1*H*-imidazole (13d)

Imidazole (0.68 g, 10.0 mmol) and 4-nitrobenzyl bromide (1.08 g, 5.0 mmol) in anhydrous acetonitrile (33.3 ml) were reacted in the presence of K_2CO_3 (2.07 g, 15.0 mmol) as **method C** to give compound **13d** as a brown solid (77%). Rf = 0.36 (6% MeOH/DCM); ^{1}H NMR (400MHz, DMSO-d_6) δ (ppm) : 5.37 (s, 2H), 6.96 (s, 1H), 7.23 (s, 1H), 7.43 – 7.46 (m, 2H), 7.82 (s, 1H), 8.20 – 8.23 (m, 2H); ^{13}C NMR (100MHz, DMSO-d_6) δ (ppm) : 48.69, 119.78, 123.89, 128.44, 128.87, 137.67, 145.46, 146.97.

1-(4-Methoxybenzyl)-1*H*-imidazole (13e)

Imidazole (0.68 g, 10.0 mmol) and 4-methoxybenzyl bromide (0.68 ml, 5.0 mmol) in anhydrous acetonitrile (33.3 ml) were reacted in the presence of K_2CO_3 (2.07 g, 15.0 mmol) as **method C** to give compound **13e** as an ivory solid (86%). Rf = 0.36 (6% MeOH/DCM); ^{1}H NMR (400MHz, Chloroform-*d*) δ (ppm) : 3.79 (s, 3H), 5.04 (s, 2H), 6.86 – 6.89 (m, 3H), 7.06 – 7.11 (m, 3H), 7.51 (s, 1H); ^{13}C NMR (100MHz, Chloroform-*d*) δ (ppm) : 50.27, 55.27, 114.26, 119.03, 128.05, 128.78, 129.64, 137.17, 159.48.

1-Isoamyl-2-methyl-1*H*-imidazole (14a)

2-Methylimdazole (0.82 g, 10.0 mmol) and 1-bromo-3-methyl butane (0.62 ml, 5.0 mmol) in anhydrous acetonitrile (17 ml) were reacted in the presence of K_2CO_3 (2.07 g, 15.0 mmol) as **method C** to give compound **14a** as a yellow oil (66%). R_f = 0.33 (6% MeOH/DCM); ^{1}H NMR (400MHz, DMSO-*d*$_6$) δ (ppm) : 0.89 (d, *J* = 6.4 Hz, 6H), 1.49 – 1.53 (m, 3H), 2.25 (s, 3H), 3.83 (t, *J* = 7.4 Hz, 2H), 6.68 (d, *J* = 1.2 Hz, 1H), 7.01 (d, *J* = 1.2 Hz, 1H); ^{13}C NMR (100MHz, DMSO-*d*$_6$) δ (ppm) : 12.60, 22.23, 25.09, 39.14, 43.50, 119.43, 126.14, 143.38.

1-Benzyl-2-methyl-1*H*-imidazole (14b)

2-Methylimdazole (0.41 g, 5.0 mmol) and benzyl bromide (0.3 ml, 2.5 mmol) in anhydrous acetonitrile (17 ml) were reacted in the presence of K_2CO_3 (1.04 g, 7.5 mmol) as **method C** to give **14b** as a yellow oil (73%). R_f = 0.64 (12% MeOH/DCM); ^{1}H NMR (400MHz, DMSO-*d*$_6$) δ (ppm) : 2.20 (s, 3H), 5.13 (s, 2H), 6.75 (d, *J* = 1.2 Hz, 1H), 7.11 – 7.13 (m, 3H), 7.27 – 7.29 (m, 1H), 7.32 – 7.36 (m, 2H); ^{13}C NMR (100MHz, DMSO-*d*$_6$) δ (ppm) : 12.73, 48.60, 120.35, 126.40, 126.94, 127.51, 128.72, 137.53, 143.90.

1-Isoamyl-1*H*-imidazole-2-carbaldehyde (14c)

2-Imidazolecarboxaldehyde (0.2 g, 2.1 mmol) and 1-bromo-3-methyl butane (0.17 ml, 1.39 mmol) in anhydrous acetonitrile (6.9 ml) were reacted in the presence of K_2CO_3 (0.57 g, 4.16 mmol) as **method C** to give compound **14c** as a green oil (80%). R_f = 0.45 (6% MeOH/DCM); ^{1}H NMR (400MHz, Chloroform-*d*) δ (ppm) : 0.96 (d, *J* = 6.4 Hz, 6H), 1.63 – 1.69 (m, 3H), 4.40 (t, *J* = 7.6 Hz, 2H), 7.15 (s, 1H), 7.27 (s, 1H), 9.81 (s, 1H); ^{13}C NMR (100MHz, Chloroform-*d*) δ (ppm) : 22.30, 25.58, 39.90, 46.19, 126.07, 131.56, 143.27, 181.98.

1-Benzyl-1*H*-imidazole-2-carbaldehyde (14d)

2-Imidazolecarboxaldehyde (0.96 g, 10.0 mmol) and benzyl bromide (0.59 ml, 5.0 mmol) in anhydrous acetonitrile (33 ml) were reacted in the presence of K_2CO_3 (2.07 g, 15.0 mmol) as **method C** to give compound **14d** as a green oil (98%). R_f = 0.44 (6% MeOH/DCM); ^{1}H NMR (400MHz, Chloroform-*d*) δ (ppm) : 5.61 (s, 2H), 7.14 (s, 1H), 7.19 – 7.21 (m, 2H), 7.30 – 7.35 (m, 4H), 9.85 (s, 1H); ^{13}C NMR (100MHz, Chloroform-*d*) δ (ppm) : 50.87, 126.22, 127.70, 128.34, 128.95, 131.87, 135.71, 143.25, 182.20.

(1-Isoamyl-1*H*-imidazol-2-yl)methanol (14e)

S4a (0.5 g, 3.0 mmol) in anhydrous THF (15 ml) was reacted with $LiAlH_4$ (3.6 ml, 3.6 mmol) as described [1.2.] to give compound **14e** as a white solid (77%). R_f = 0.69 (13% MeOH/DCM); ^{1}H NMR (400MHz, DMSO-*d*$_6$) δ (ppm) : 0.90 (d, *J* = 6.4 Hz, 6H), 1.50 – 1.62 (m, 3H), 3.97 (t, *J* = 7.6 Hz, 2H), 4.45 (s, 2H), 5.26 (s, 1H), 6.75 (d, *J* = 1.6 Hz, 1H), 7.10 (d, *J* = 1.2 Hz, 1H); ^{13}C NMR (100MHz, Chloroform-*d*) δ (ppm) : 22.35, 25.68, 39.90, 44.42, 55.76, 119.82, 126.77, 147.52.

(1-Benzyl-1*H*-imidazol-2-yl)methanol (14f)

S4b (0.2 g, 1.07 mmol) in anhydrous THF (5.4 ml) was reacted with $LiAlH_4$ (1.61 ml, 1.61 mmol) as described [1.2.] to give compound **14f** as a light brown solid (86%). R_f = 0.44 (6% MeOH/DCM); ^{1}H NMR (400MHz, DMSO-*d*$_6$) δ (ppm) : 4.45 (s, 2H), 5.23 (s, 2H), 6.82 (d, *J* = 1.6 Hz, 1H), 7.11 (d, *J* = 1.6Hz, 1H), 7.19 – 7.21 (m, 2H), 7.26 – 7.36 (m, 3H); ^{13}C NMR (100MHz, DMSO-*d*$_6$) δ (ppm) : 48.58, 55.70, 121.05, 126.55, 127.32, 127.55, 128.60, 137.59, 147.13.

1-Methyl-1*H*-benzo[*d*]imidazole (15a)

A mixture of benzimidazole (0.5 g, 4.23 mmol), TMEDA (50 mg, 0.42 mmol) and dimethyl carbonate (4.2 ml) in DMF (2.1 ml) was stirred under reflux until the starting material disappeared. After the reaction was completed, it was quenched with water and extracted with ethyl acetate three times. The combined organic layer was washed with water, dried over Na_2SO_4, filtered, and then concentrated under reduced pressure. The concentrate was purified by column chromatography to give compound **15a** as a brown oil (78%) [27]. R_f = 0.50 (10% MeOH/DCM); ^1H NMR (400MHz, Chloroform-*d*) δ (ppm) : 3.84 (s, 3H), 7.26 - 7.34 (m, 2H), 7.38 - 7.41 (m, 1H), 7.80 - 7.82 (m, 1H), 7.86 (s, 1H); ^{13}C NMR (100MHz, Chloroform-*d*) δ (ppm) : 30.97, 109.28, 120.24, 122.05, 122.89, 134.49, 143.48, 143.67.

1-Isoamyl-1*H*-benzo[*d*]imidazole (15b)

Benzimidazole (0.5 g, 4.23 mmol) and 1-bromo-3-methyl butane (0.53 ml, 4.23 mmol) in DMSO (8.5 ml) were reacted in the presence of KOH (0.36 g, 6.35 mmol) as **method D** to give compound **15b** as a brown oil (95%). R_f = 0.51 (10% MeOH/DCM); ^1H NMR (400MHz, Chloroform-*d*) δ (ppm) : 0.99 (d, *J* = 6.4 Hz, 6H), 1.59 - 1.65 (m, 1H), 1.76 - 1.81 (m, 2H), 4.18 (t, *J* = 7.4 Hz, 2H), 7.26 - 7.39 (m, 2H), 7.39 - 7.41 (m, 1H), 7.80 - 7.82 (m, 1H), 7.89 (s, 1H); ^{13}C NMR (100MHz, Chloroform-*d*) δ (ppm) : 22.27, 25.60, 38.57, 43.30, 109.57, 120.38, 121.95, 122.73, 133.76, 142.78, 143.87.

1-Benzyl-1*H*-benzo[*d*]imidazole (15c)

Benzimidazole (0.5 g, 4.23 mmol) and benzyl chloride (0.49 ml, 4.23 mmol) in DMSO (8.5 ml) were reacted in the presence of KOH (0.36 g, 6.35 mmol) as **method D** to give compound **14c** as an ivory solid (82%). R_f = 0.48 (10% MeOH/DCM); ^1H NMR (400MHz, Chloroform-*d*) δ (ppm) : 5.36 (s, 2H), 7.17 - 7.20 (m, 2H), 7.23 - 7.37 (m, 6H), 7.82 - 7.84 (m, 1H), 7.95 (s, 1H); ^{13}C NMR (100MHz, Chloroform-*d*) δ (ppm) : 48.82, 109.98, 120.45, 122.24, 123.06, 127.07, 128.25, 129.03, 133.92, 135.46, 143.20, 143.99.

Ⅱ. NMR Data

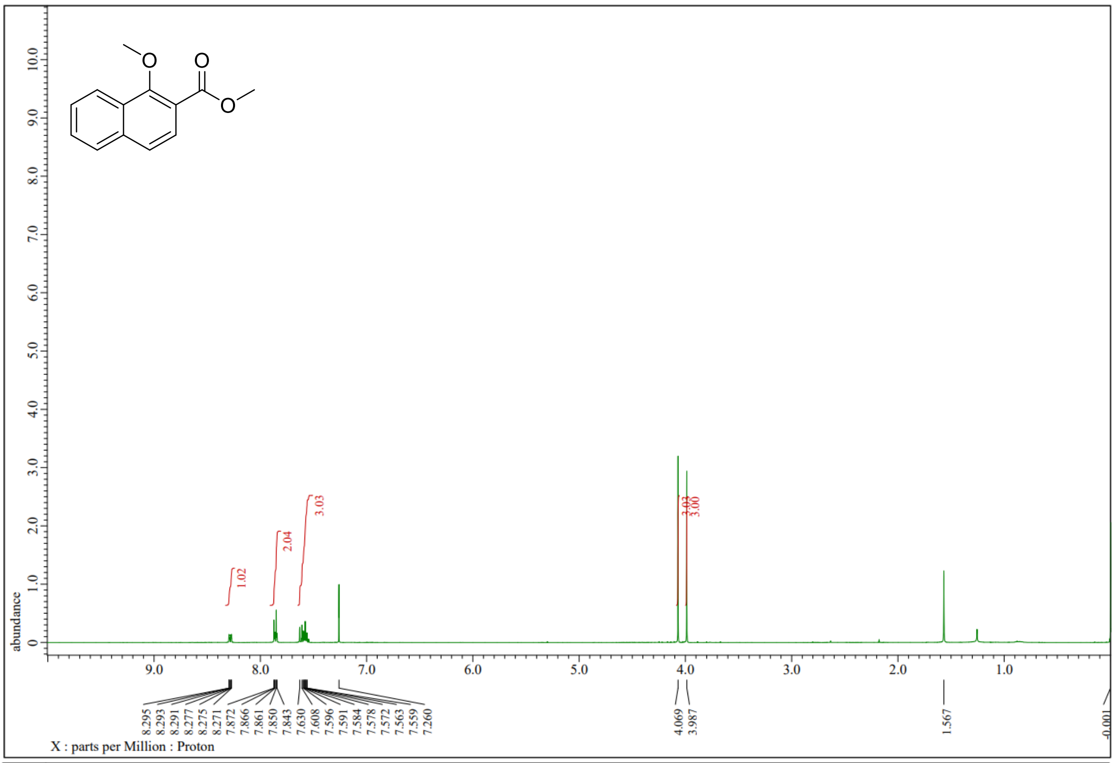

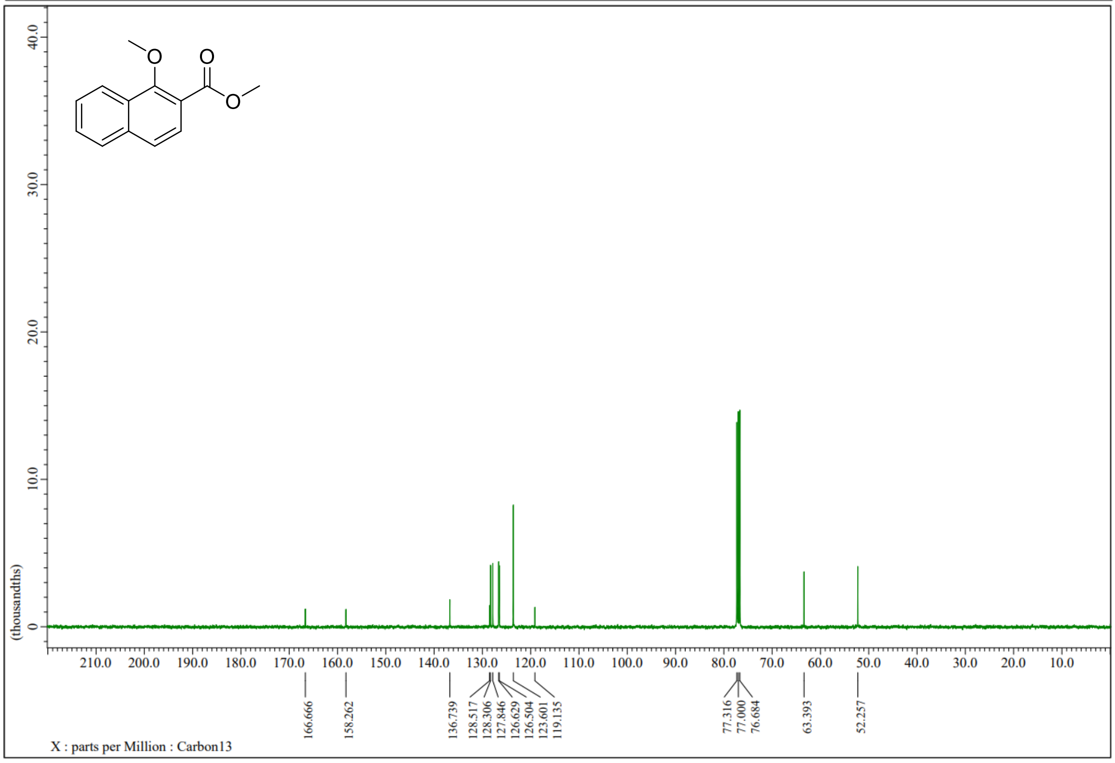

Figure S1. Methyl 1-methoxy-2-naphthoate **(S1a)**

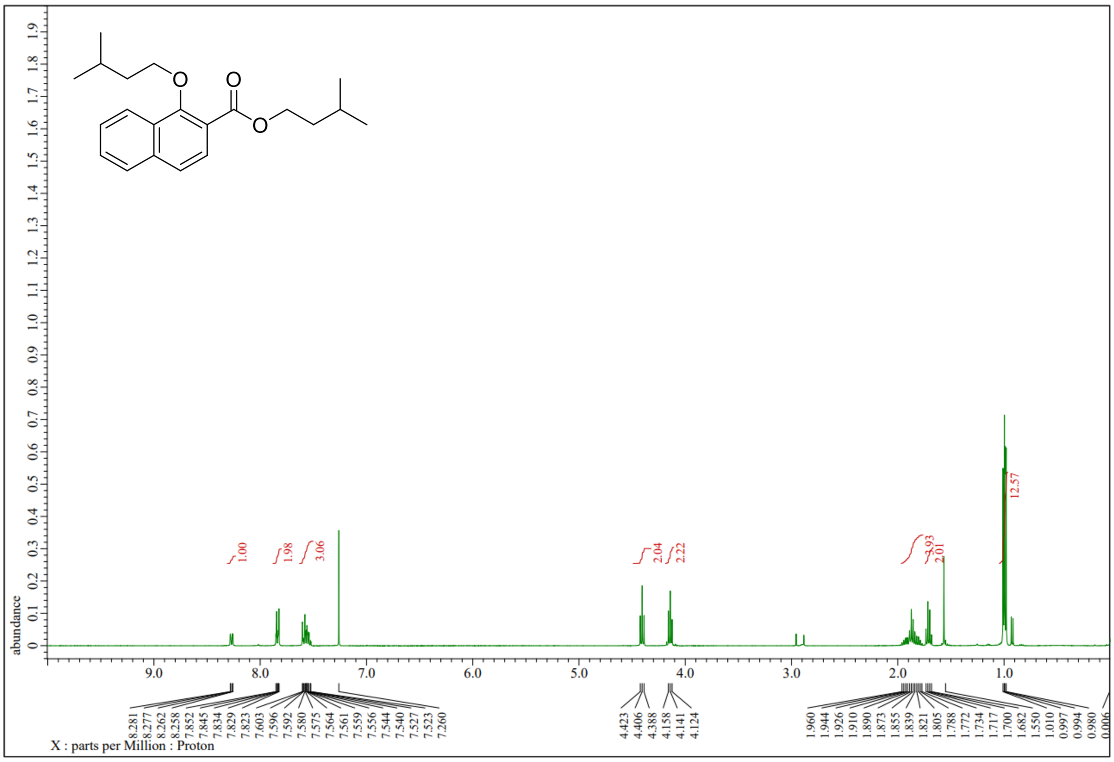

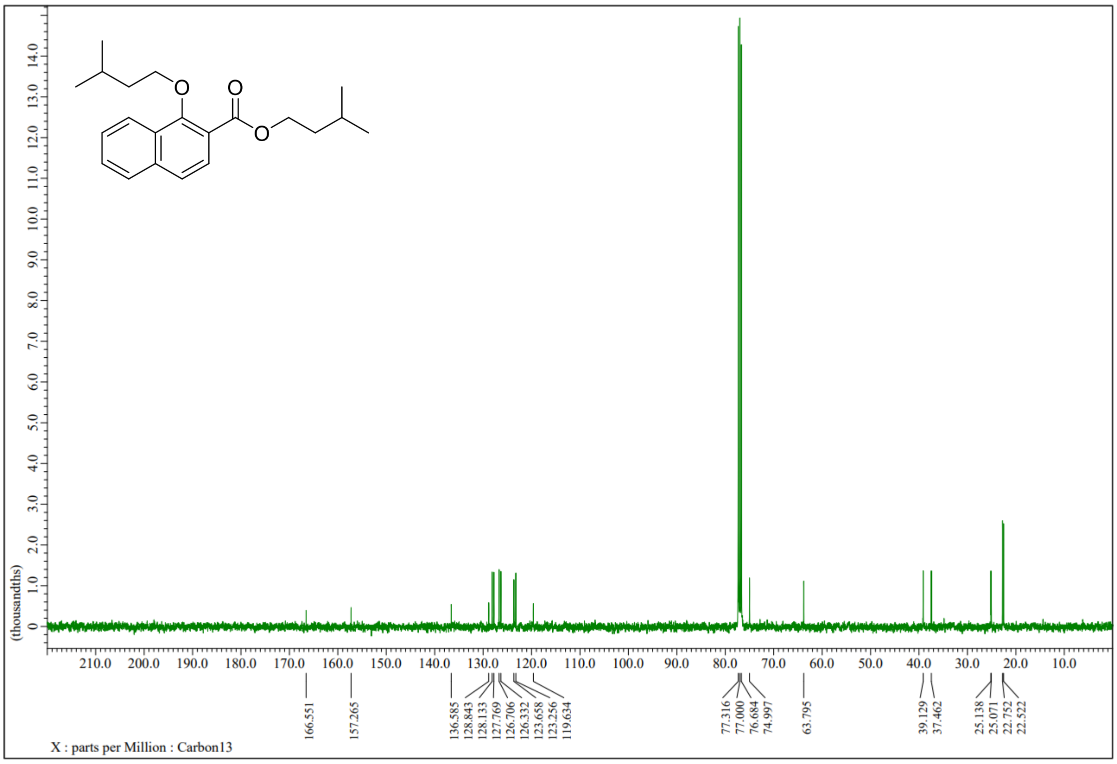

Figure S2. Isoamyl 1-(isoamyloxy)-2-naphthoate **(S1b)**

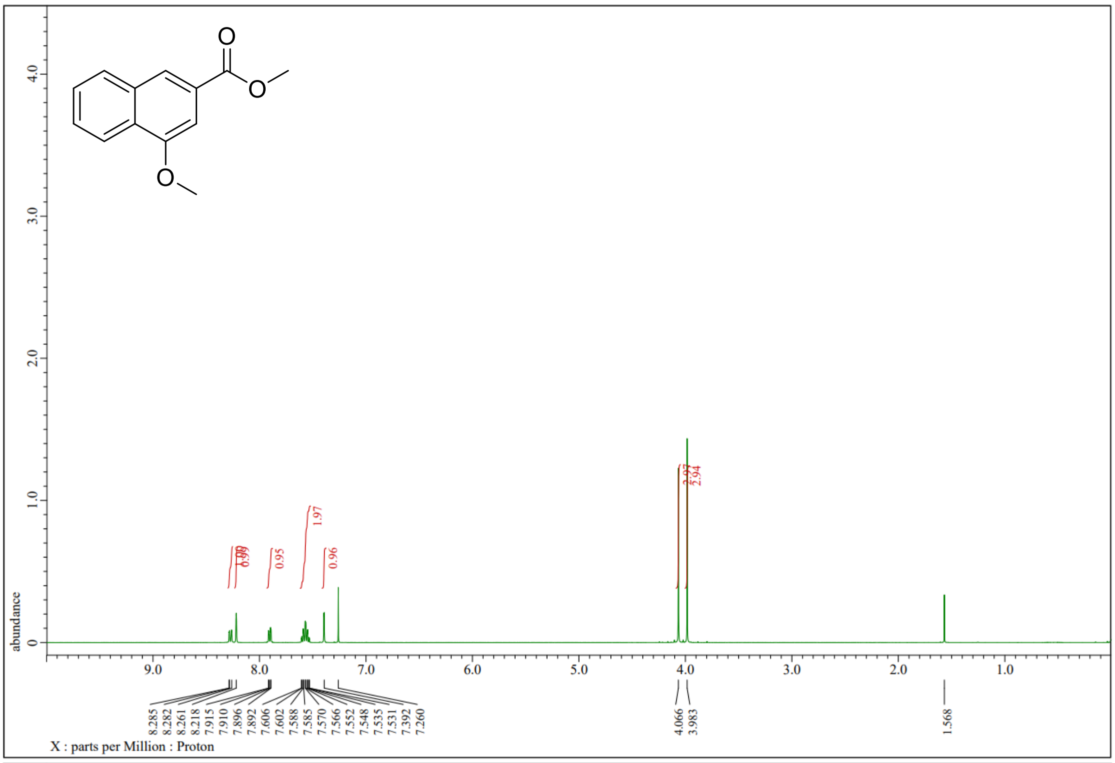

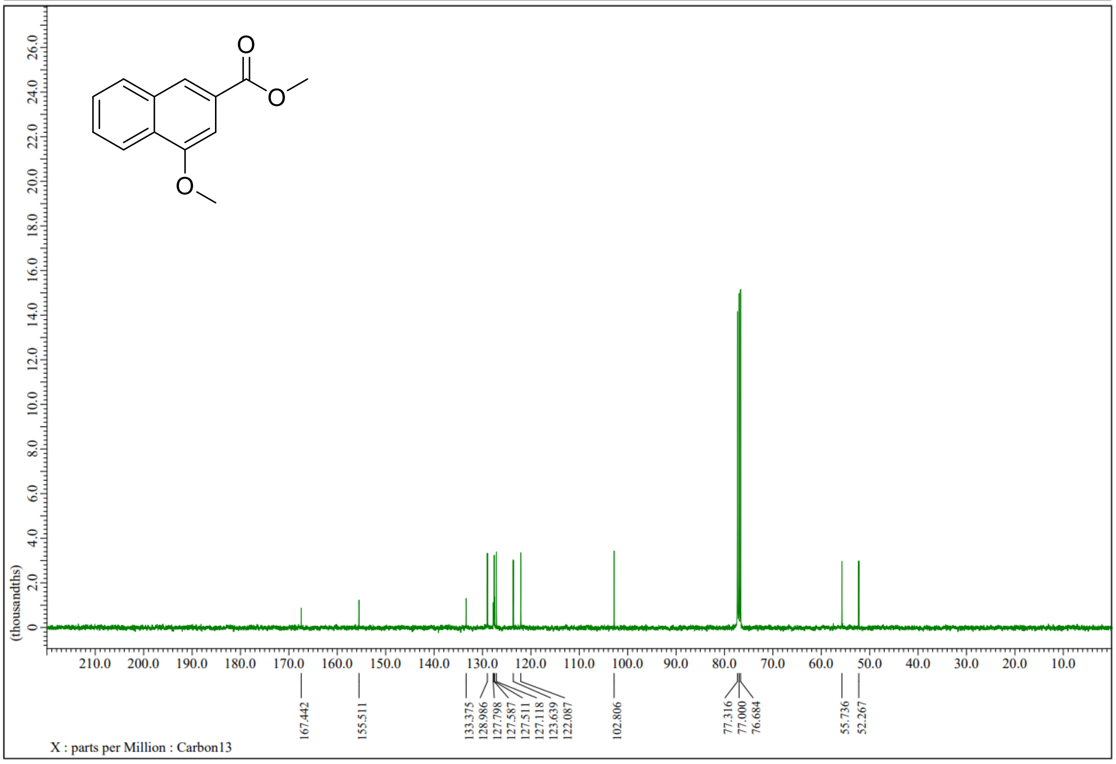

Figure S3. Methyl 4-methoxy-2-naphthoate **(S1c)**

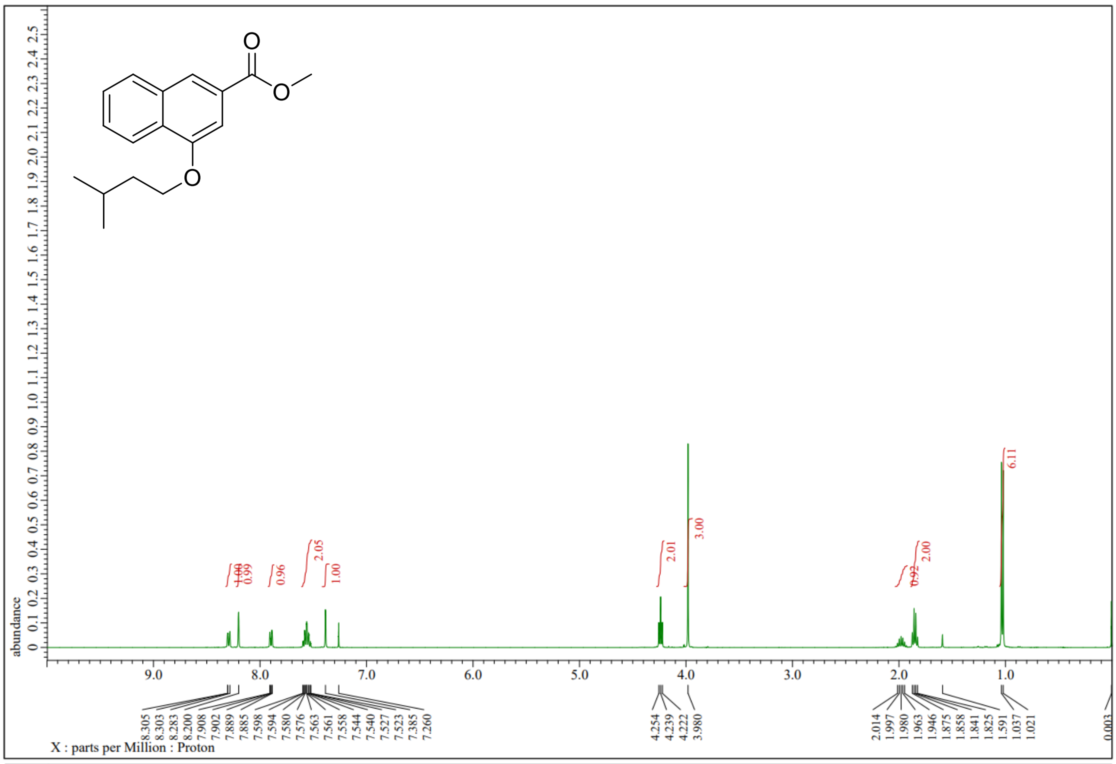

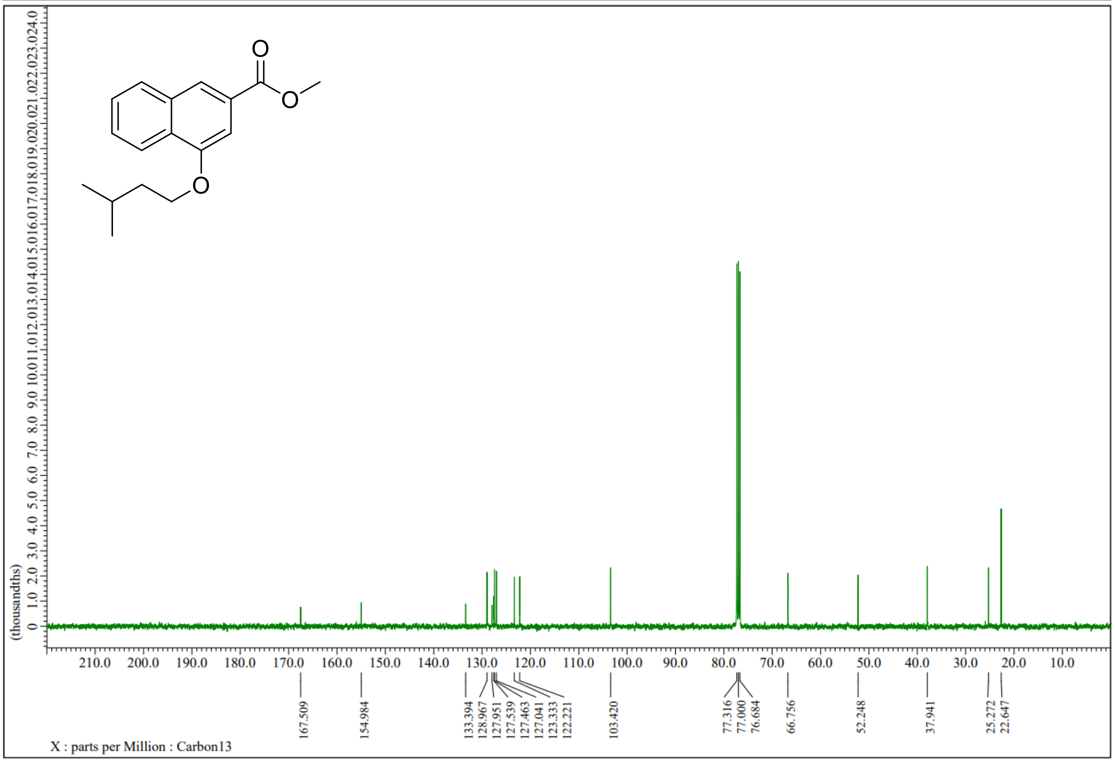

Figure S4. Methyl 4-(isoamyloxy)-2-naphthoate **(S1d)**

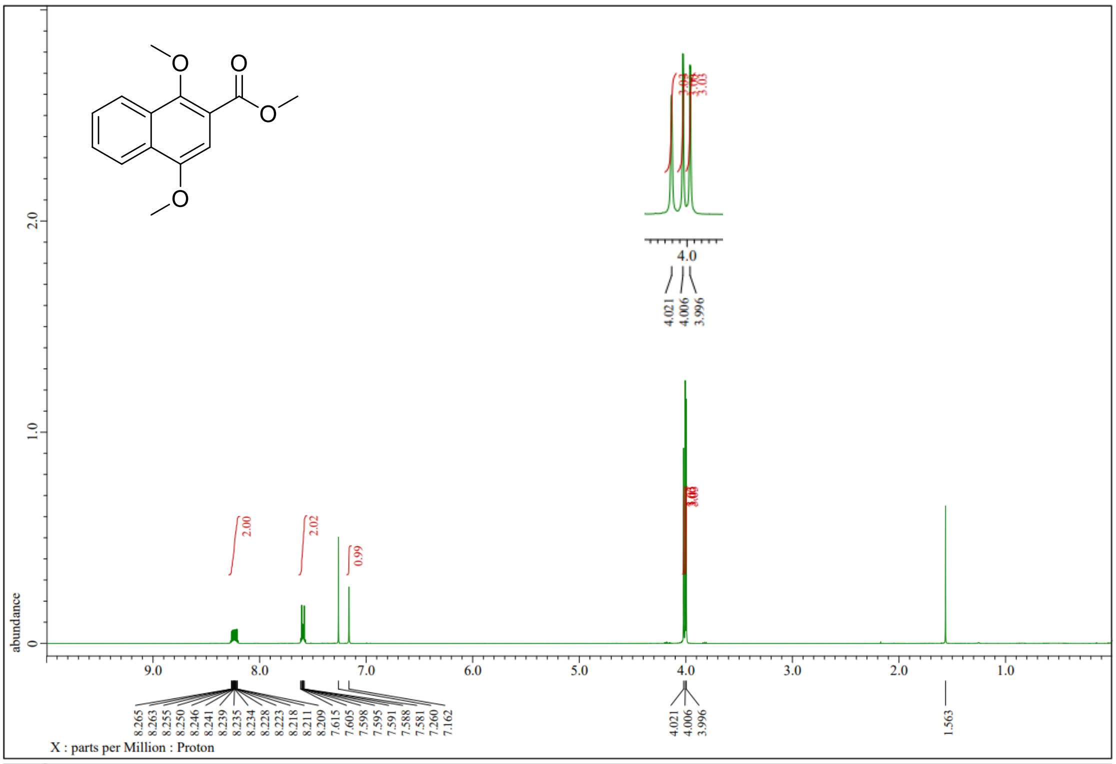

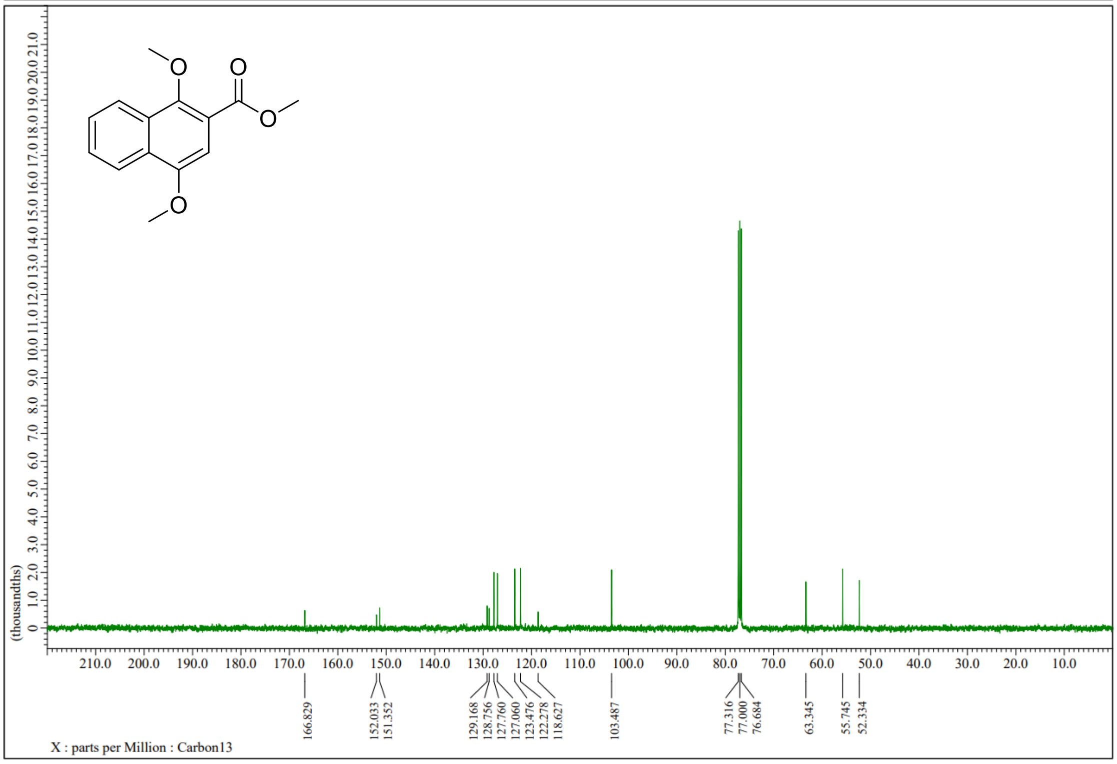

Figure S5. Methyl 1,4-dimethoxy-2-naphthoate **(S1e)**

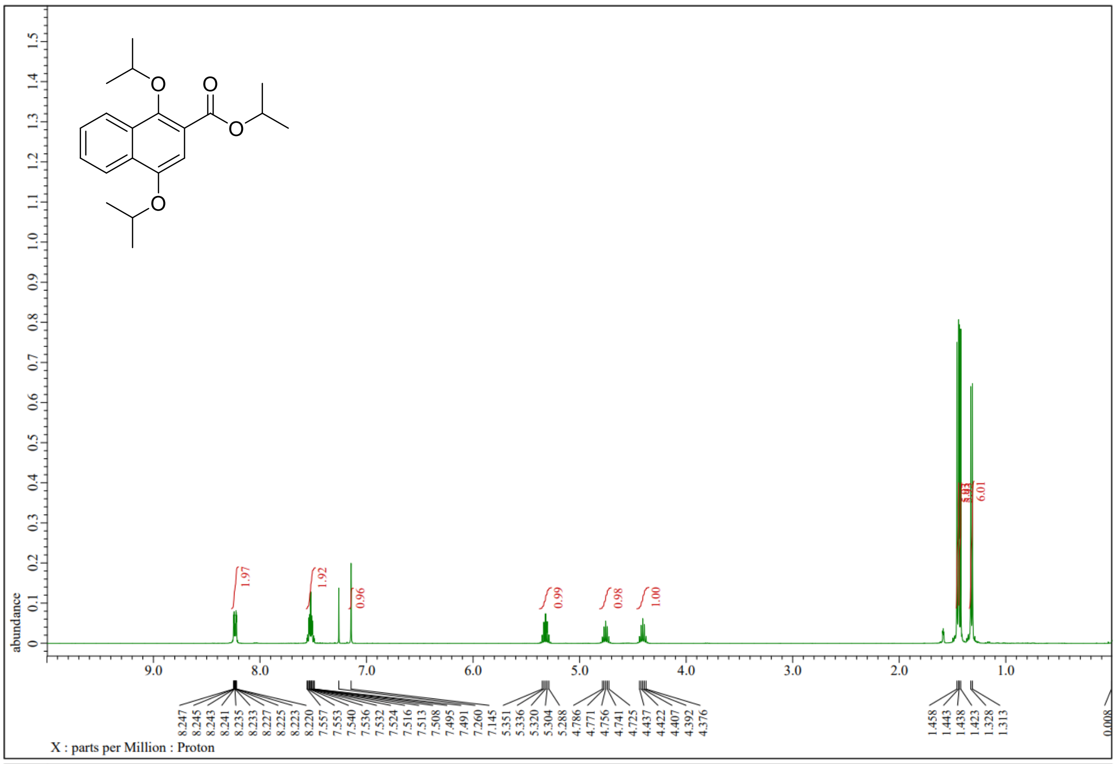

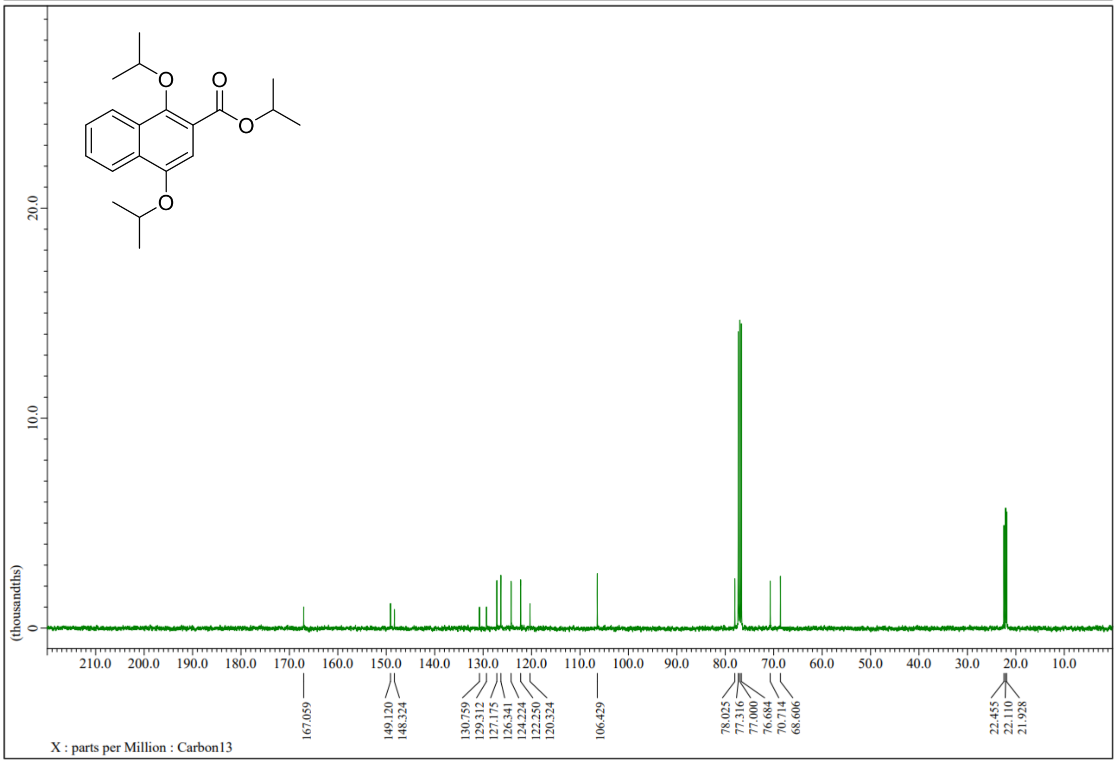

Figure S6. Isopropyl 1,4-diisopropoxy-2-naphthoate **(S1f)**

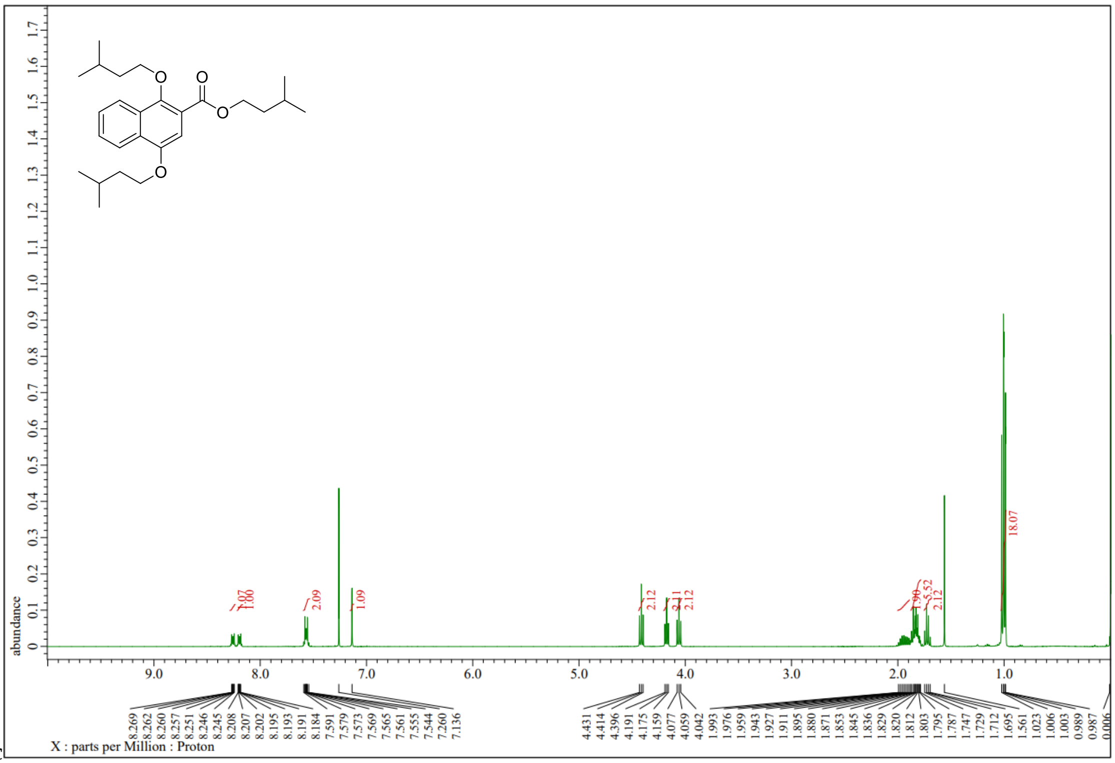

c

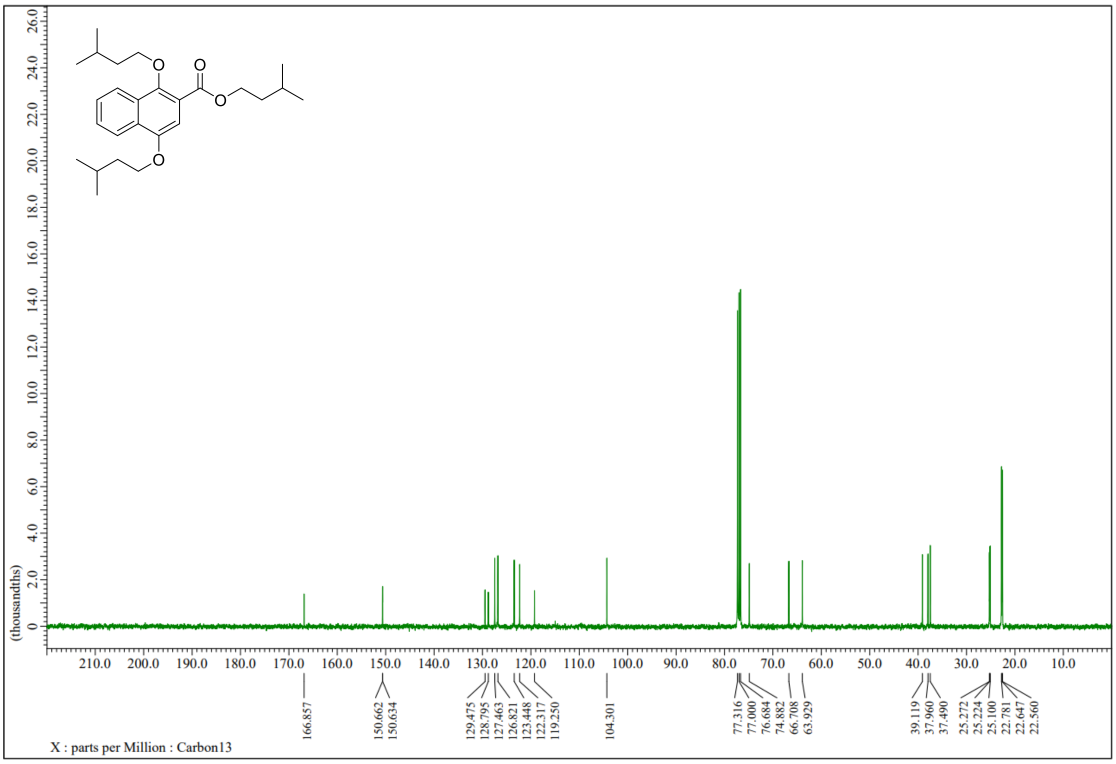

Figure S7. Isoamyl 1,4-bis(isoamyloxy)-2-naphthoate **(S1g)**

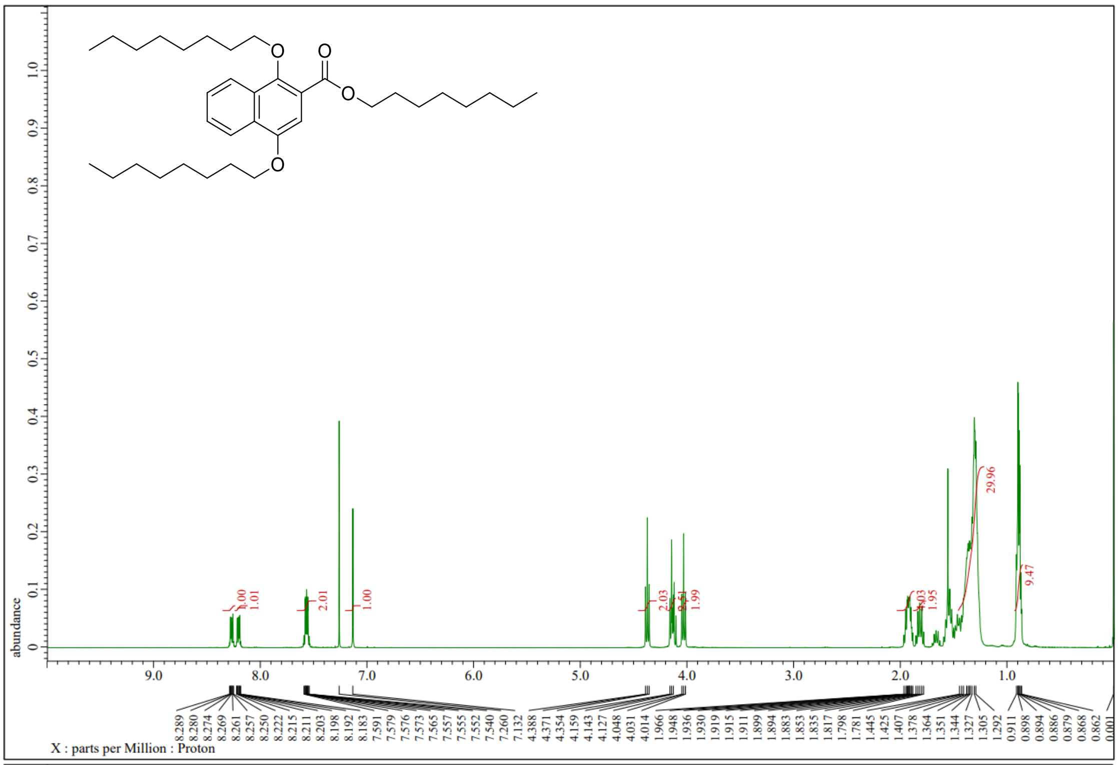

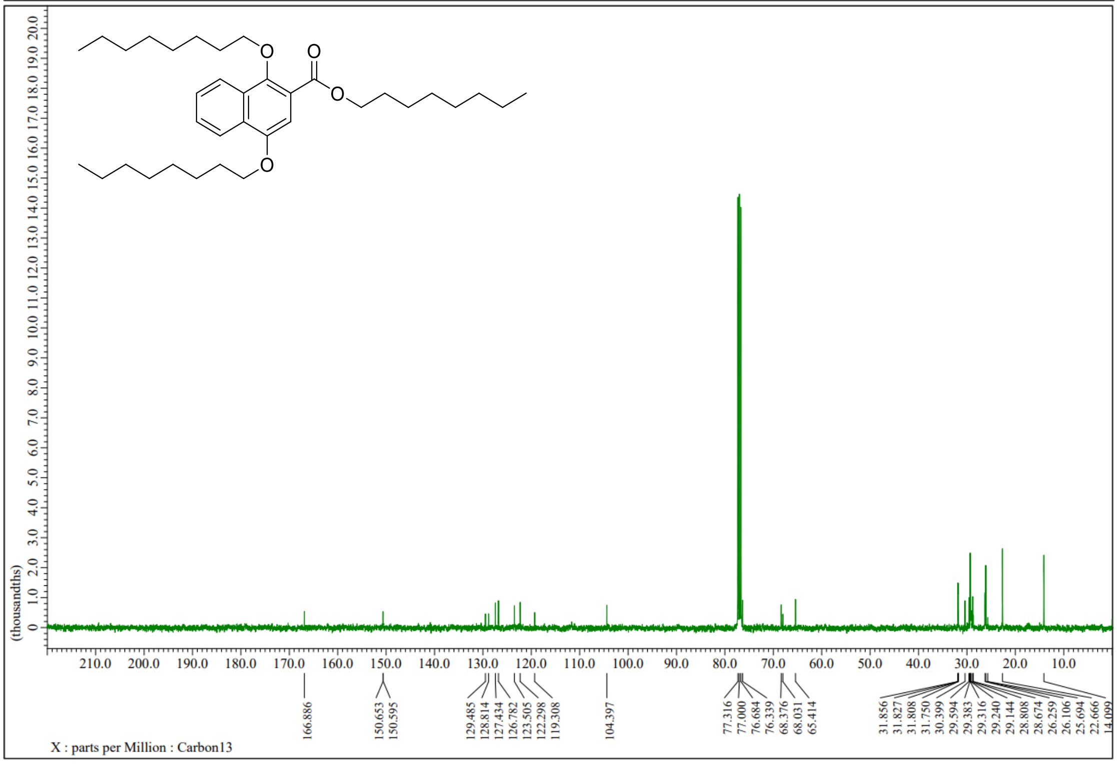

Figure S8. Octyl 1,4-bis(octyloxy)-2-naphthoate **(S1h)**

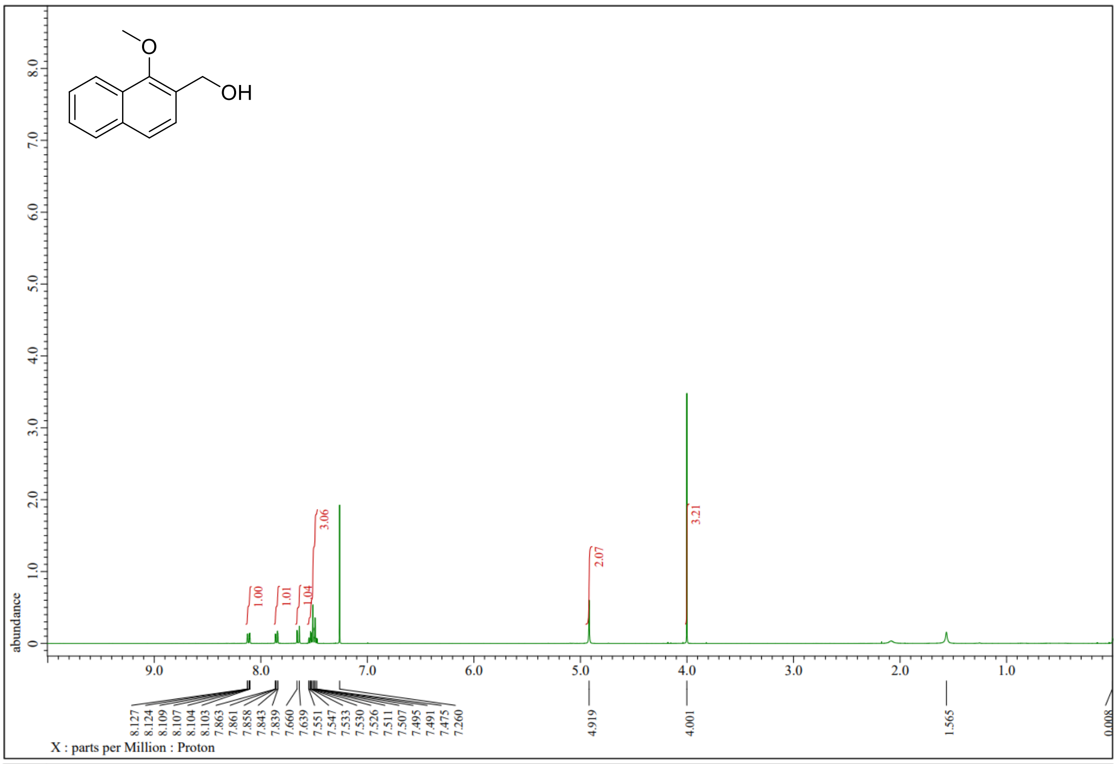

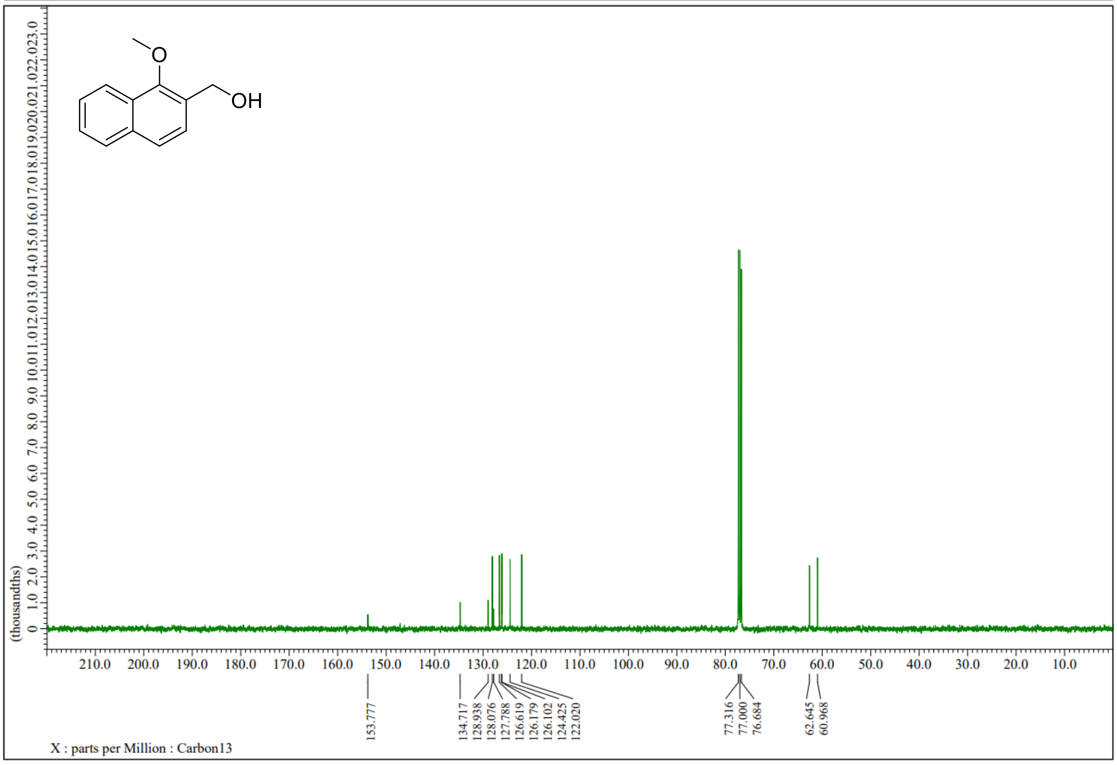

Figure S9. (1-Methoxynaphthalen-2-yl)methanol **(5a)**

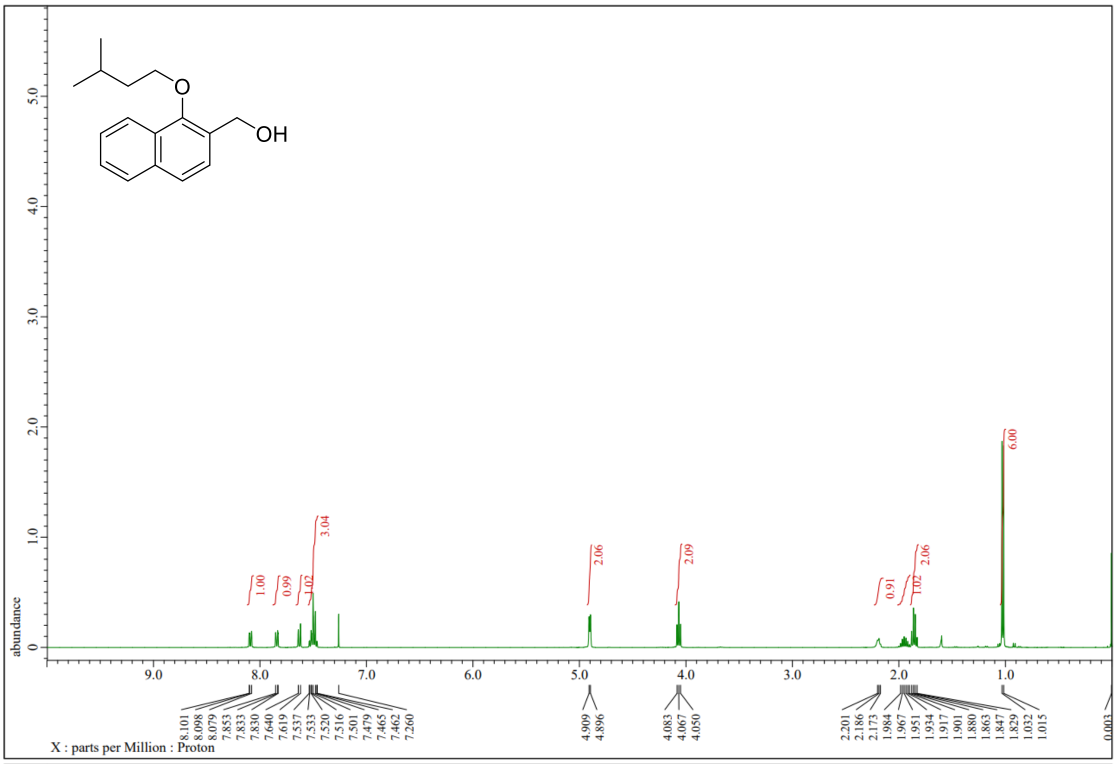

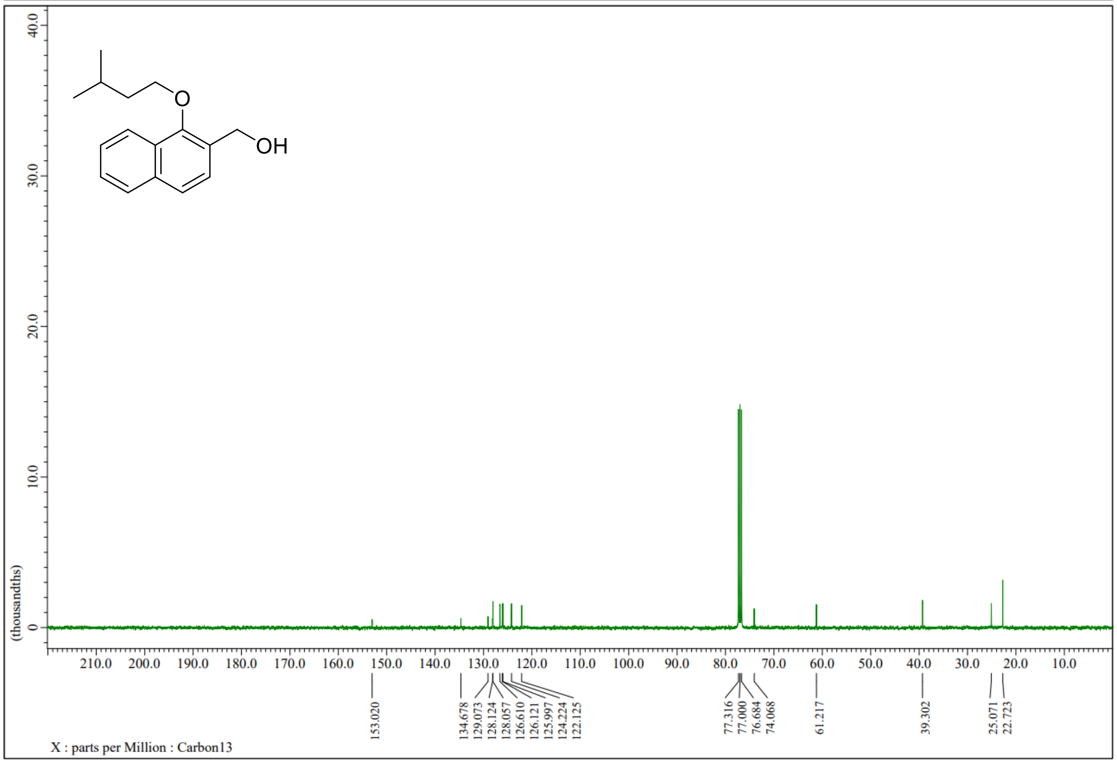

Figure S10. (1-(Isoamyloxy)naphthalen-2-yl)methanol **(5b)**

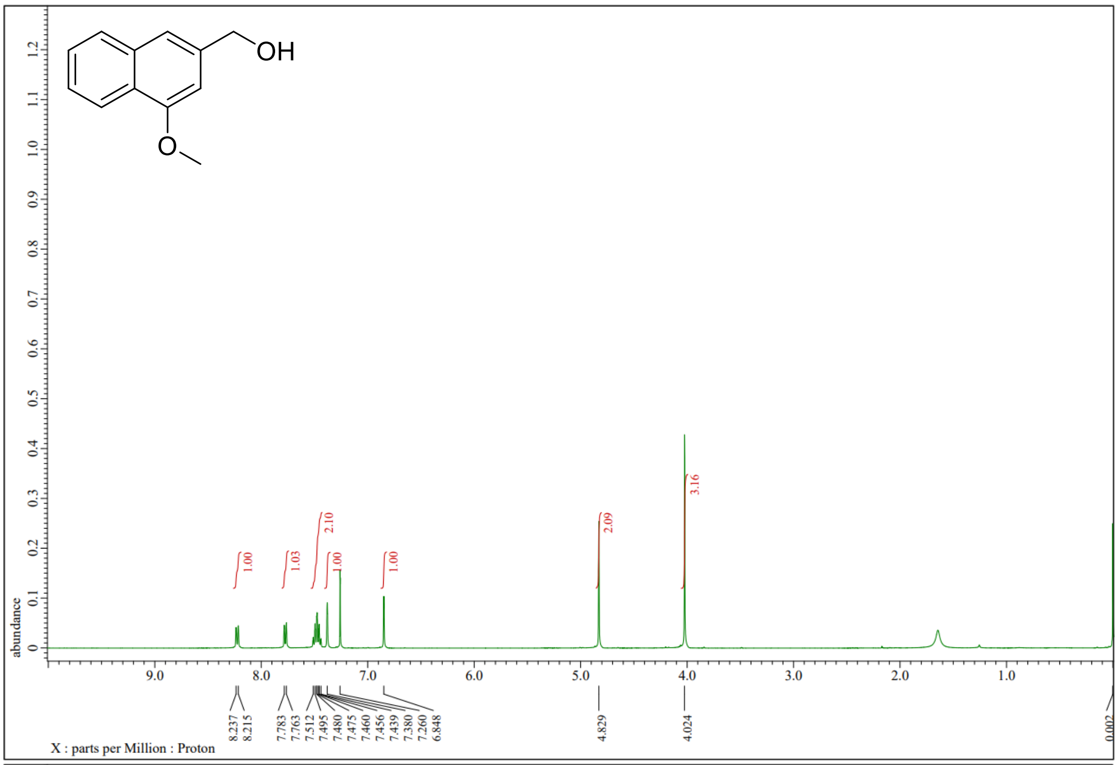

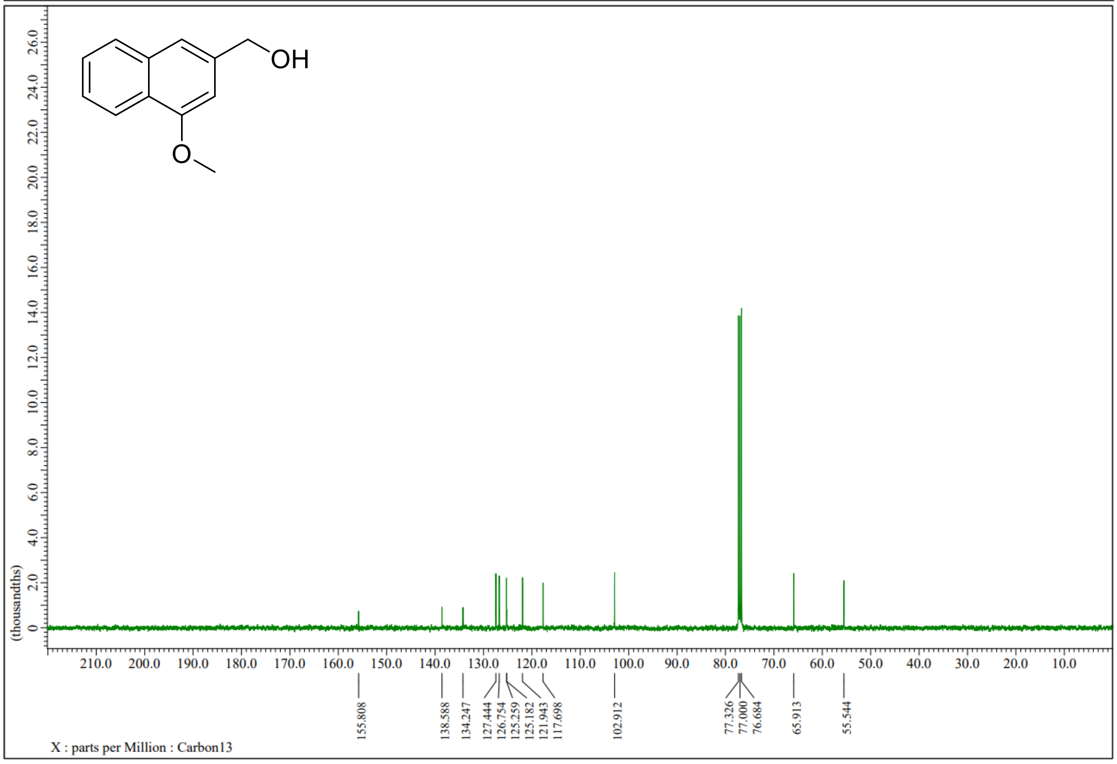

Figure S11. (4-Methoxynaphthalen-2-yl)methanol **(5c)**

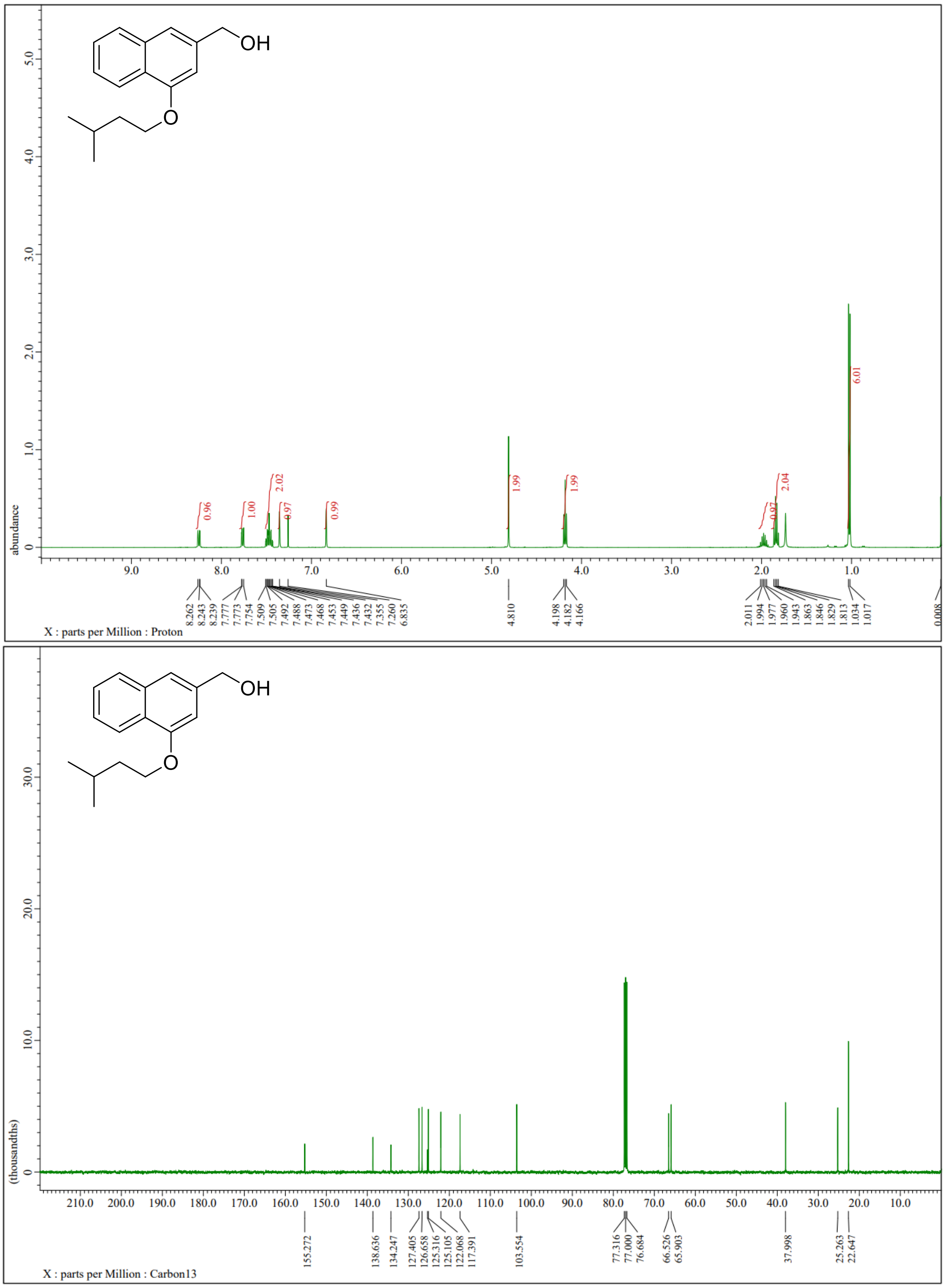

Figure S12. (4-(Isoamyloxy)naphthalen-2-yl)methanol **(5d)**

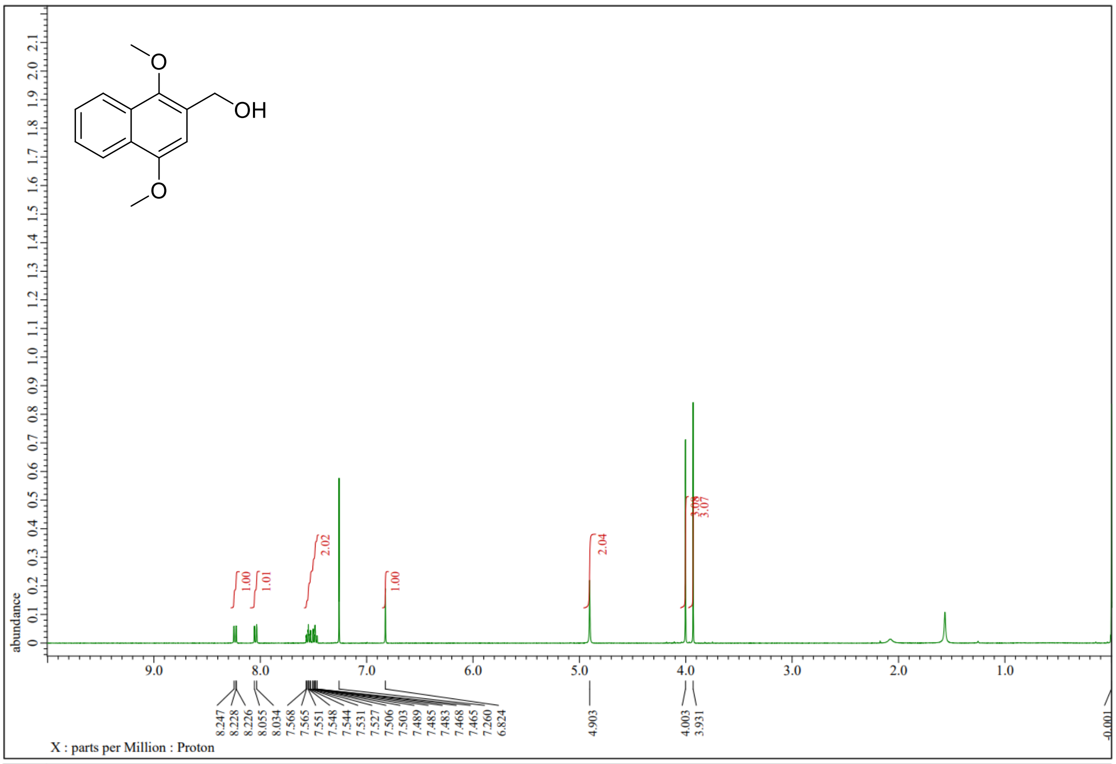

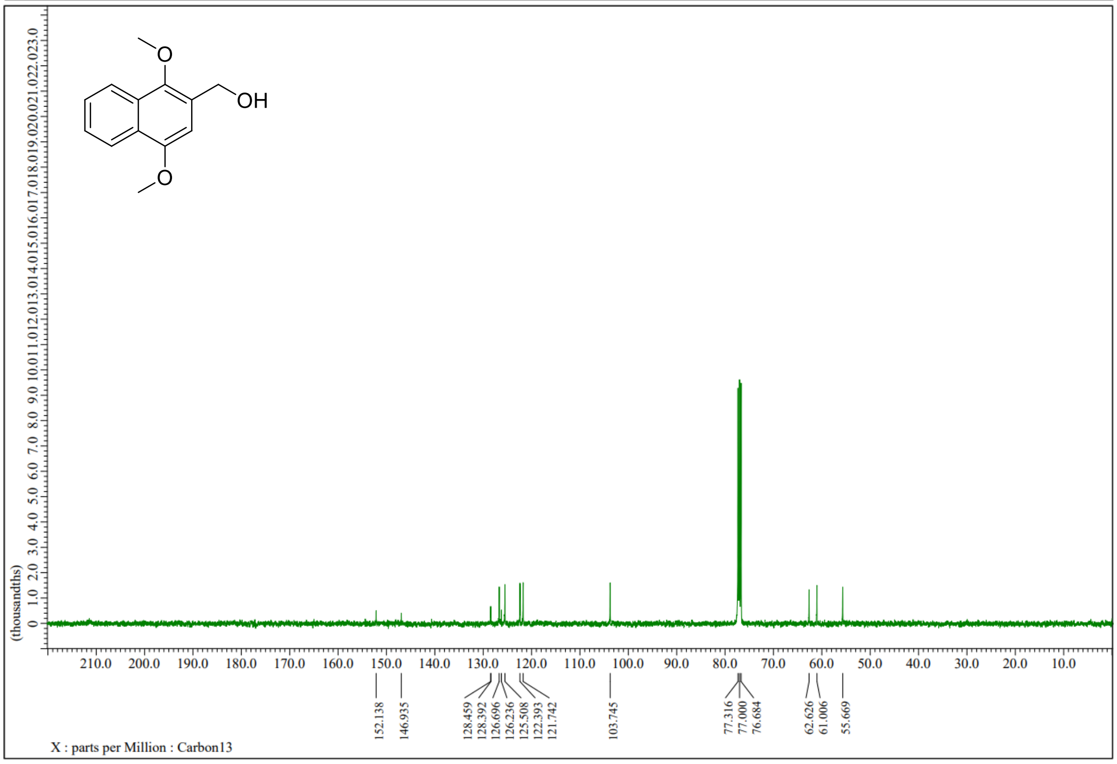

Figure S13. (1,4-Dimethoxynaphthalen-2-yl)methanol **(5e)**

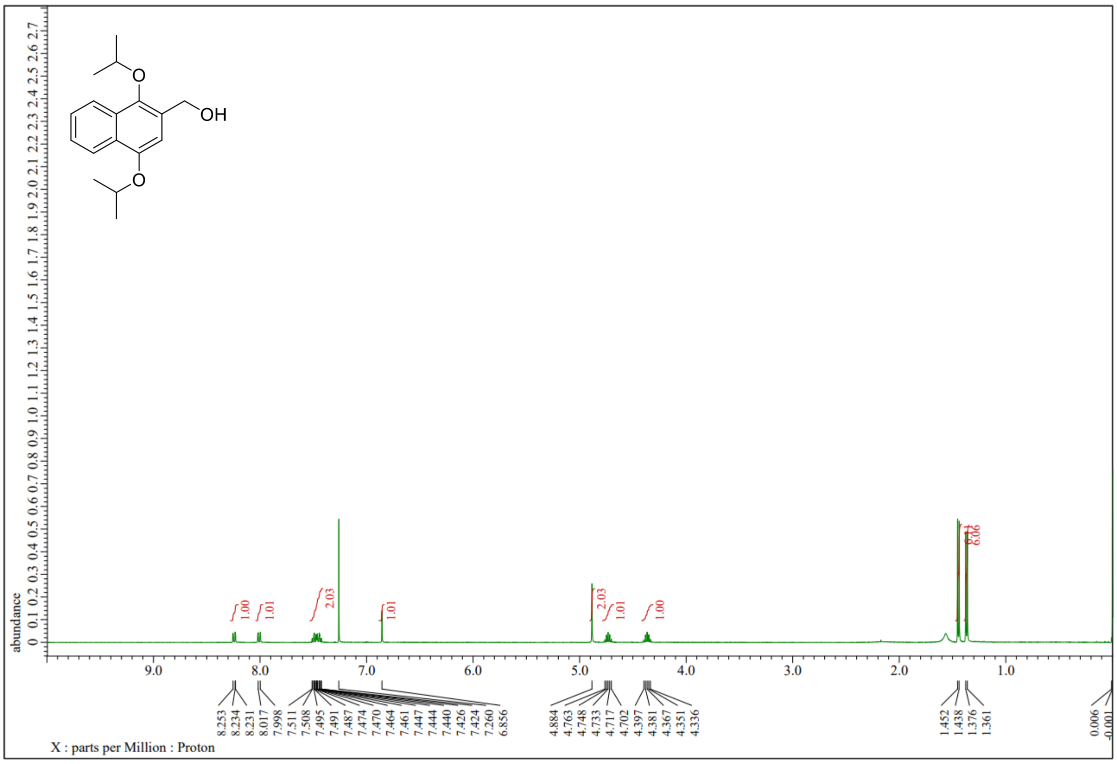

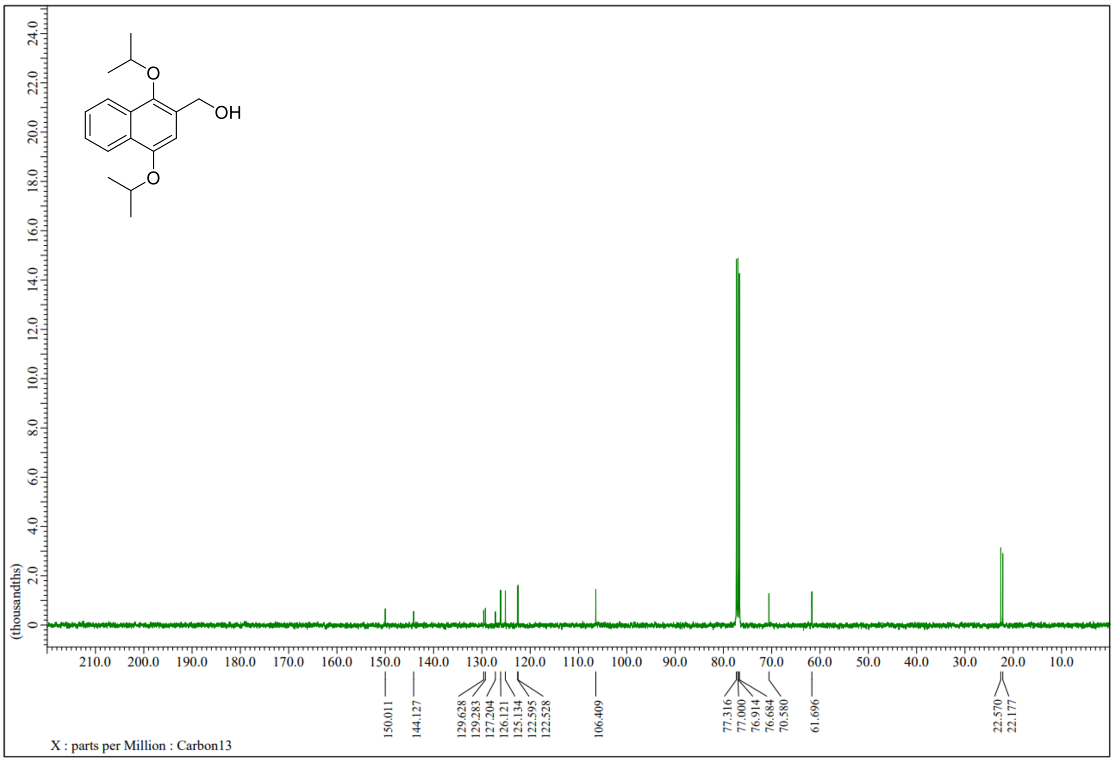

Figure S14. (1,4-Diisopropoxynaphthalen-2-yl)methanol **(5f)**

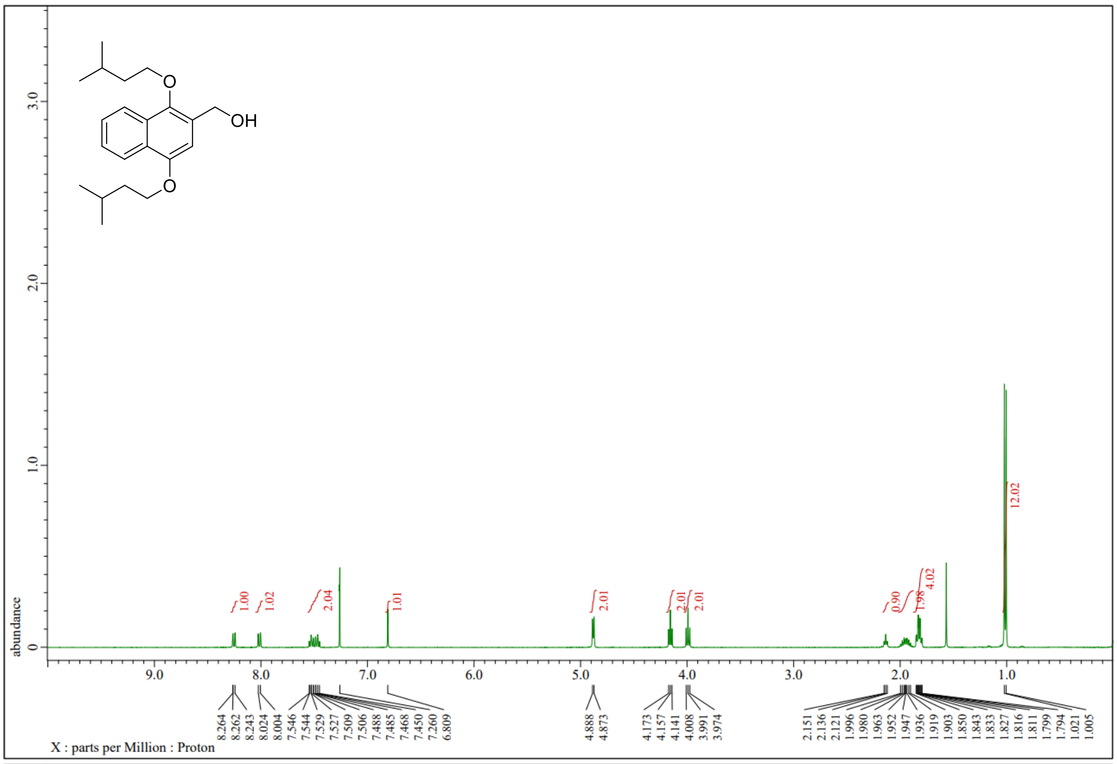

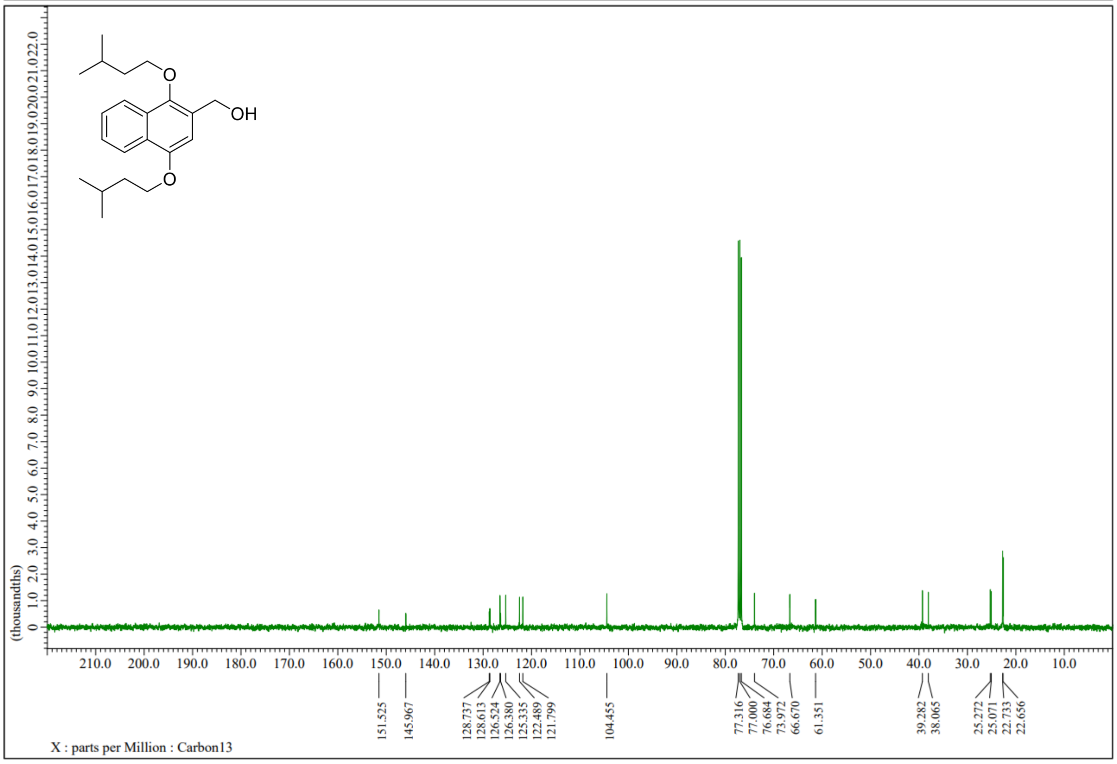

Figure S15. (1,4-Bis(isoamyloxy)naphthalen-2-yl)methanol **(5g)**

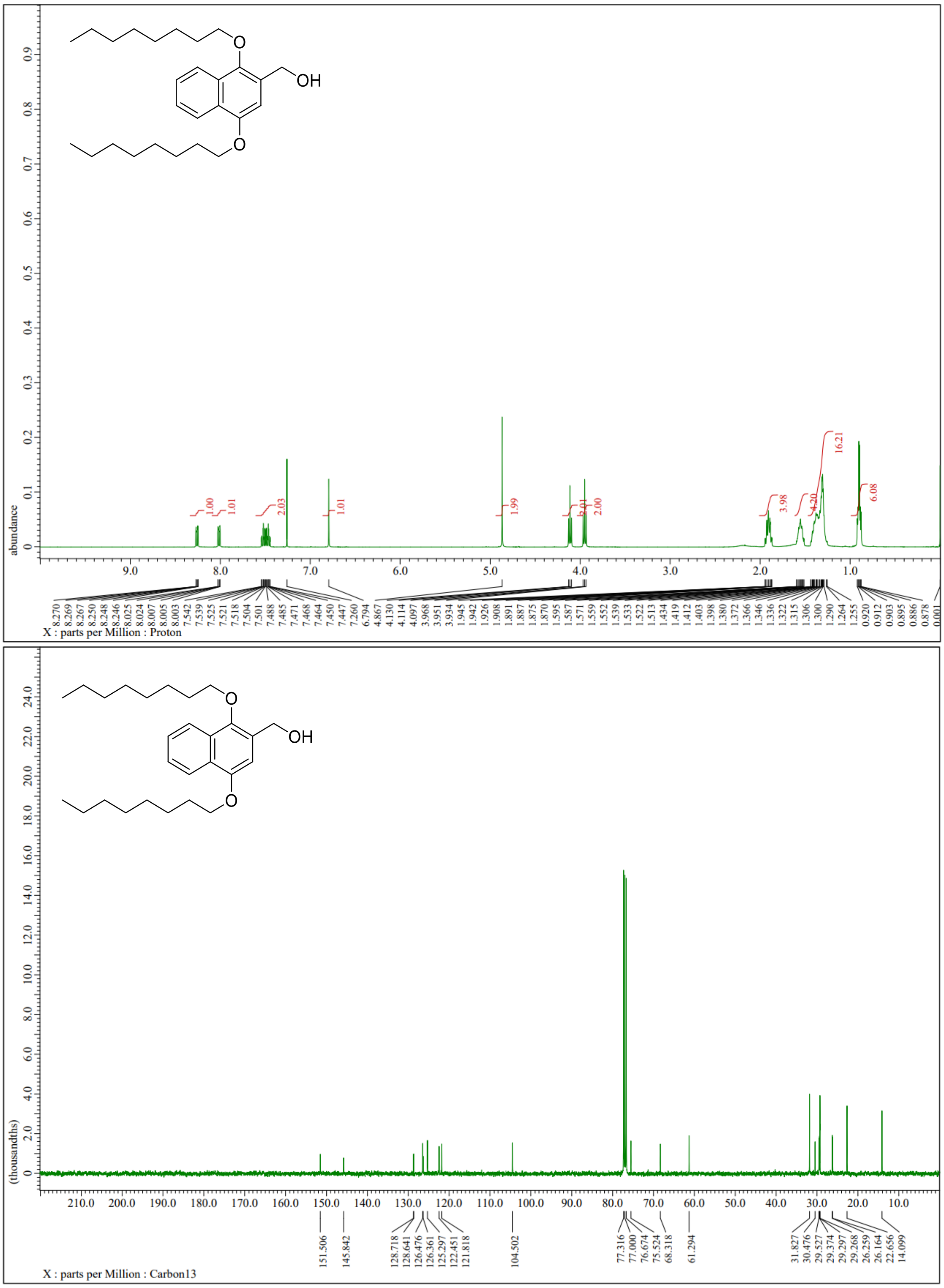

Figure S16. (1,4-Bis(octyloxy)naphthalen-2-yl)methanol **(5h)**

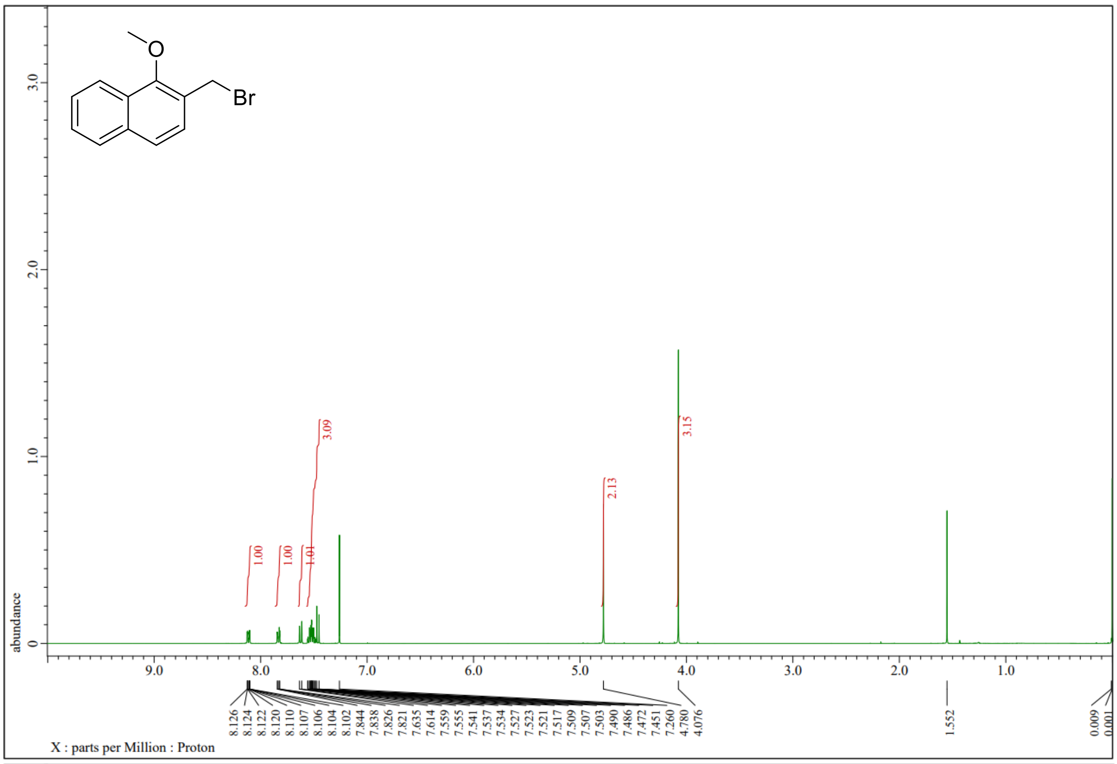

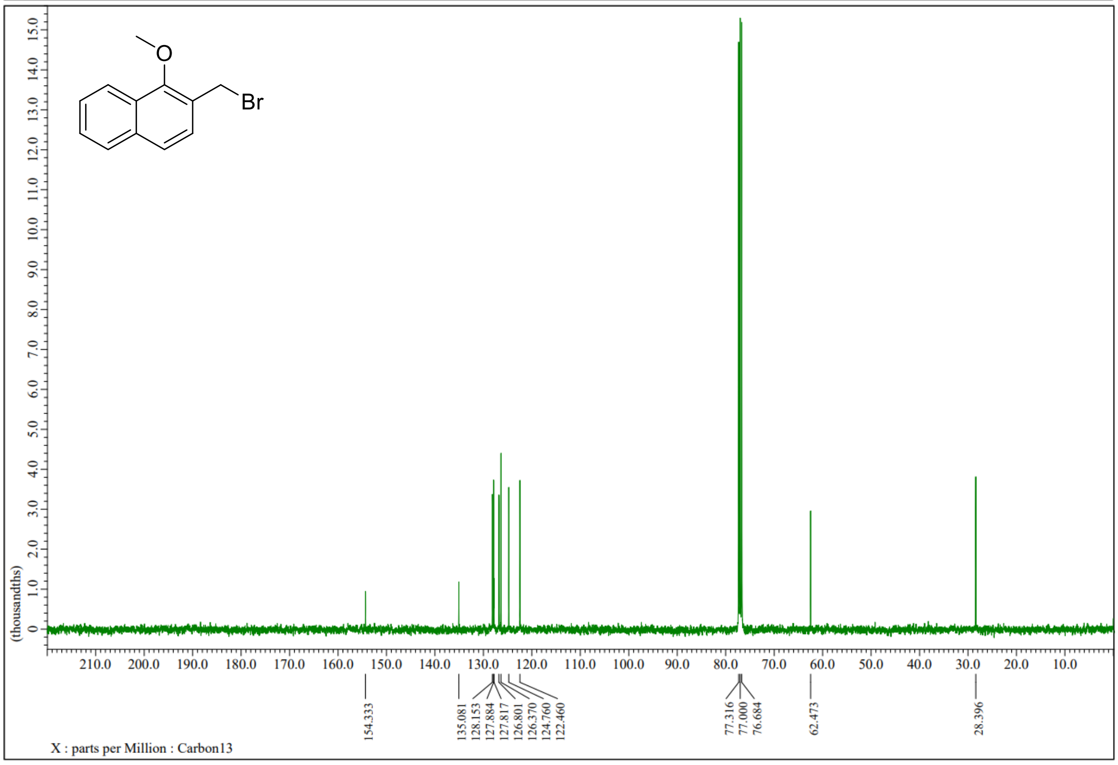

Figure S17. 2-(Bromomethyl)-1-methoxynaphthalene **(12a)**

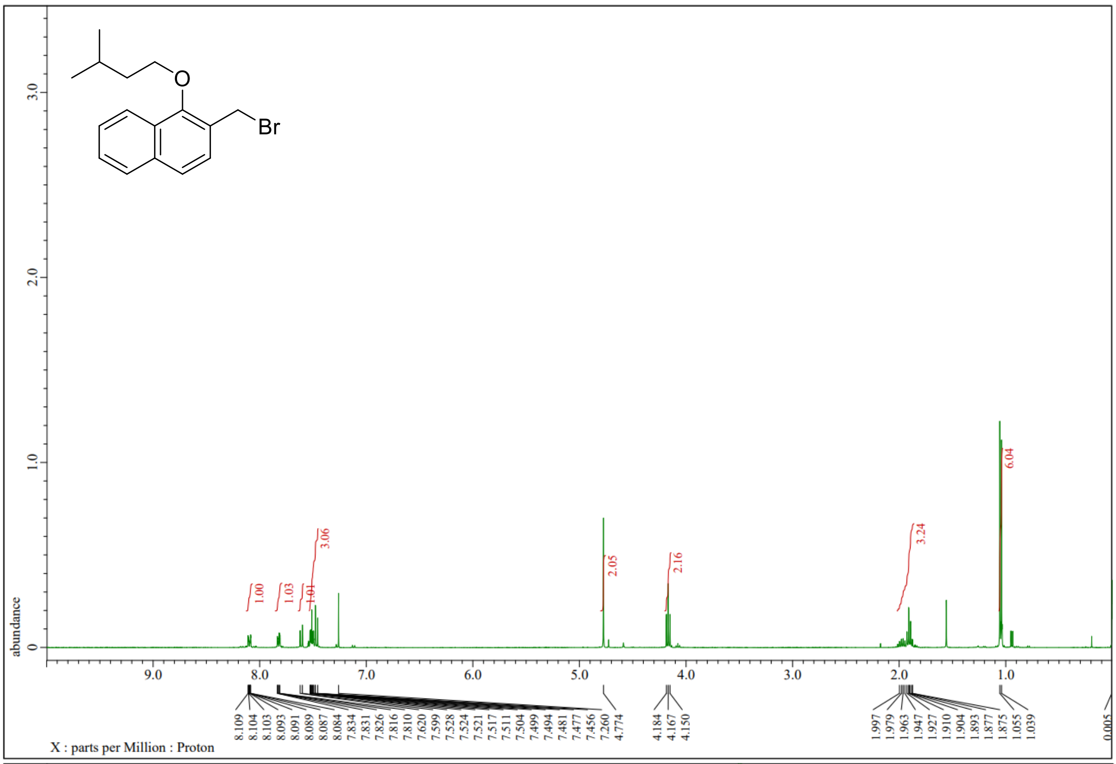

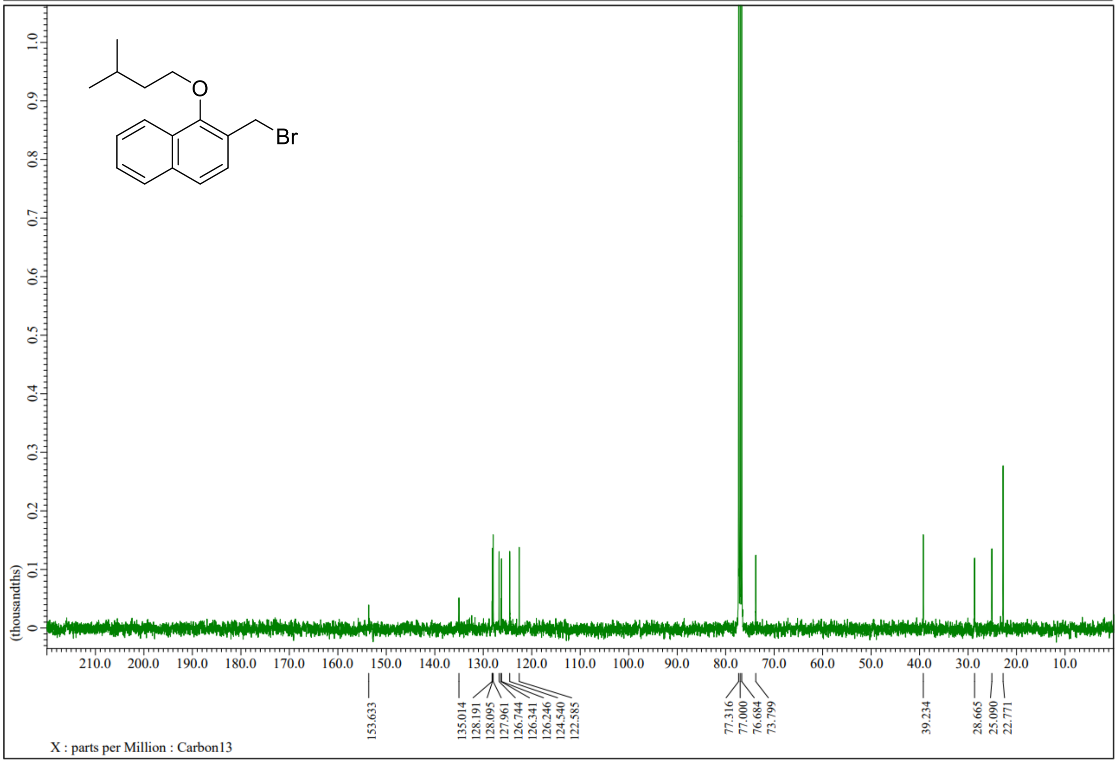

Figure S18. 2-(Bromomethyl)-1-(isoamyloxy)naphthalene **(12b)**

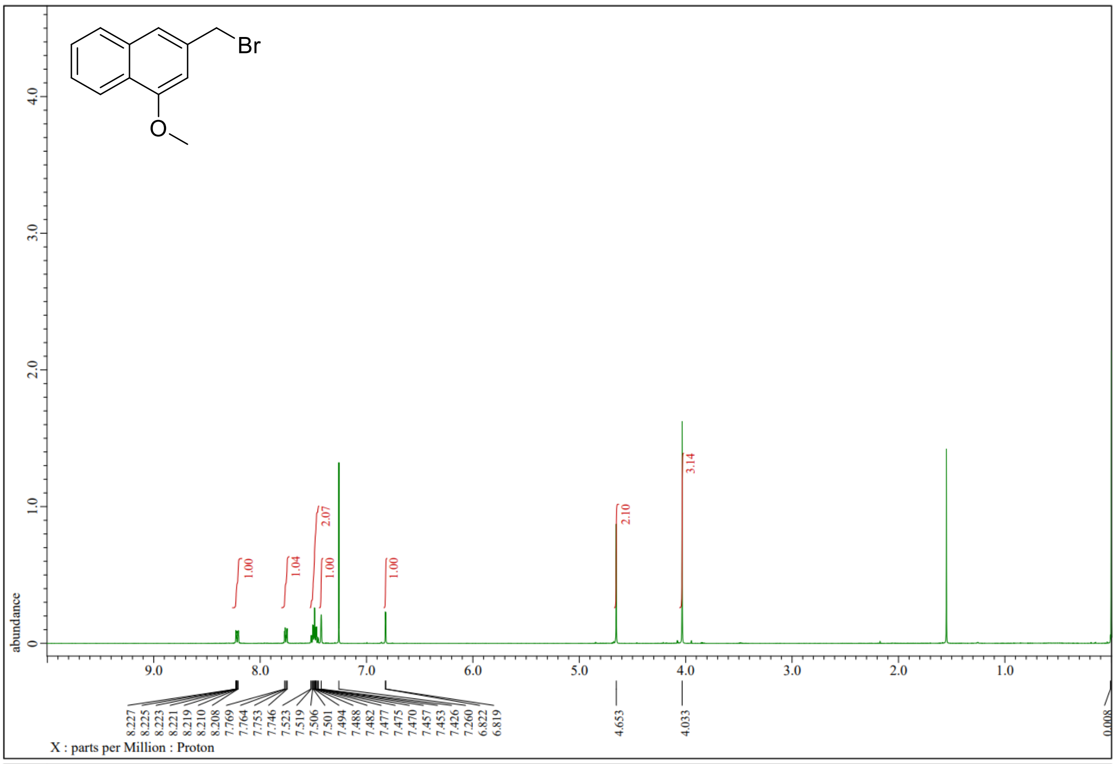

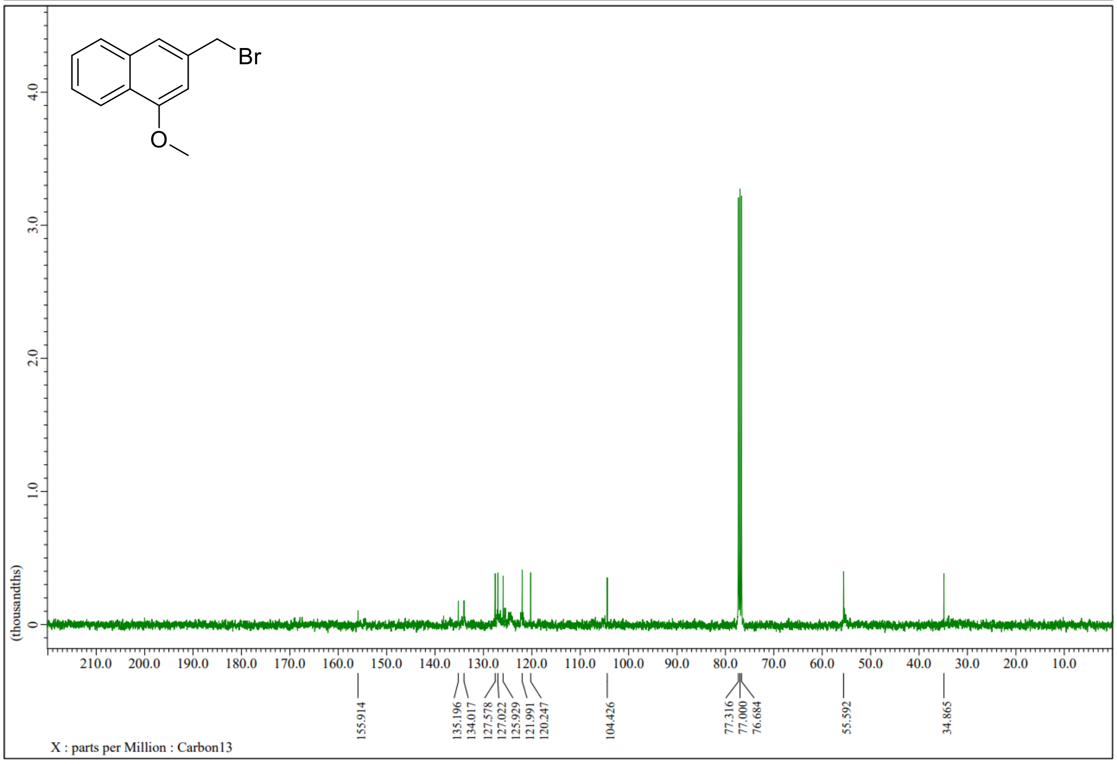

Figure S19. 3-(Bromomethyl)-1-methoxynaphthalene **(12c)**

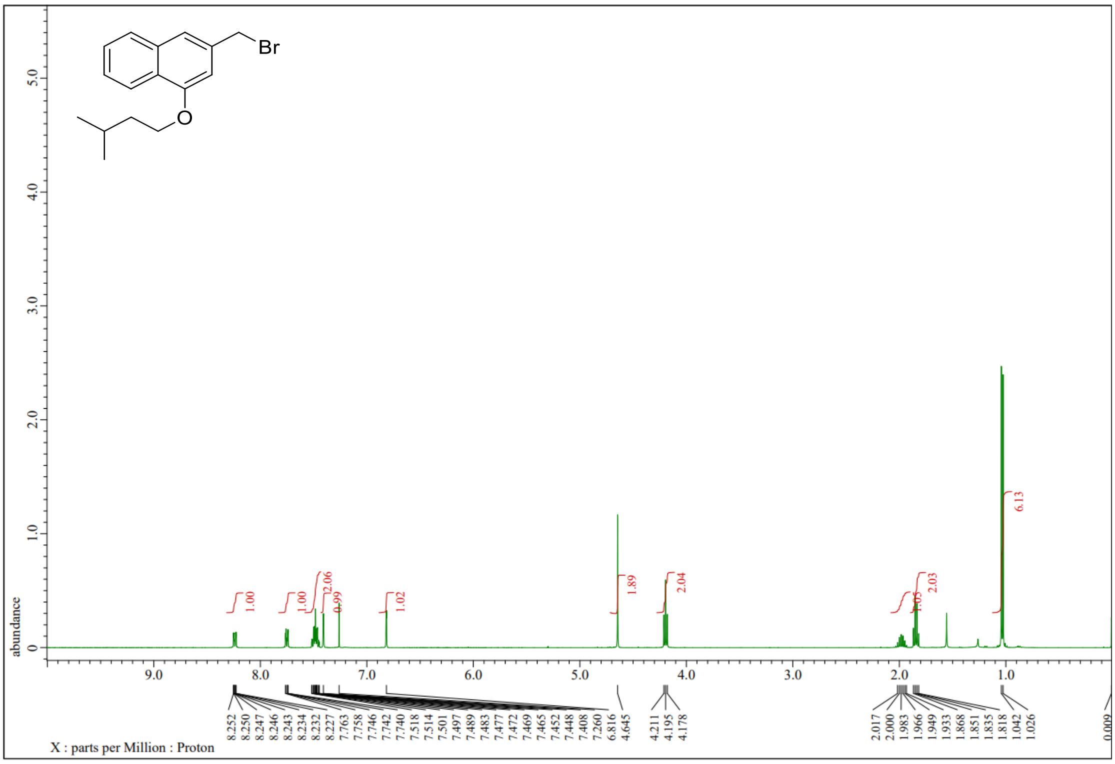

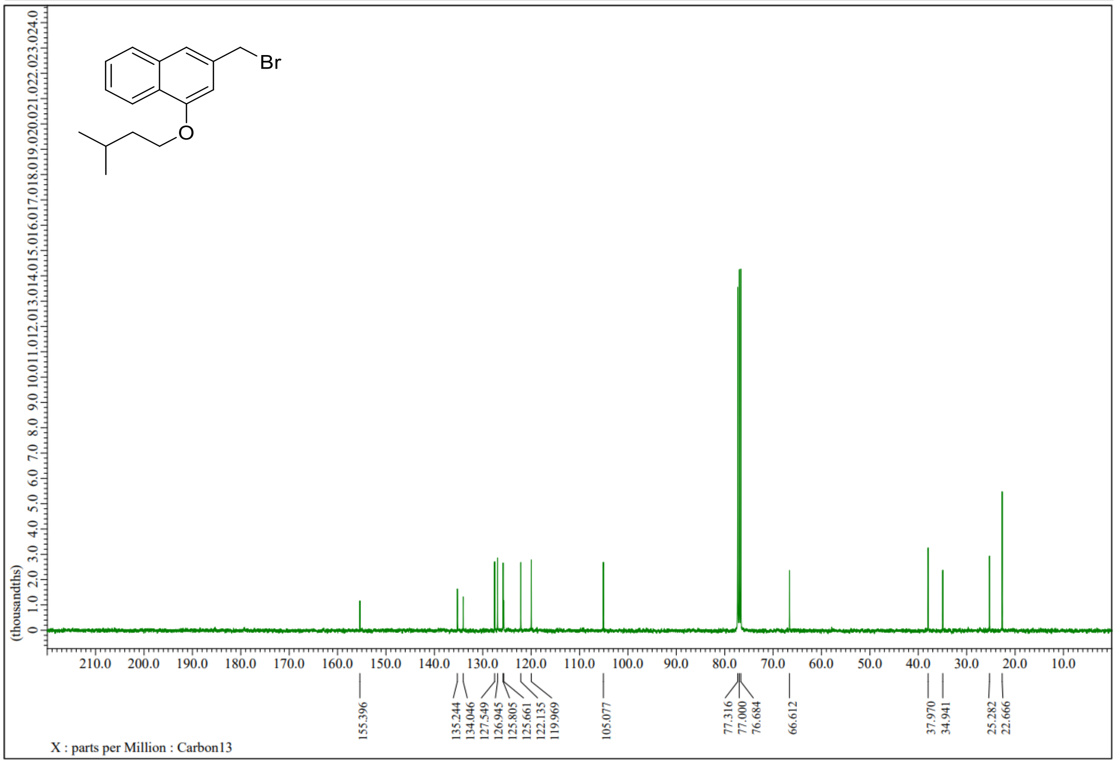

Figure S20. 3-(Bromomethyl)-1-(isoamyloxy)naphthalene **(12d)**

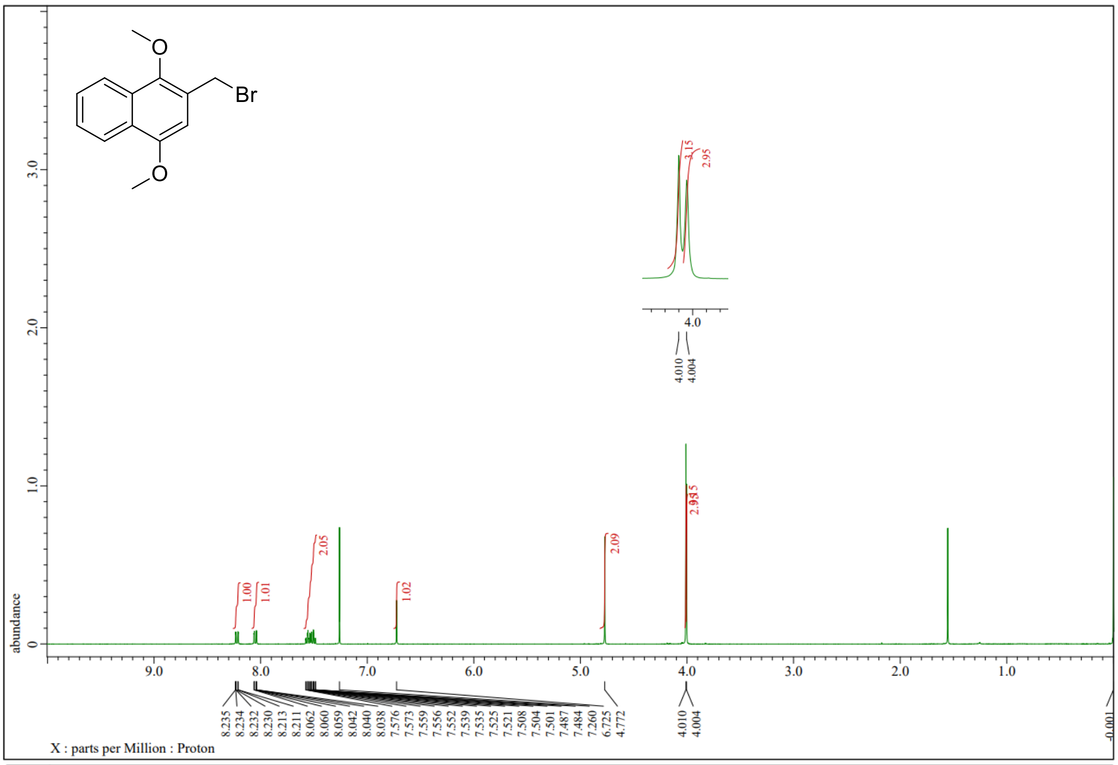

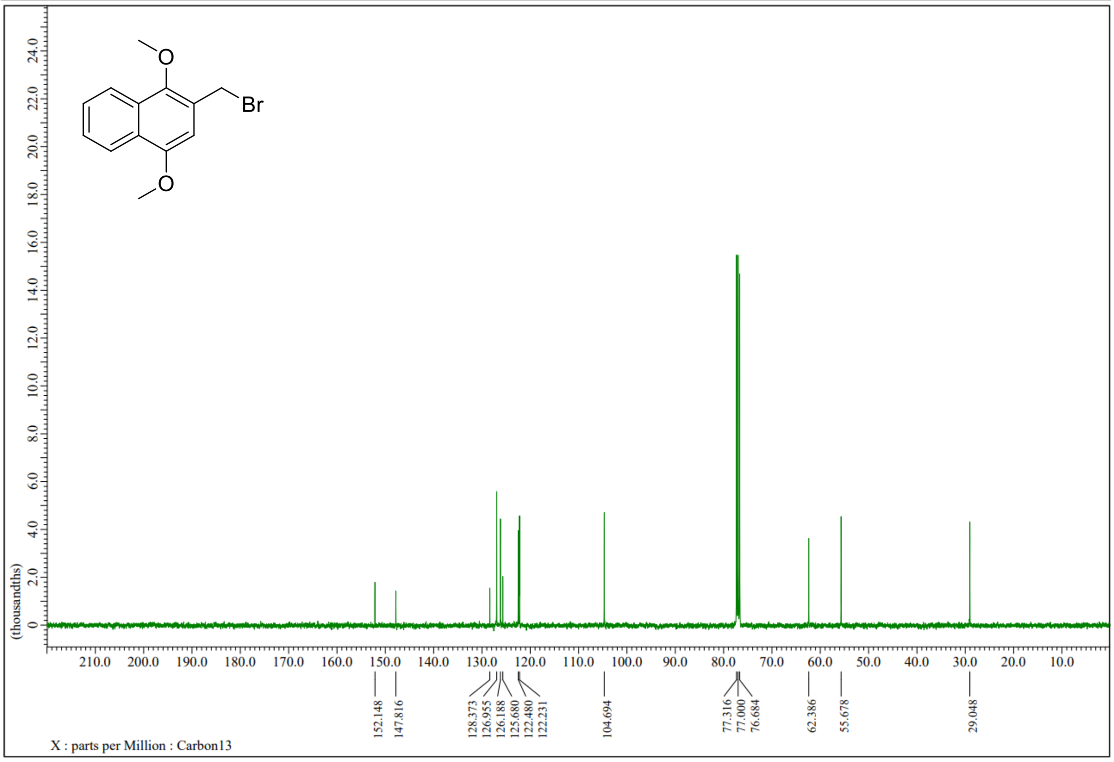

Figure S21. 2-(Bromomethyl)-1,4-dimethoxynaphthalene **(12e)**

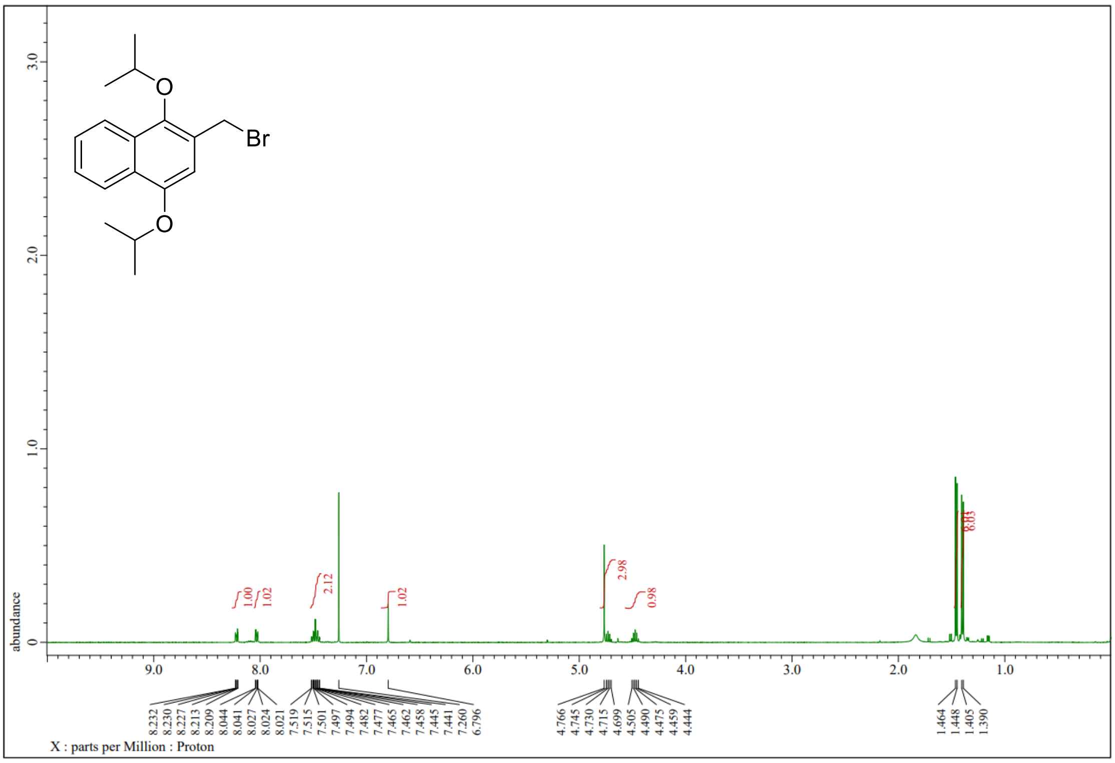

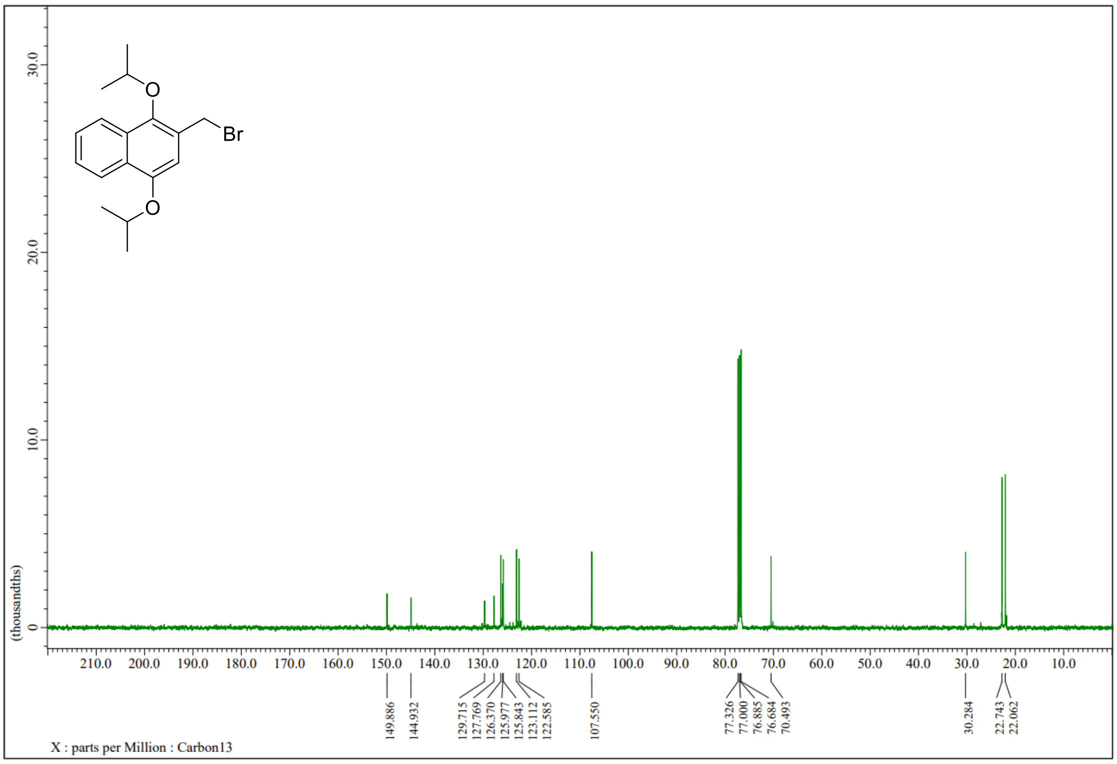

Figure S22. 2-(Bromomethyl)-1,4-diisopropoxynaphthalene **(12f)**

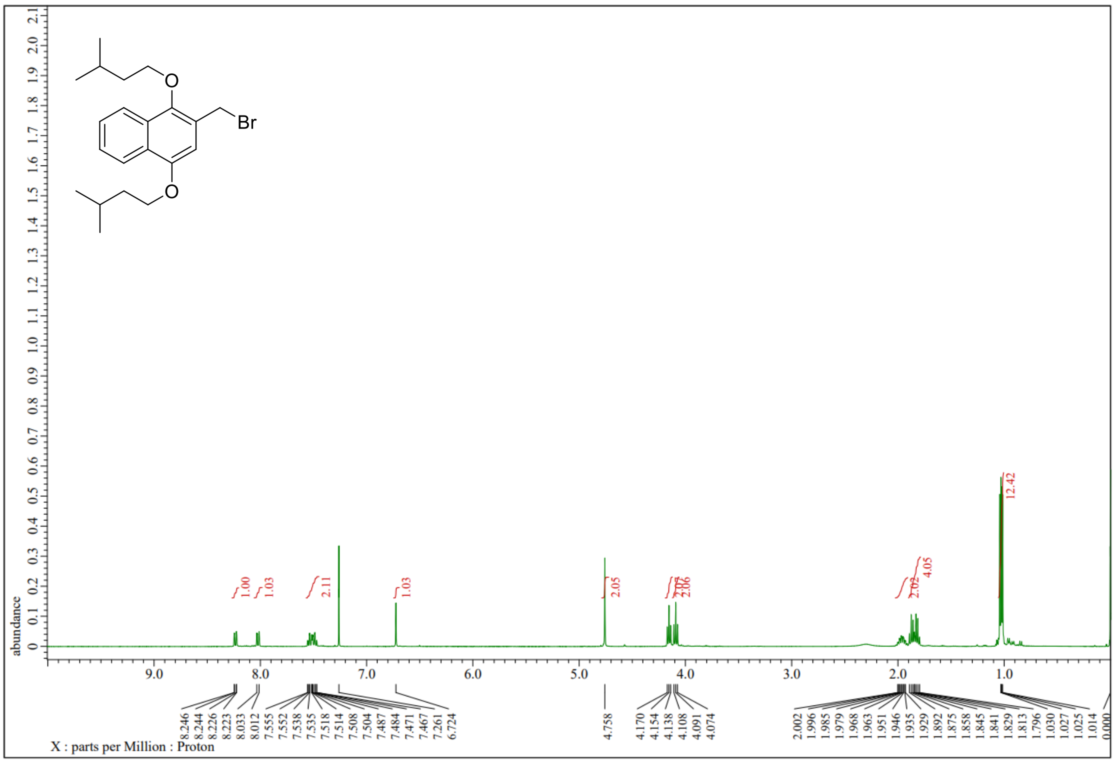

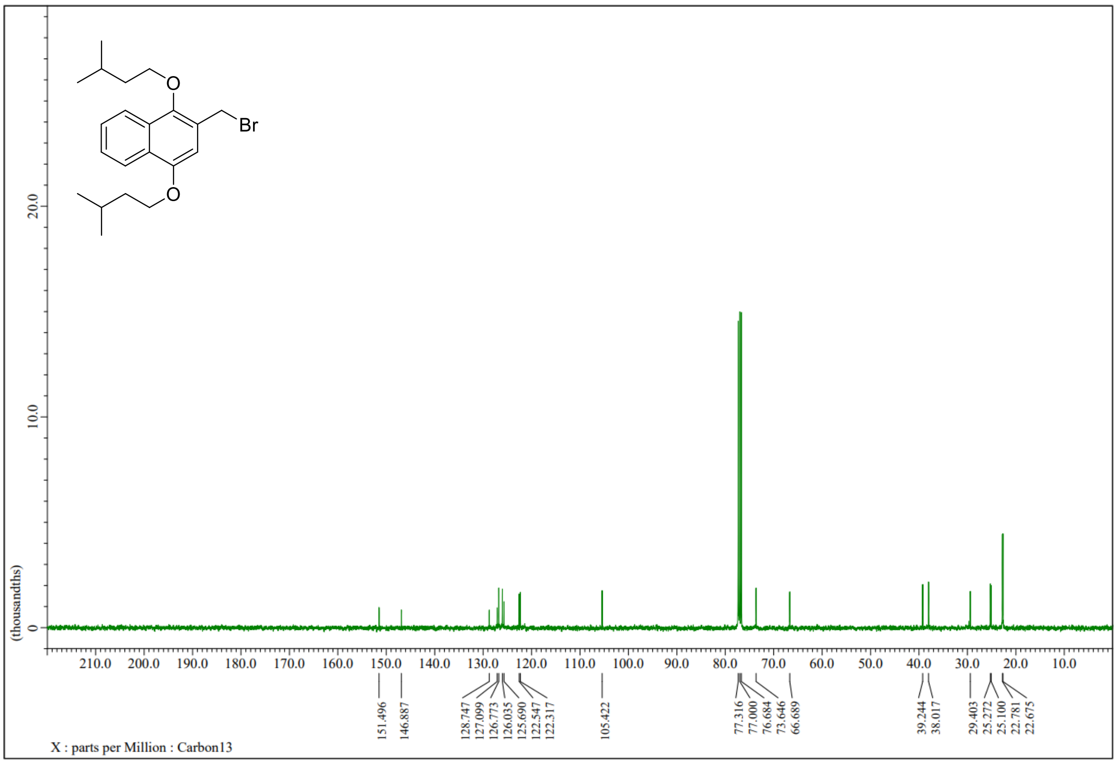

Figure S23. 2-(Bromomethyl)-1,4-bis(isoamyloxy)naphthalene **(12g)**

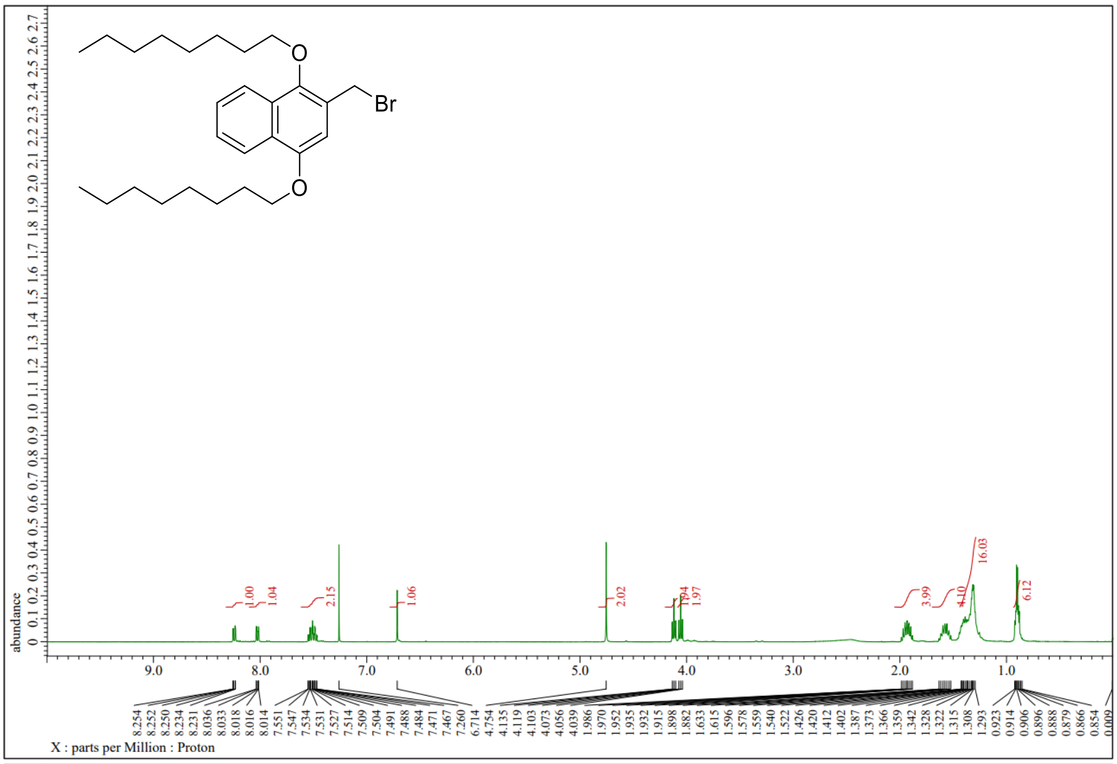

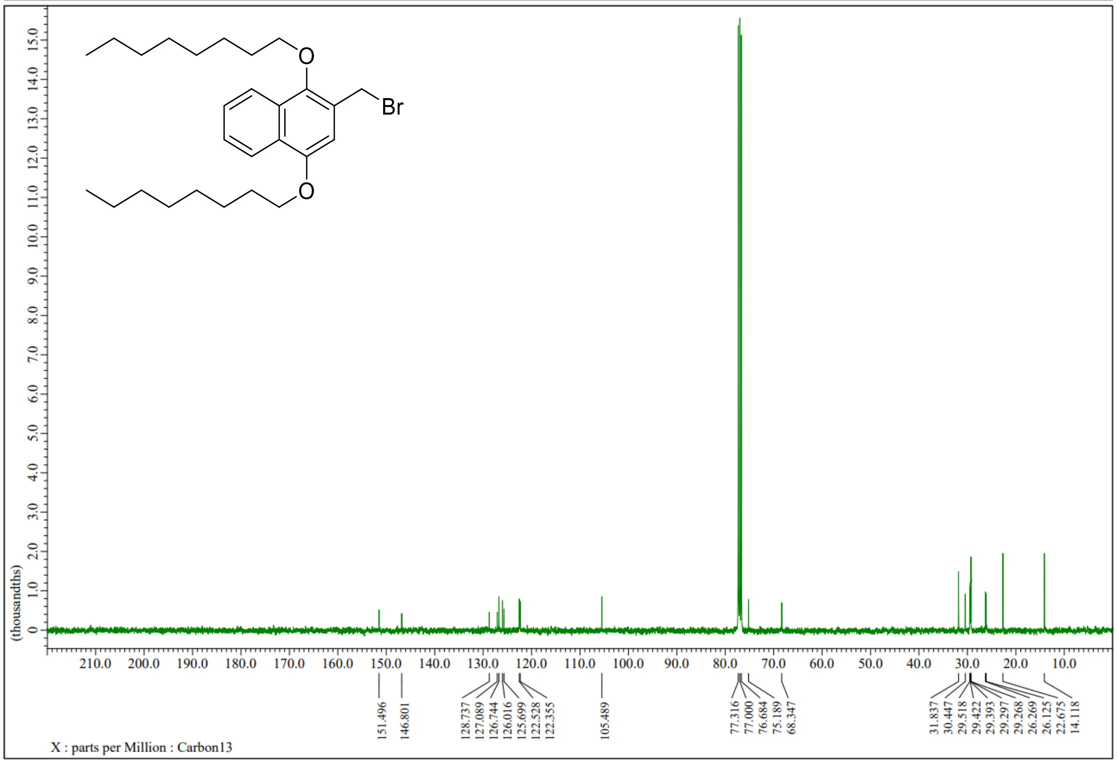

Figure S24. 2-(Bromomethyl)-1,4-bis(octyloxy)naphthalene **(12h)**

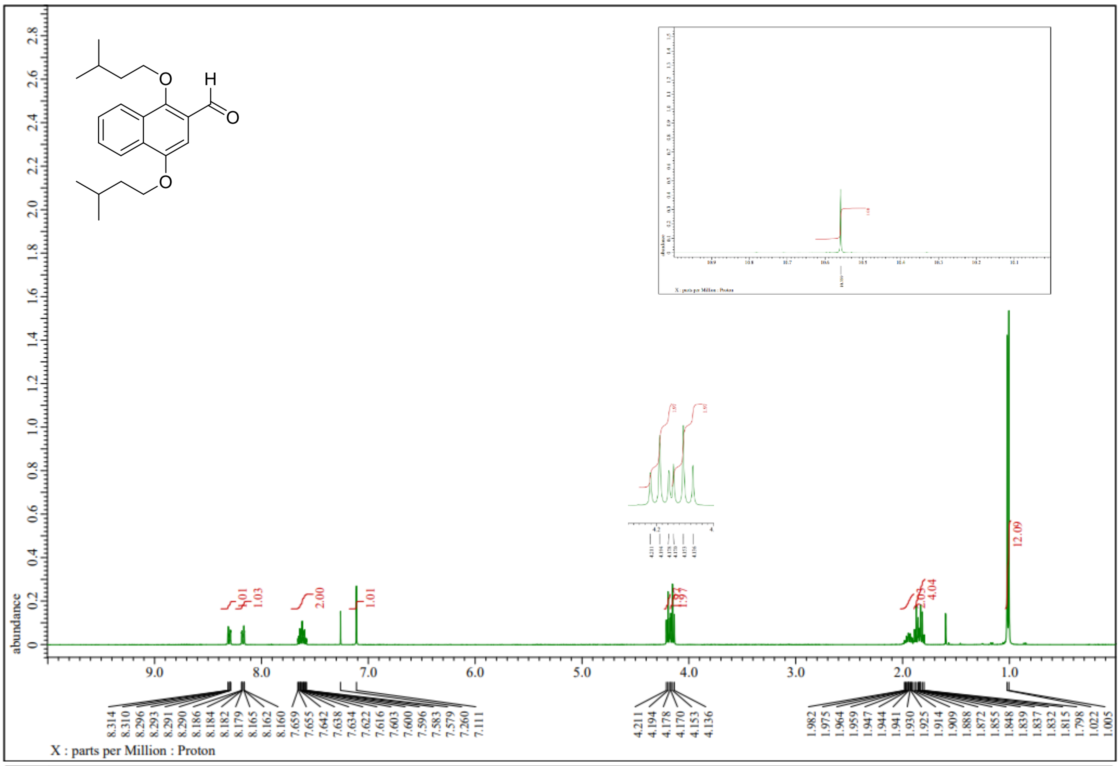

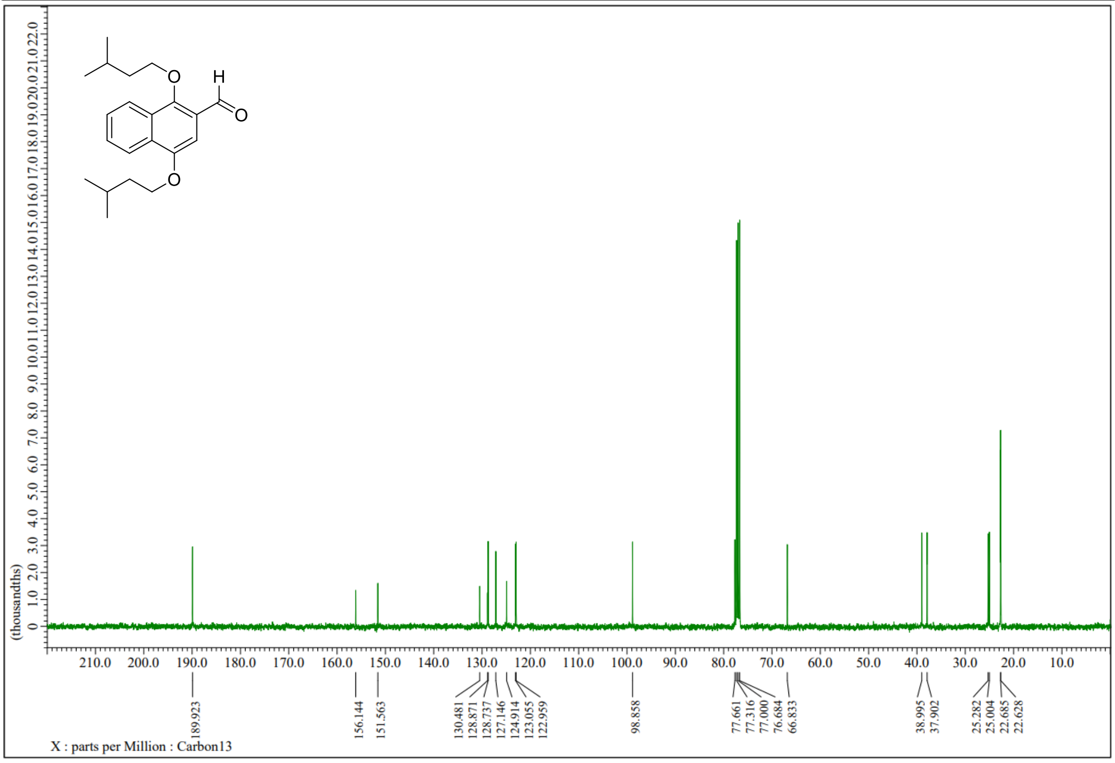

Figure S25. 1,4-Bis(isoamyloxy)-2-naphthaldehyde **(6)**

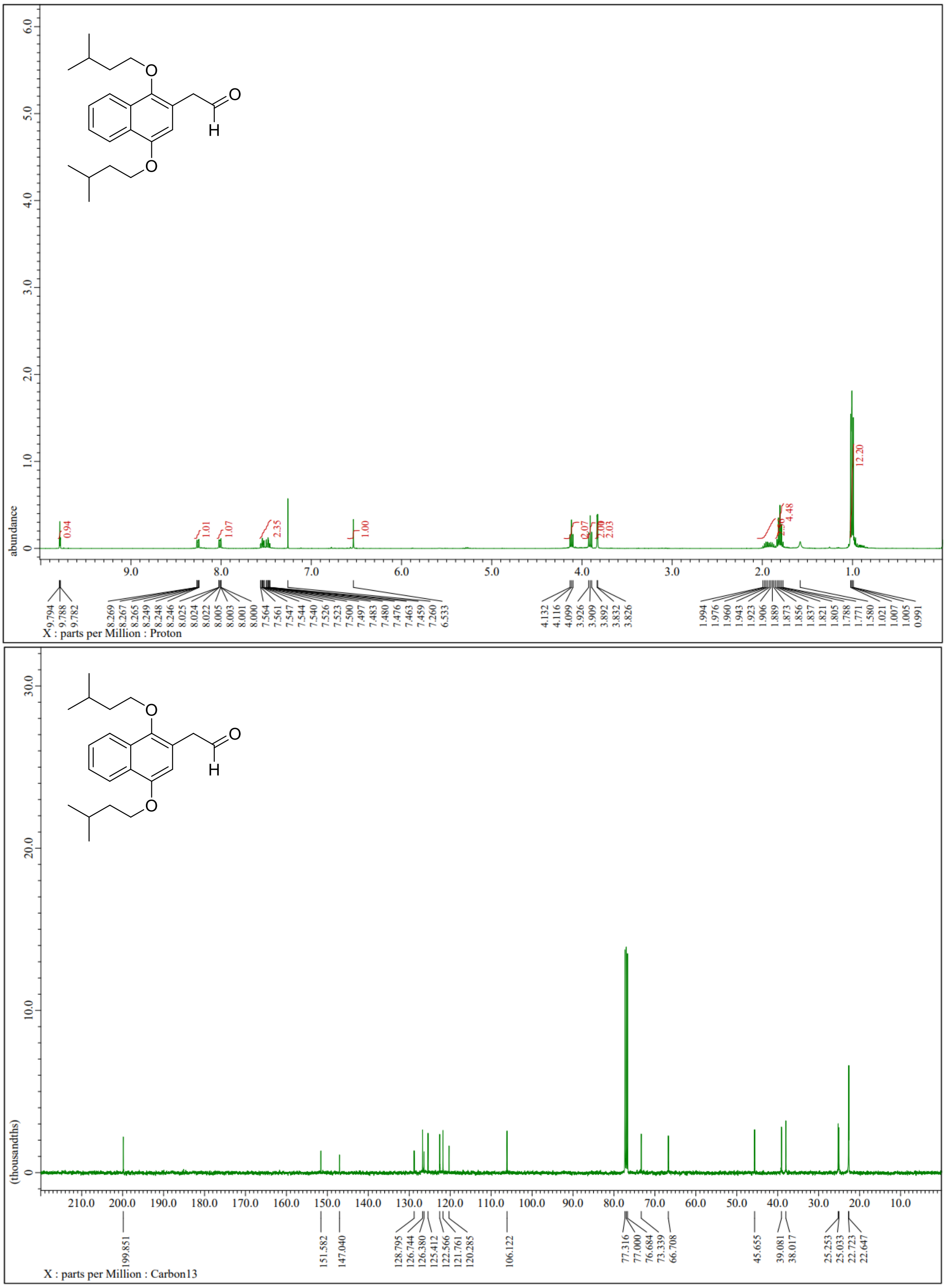

Figure S26. 2-(1,4-Bis(isoamyloxy)naphthalen-2-yl)acetaldehyde **(7)**

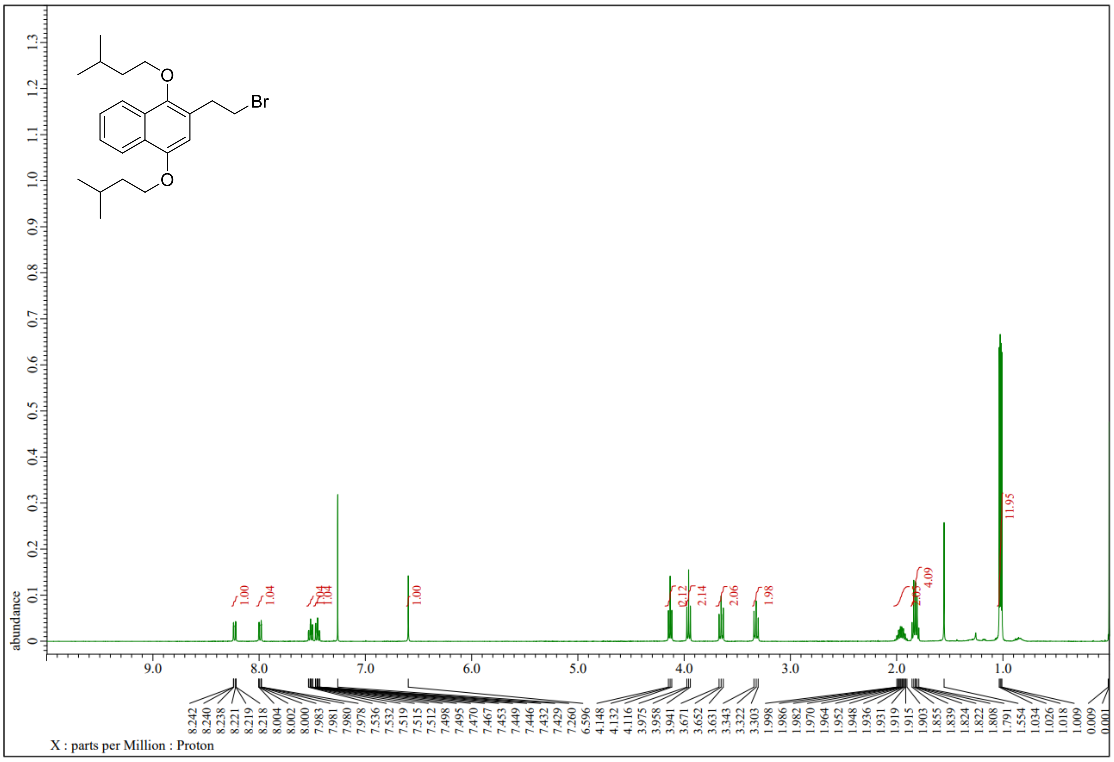

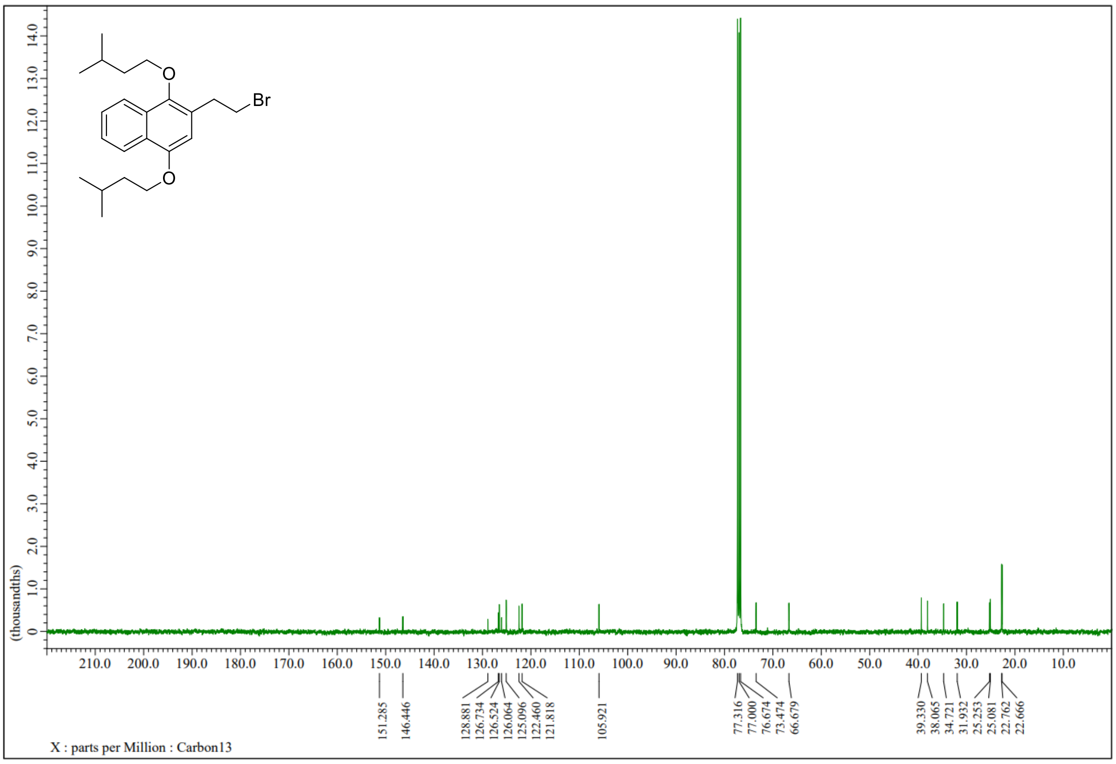

Figure S27. 2-(2-Bromoethyl)-1,4-bis(isoamyloxy)naphthalene **(8)**

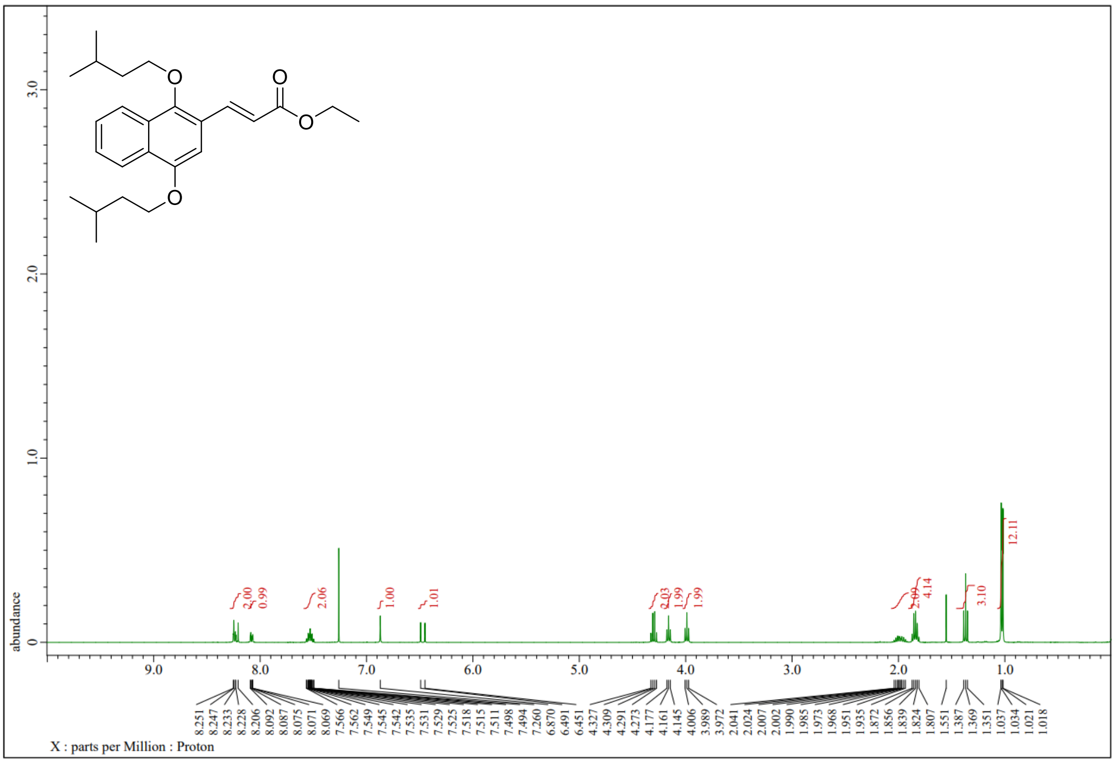

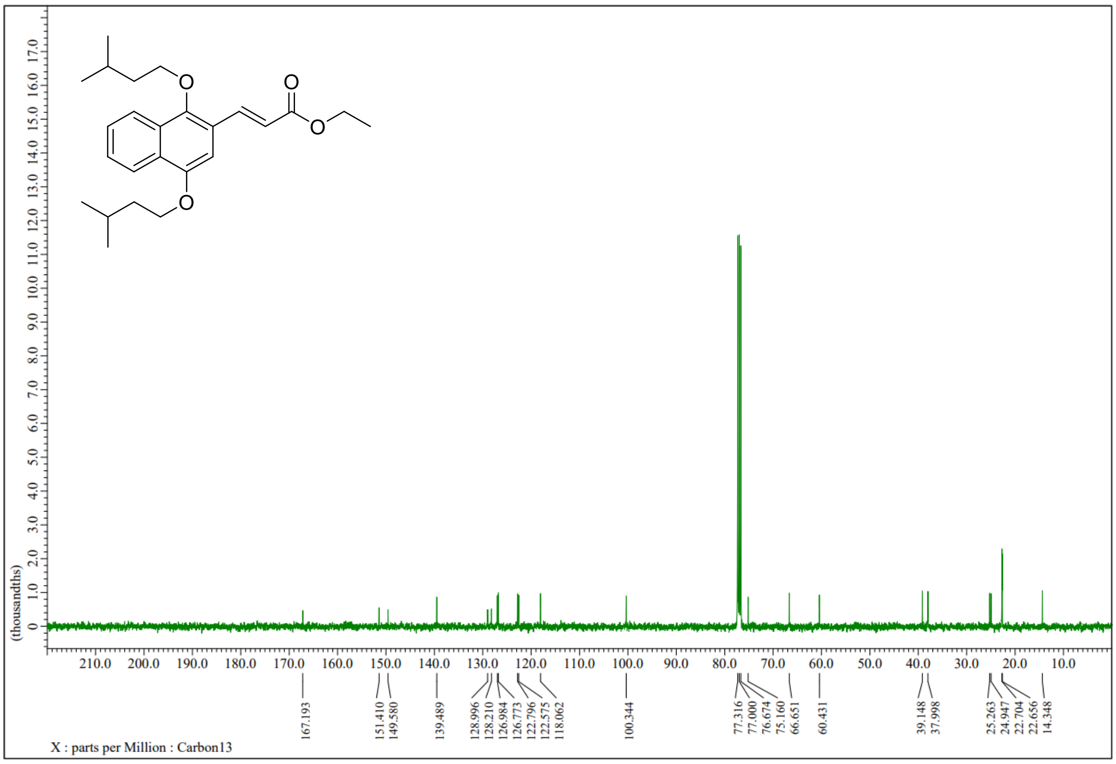

Figure S28. Ethyl 3-(1,4-bis(isoamyloxy)naphthalen-2-yl)acrylate **(9)**

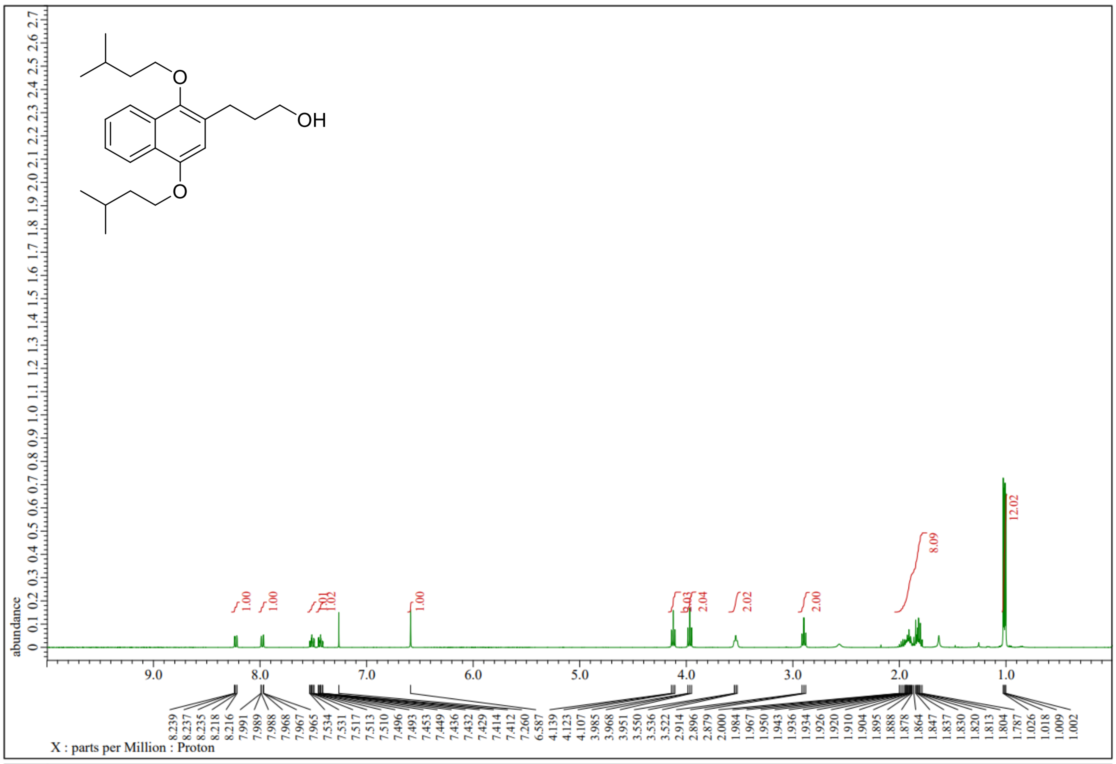

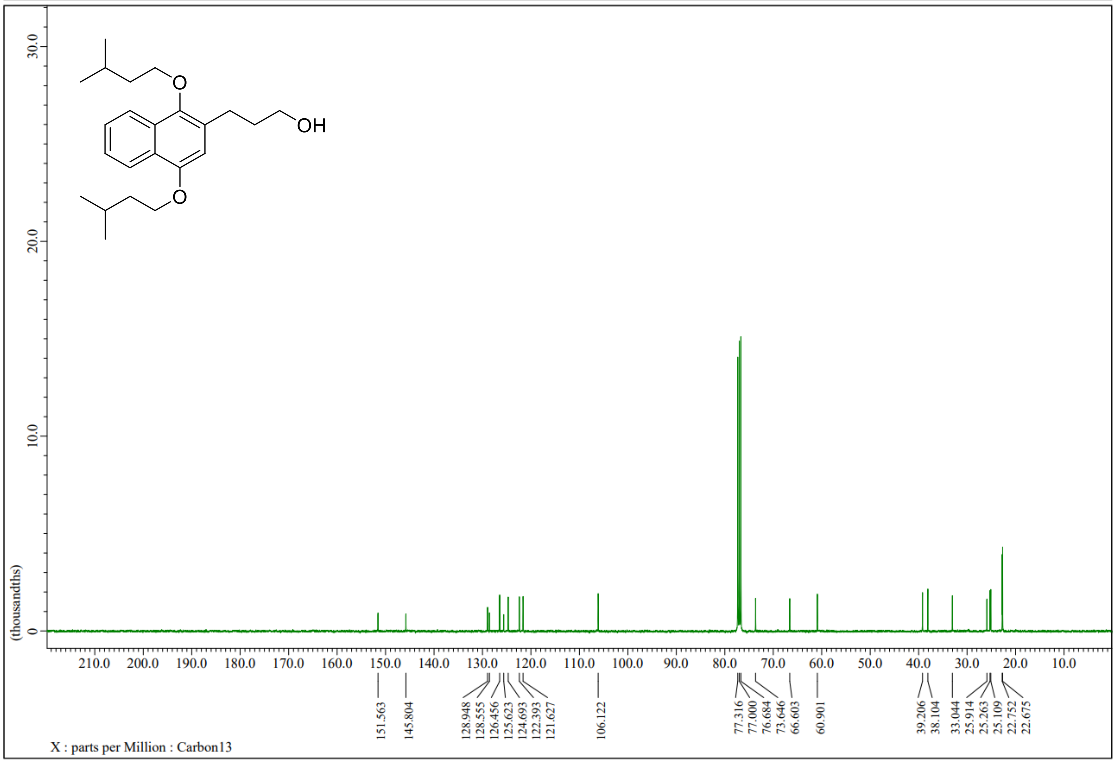

Figure S29. 3-(1,4-Bis(isoamyloxy)naphthalen-2-yl)propan-1-ol **(10)**

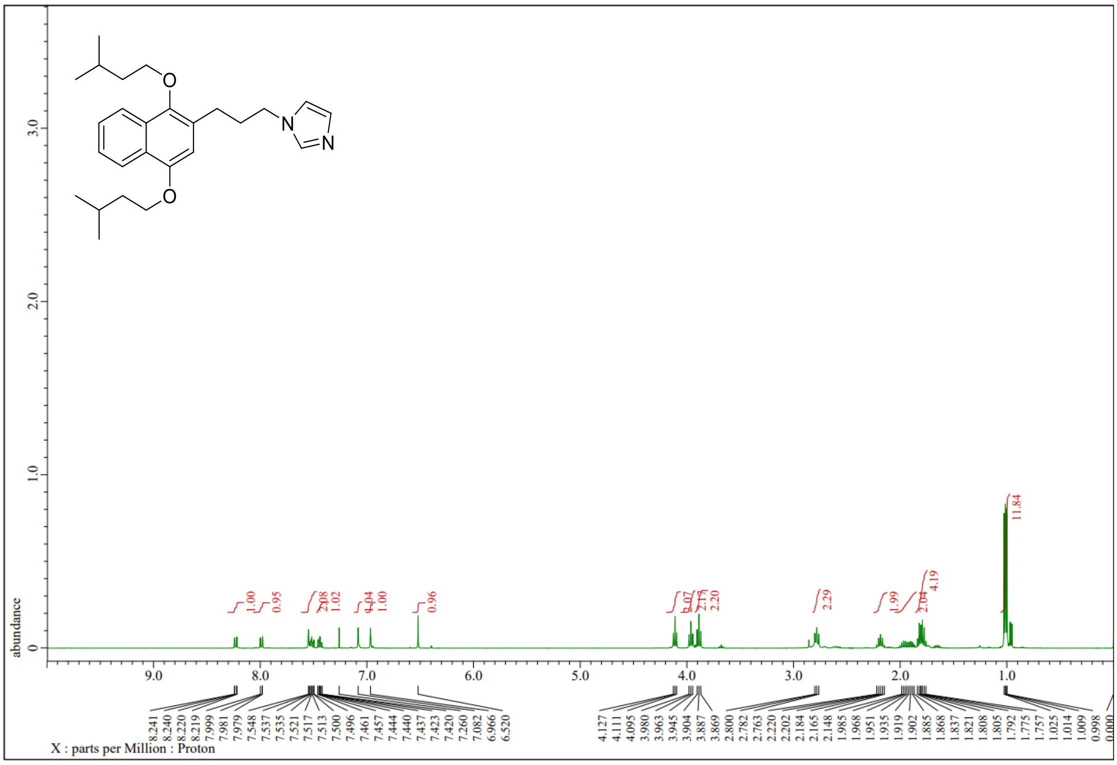

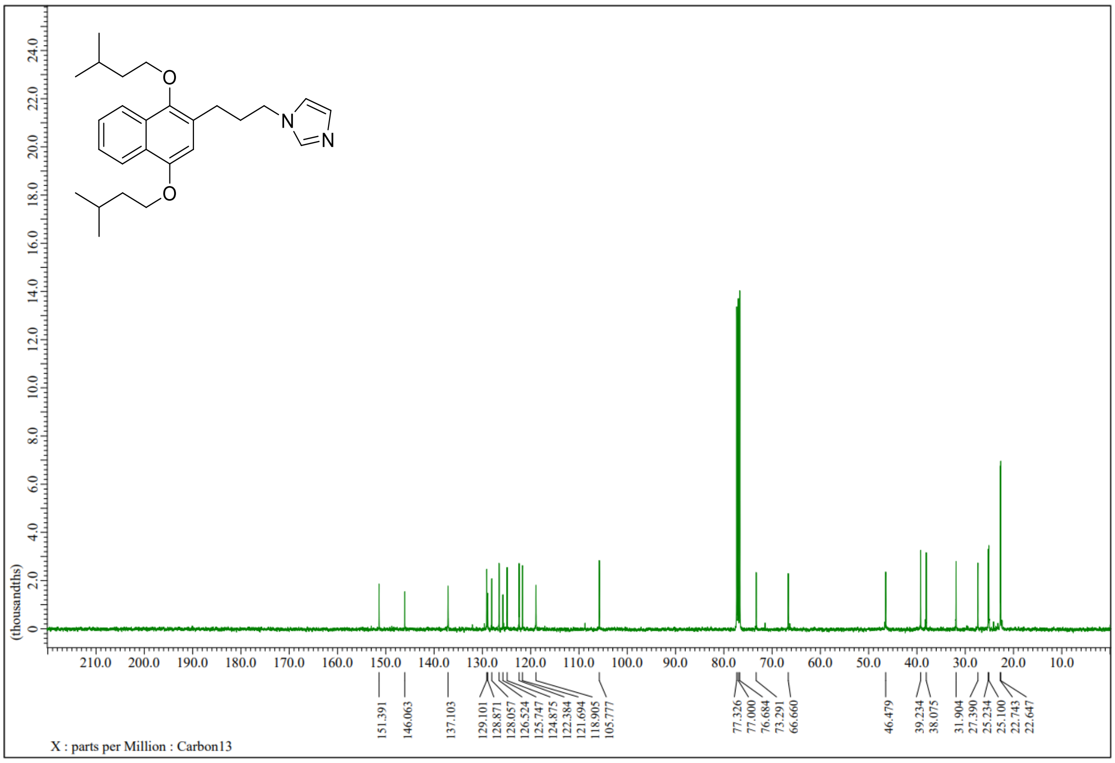

Figure S30. 1-(3-(1,4-Bis(isoamyloxy)naphthalen-2-yl)propyl)-1H-imidazole **(11)**

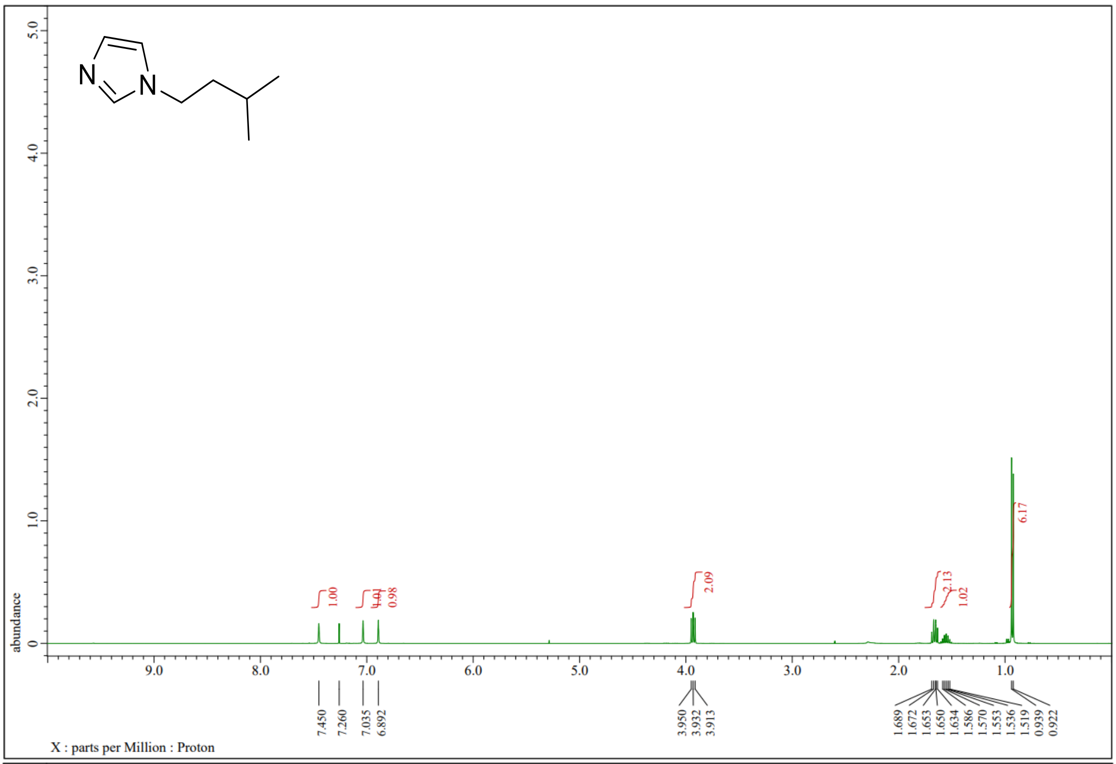

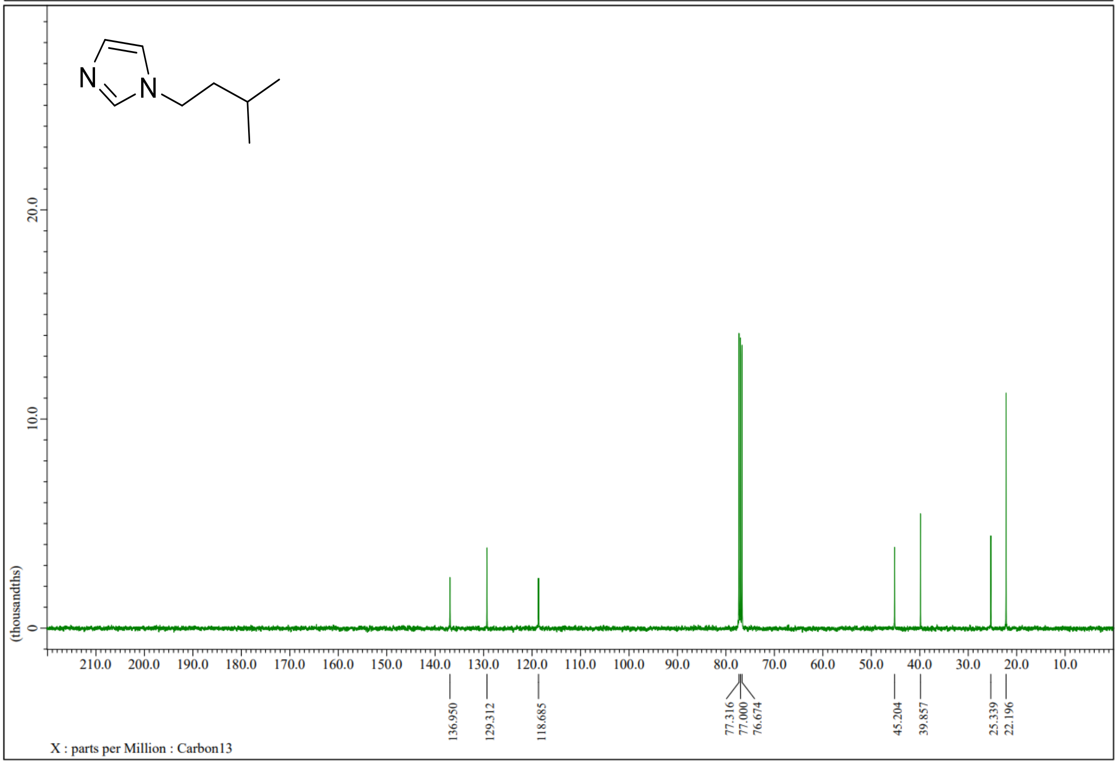

Figure S31. 1-Isoamyl-1*H*-imidazole **(13b)**

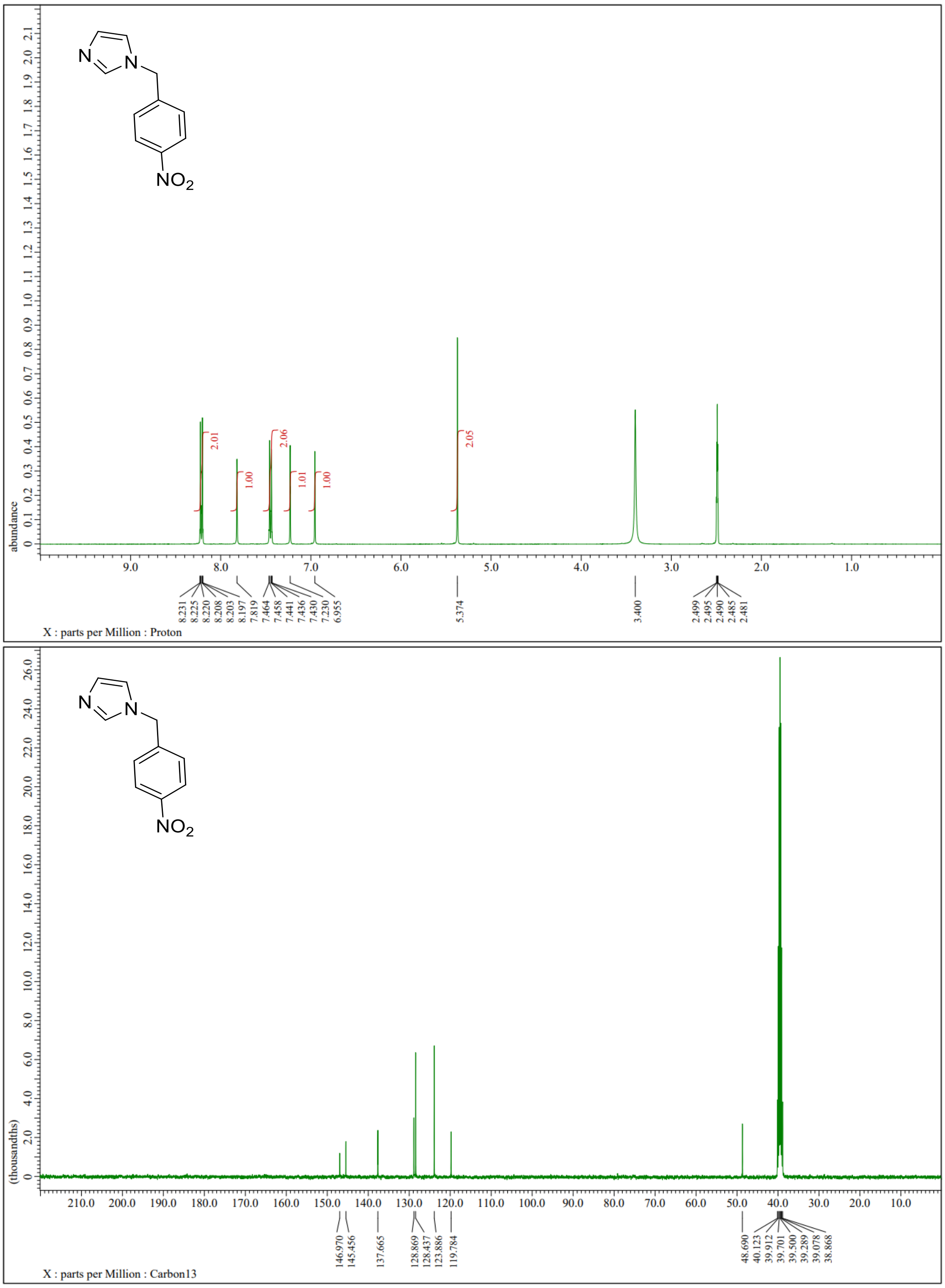

Figure S32. 1-(4-Nitrobenzyl)-1*H*-imidazole **(13d)**

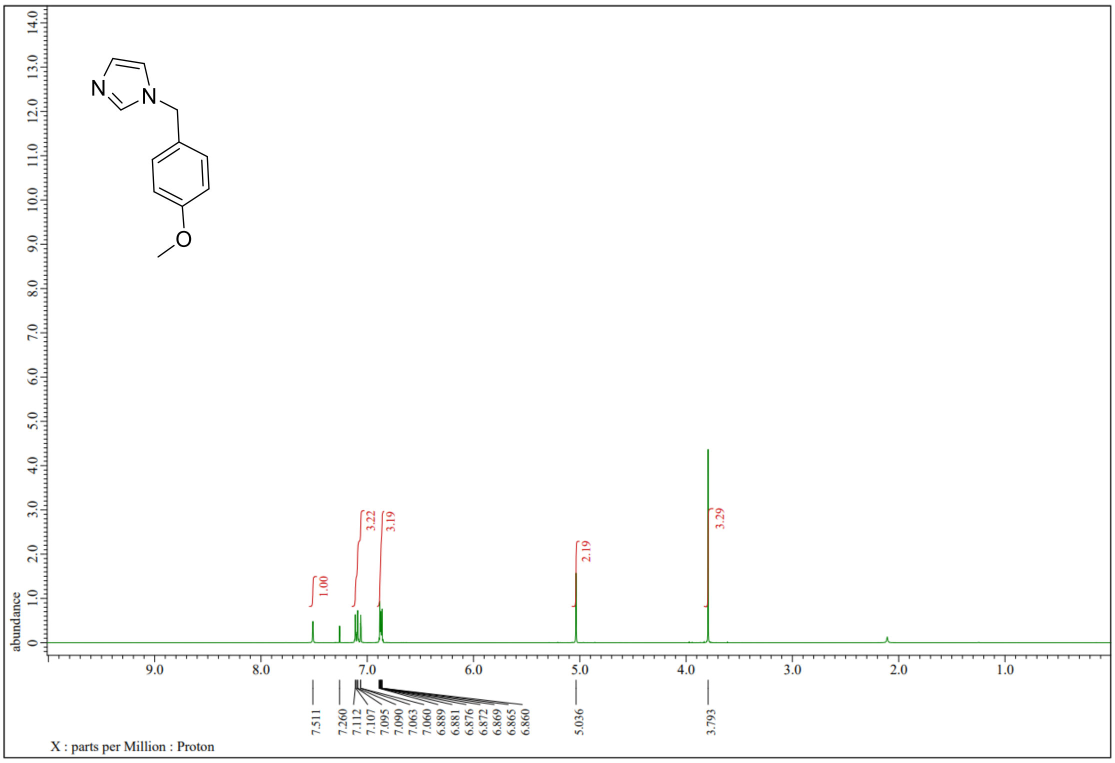

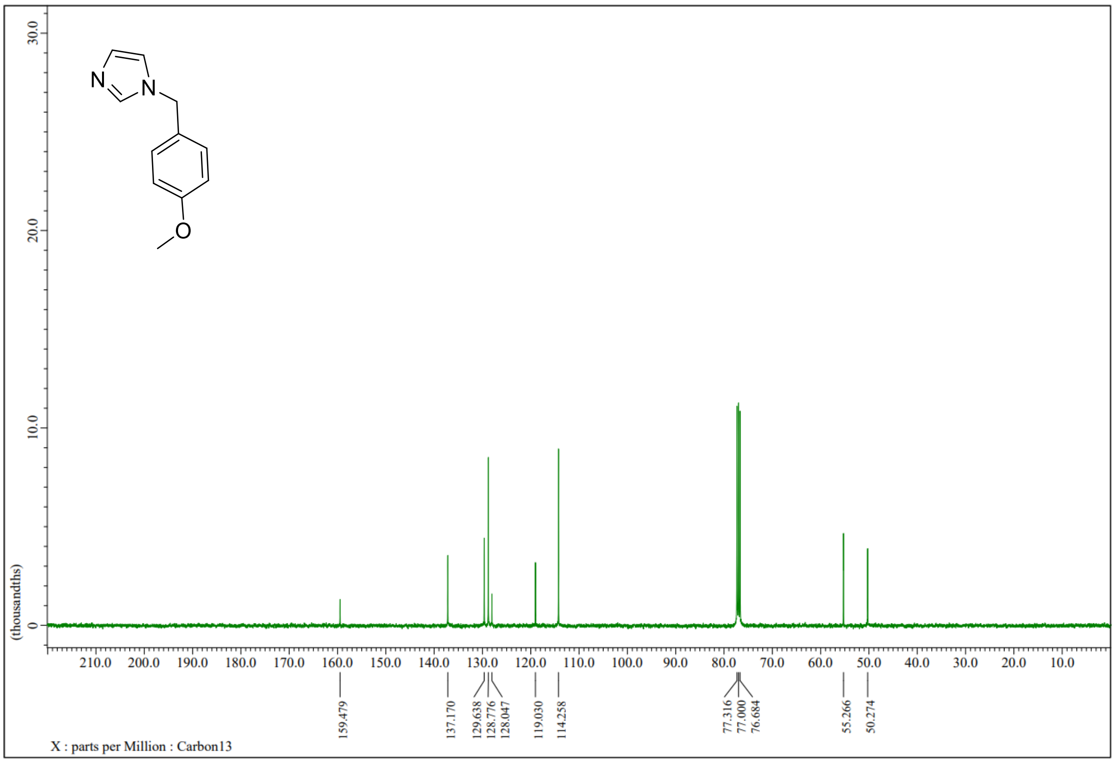

Figure S33. 1-(4-Methoxybenzyl)-1*H*-imidazole **(13e)**

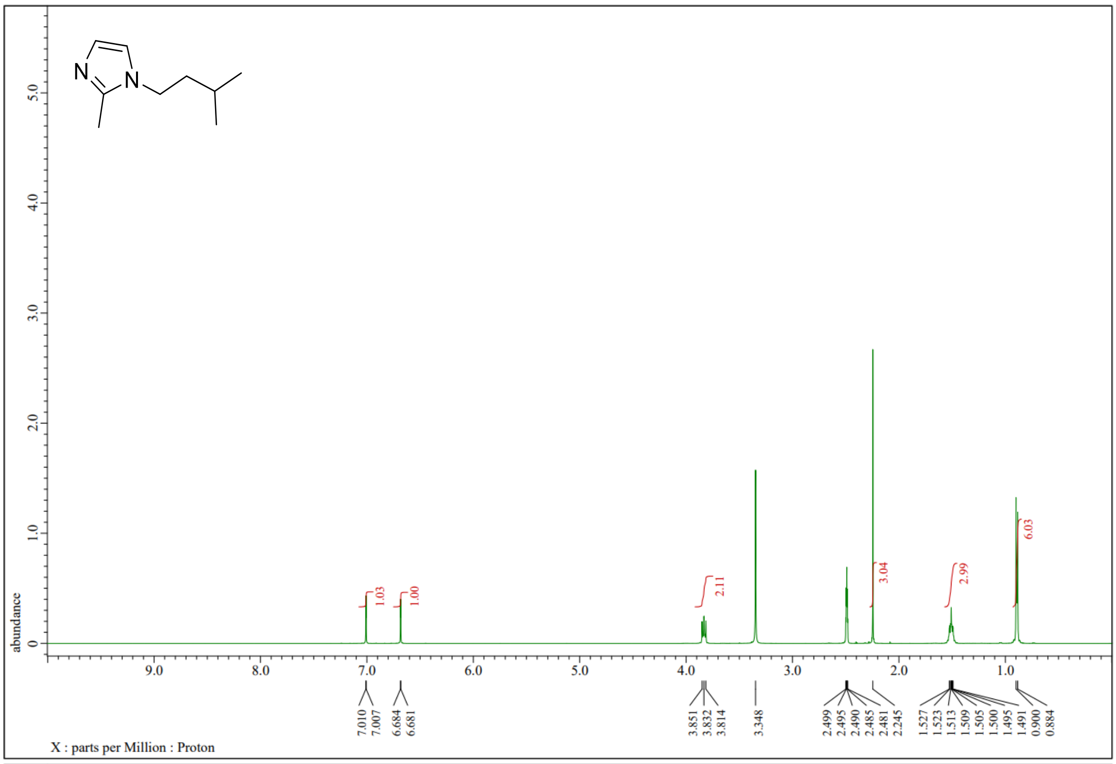

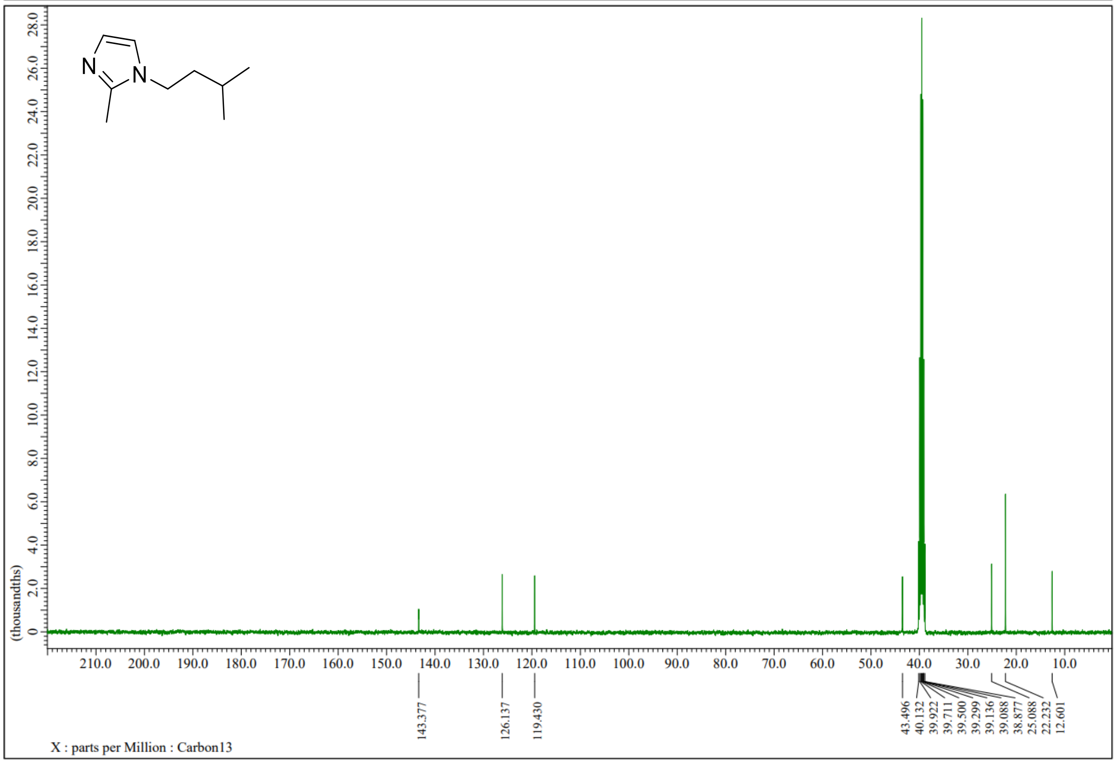

Figure S34. 1-Isoamyl-2-methyl-1*H*-imidazole **(14a)**

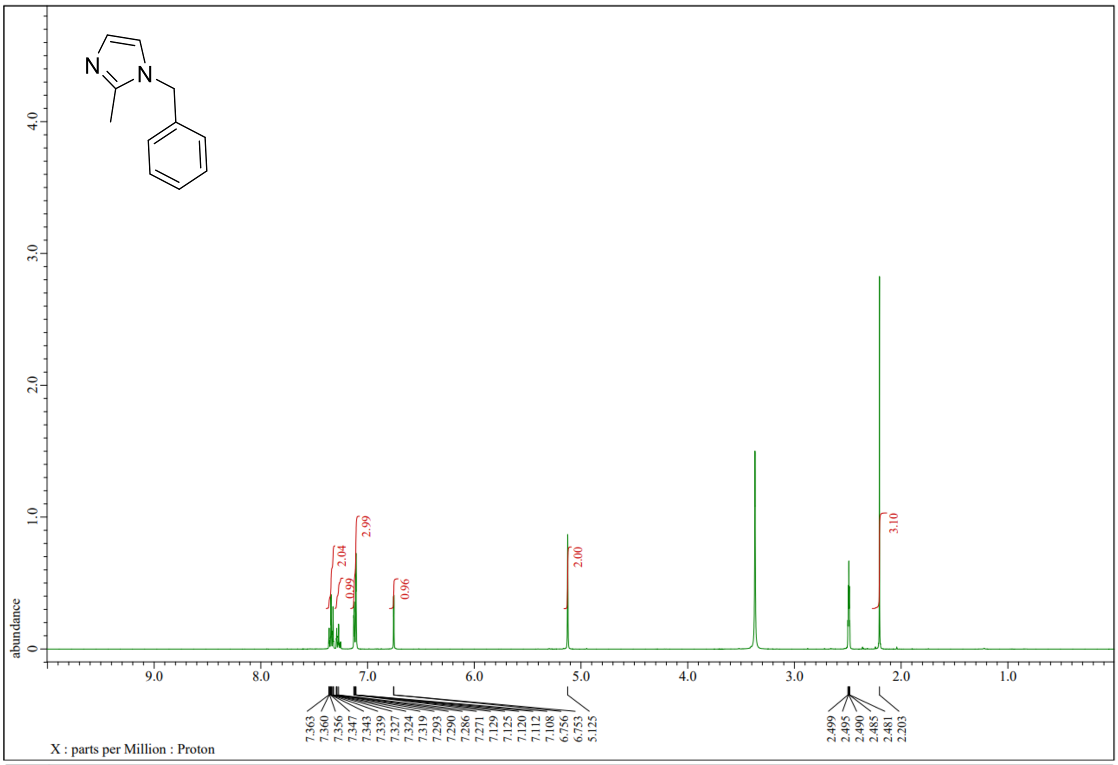

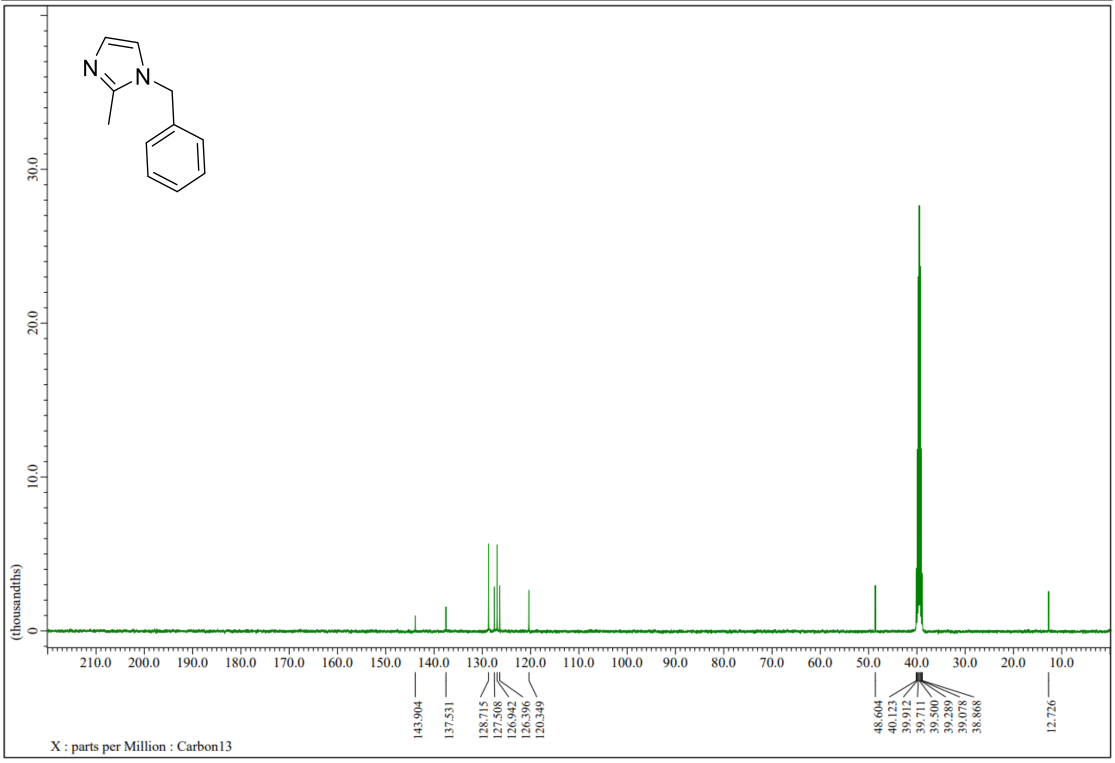

Figure S35. 1-Benzyl-2-methyl-1*H*-imidazole **(14b)**

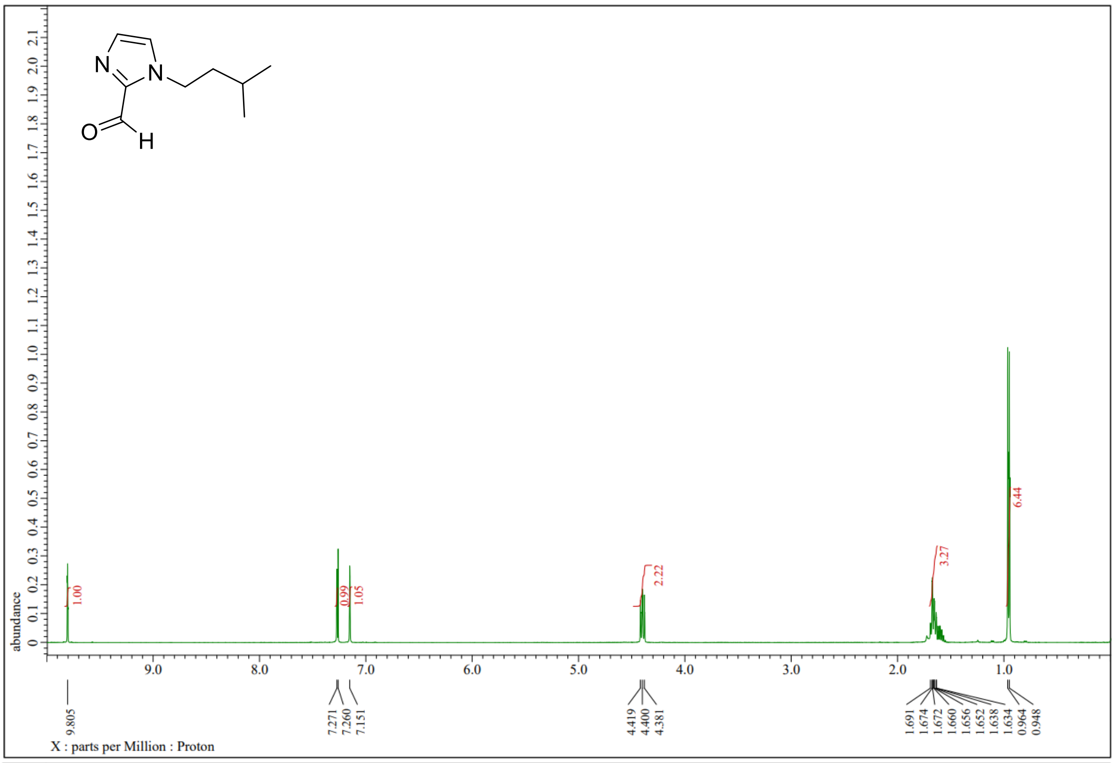

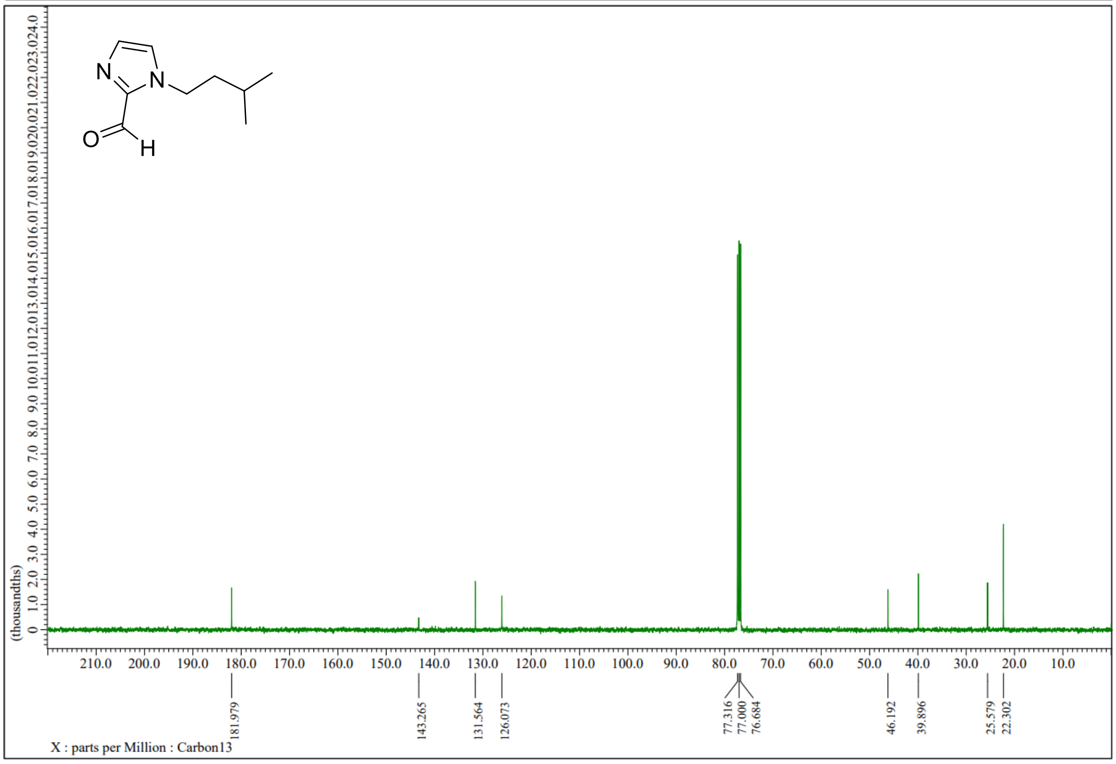

Figure S36. 1-Isoamyl-1*H*-imidazole-2-carbaldehyde **(14c)**

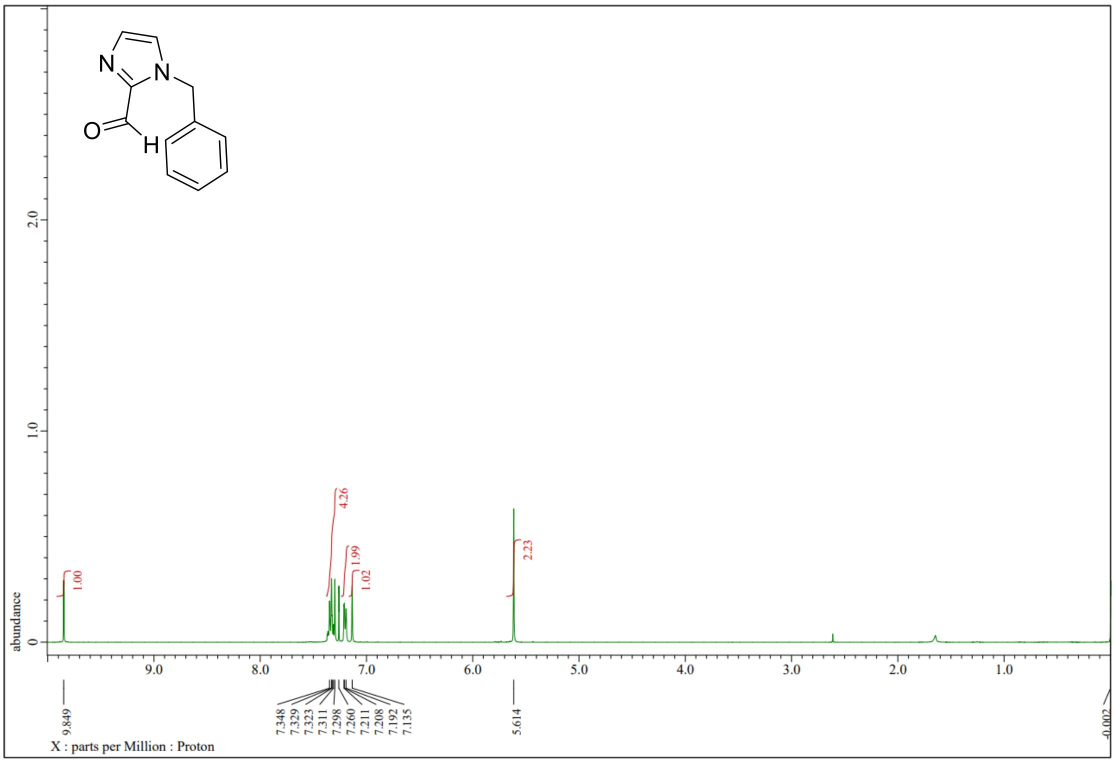

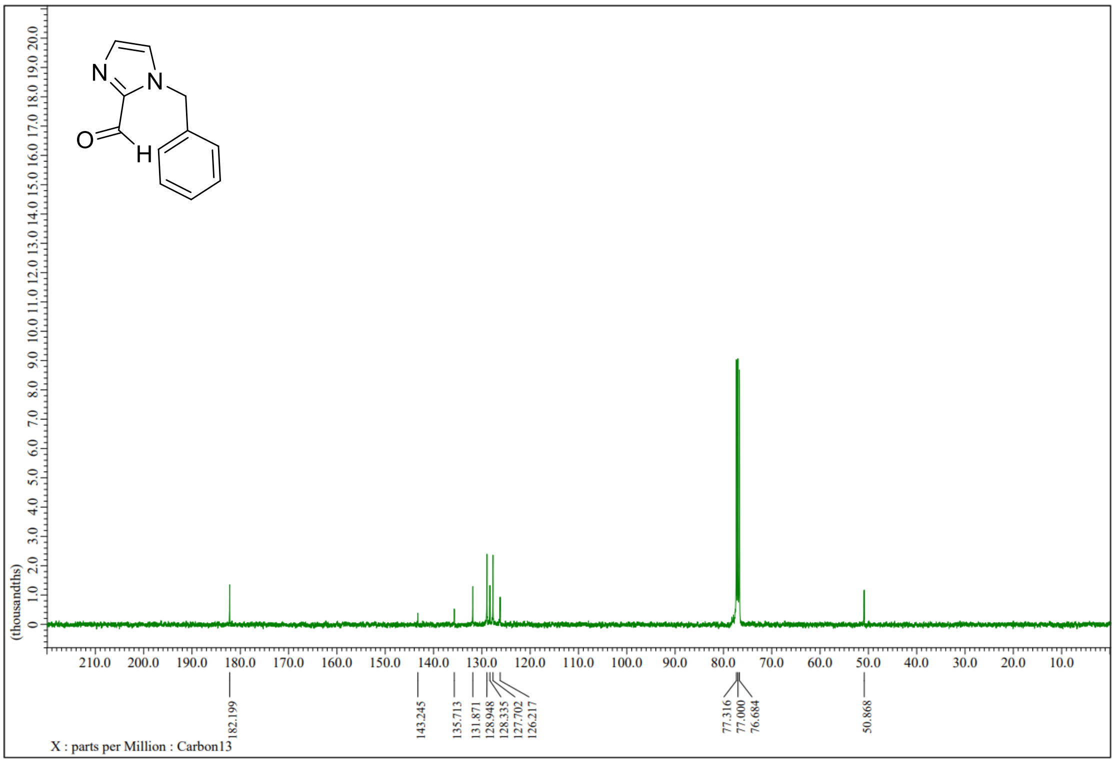

Figure S37. 1-Benzyl-1H-imidazole-2-carbaldehyde **(14d)**

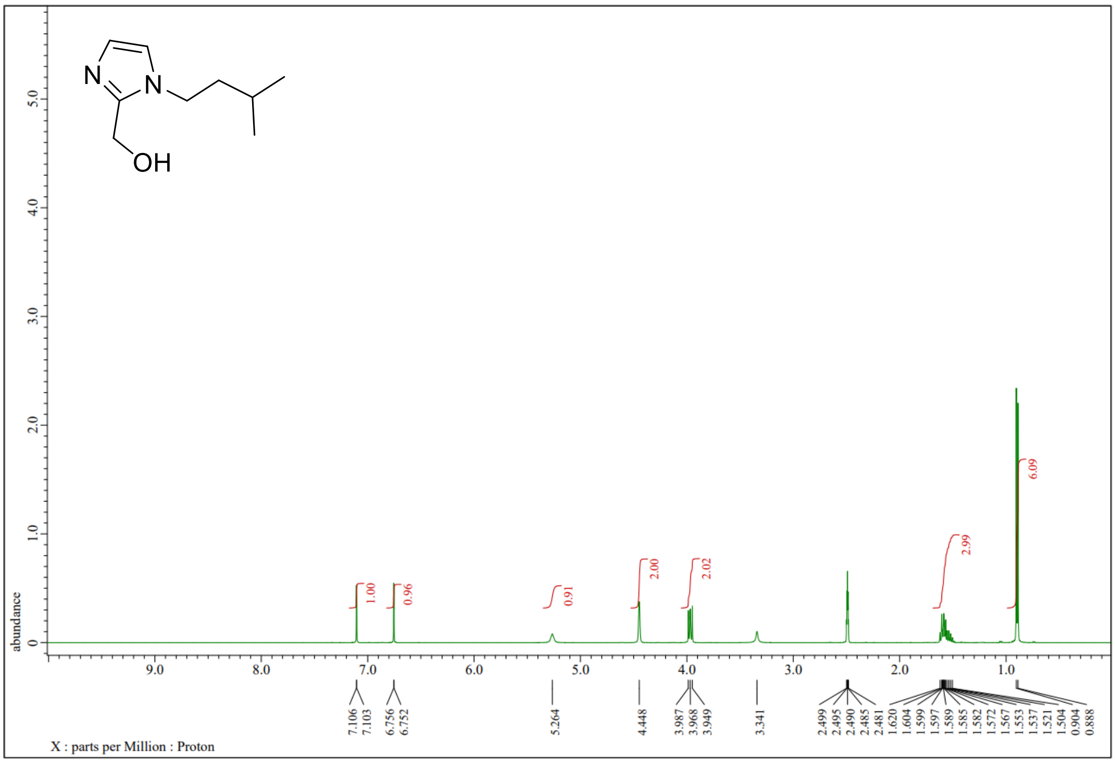

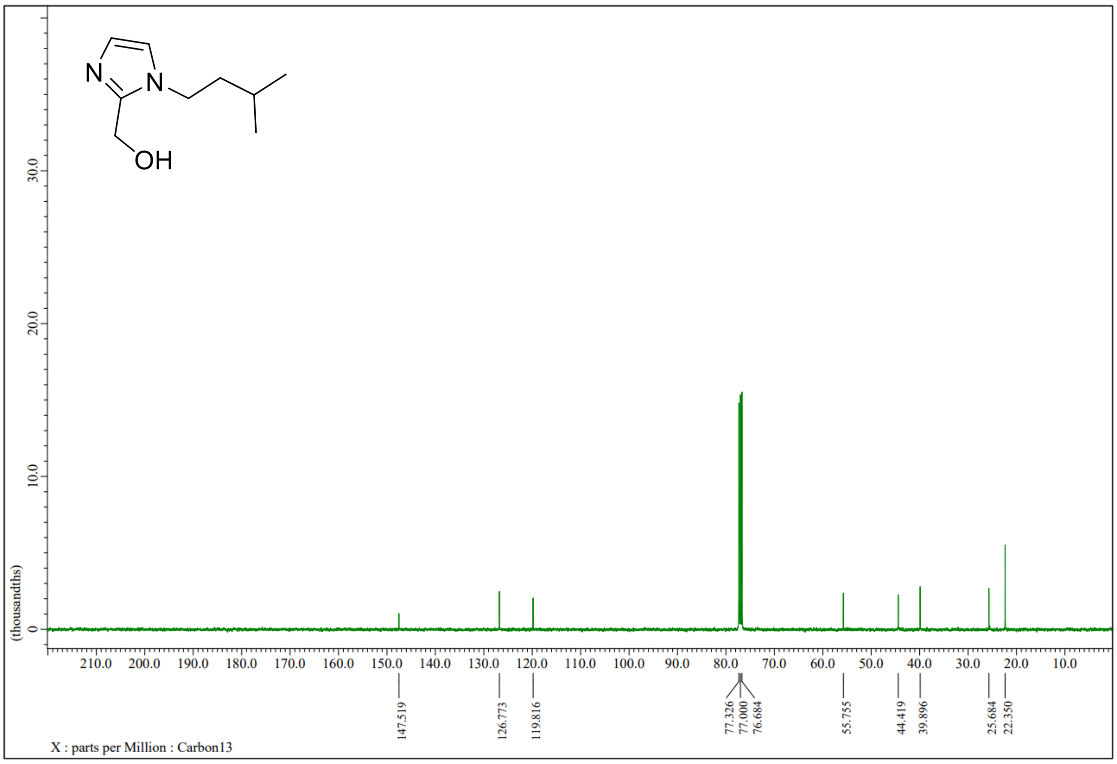

Figure S38. (1-Isoamyl-1H-imidazol-2-yl)methanol **(14e)**

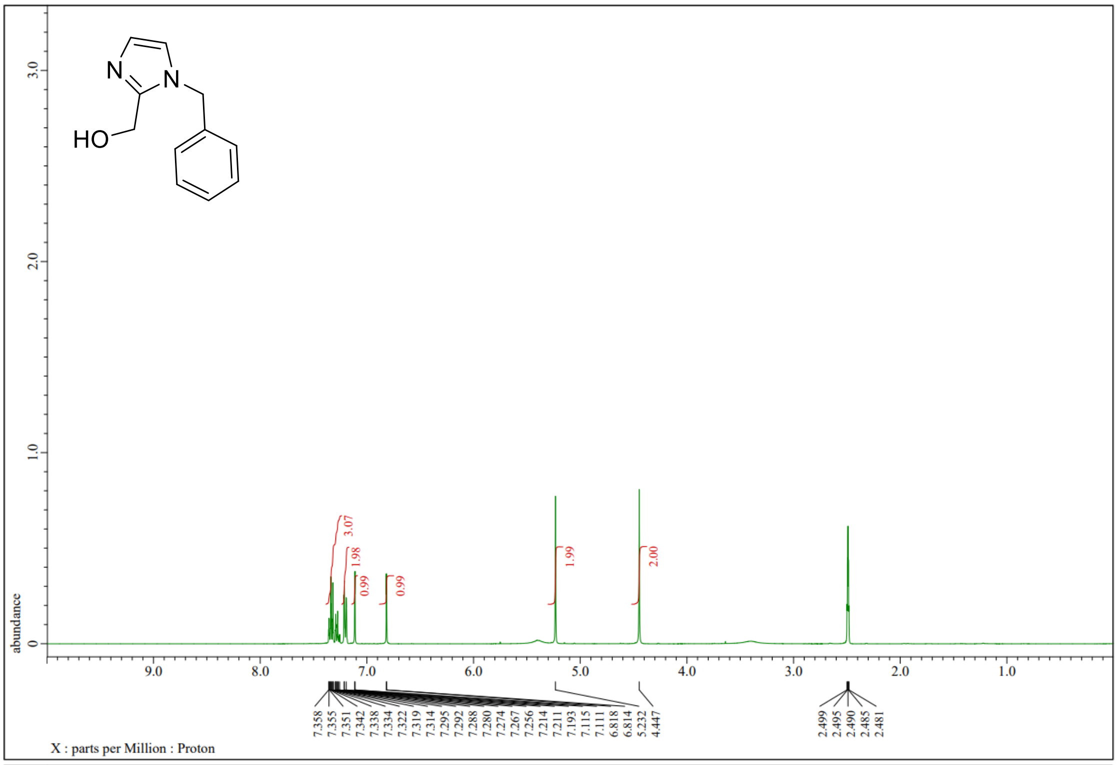

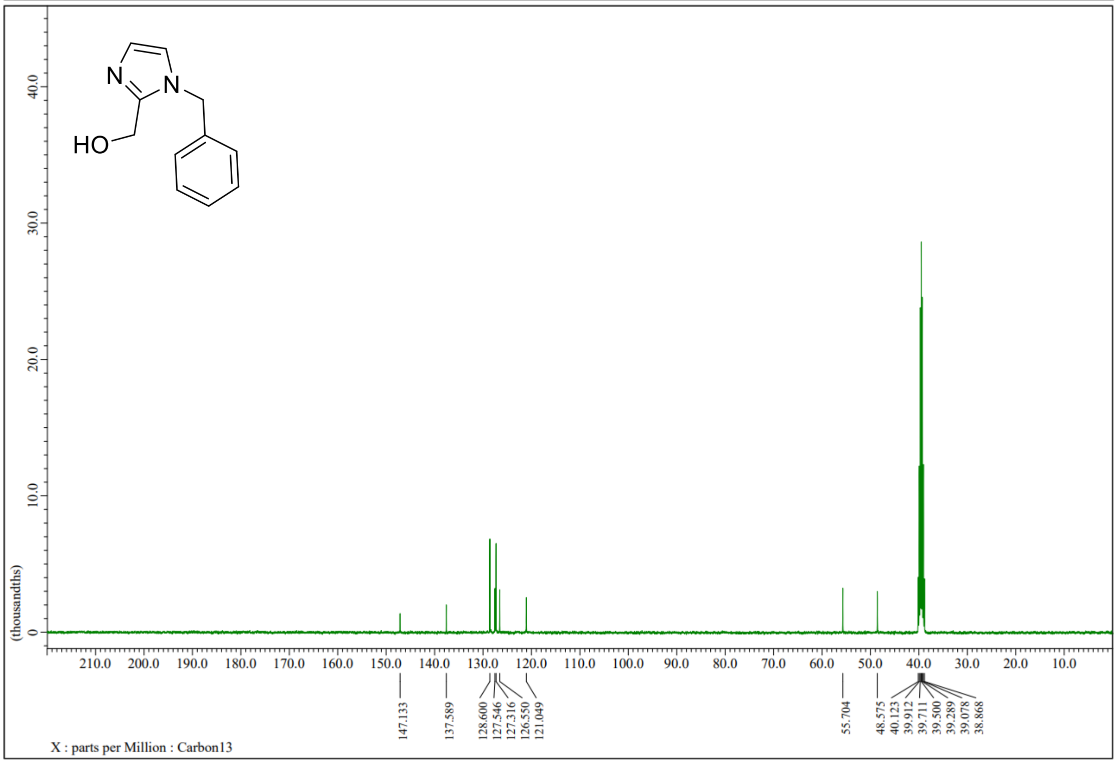

Figure S39. (1-Benzyl-1H-imidazol-2-yl)methanol **(14f)**

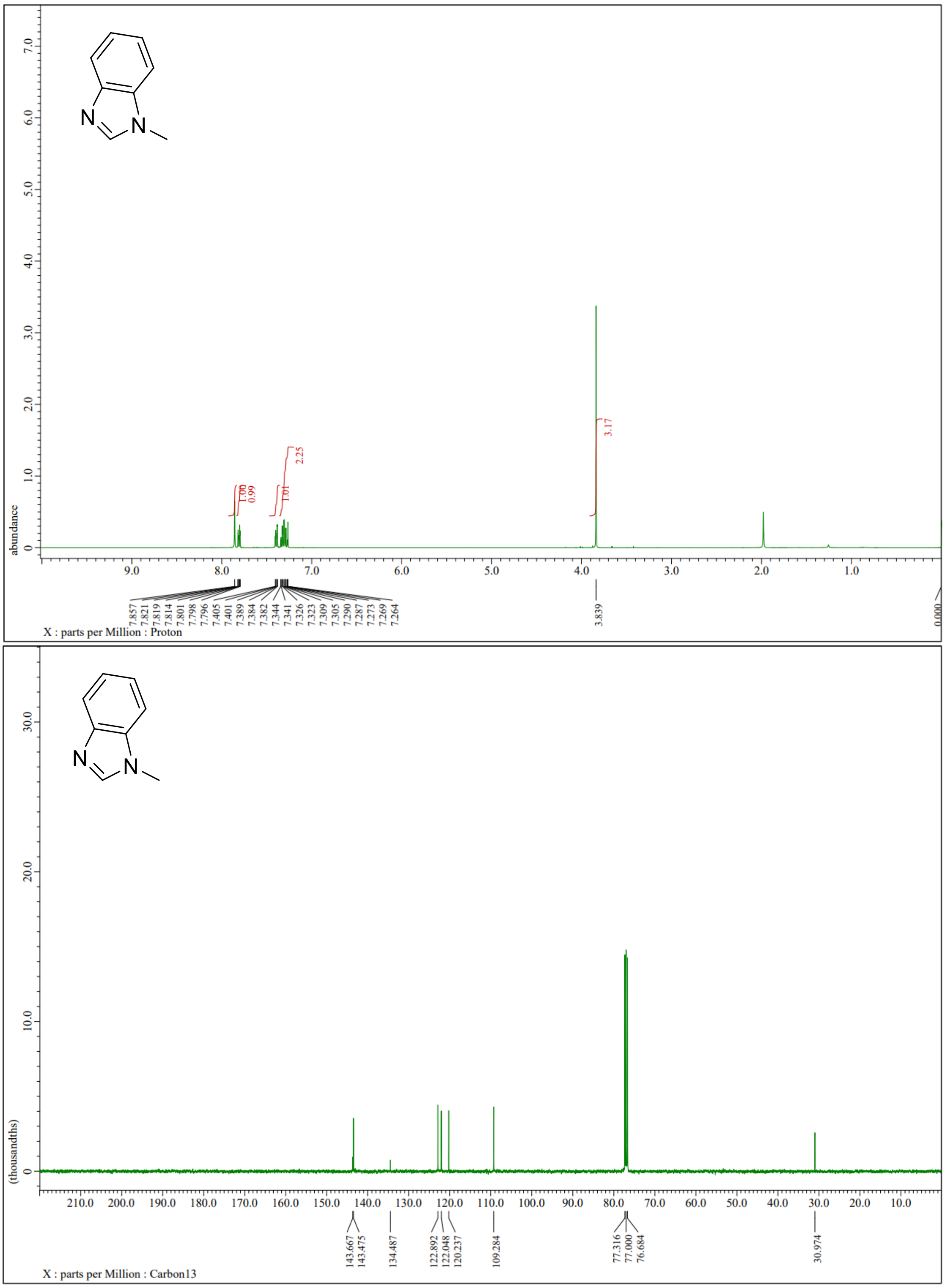

Figure S40. 1-Methyl-1*H*-benzo[*d*]imidazole **(15a)**

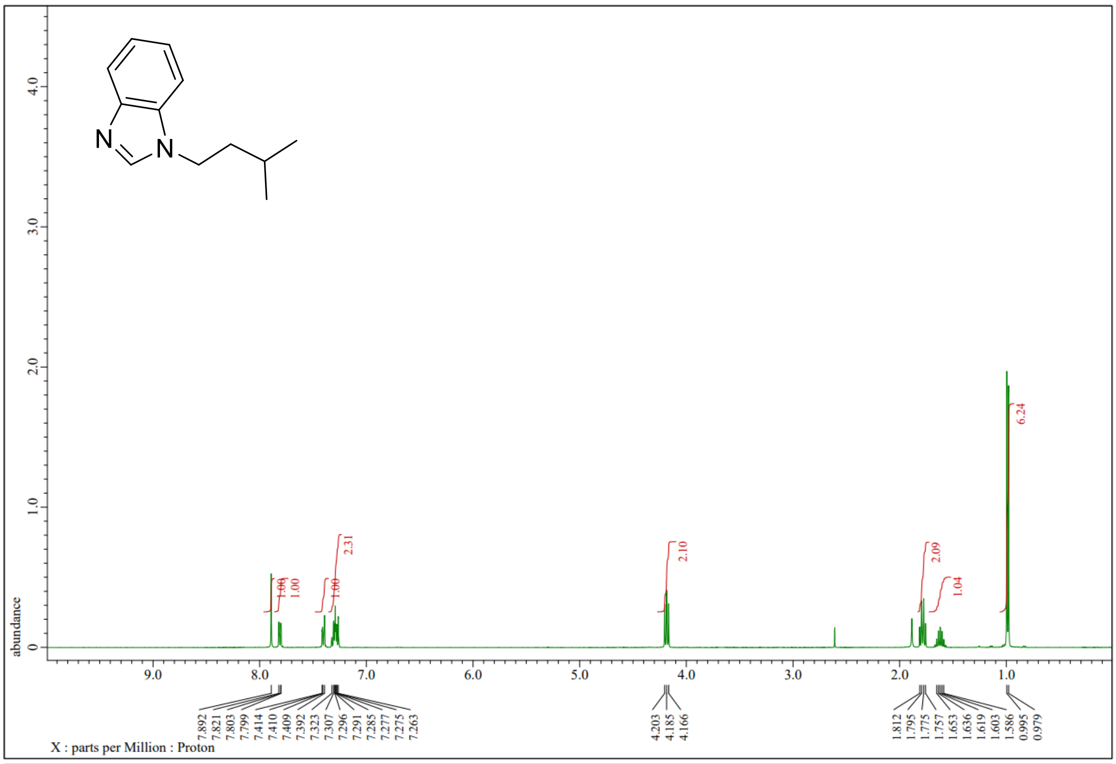

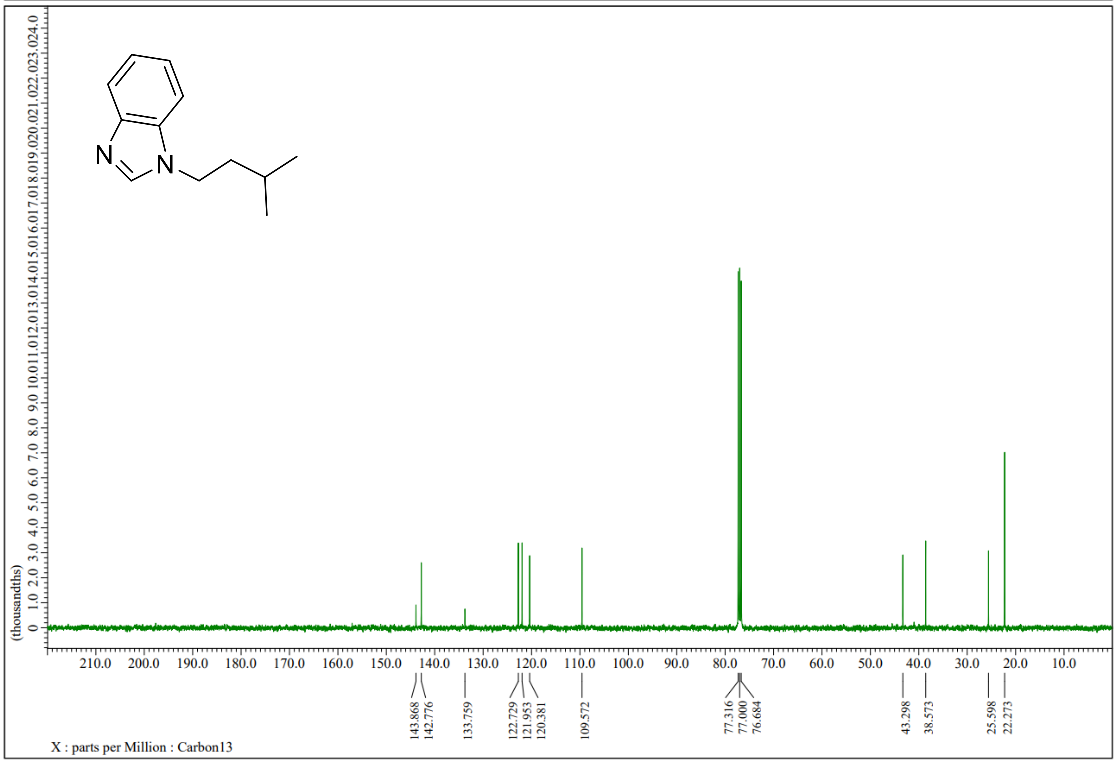

Figure S41. 1-Isoamyl-1*H*-benzo[*d*]imidazole **(15b)**

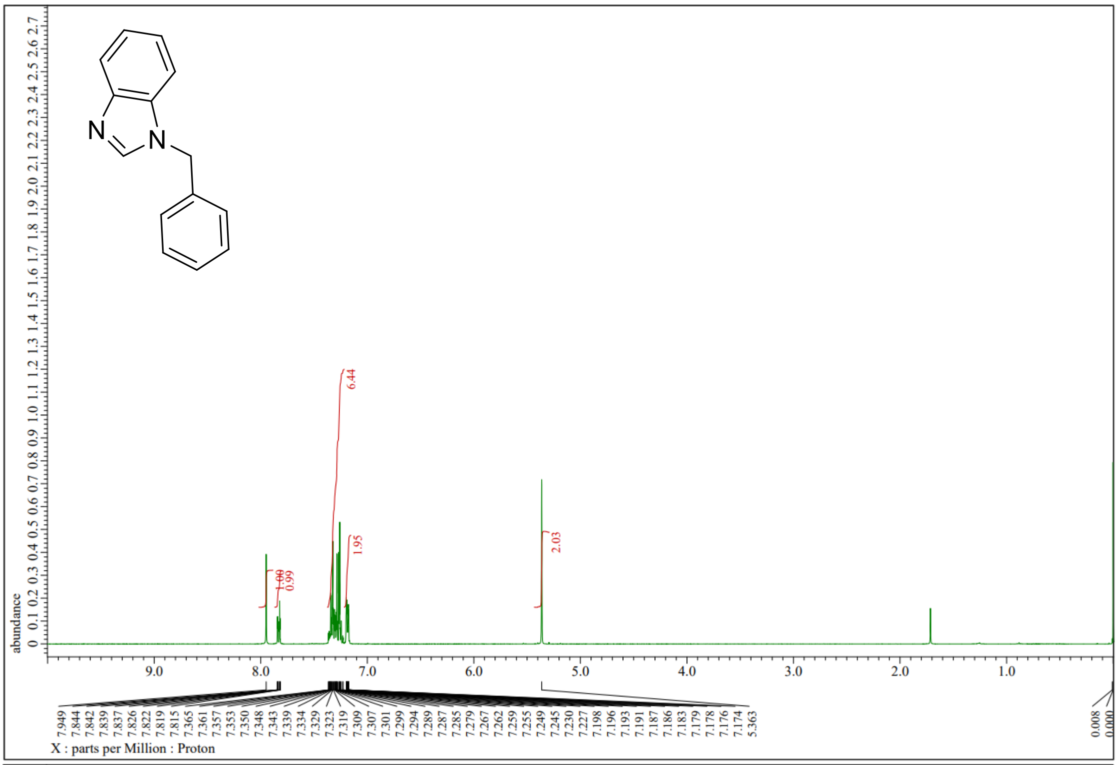

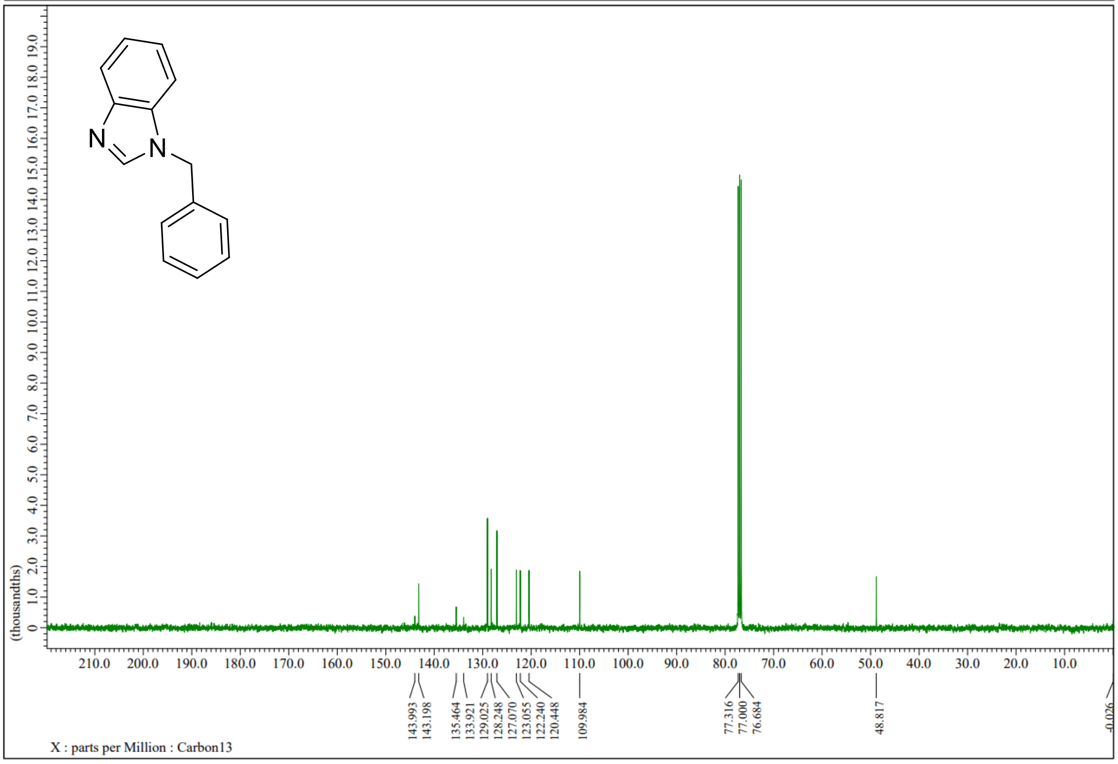

Figure S42. 1-Benzyl-1*H*-benzo[*d*]imidazole **(15c)**

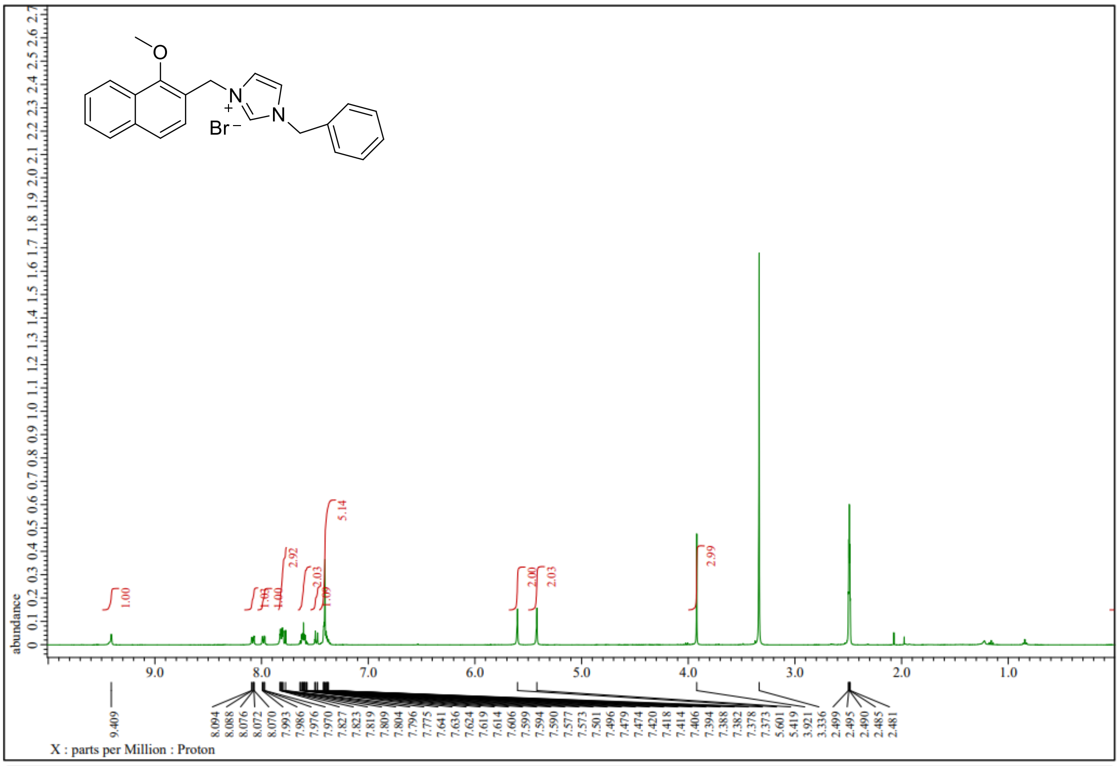

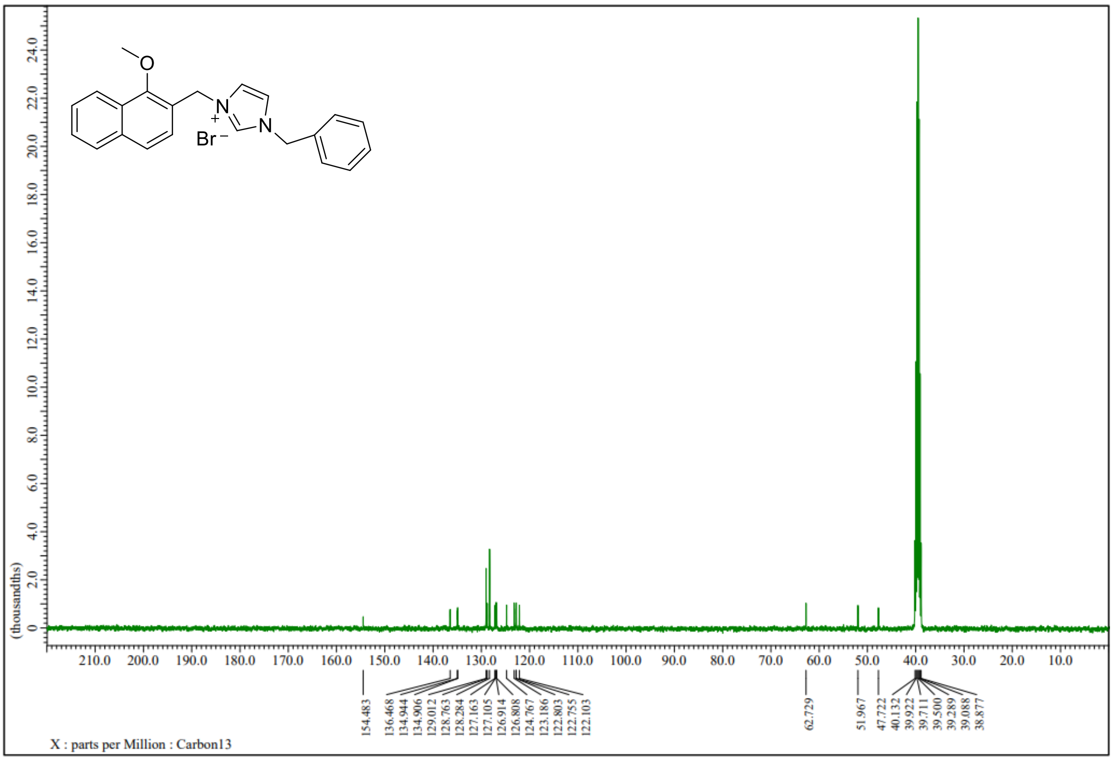

Figure S43. 1-Benzyl-3-((1-methoxynaphthalen-2-yl)methyl)-1*H*-imidazol-3-ium bromide **(IMS-01)**

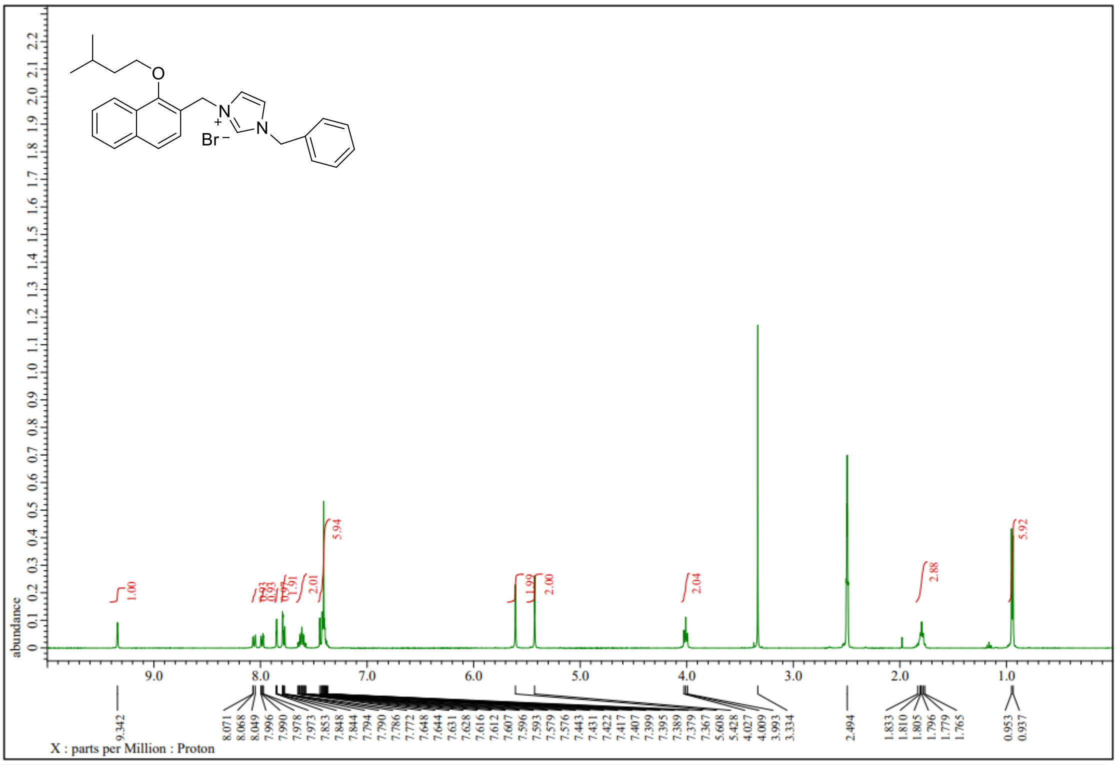

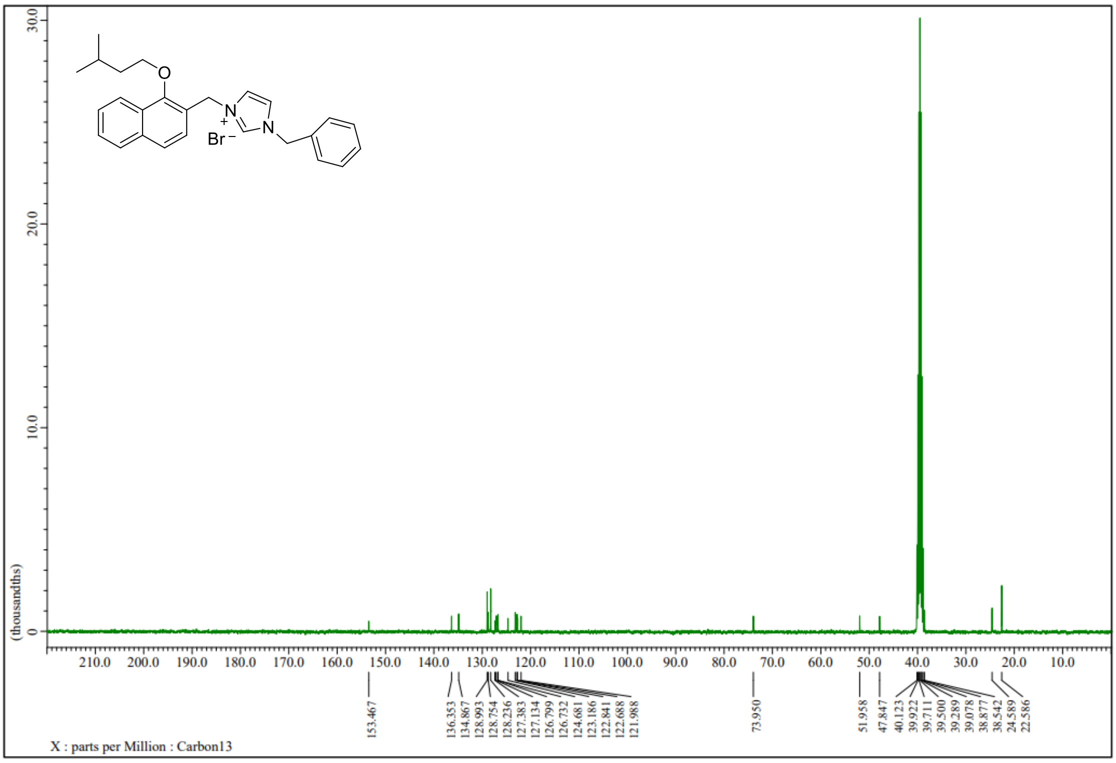

Figure S44. 1-Benzyl-3-((1-(isoamyloxy)naphthalen-2-yl)methyl)-1*H*-imidazol-3-ium bromide **(IMS-02)**

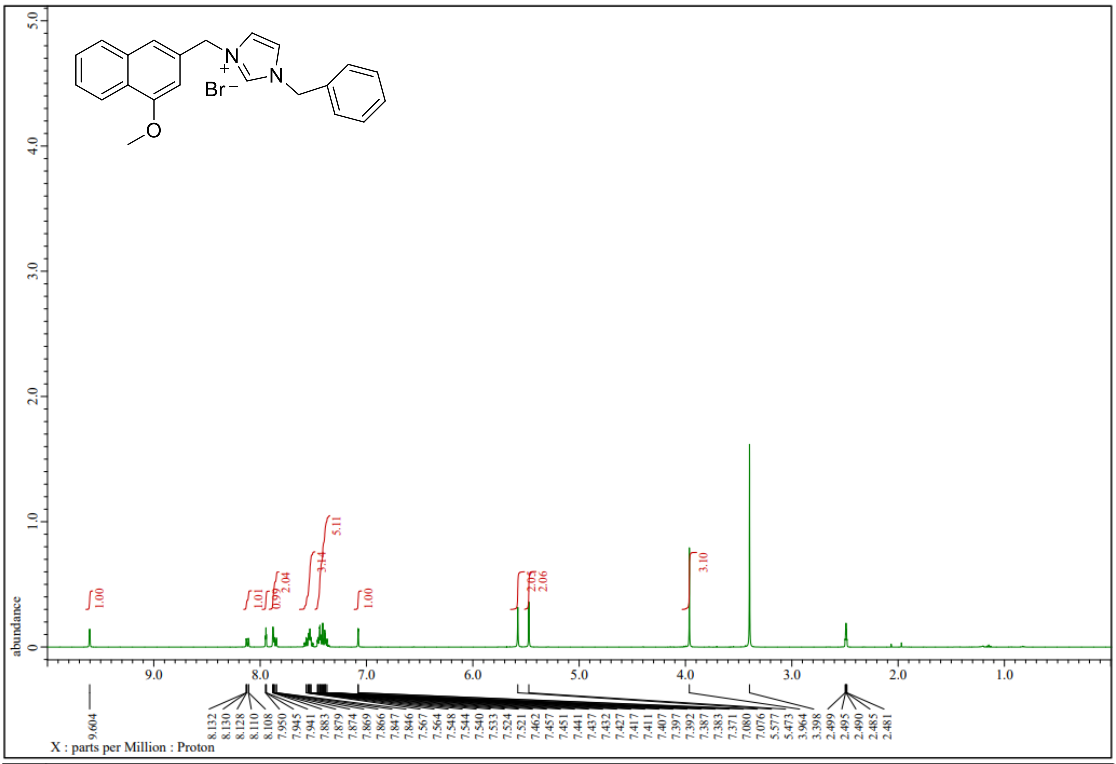

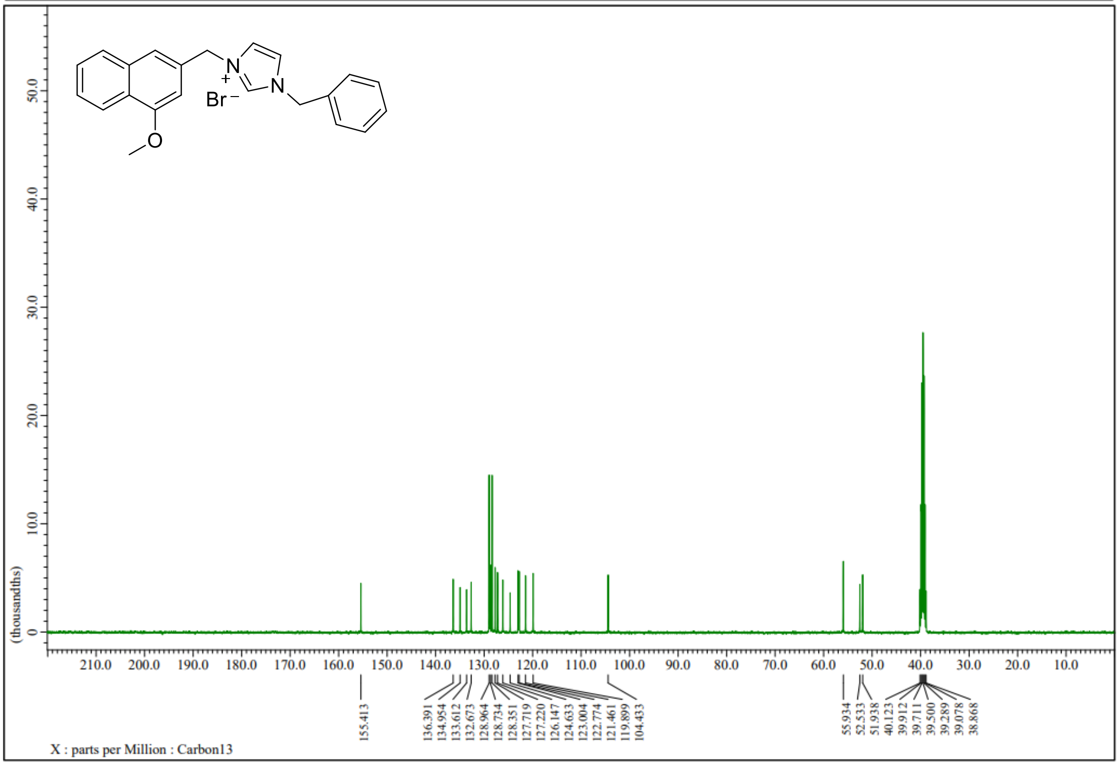

Figure S45. 1-Benzyl-3-((4-methoxynaphthalen-2-yl)methyl)-1*H*-imidazol-3-ium bromide **(IMS-03)**

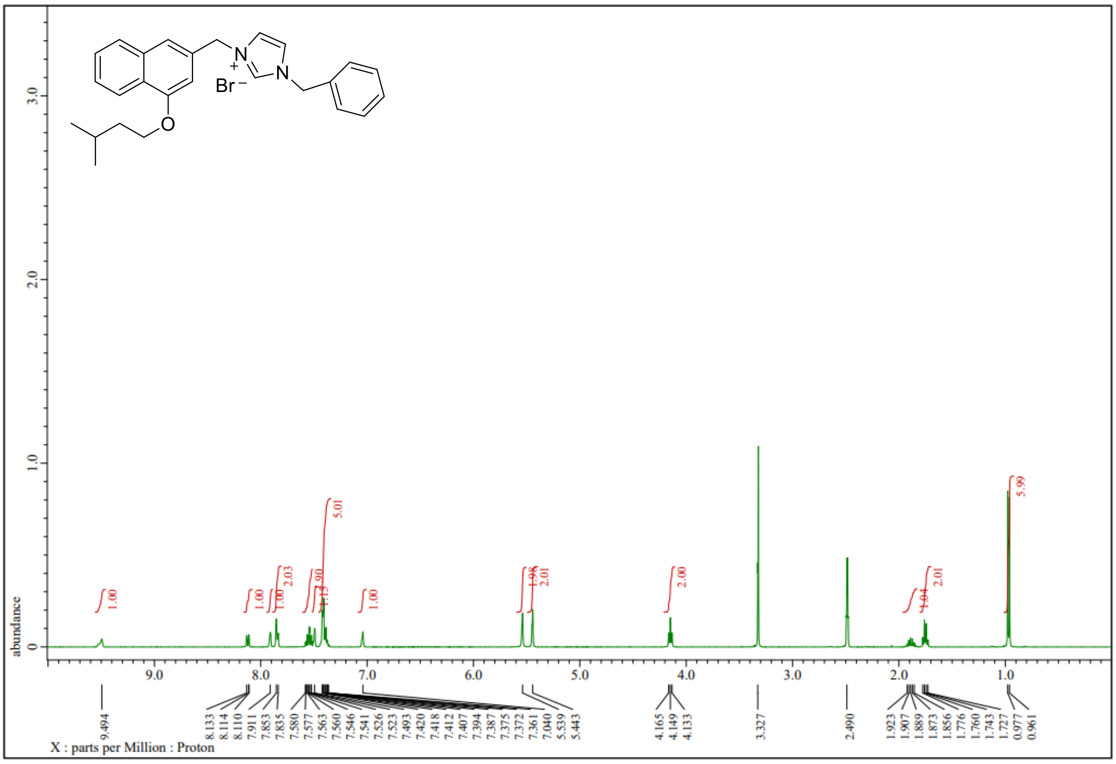

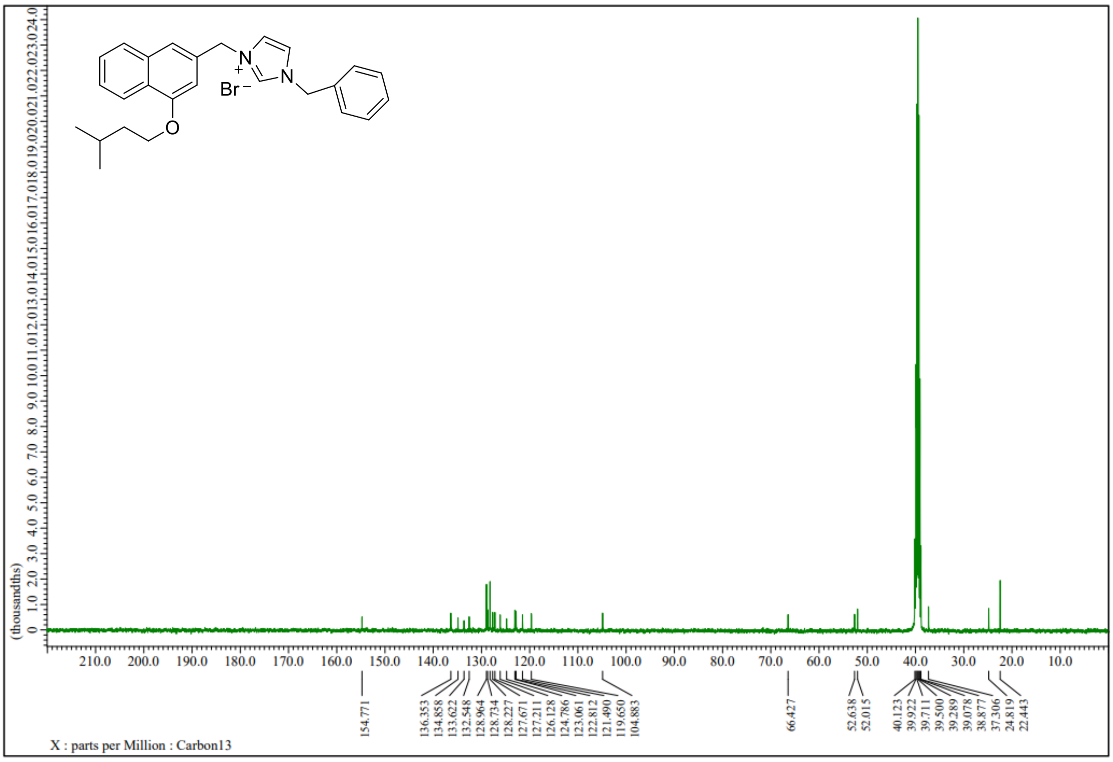

Figure S46. 1-Benzyl-3-((4-(isoamyloxy)naphthalen-2-yl)methyl)-1*H*-imidazol-3-ium bromide **(IMS-04)**

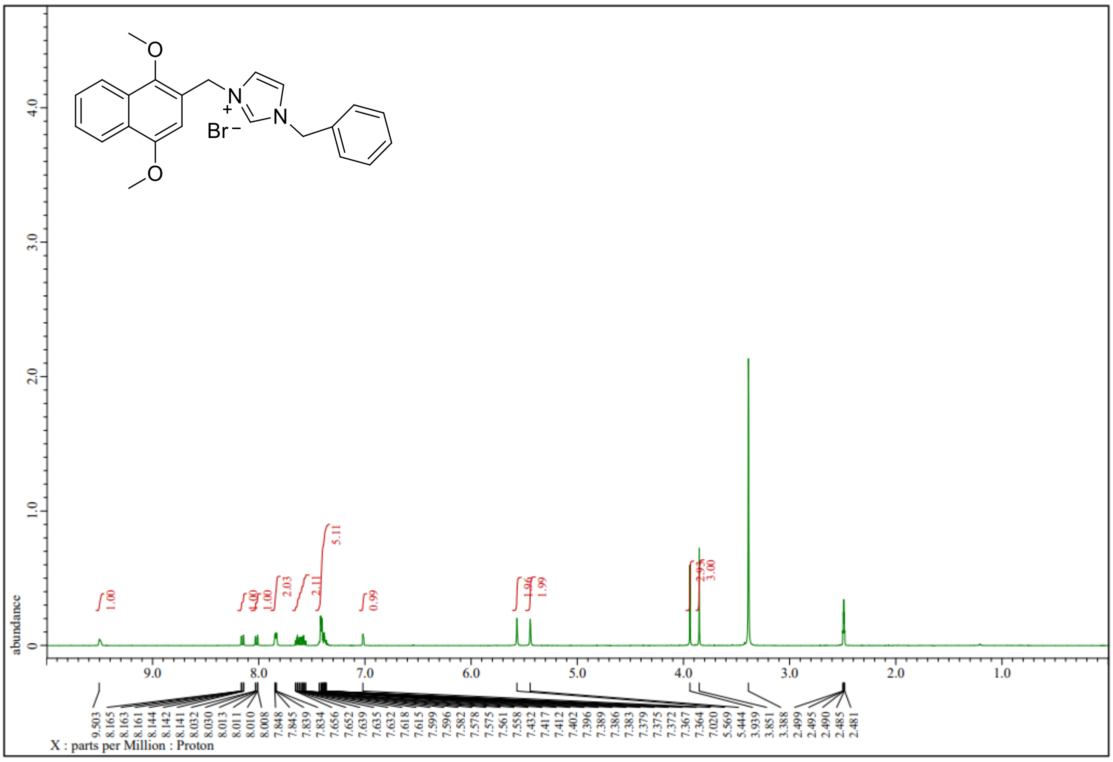

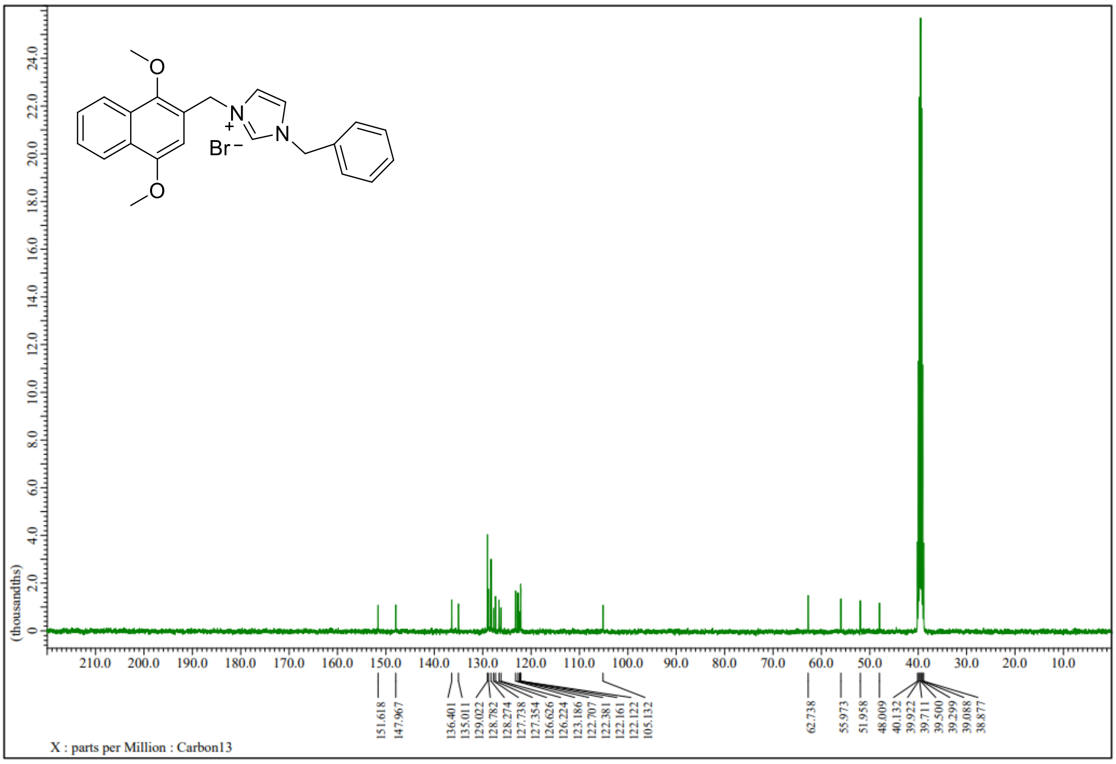

Figure S47. 1-Benzyl-3-((1,4-dimethoxynaphthalen-2-yl)methyl)-1*H*-imidazol-3-ium bromide **(IMS-05)**

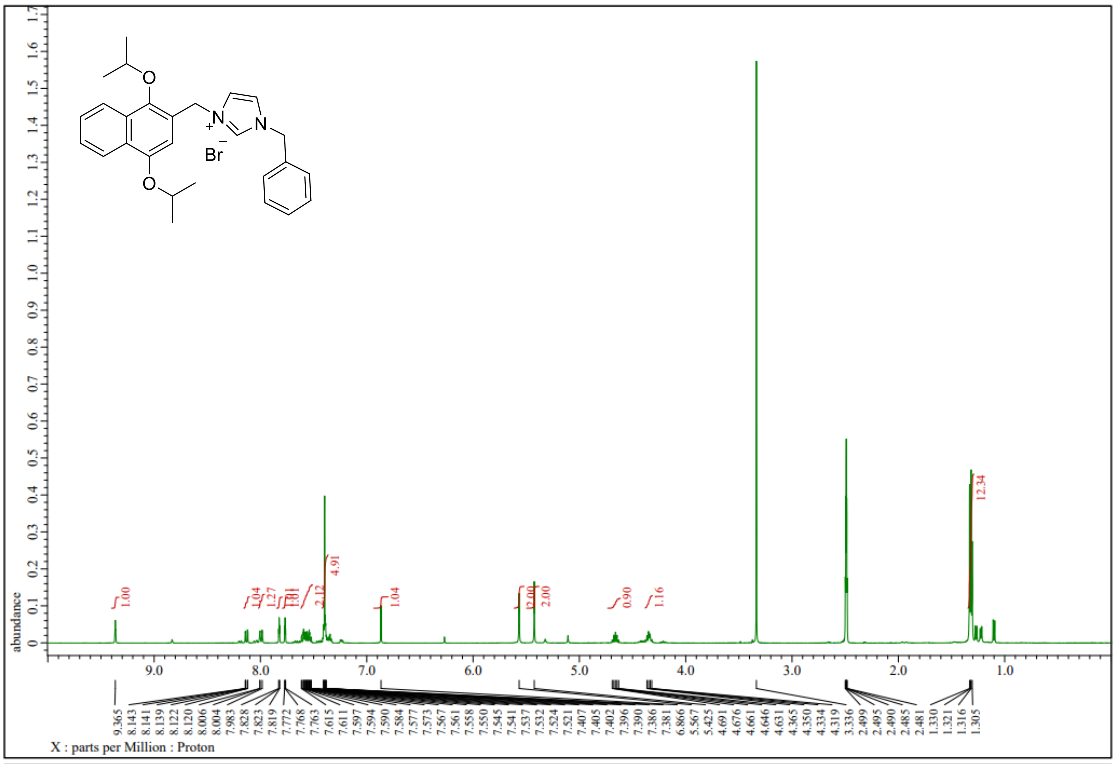

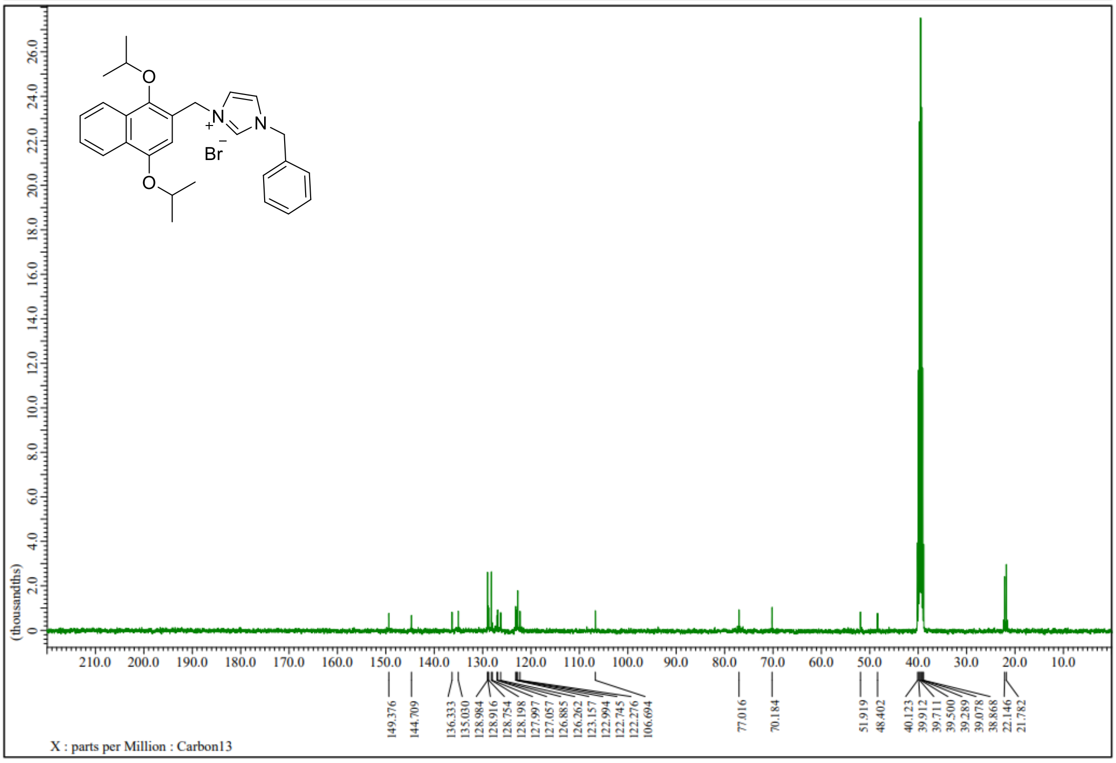

Figure S48. 1-Benzyl-3-((1,4-diisopropoxynaphthalen-2-yl)methyl)-1*H*-imidazol-3-ium bromide **(IMS-06)**

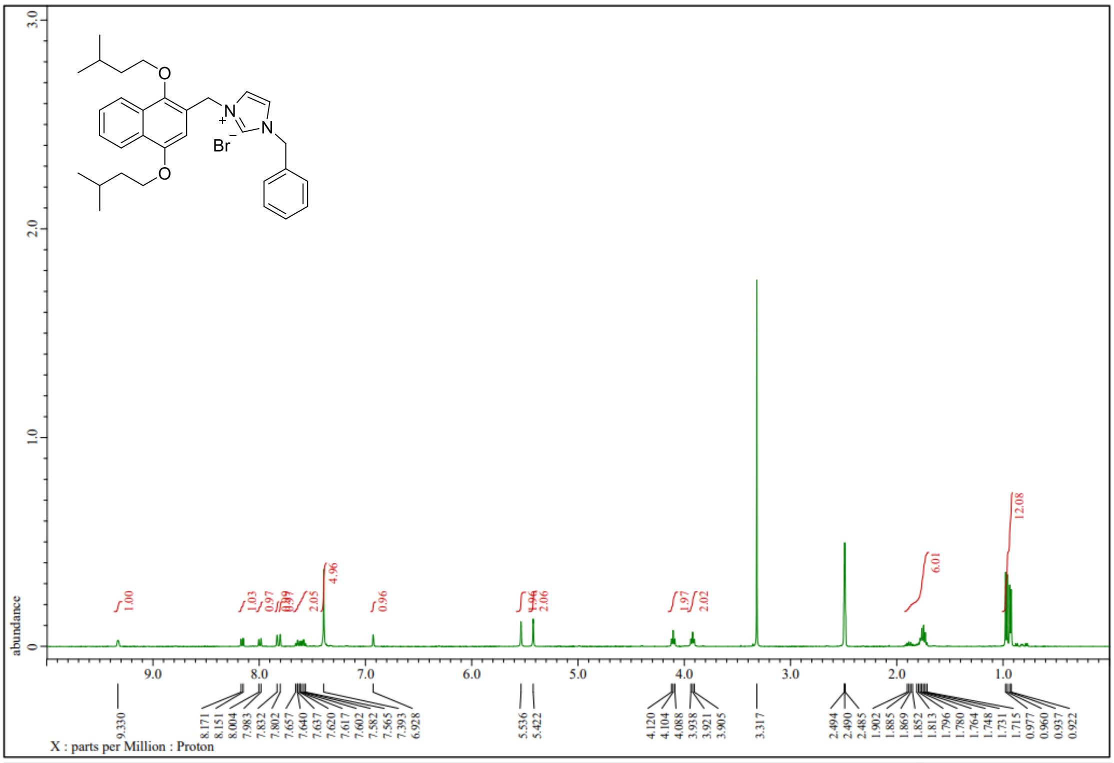

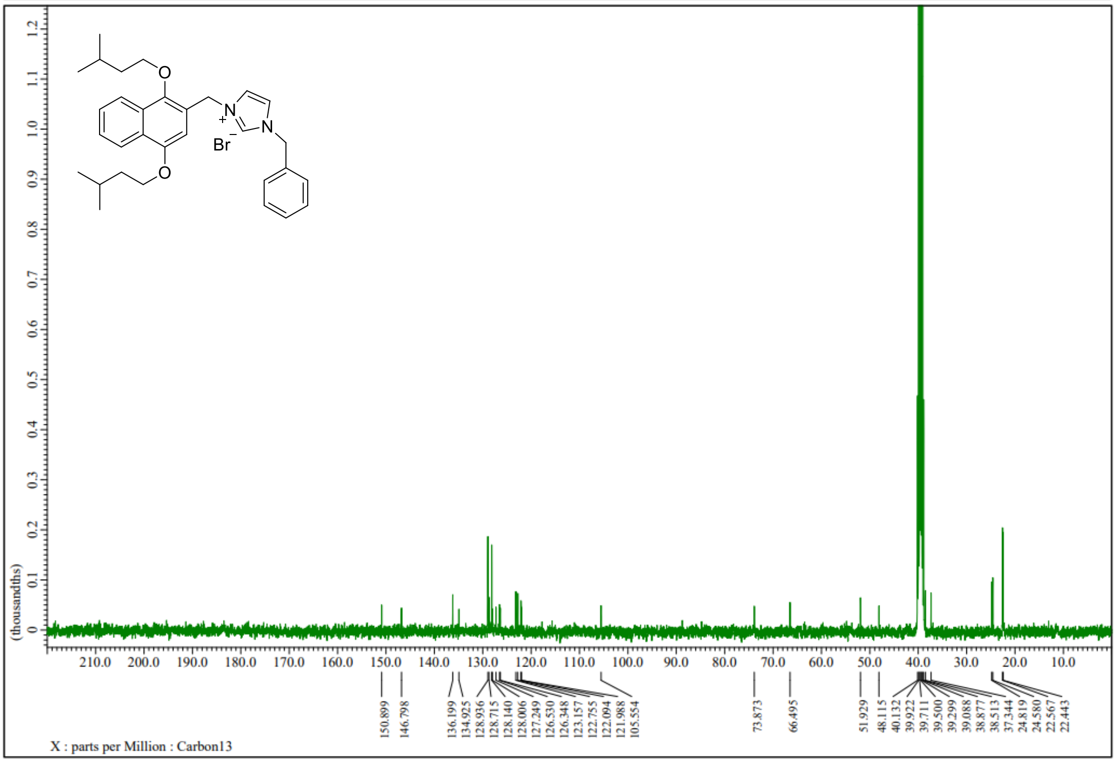

Figure S49. 1-Benzyl-3-((1,4-bis(isoamyloxy)naphthalen-2-yl)methyl)-1*H*-imidazol-3-ium bromide **(IMS-07)**

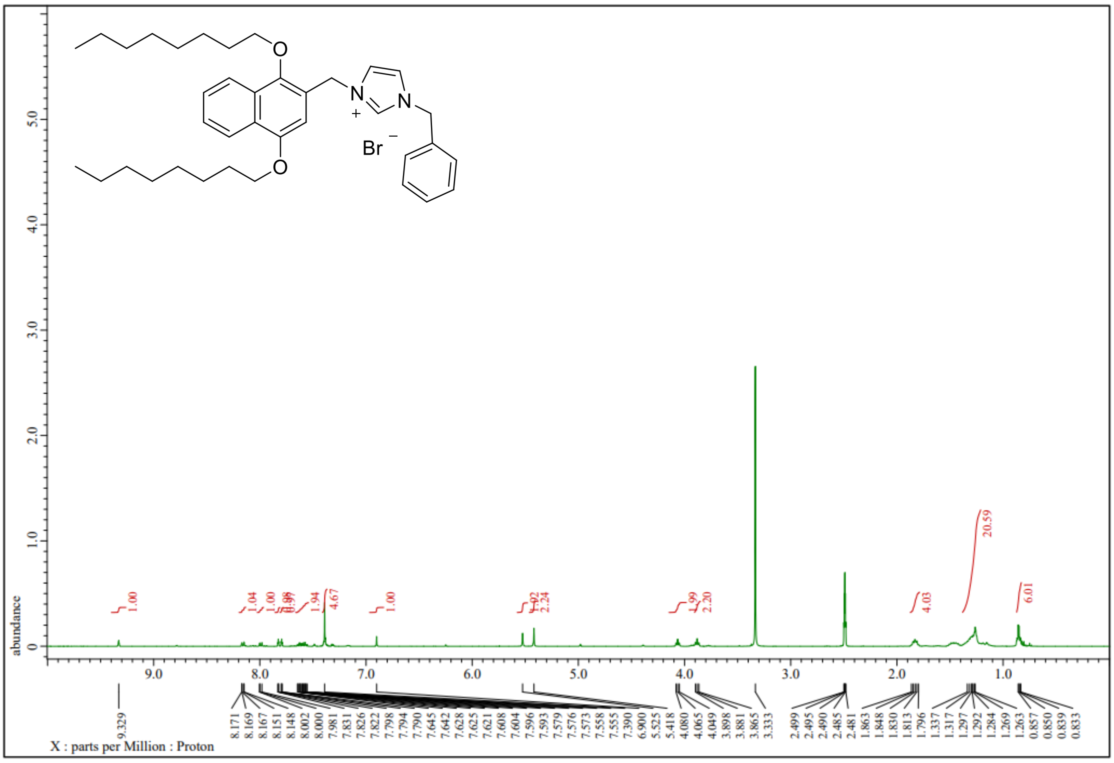

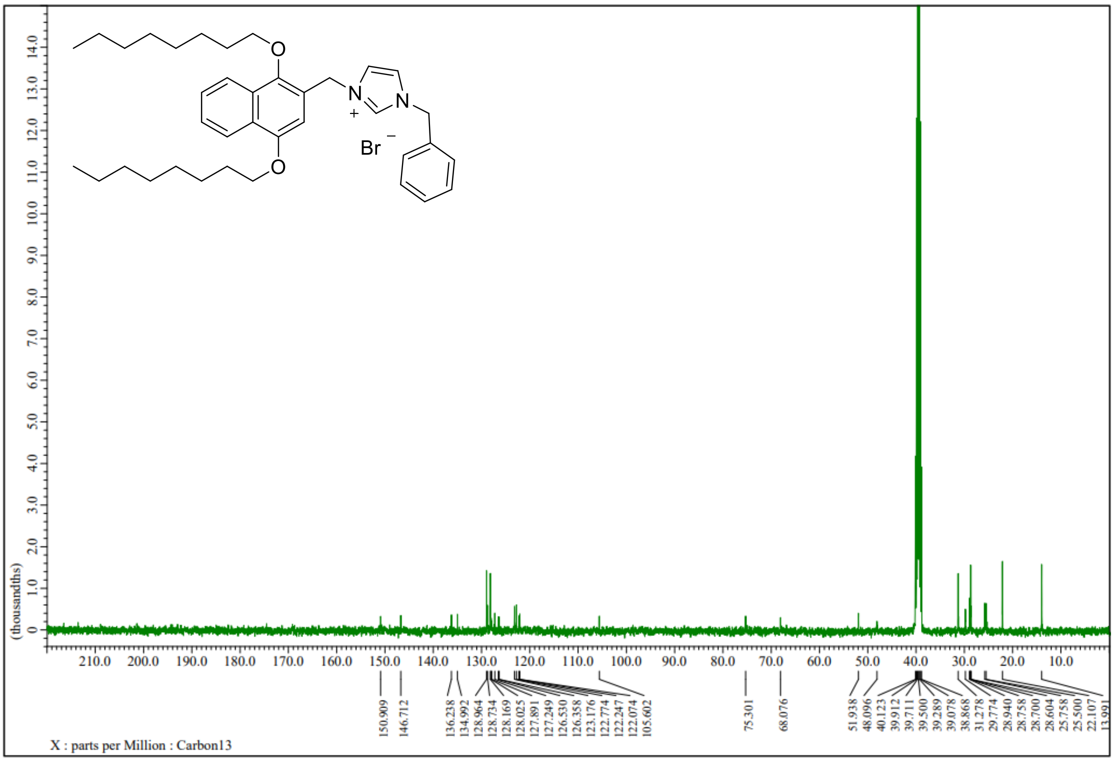

Figure S50. 1-Benzyl-3-((1,4-bis(octyloxy)naphthalen-2-yl)methyl)-1*H*-imidazol-3-ium bromide **(IMS-08)**

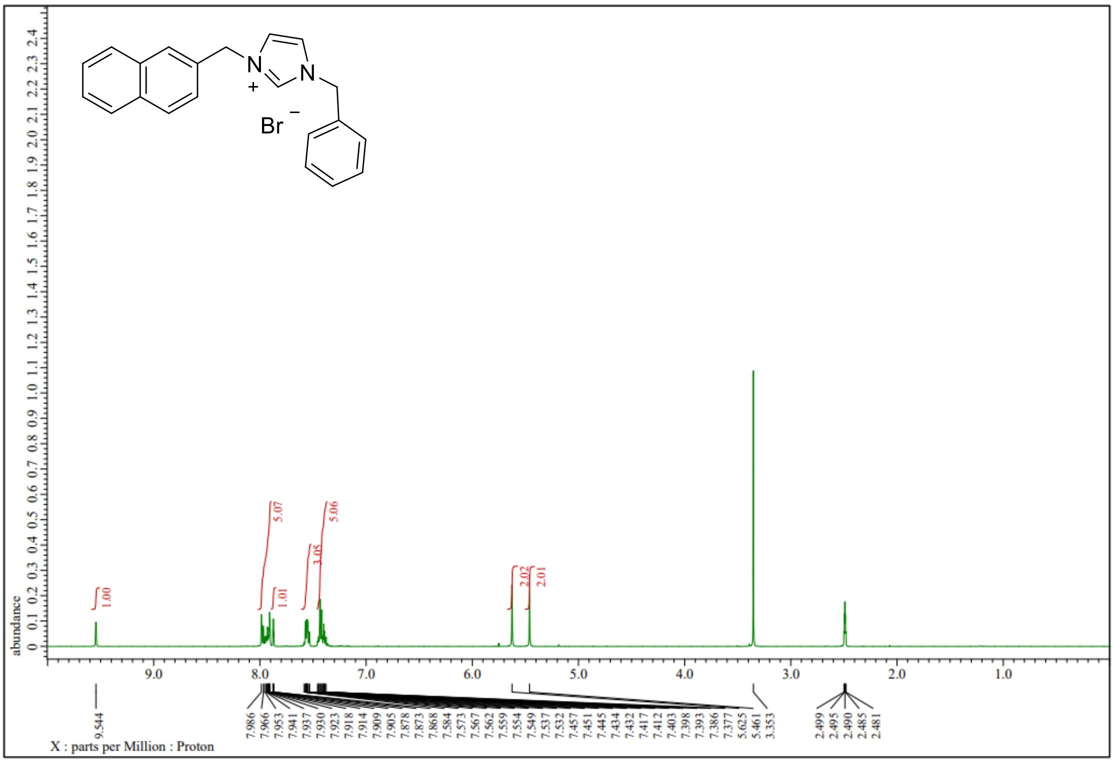

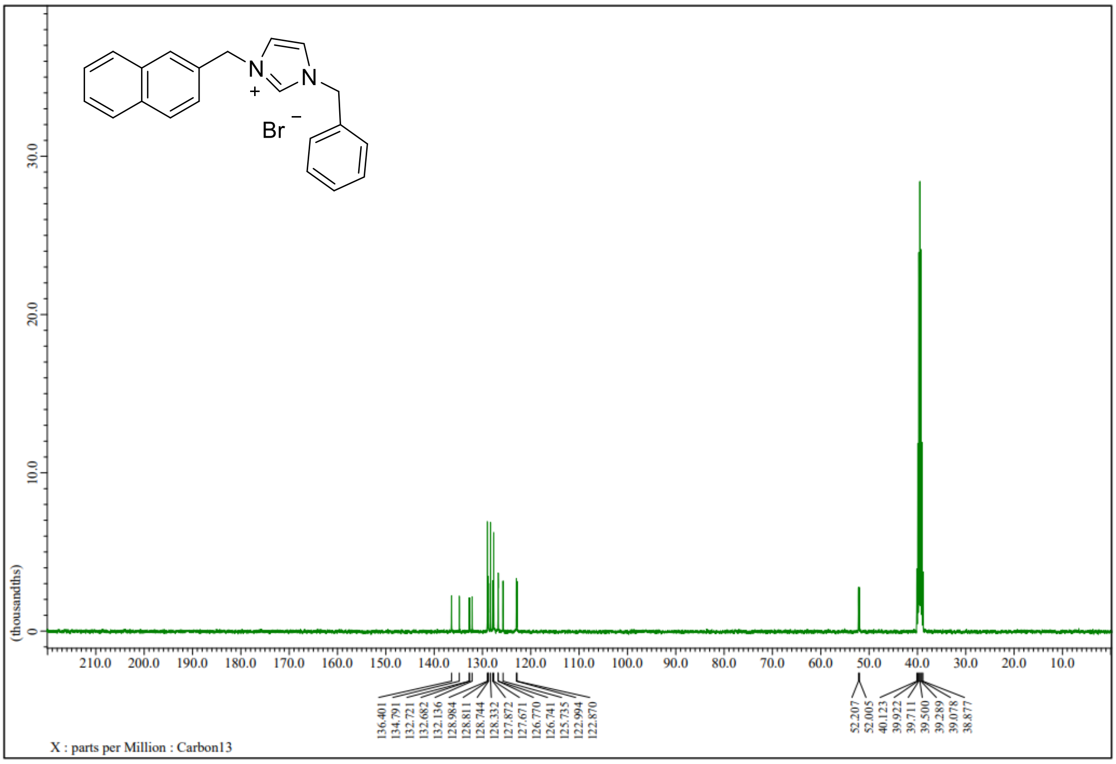

Figure S51. 1-Benzyl-3-(naphthalen-2-ylmethyl)-1*H*-imidazol-3-ium bromide **(IMS-09)**

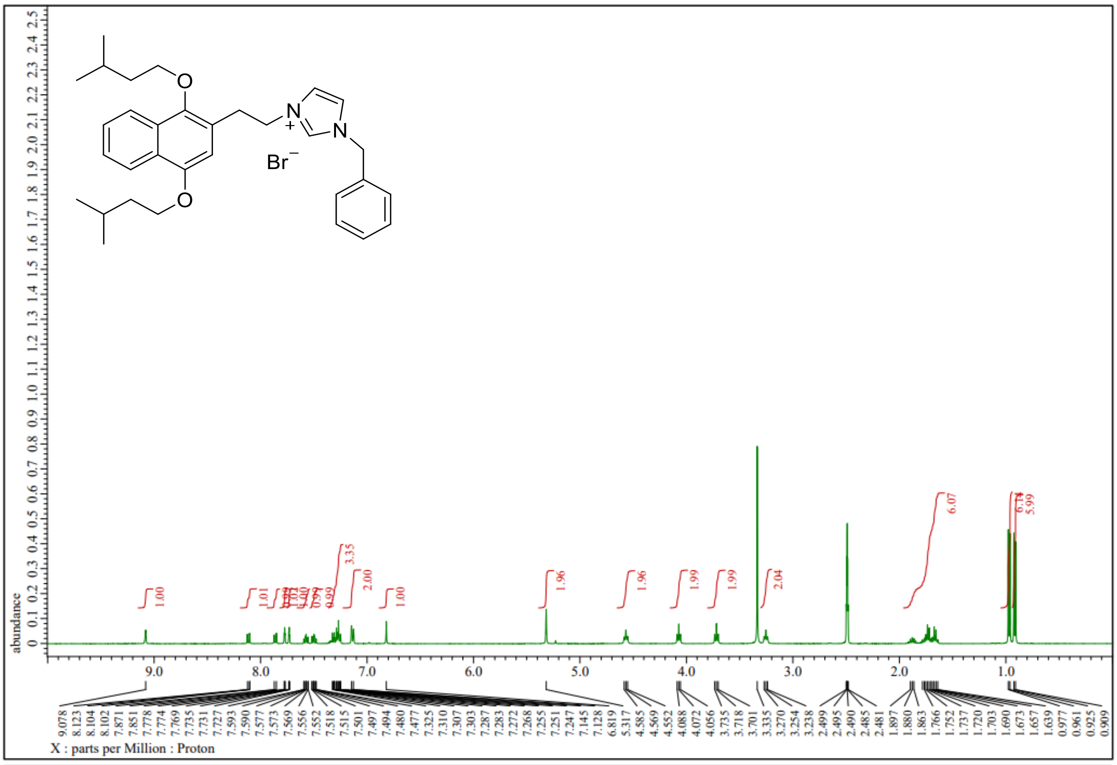

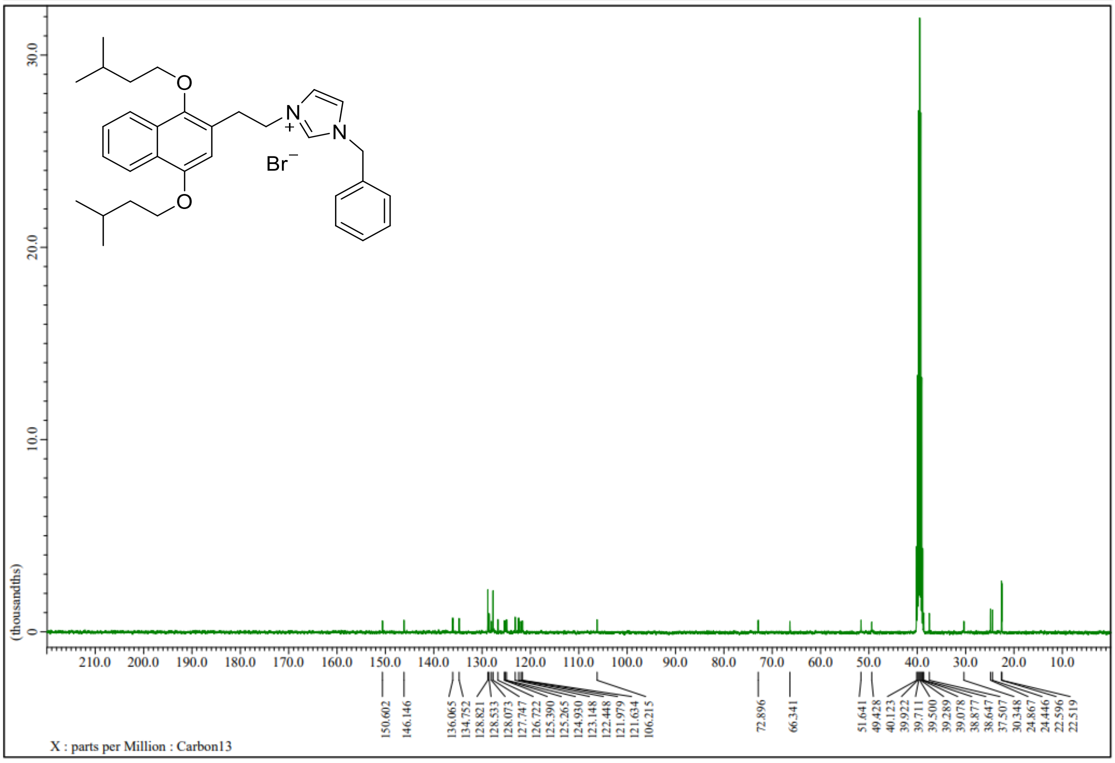

Figure S52. 1-Benzyl-3-(2-(1,4-bis(isoamyloxy)naphthalen-2-yl)ethyl)-1*H*-imidazol-3-ium bromide **(IMS-10)**

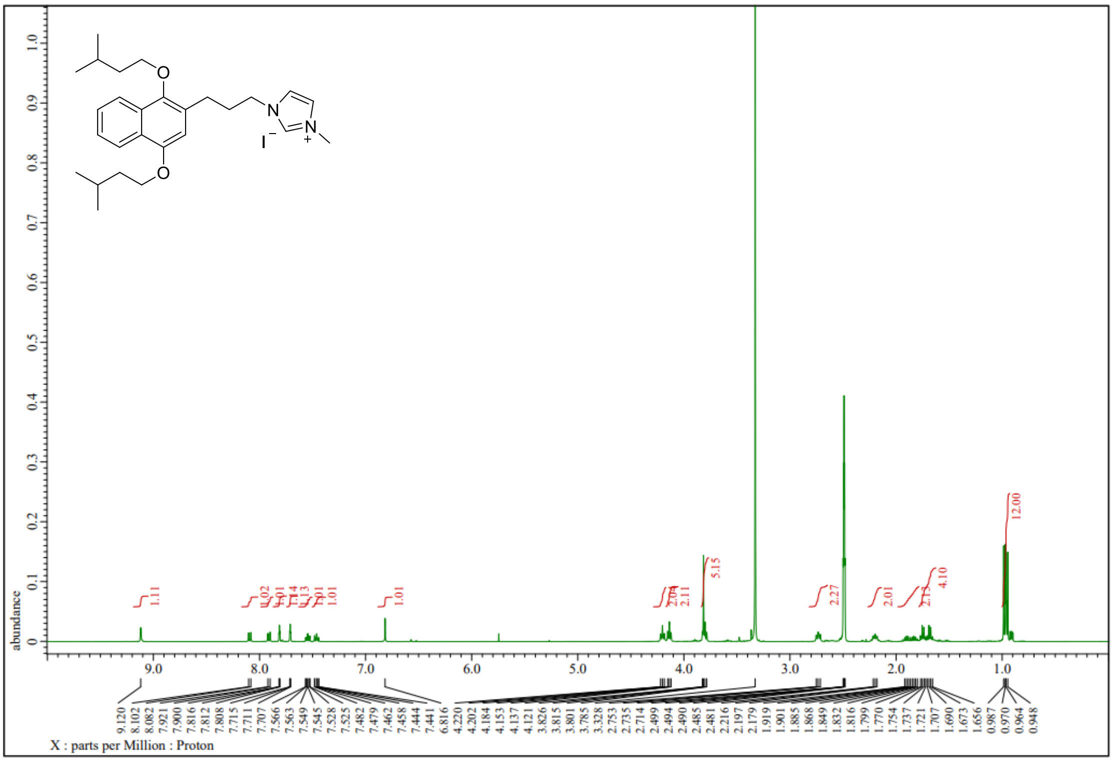

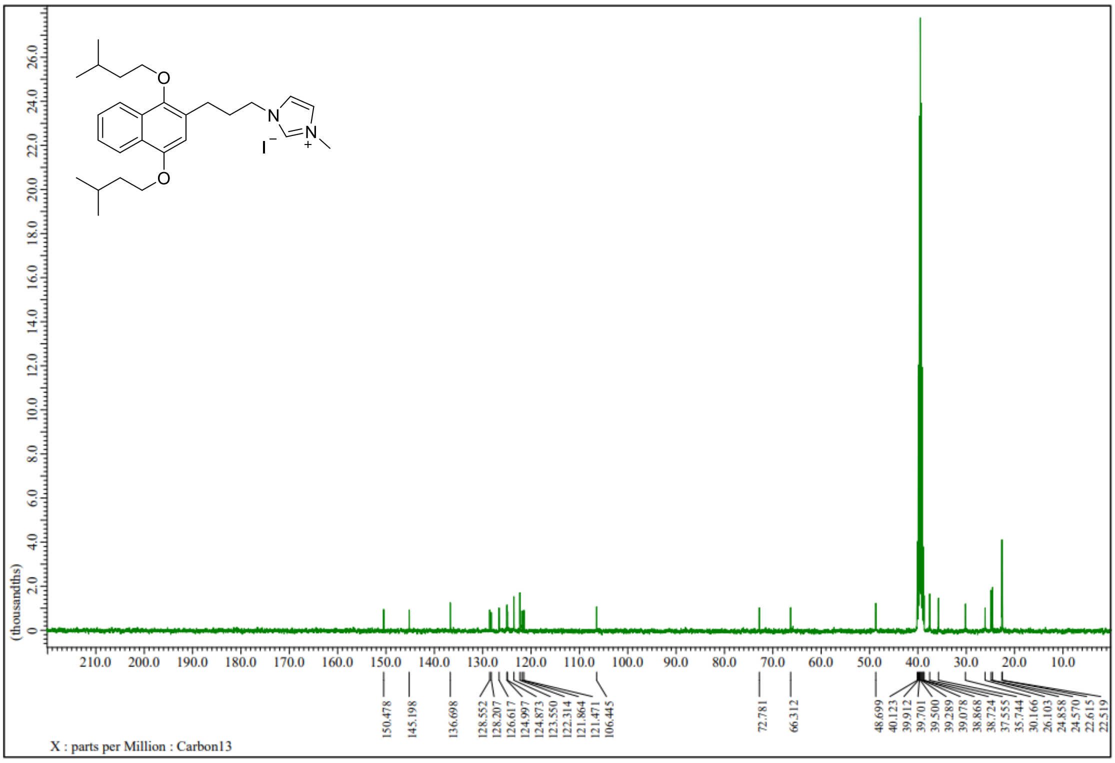

Figure S53. 1-(3-(1,4-Bis(isoamyloxy)naphthalen-2-yl)propyl)-3-methyl-1*H*-imidazol-3-ium iodide **(IMS-11)**

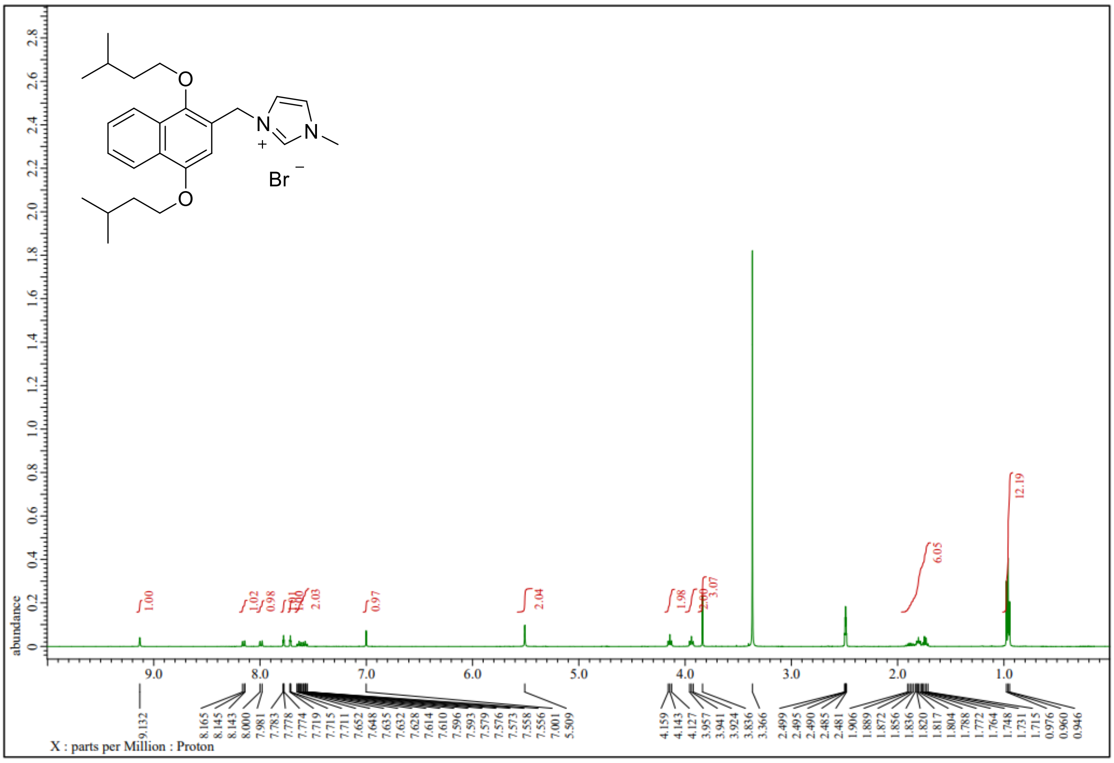

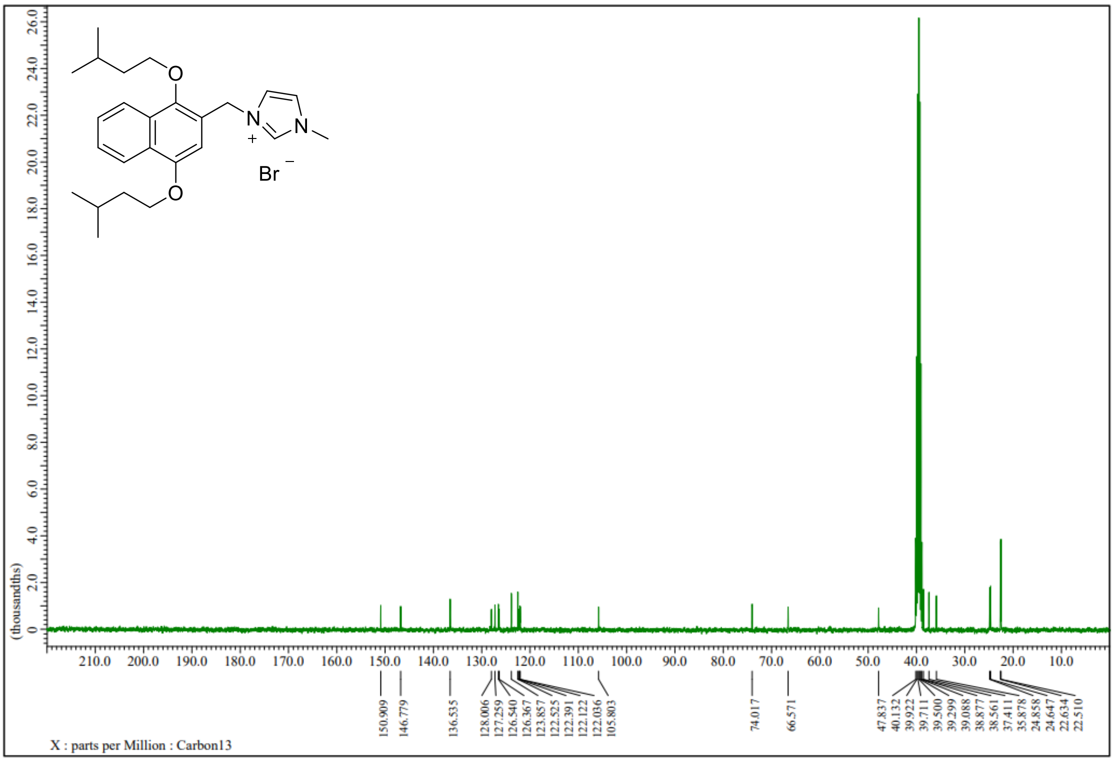

Figure S54. 3-((1,4-Bis(isoamyloxy)naphthalen-2-yl)methyl)-1-methyl-1*H*-imidazol-3-ium bromide **(IMS-12)**

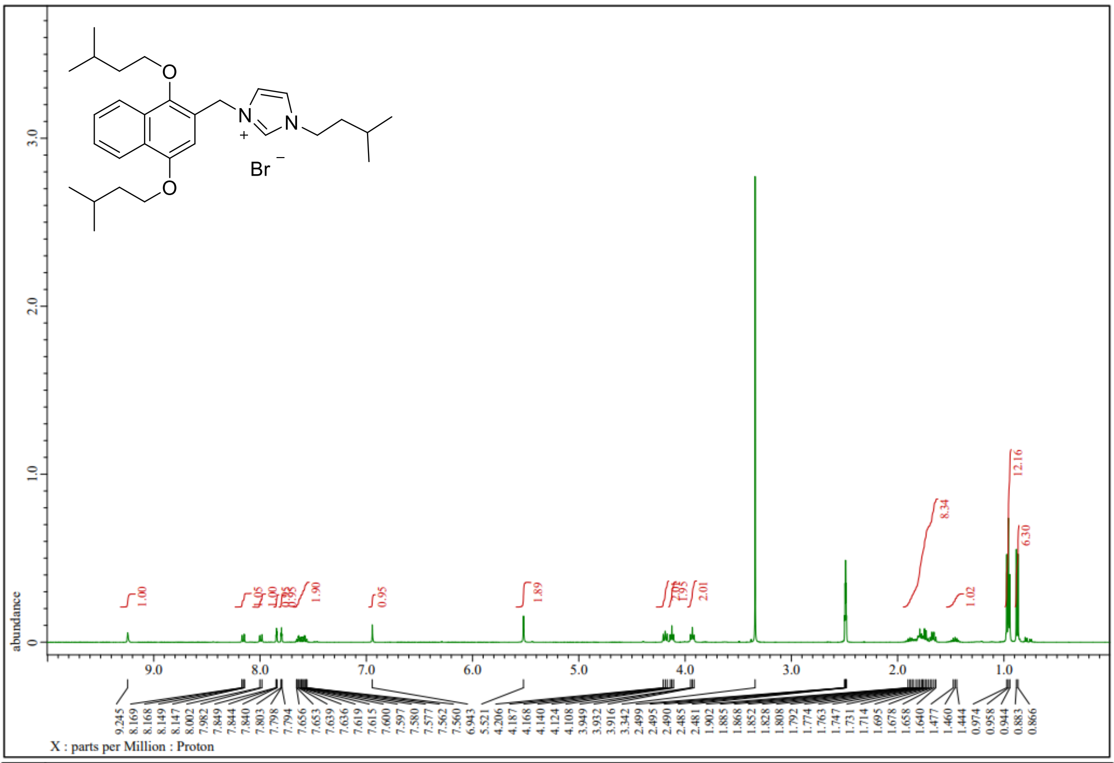

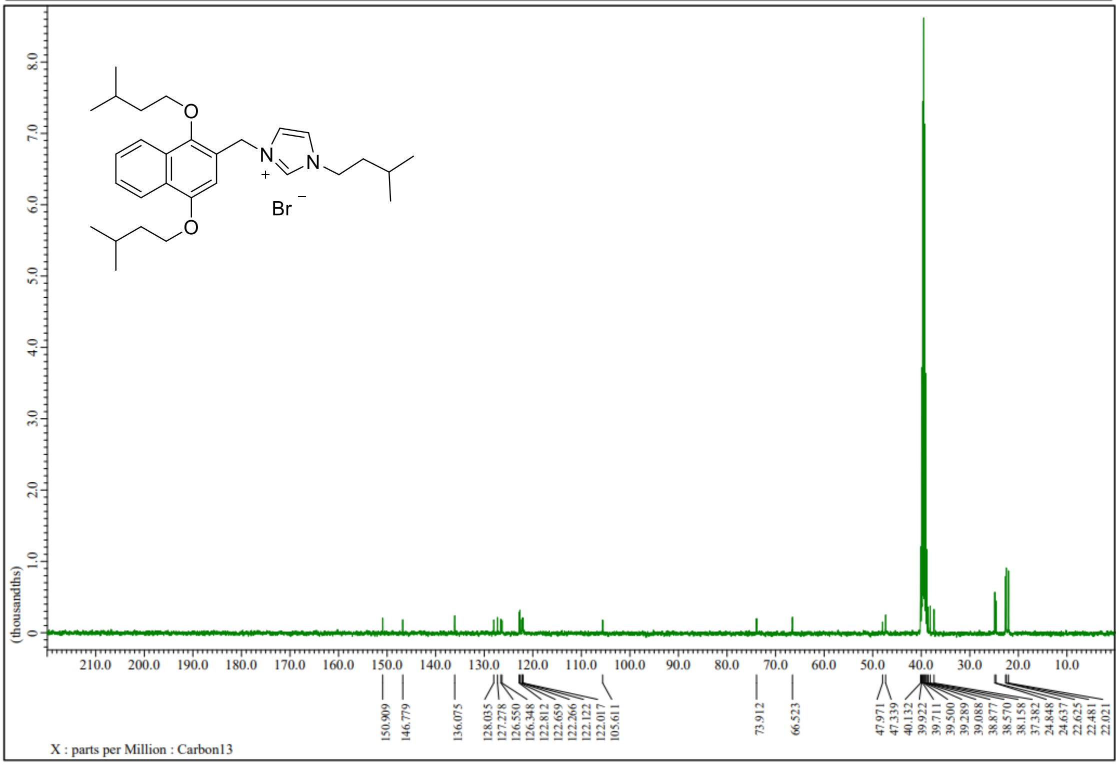

Figure S55. 3-((1,4-Bis(isoamyloxy)naphthalen-2-yl)methyl)-1-isoamyl-1*H*-imidazol-3-ium bromide **(IMS-13)**

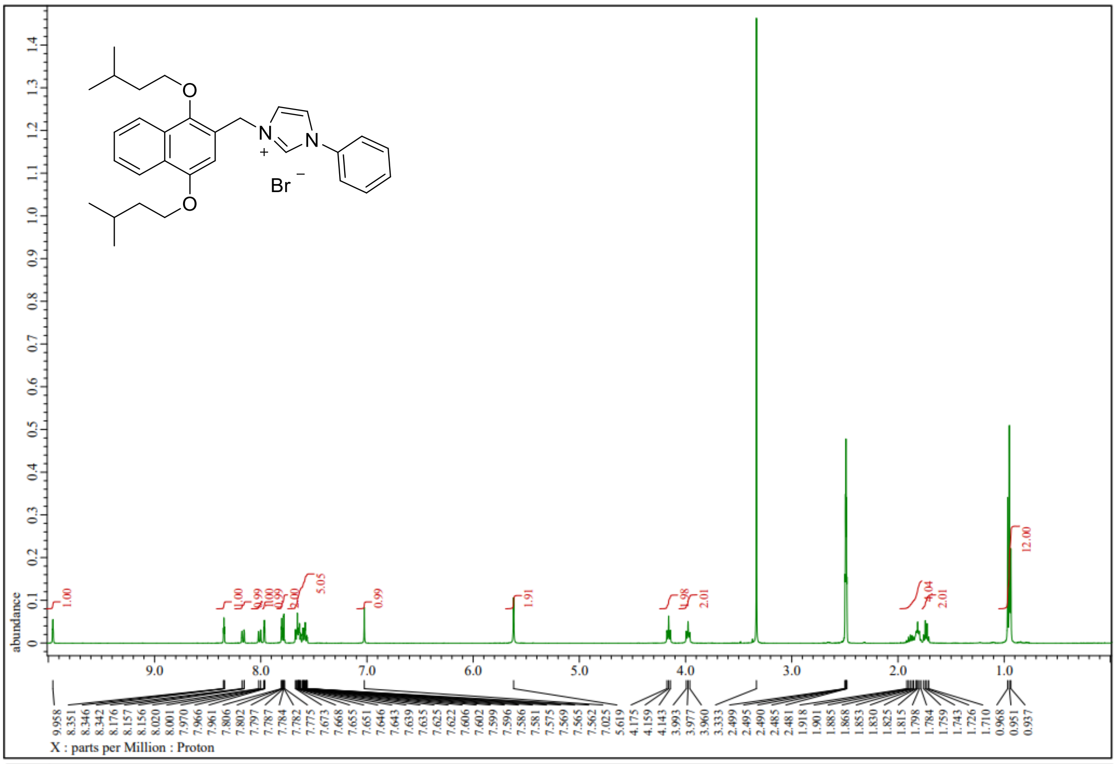

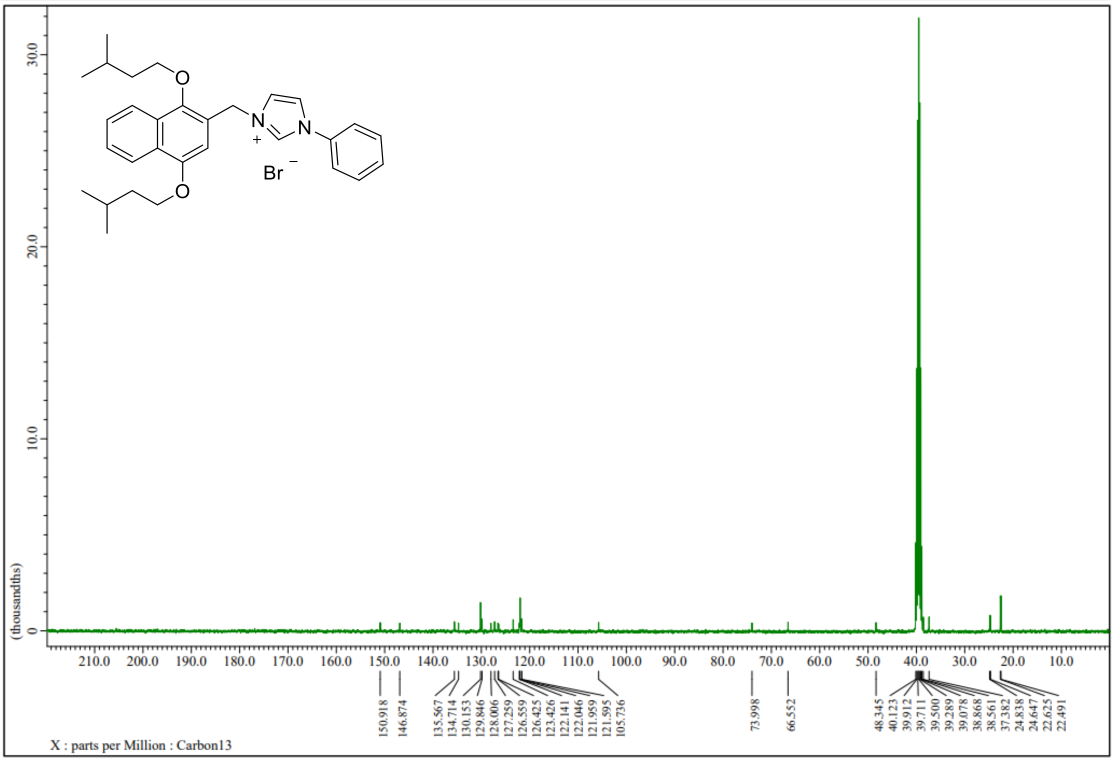

Figure S56. 3-((1,4-Bis(isoamyloxy)naphthalen-2-yl)methyl)-1-phenyl-1*H*-imidazol-3-ium bromide **(IMS-14)**

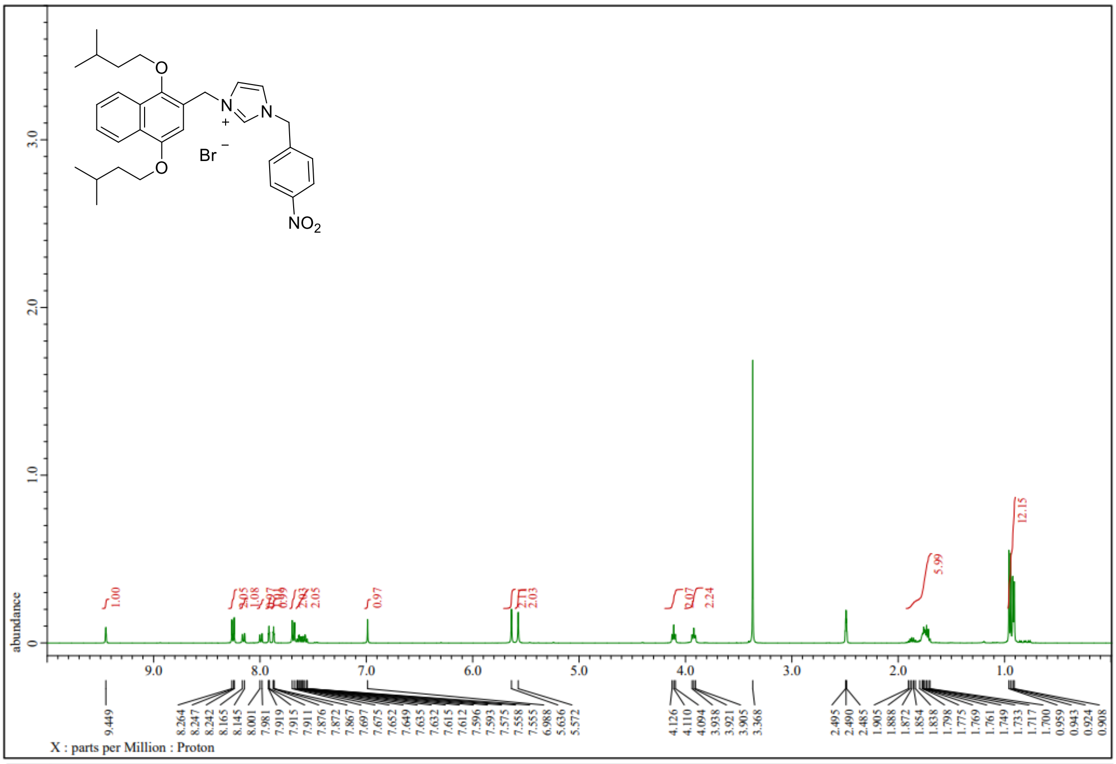

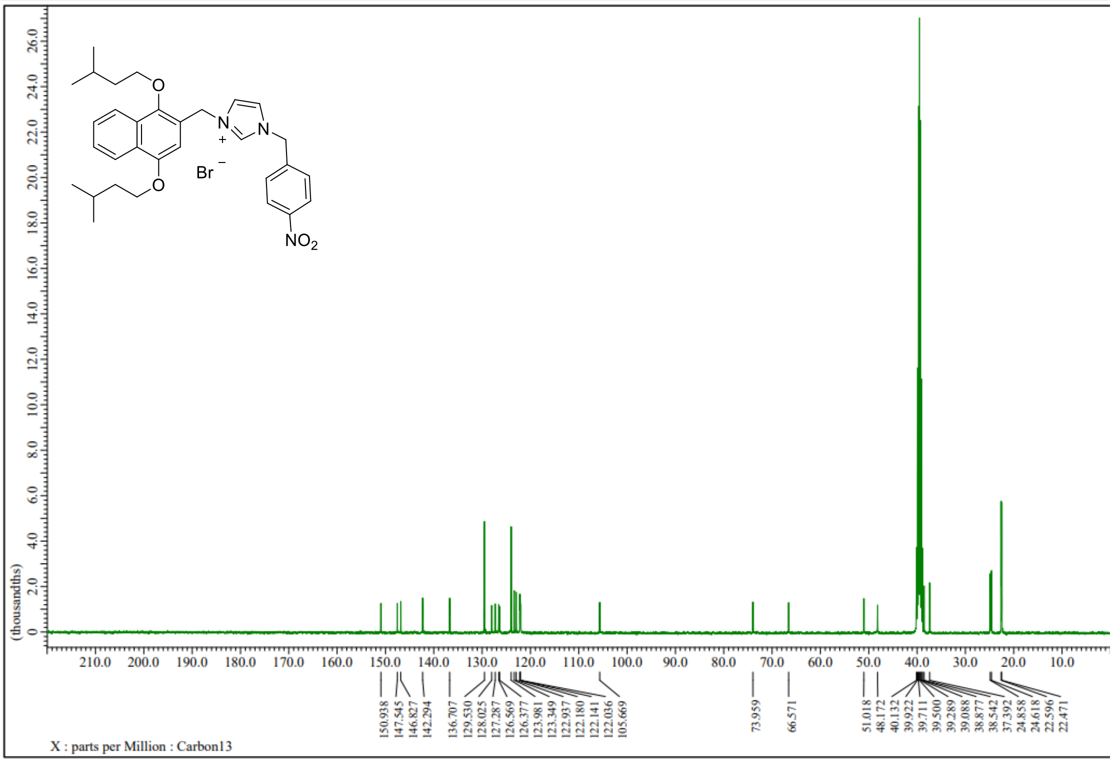

Figure S57. 3-((1,4-Bis(isoamyloxy)naphthalen-2-yl)methyl)-1-(4-nitrobenzyl)-1*H*-imidazol-3-ium bromide **(IMS-15)**

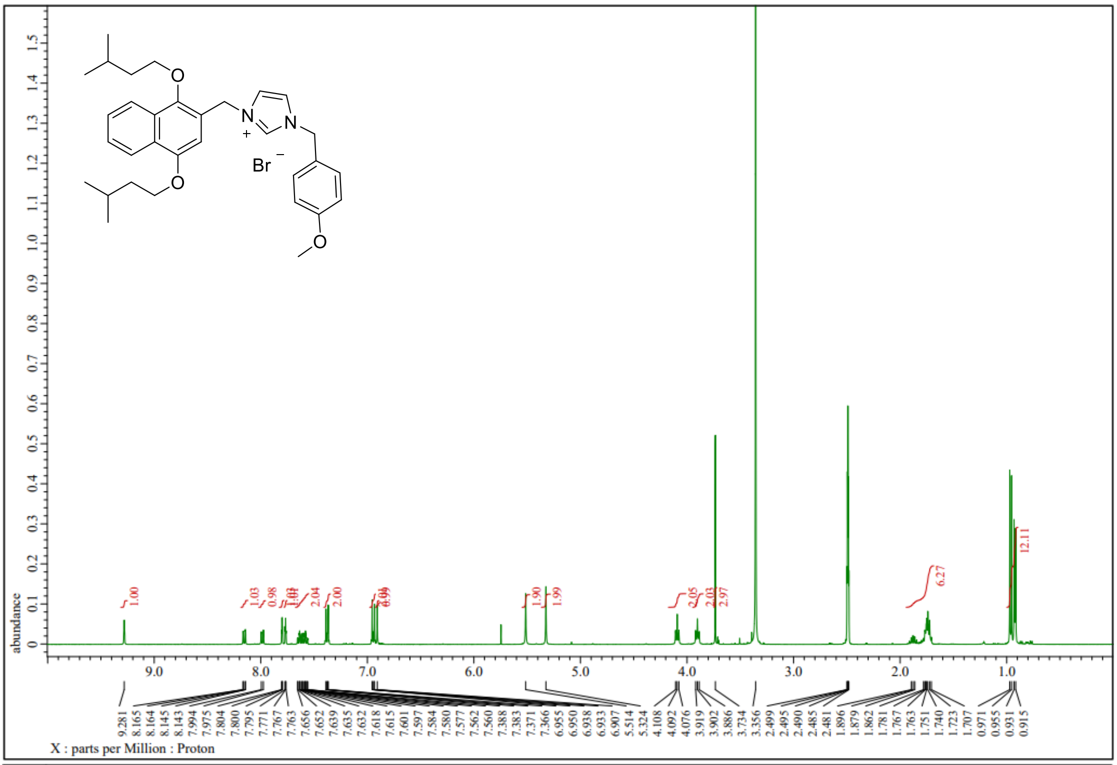

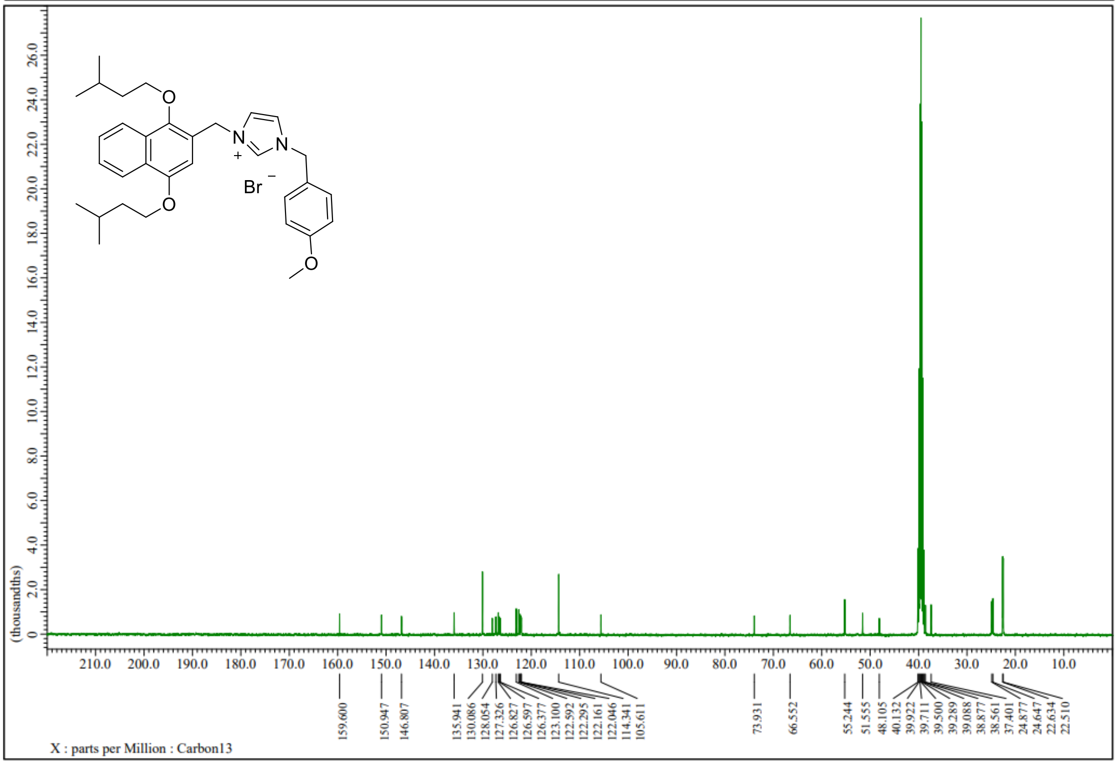

Figure S58. 3-((1,4-Bis(isoamyloxy)naphthalen-2-yl)methyl)-1-(4-methoxybenzyl)-1*H*-imidazol-3-ium bromide **(IMS-16)**

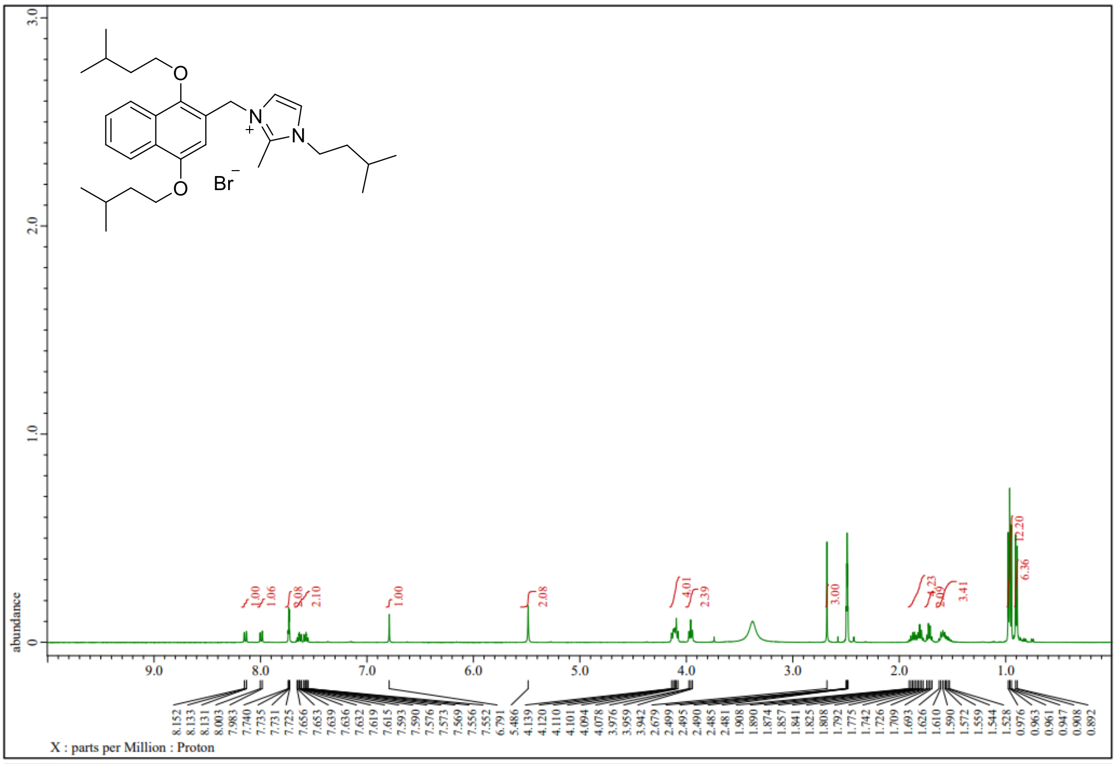

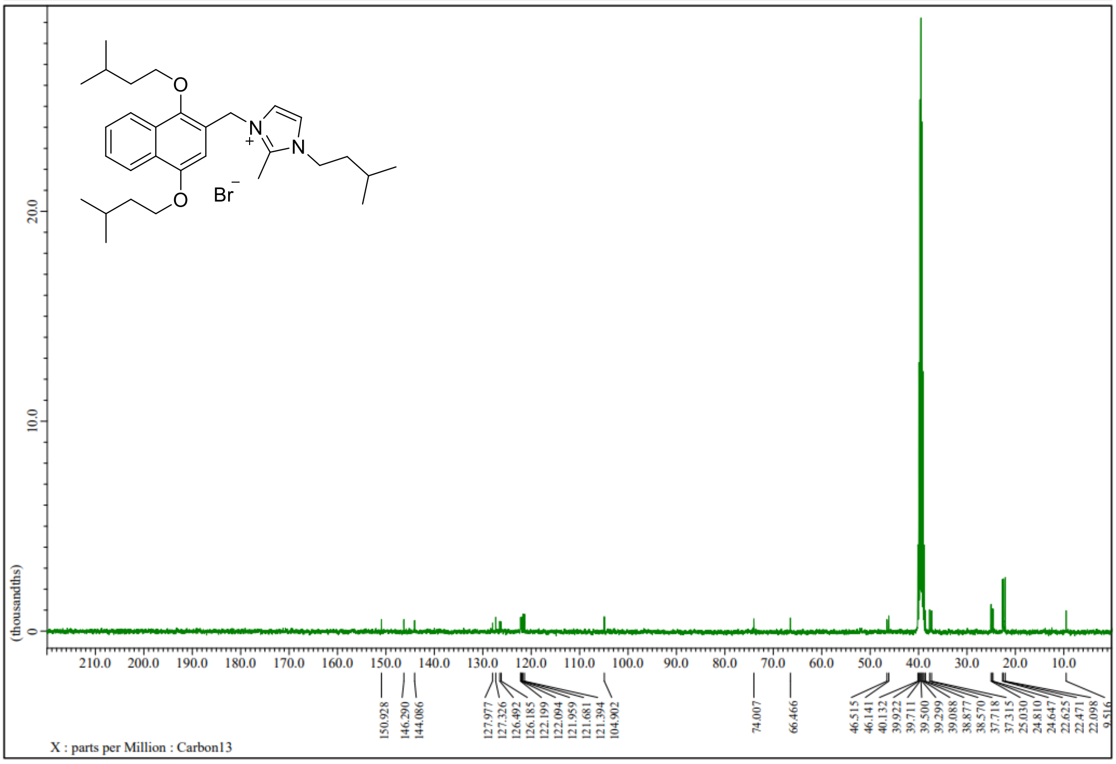

Figure S59. 3-((1,4-Bis(isoamyloxy)naphthalen-2-yl)methyl)-1-isoamyl-2-methyl-1*H*-imidazol-3-ium bromide **IMS-17)**

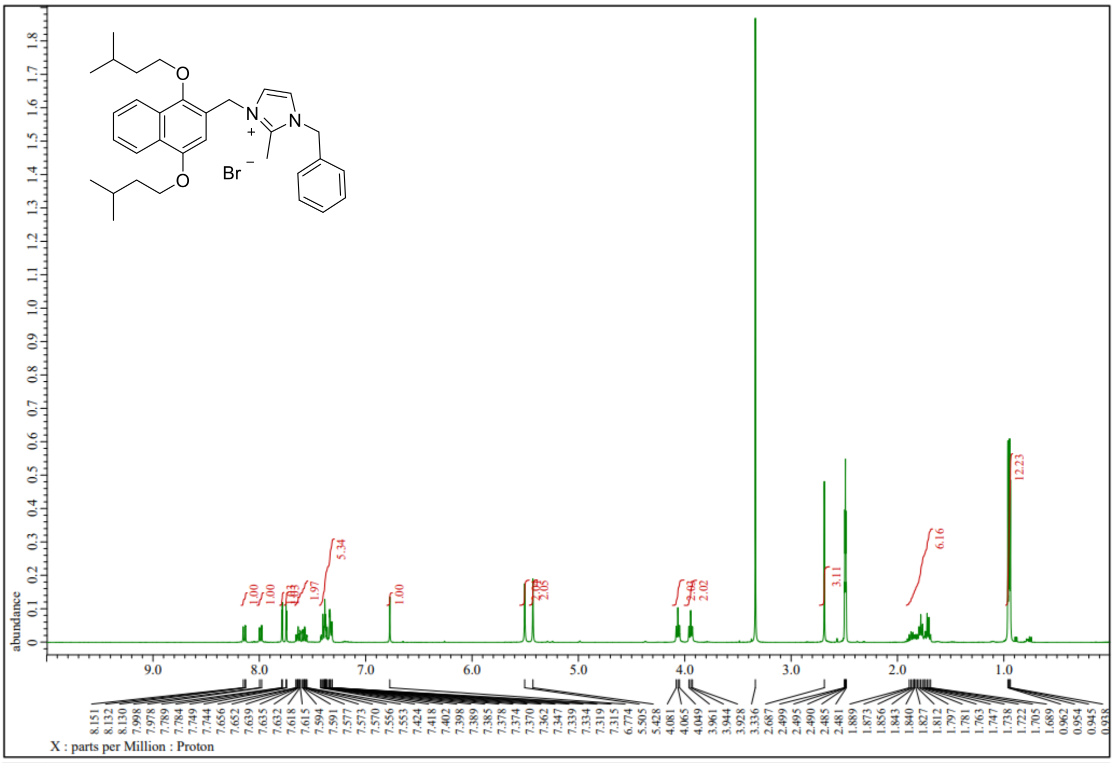

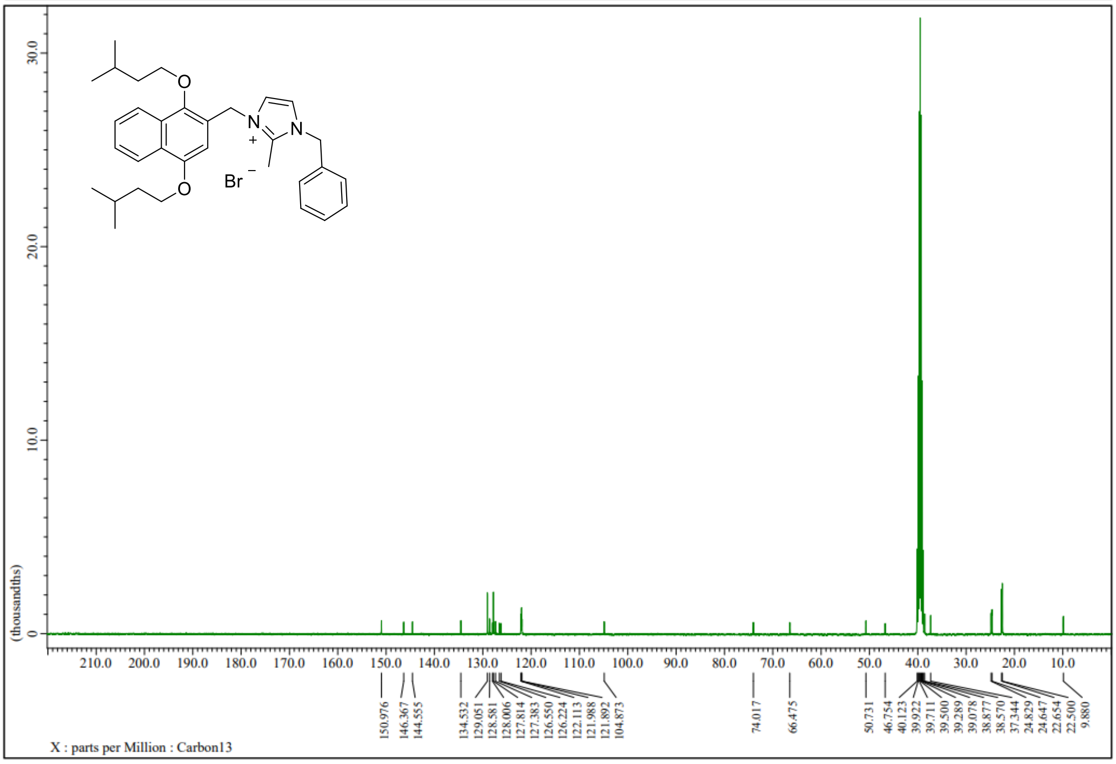

Figure S60. 1-Benzyl-3-((1,4-bis(isoamyloxy)naphthalen-2-yl)methyl)-2-methyl-1*H*-imidazol-3-ium bromide **(IMS-18)**

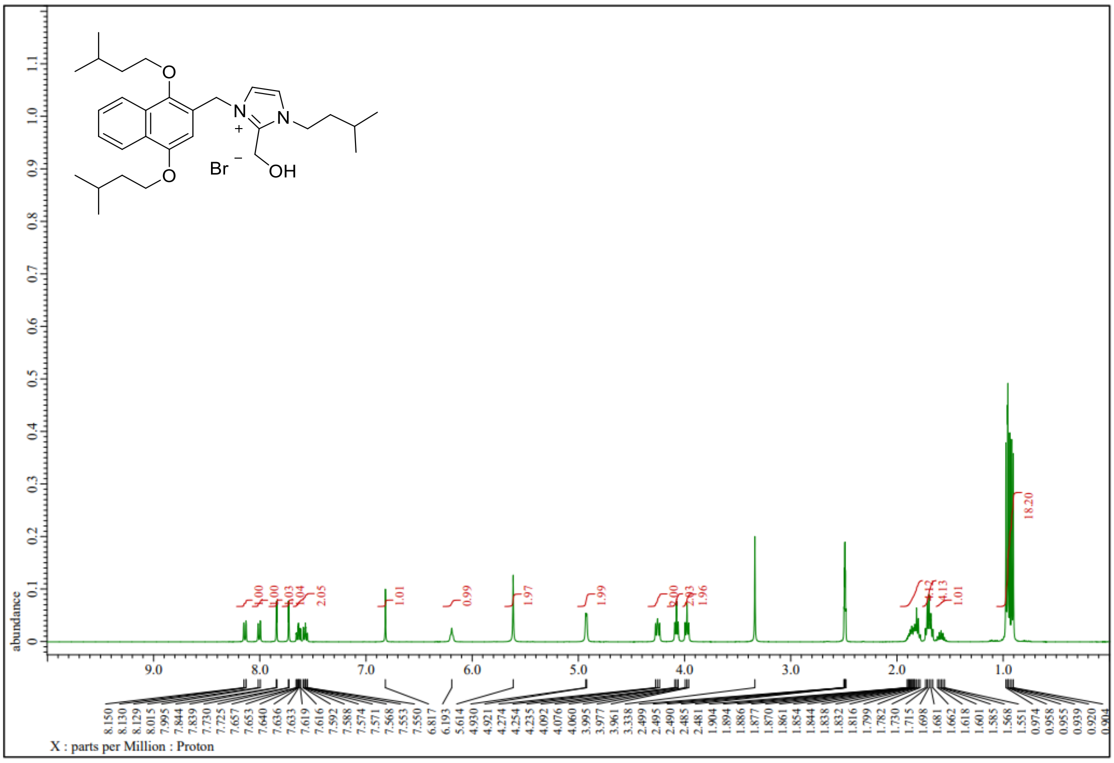

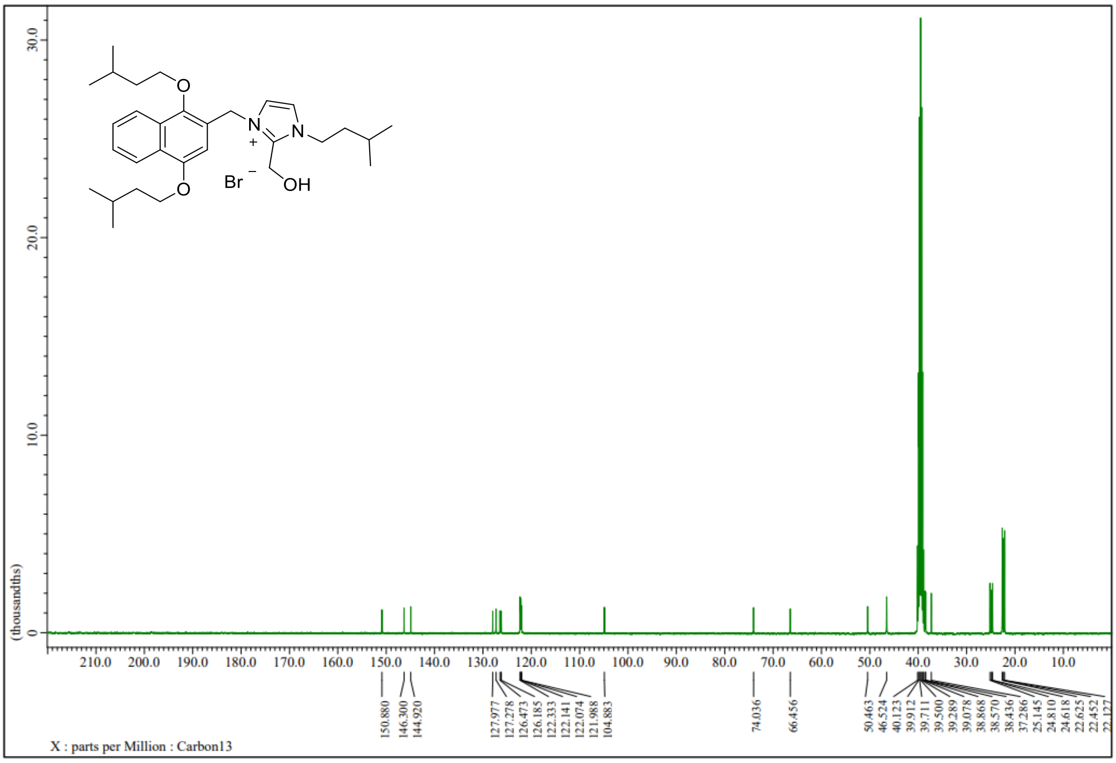

Figure S61. 3-((1,4-Bis(isoamyloxy)naphthalen-2-yl)methyl)-2-(hydroxymethyl)-1-isoamyl-1*H*-imidazol-3-ium bromide **(IMS-19)**

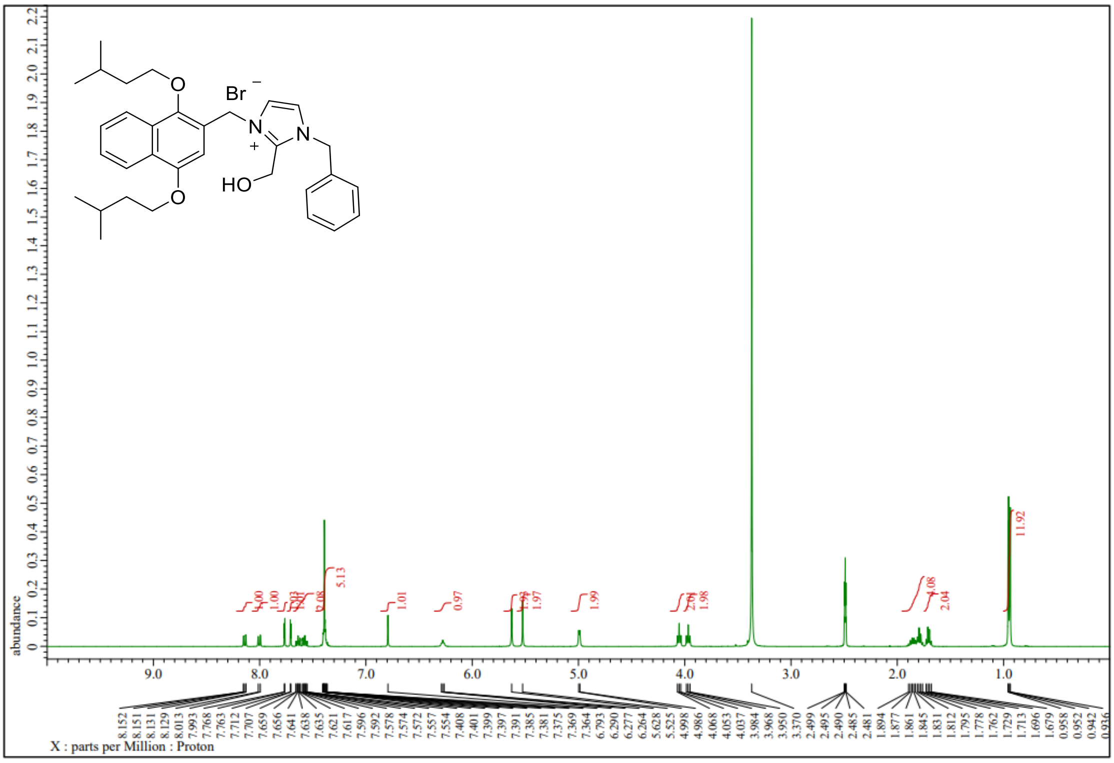

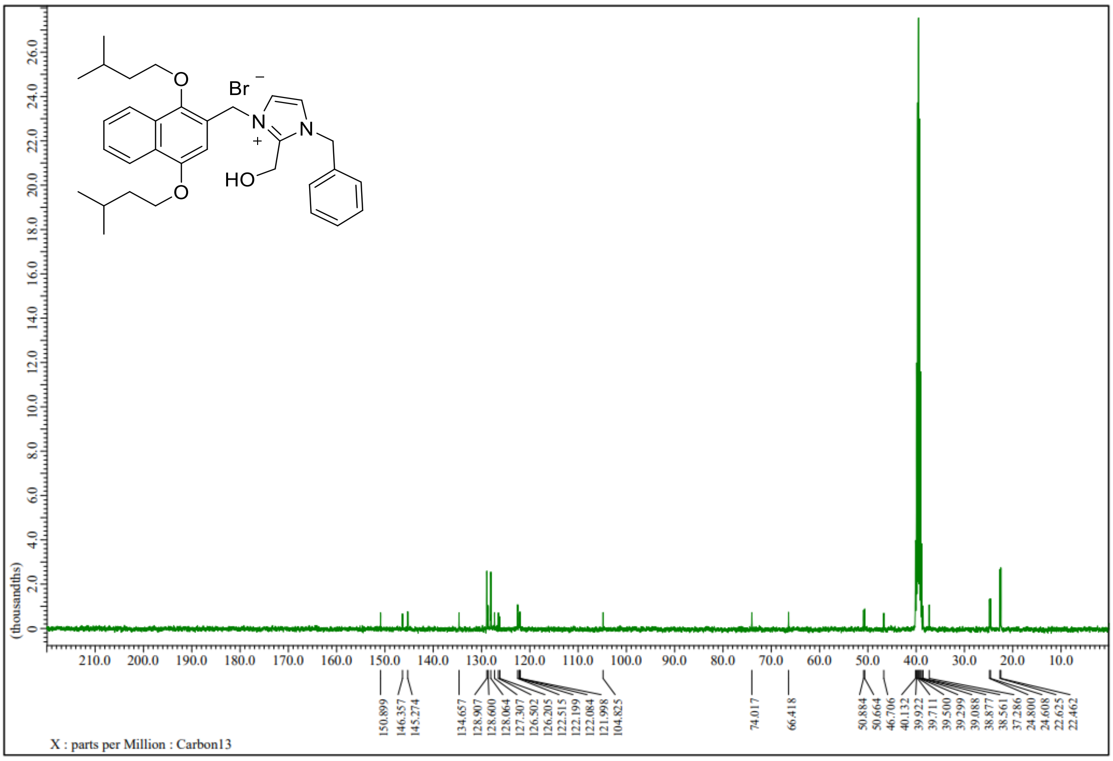

Figure S62. 1-Benzyl-3-((1,4-bis(isoamyloxy)naphthalen-2-yl)methyl)-2-(hydroxymethyl)-1*H*-imidazol-3-ium bromide **(IMS-20)**

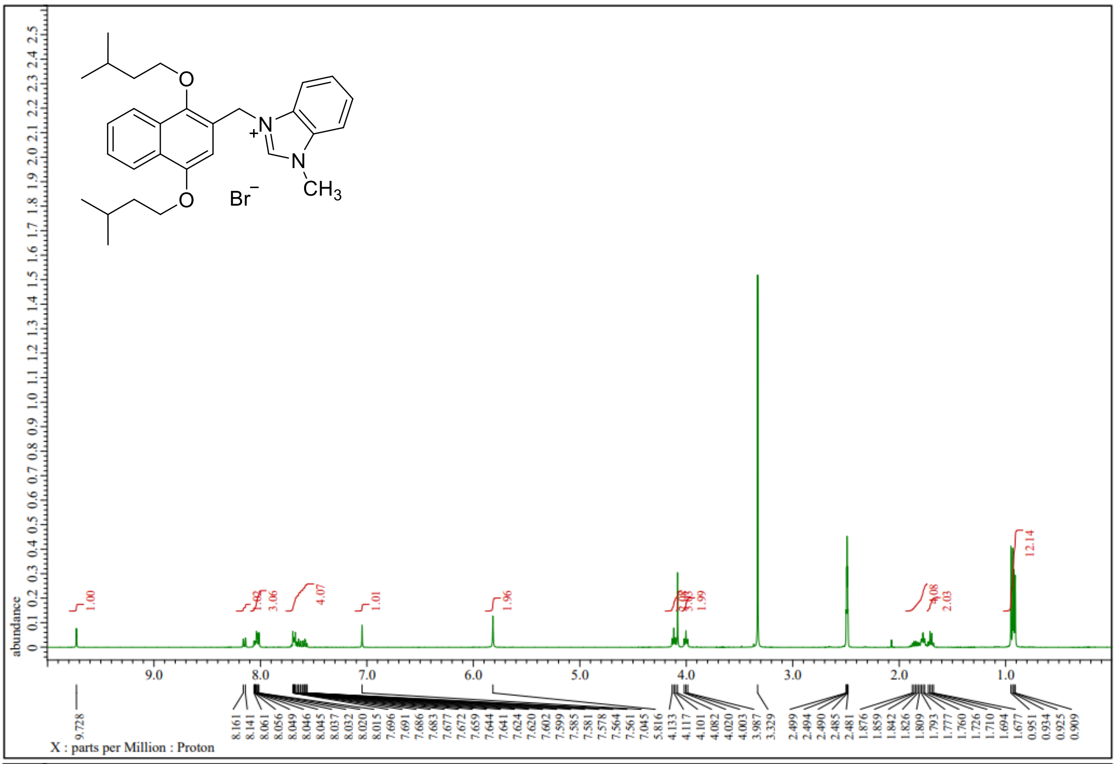

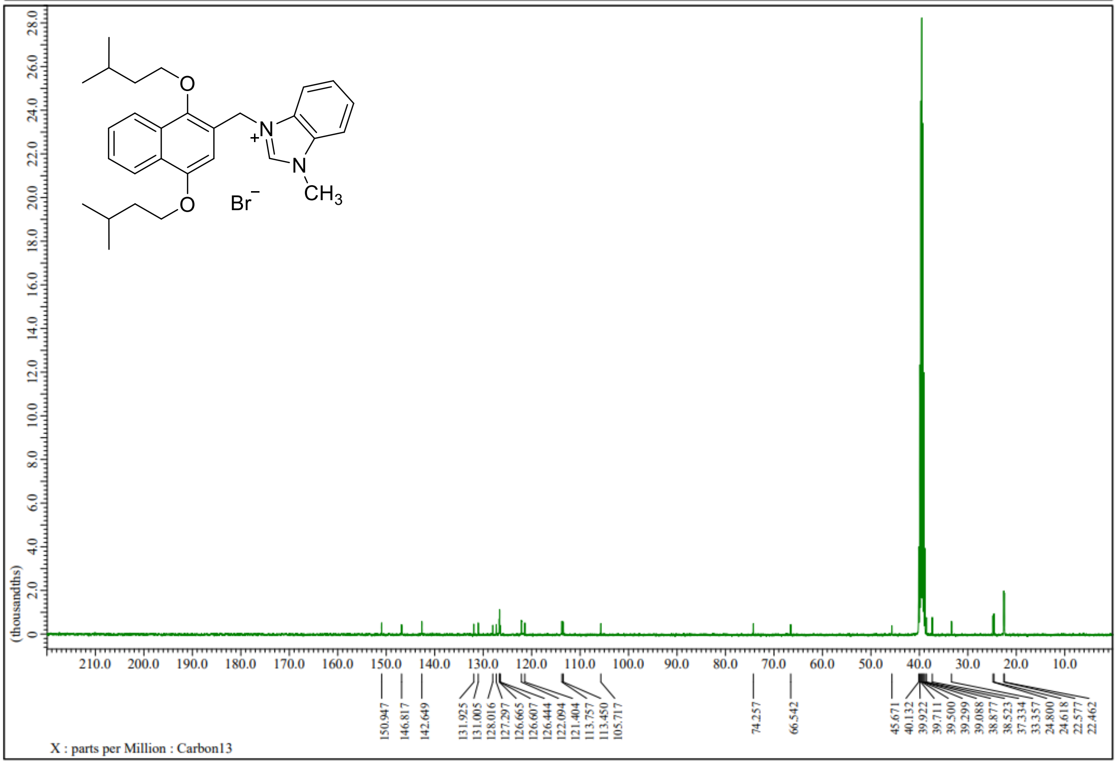

Figure S63. 3-((1,4-Bis(isoamyloxy)naphthalen-2-yl)methyl)-1-methyl-1*H*-benzo[*d*]imidazol-3-ium bromide **(IMS-21)**

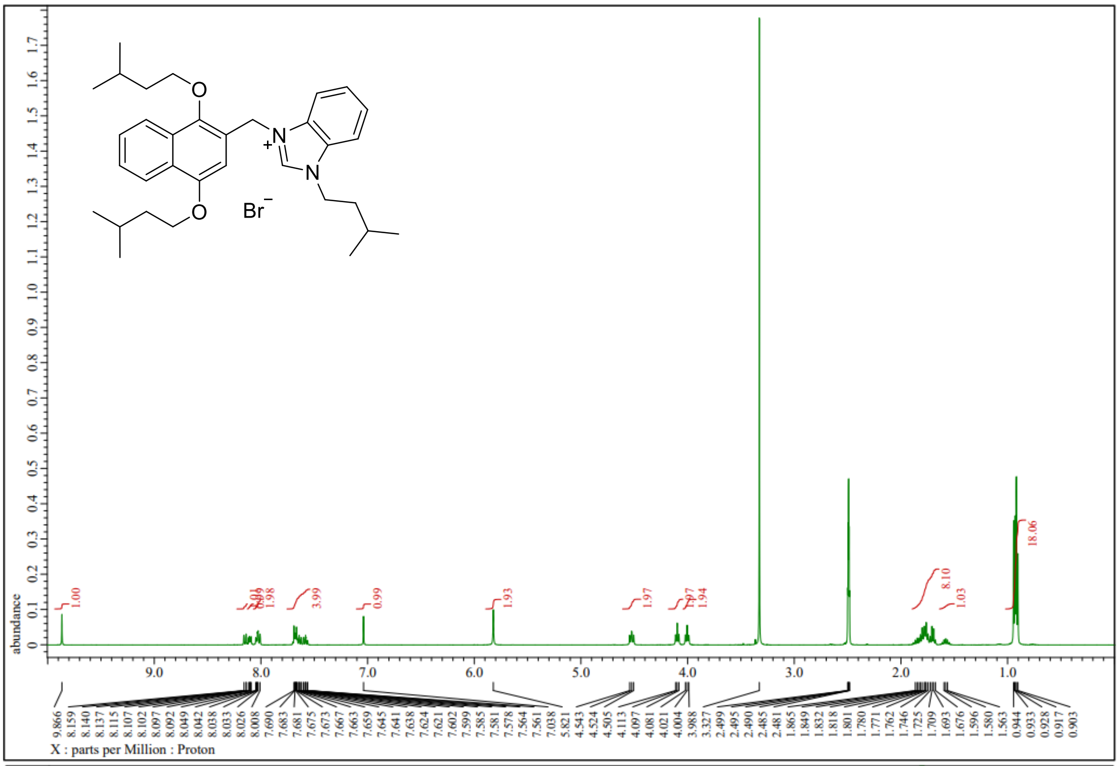

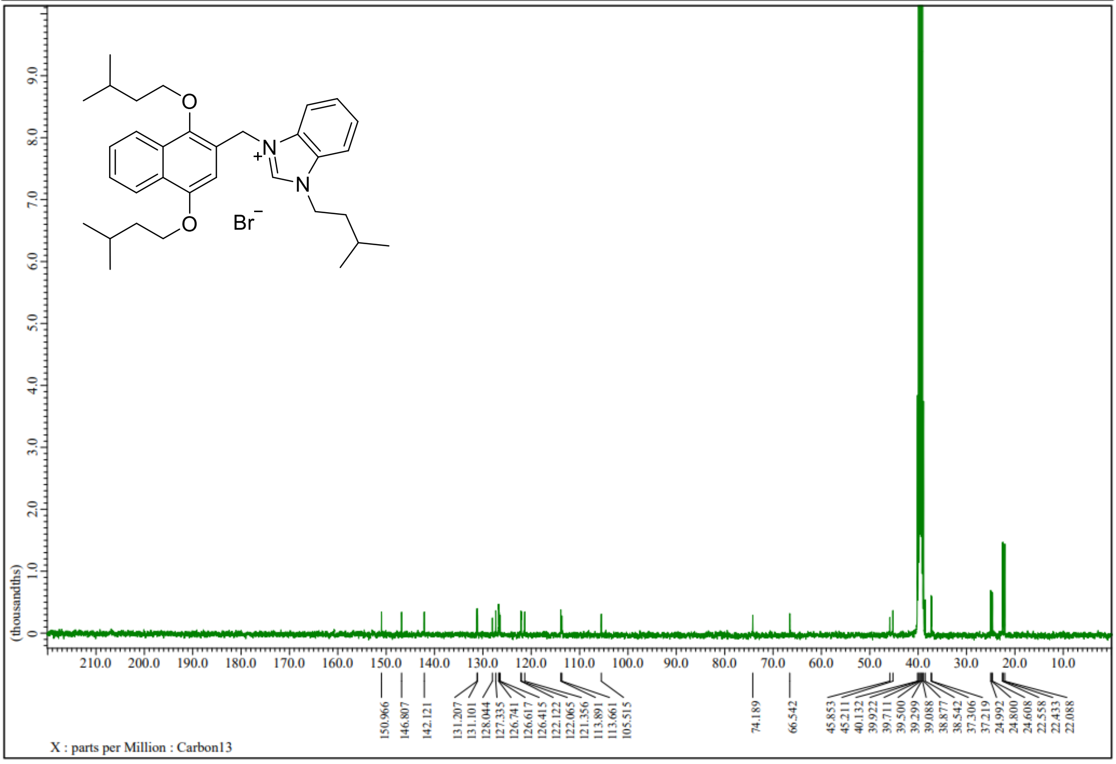

Figure S64. 3-((1,4-Bis(isoamyloxy)naphthalen-2-yl)methyl)-1-isoamyl-1*H*-benzo[*d*]imidazol-3-ium bromide **(IMS-22)**

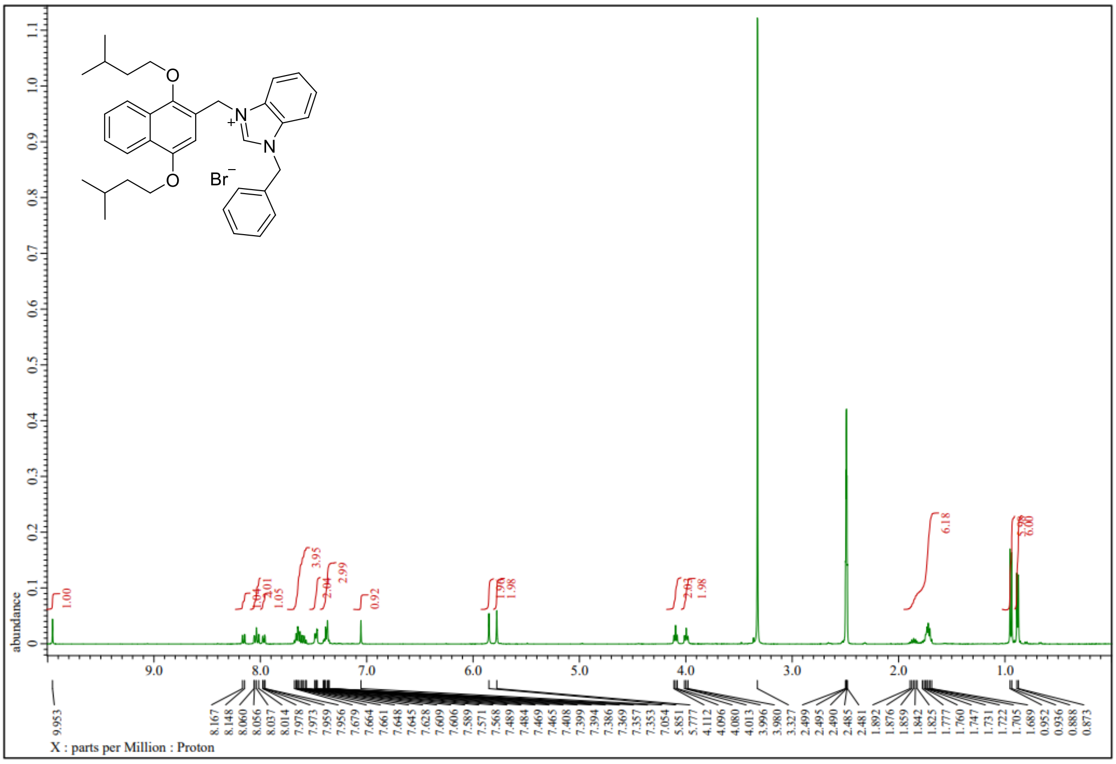

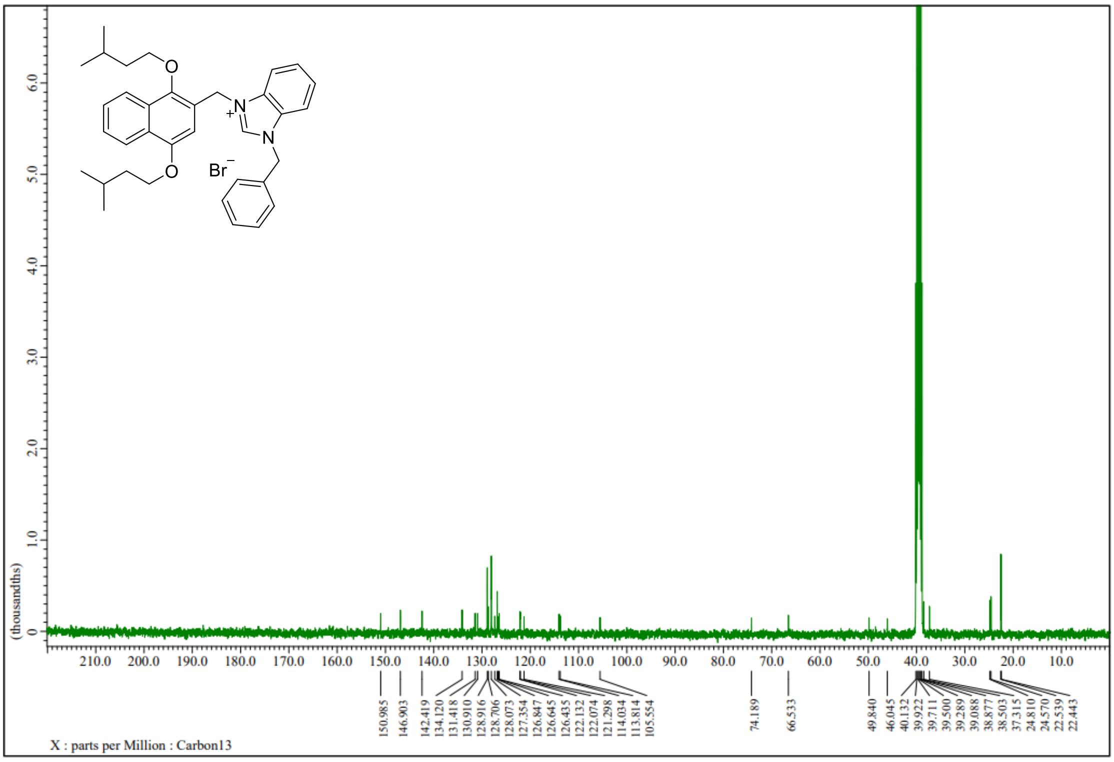

Figure S65. 1-**b**enzyl-3-((1,4-bis(isoamyloxy)naphthalen-2-yl)methyl)-1*H*-benzo[*d*]imidazol-3-ium bromide **(IMS-23)**

Ⅲ. Cell viability assay

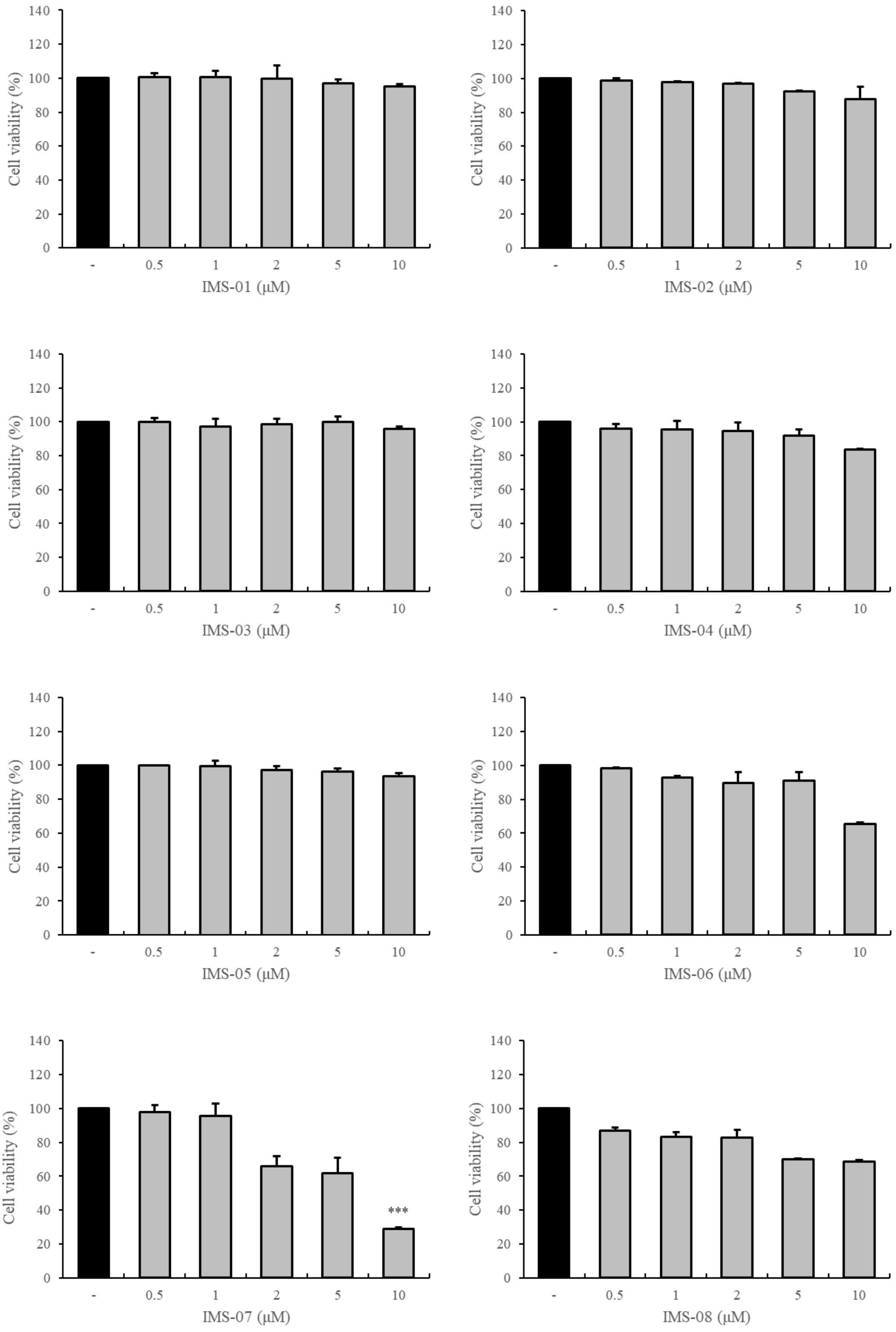

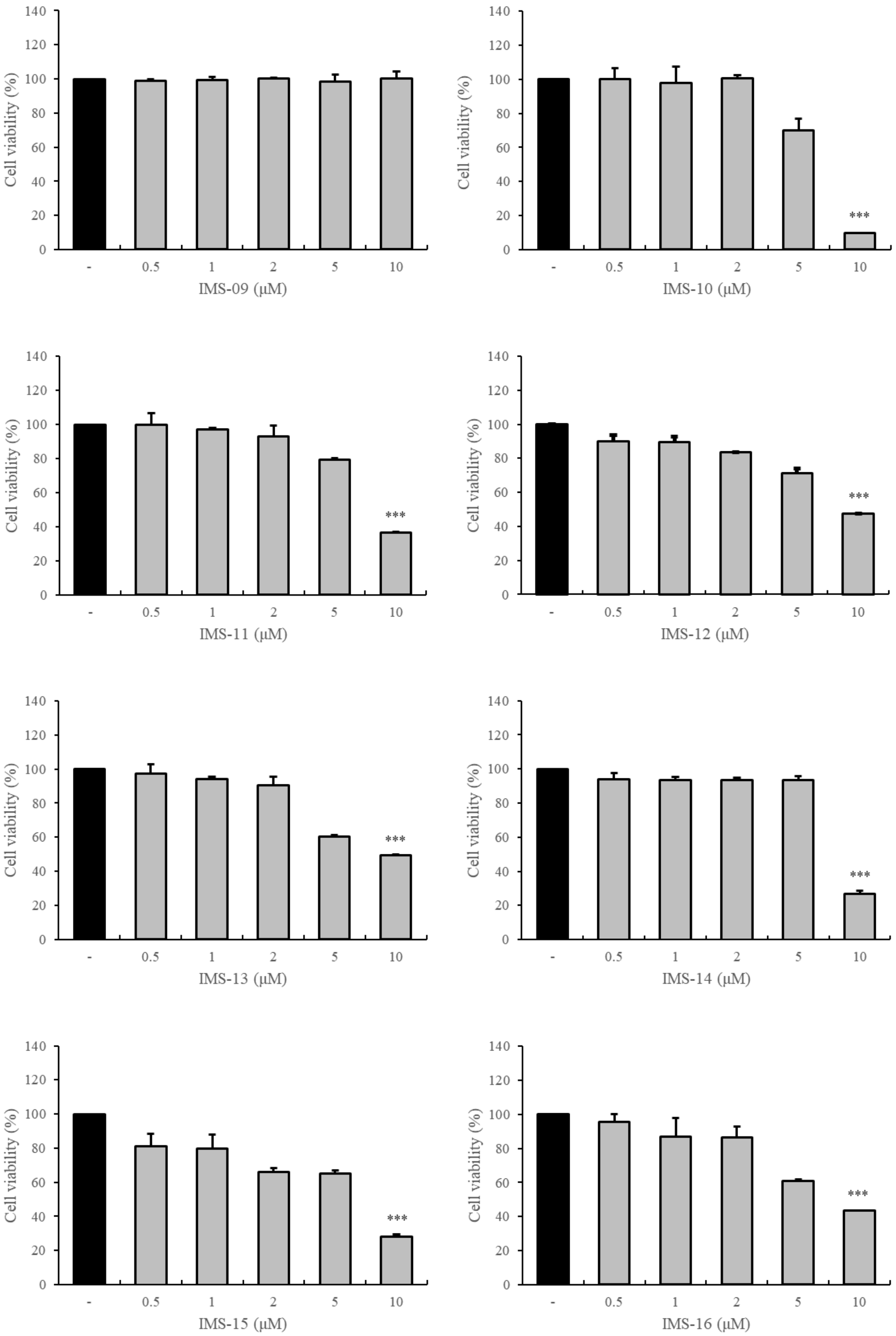

Cell viability (%)
IMS-09 (μM)
IMS-10 (μM)
IMS-11 (μM)
IMS-12 (μM)
IMS-13 (μM)
IMS-14 (μM)
IMS-15 (μM)
IMS-16 (μM)
- 0.5 1 2 5 10
0 20 40 60 80 100 120 140
***

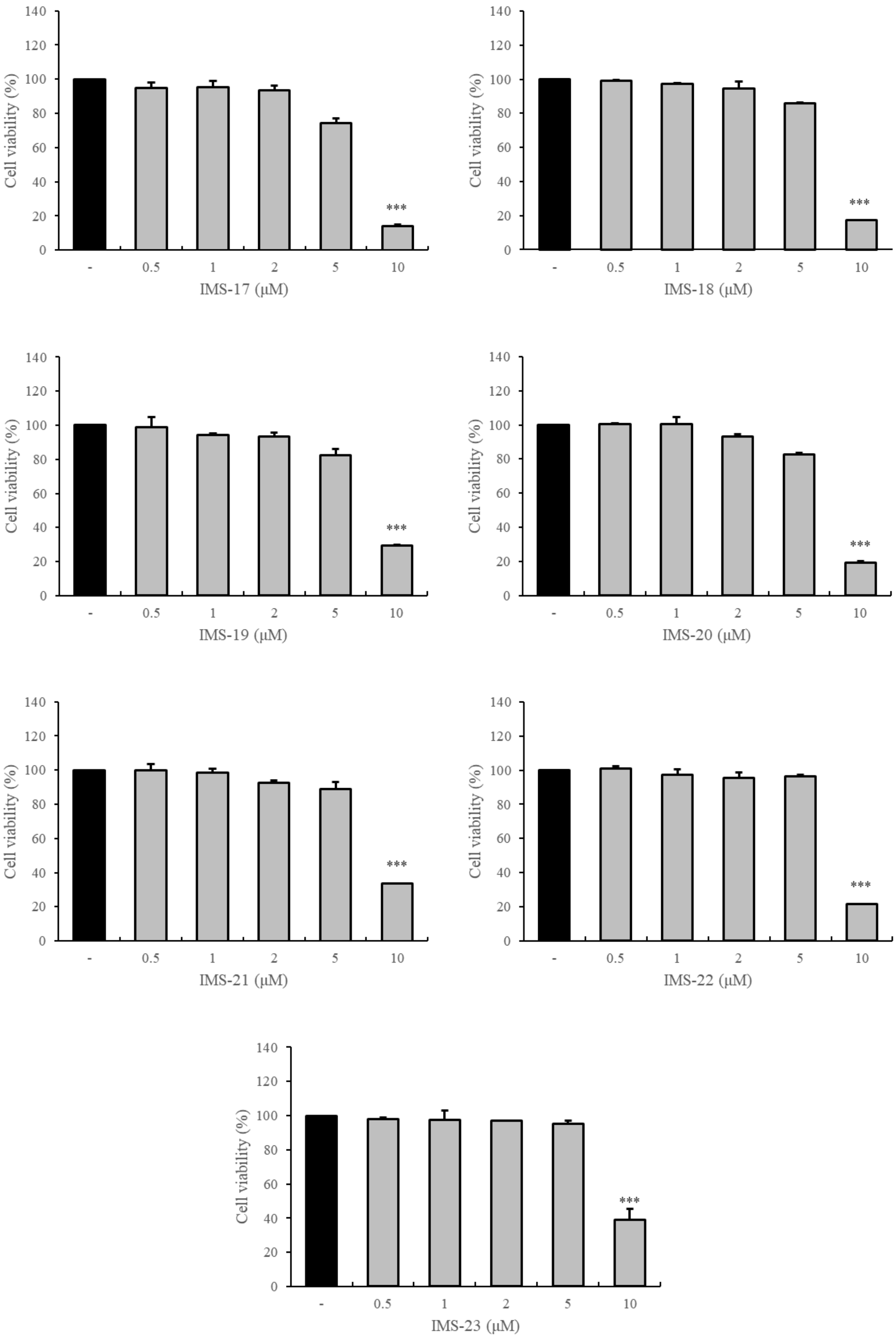

Figure S66. Cell viability of HepG2 cells exposed to 5 concentrations of **IMS-01**-**IMS-23**. (***$p \leq 0.001$)

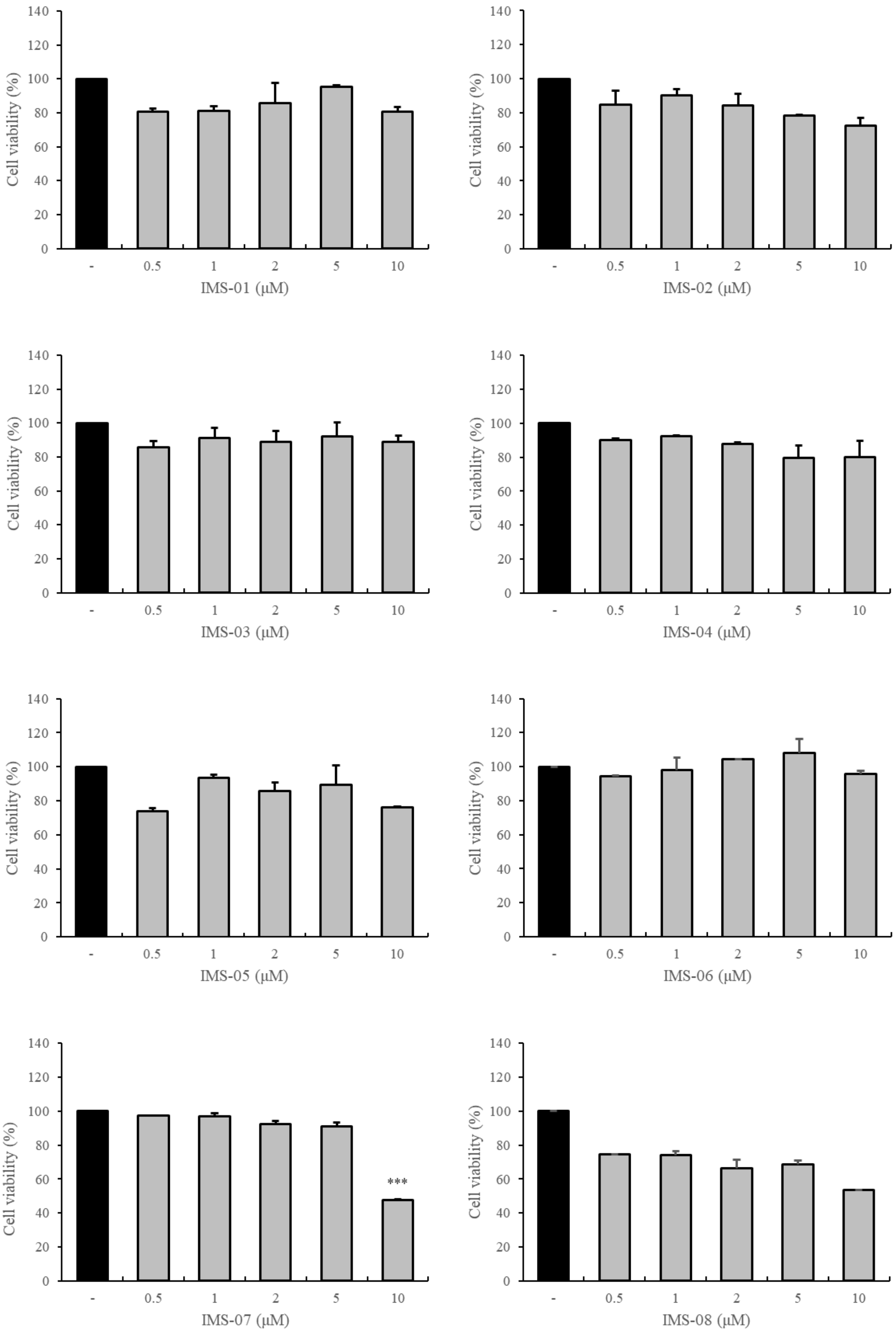
Cell viability (%)
0
20
40
60
80
100
120
140
-
0.5
1
2
5
10
IMS-01 (μM)
IMS-02 (μM)
IMS-03 (μM)
IMS-04 (μM)
IMS-05 (μM)
IMS-06 (μM)
IMS-07 (μM)
***
IMS-08 (μM)

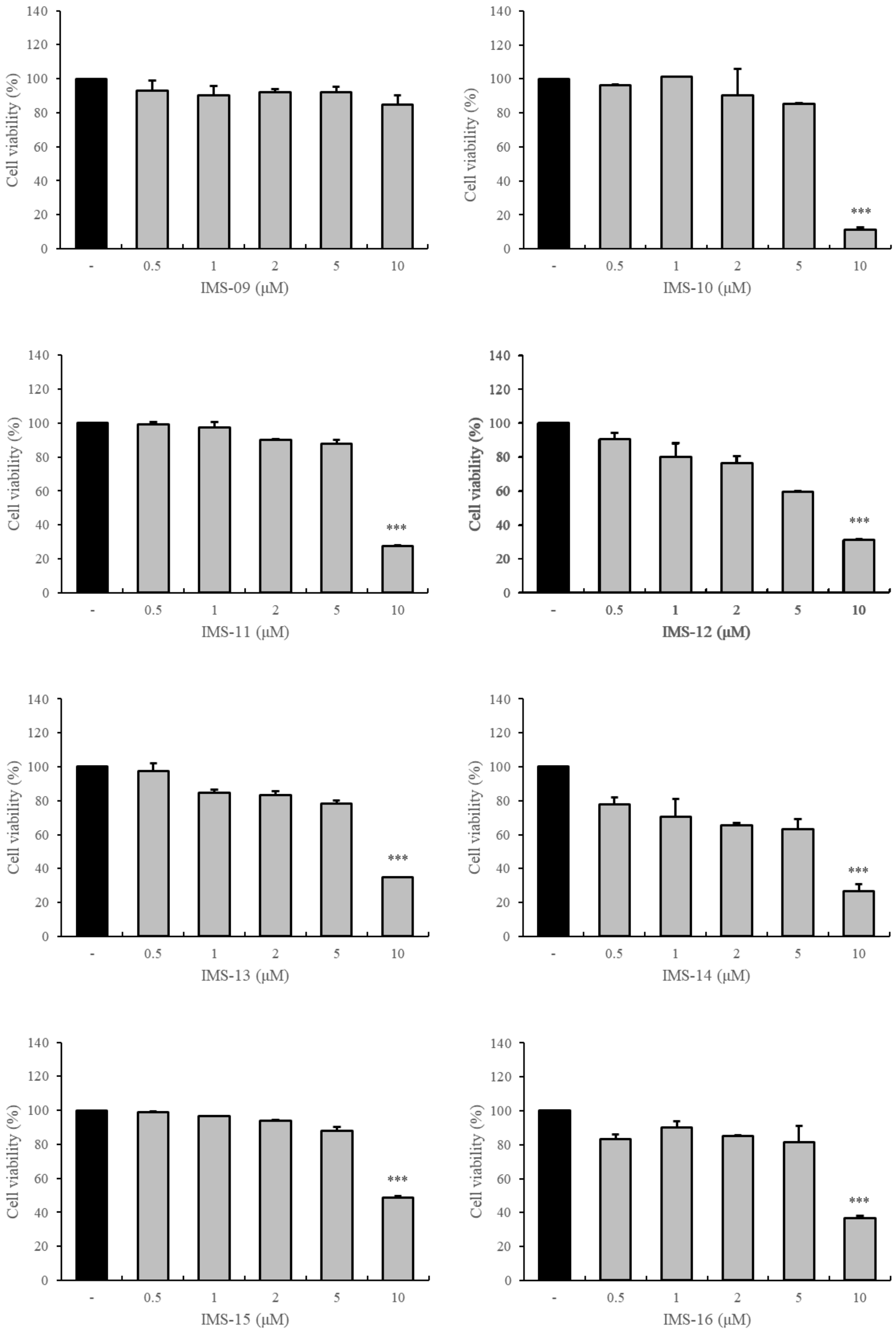
Cell viability (%)
IMS-09 (μM)
IMS-10 (μM)
IMS-11 (μM)
IMS-12 (μM)
IMS-13 (μM)
IMS-14 (μM)
IMS-15 (μM)
IMS-16 (μM)
- 0.5 1 2 5 10
0 20 40 60 80 100 120 140
***

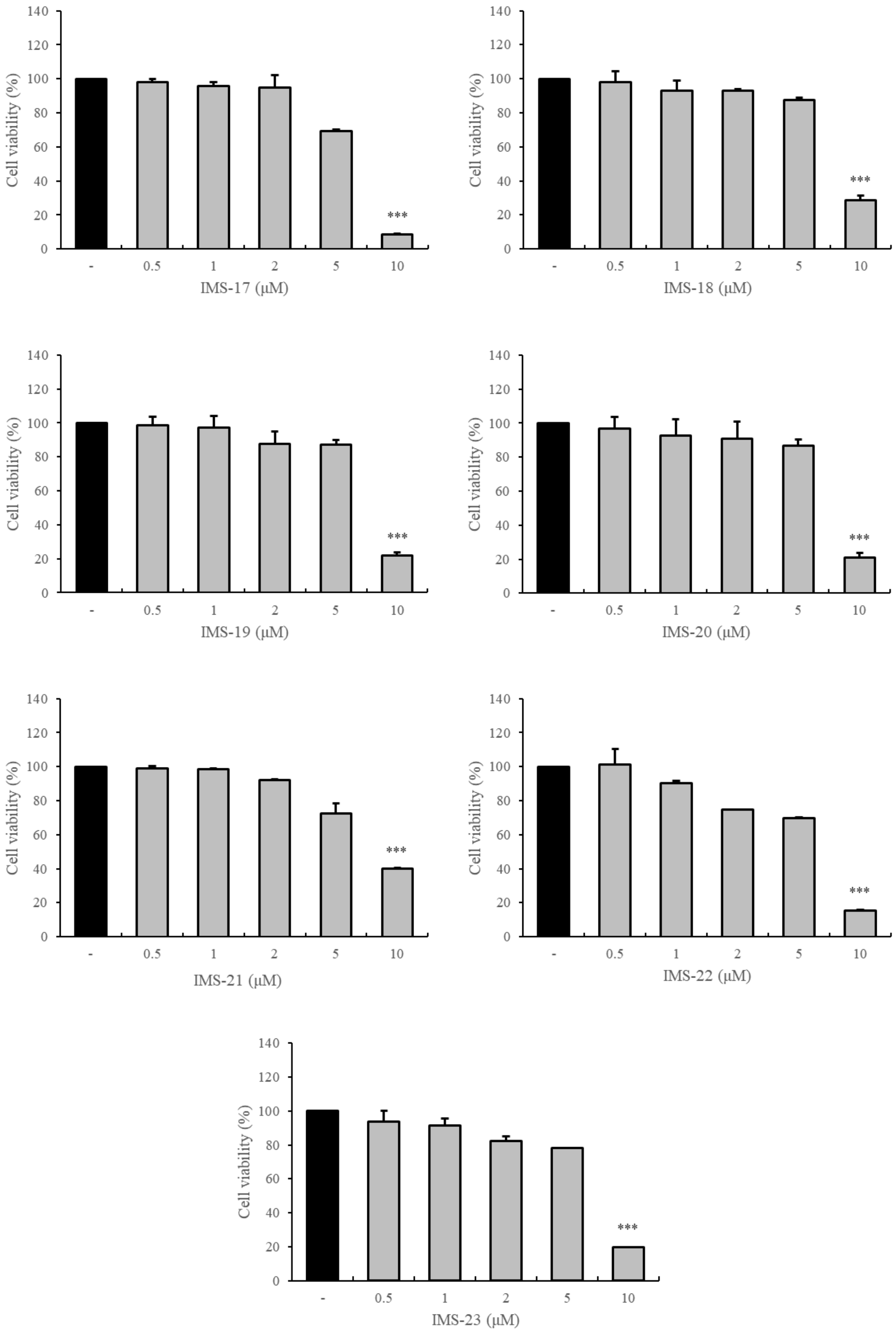

Figure S67. Cell viability of HT-29 cells exposed to 5 concentrations of **IMS-01**-**IMS-23** (****p*<0.001)

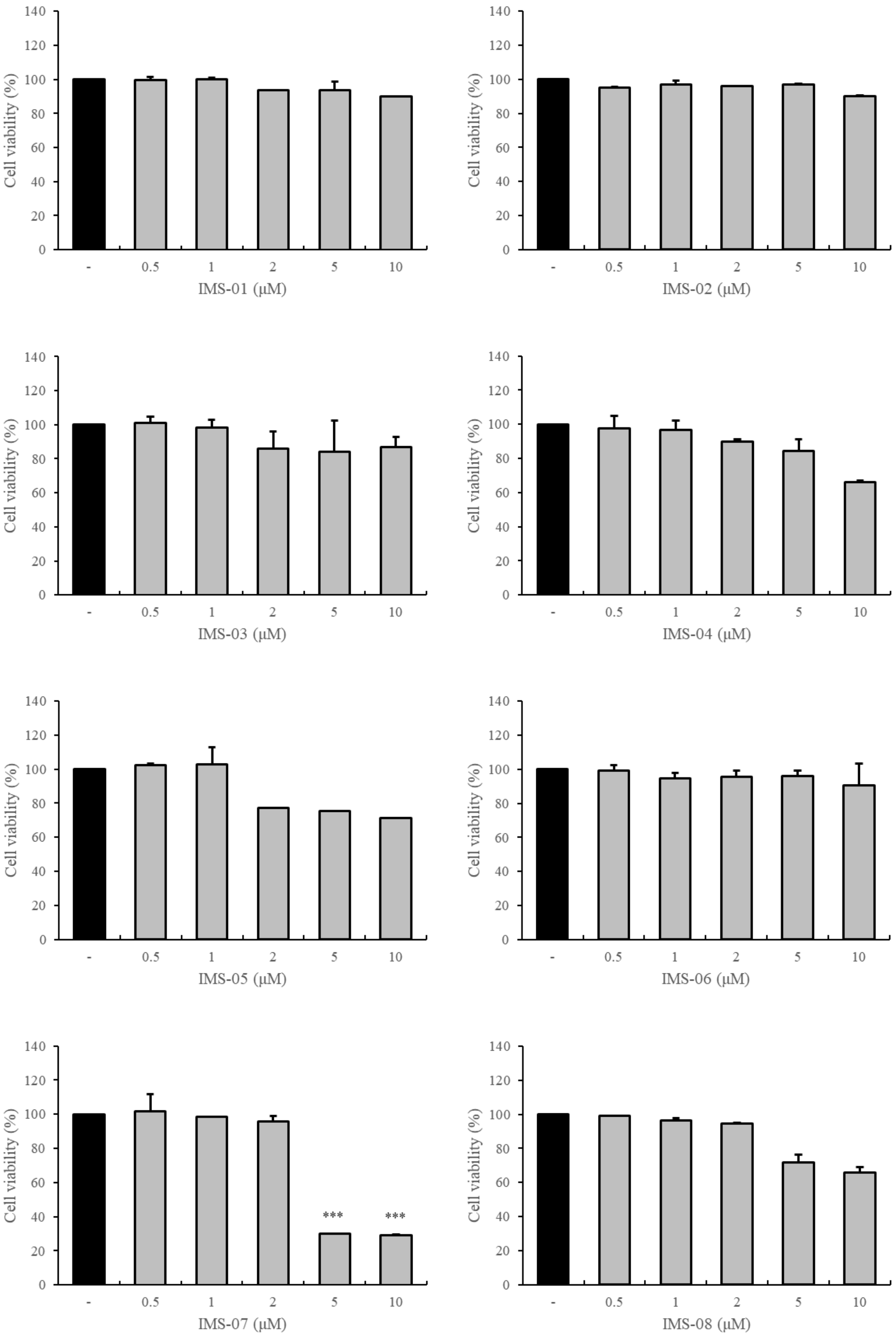
Cell viability (%)
140
120
100
80
60
40
20
0
-
0.5
1
2
5
10
IMS-01 (μM)
IMS-02 (μM)
IMS-03 (μM)
IMS-04 (μM)
IMS-05 (μM)
IMS-06 (μM)
***
***
IMS-07 (μM)
IMS-08 (μM)

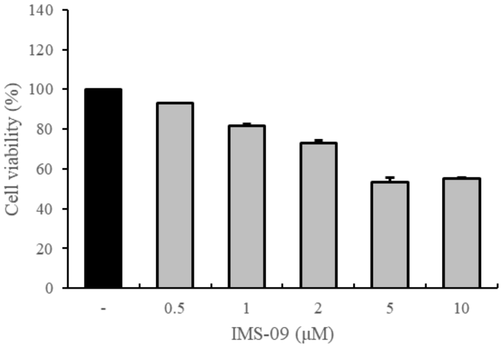
Cell viability (%)
0
20
40
60
80
100
120
140
-
0.5
1
2
5
10
IMS-09 (μM)

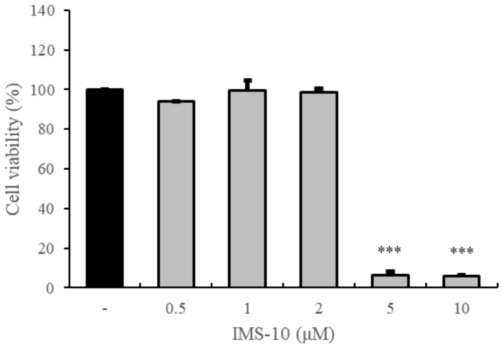
Cell viability (%)
0
20
40
60
80
100
120
140
***
***
-
0.5
1
2
5
10
IMS-10 (μM)

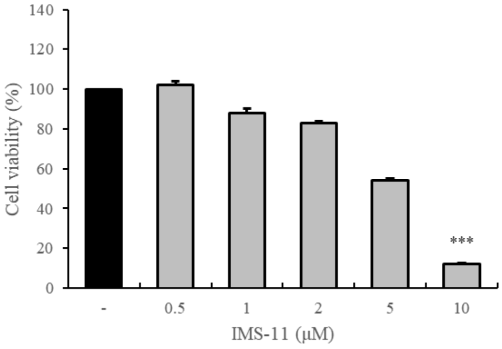
Cell viability (%)
0
20
40
60
80
100
120
140
***
-
0.5
1
2
5
10
IMS-11 (μM)

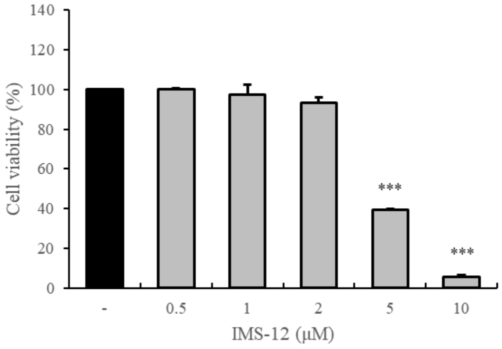
Cell viability (%)
0
20
40
60
80
100
120
140
***
***
-
0.5
1
2
5
10
IMS-12 (μM)

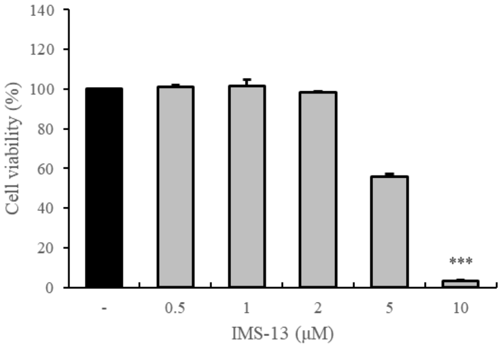
Cell viability (%)
0
20
40
60
80
100
120
140
***
-
0.5
1
2
5
10
IMS-13 (μM)

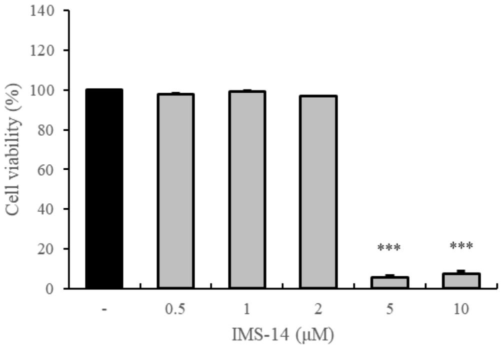
Cell viability (%)
0
20
40
60
80
100
120
140
***
***
-
0.5
1
2
5
10
IMS-14 (μM)

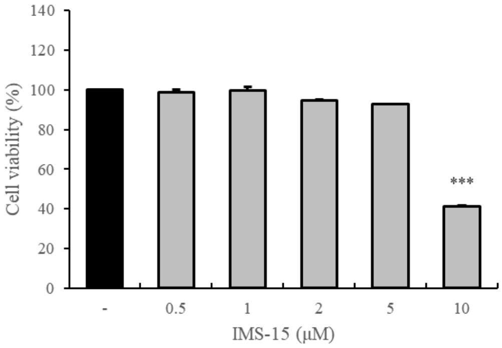
Cell viability (%)
0
20
40
60
80
100
120
140
***
-
0.5
1
2
5
10
IMS-15 (μM)

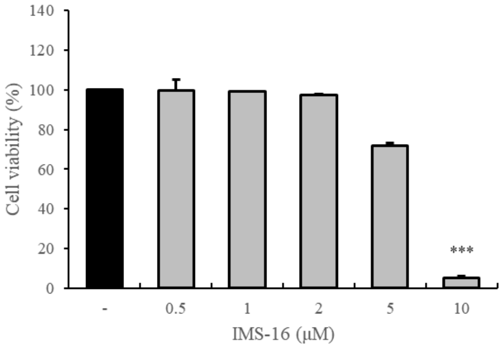
Cell viability (%)
0
20
40
60
80
100
120
140
***
-
0.5
1
2
5
10
IMS-16 (μM)

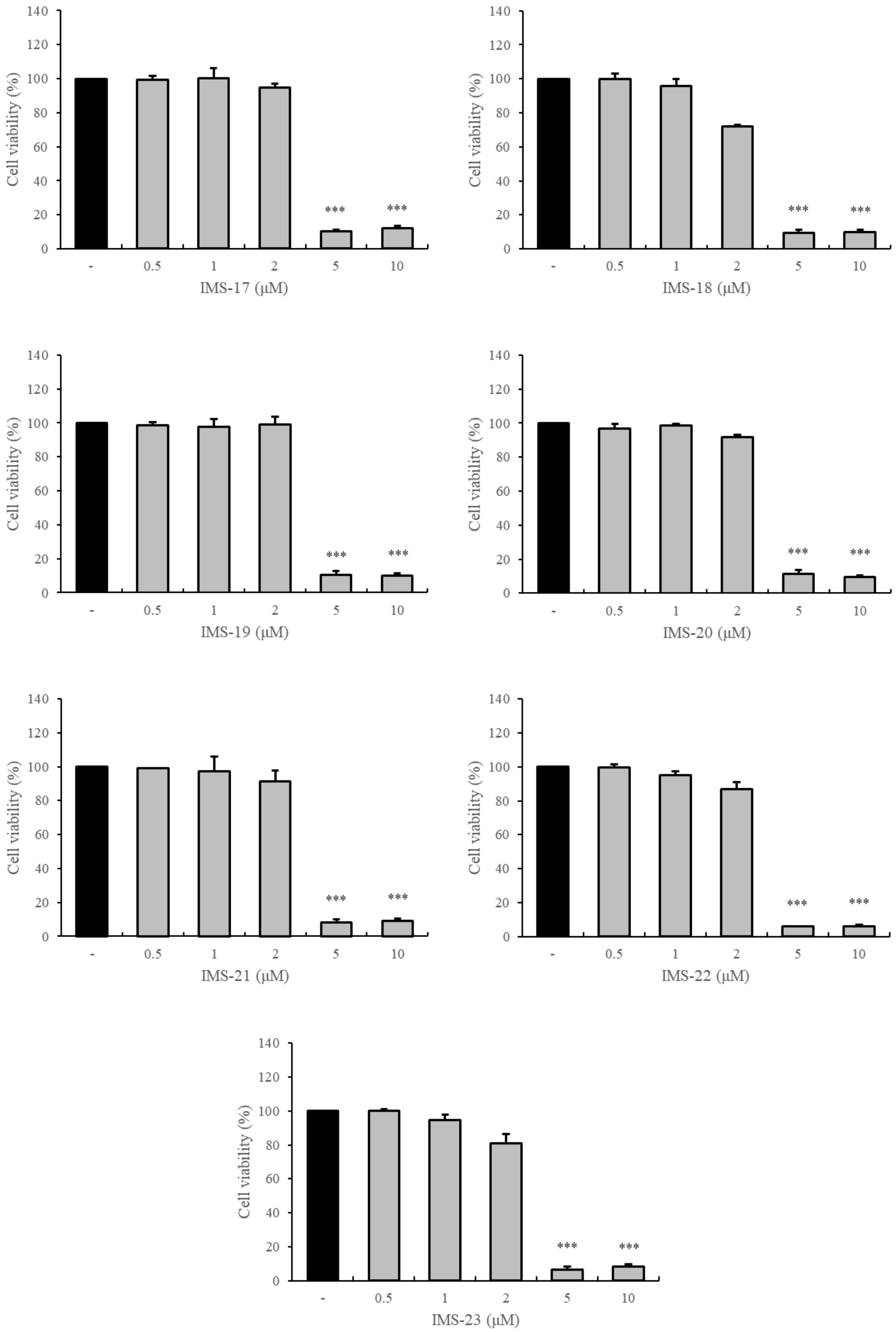

Figure S68. Cell viability of CCD-18Co cells exposed to 5 concentrations of **IMS-01-IMS-23**. (***p<0.001)
